# An Aminative Rearrangement of *O*‐(Arenesulfonyl)hydroxylamines: Facile Access to *ortho*‐Sulfonyl Anilines

**DOI:** 10.1002/anie.202204025

**Published:** 2022-07-07

**Authors:** Charlotte Morrill, James E. Gillespie, Robert J. Phipps

**Affiliations:** ^1^ Yusuf Hamied Department of Chemistry University of Cambridge Lensfield Road Cambridge CB2 1EW UK

**Keywords:** Arene Amination, Ion-Pairing, Non-Covalent Interactions, Radical Reactions, Regioselectivity

## Abstract

*Ortho*‐sulfonyl anilines are important building blocks for a range of applications. We report the discovery of an aromatic rearrangement reaction of *O*‐(arenesulfonyl)hydroxylamines which leads directly to *ortho*‐sulfonyl anilines through formation of a new C−N bond with excellent levels of regiocontrol for the *ortho* position(s) over all others. We establish that the rearrangement is proceeding through an intermolecular mechanism and propose that the regiocontrol observed is the result of attractive non‐covalent interactions occurring during the C−N bond‐forming step. Importantly, this method is complementary to classical aniline sulfonation in terms of the variously substituted regioisomers that can be obtained and it is also applicable to *O*‐(benzylsulfonyl) hydroxylamines.

## Graphical abstract

An aromatic rearrangement of hydroxylamine derivatives that leads directly to *ortho*‐sulfonyl anilines from simple starting materials is reported. This is achieved through formation of a new C−N bond with excellent levels of regiocontrol for the *ortho* isomer(s), which is proposed to be the result of attractive non‐covalent interactions occurring during the C−N bond‐forming step.

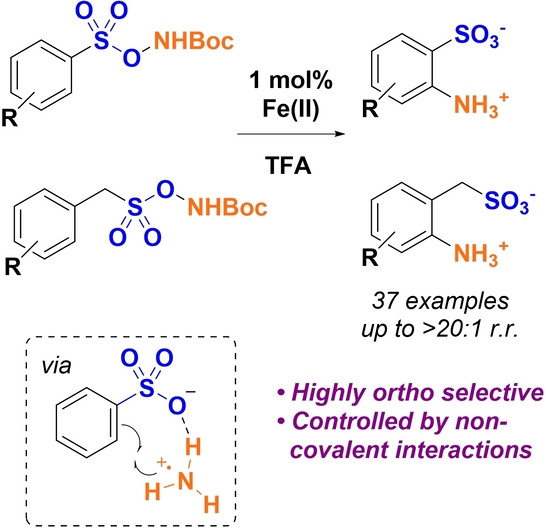

Anilines that bear adjacent sulfonyl functionality are important building blocks in the synthesis of medicinally relevant molecules (Figure 1A). When considering synthetic access to *ortho*‐sulfonyl anilines, by far the most common approach forms the C−S bond by direct sulfonation of an aniline (Figure 1B, upper).^[1]^ A variety of transformations can then be carried out on the sulfonate to access functional groups such as sulfonamides and sulfones, both ubiquitous in medicinal chemistry.^[2]^ Whilst direct, this process uses very harsh conditions and will only give access to the *ortho* isomer reliably if the *para* position is blocked, drastically restricting available substitution patterns for these important building blocks. Indeed, for a monosubstituted aniline, three of the four hypothetical regioisomers resulting from direct sulfonation are wholly or partially inaccessible using a classical sulfonation approach (Figure 1B, lower).^[3]^ In this work we disclose a complementary disconnection that provides rapid access to this important class of compounds, which installs the C−N bond rather than the C−S bond and does so through a serendipitously discovered rearrangement reaction of arenesulfonyl *N*‐*O* reagents (Figure 1C). The starting materials are easily accessed in a single step from aryl sulfonyl chlorides, of which thousands are commercially available. Of the three constitutional isomers challenging to obtain through direct sulfonation, two are now fully accessible using this complementary approach.

**Figure 1:**
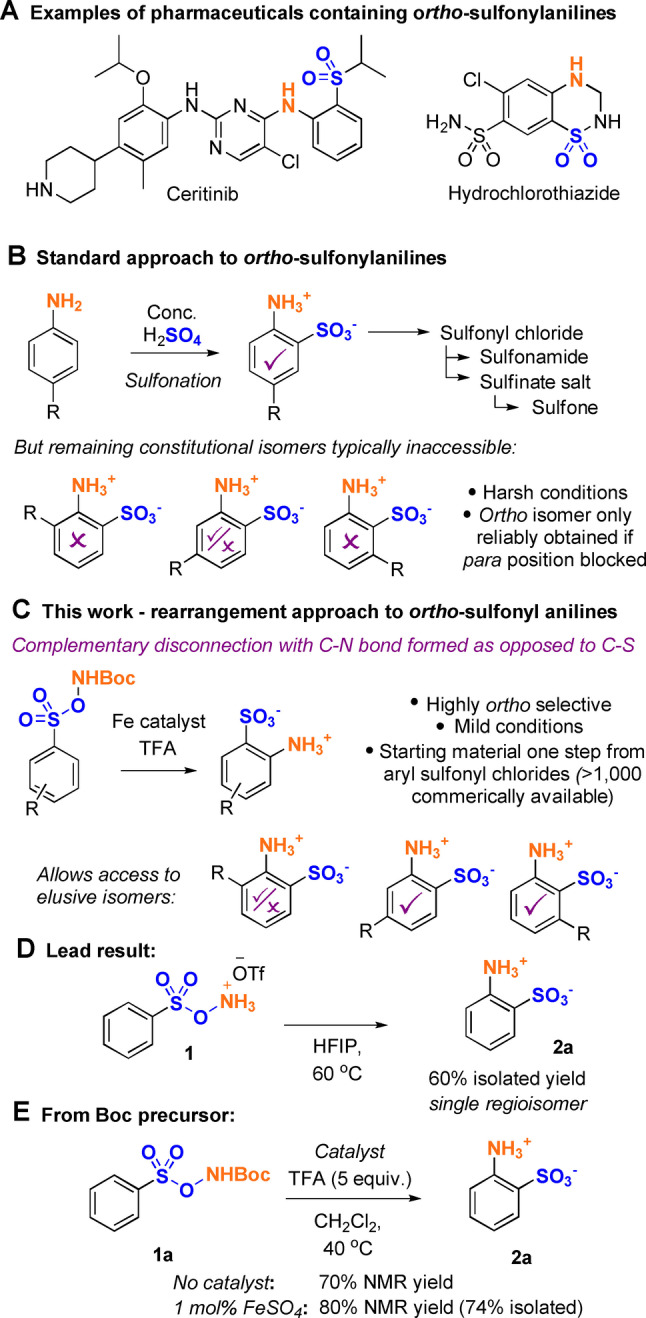
Introduction and initial results.

Inspired by previous reports of iron‐catalyzed arene amination using hydroxylamine‐derived aminating agents[^4^, ^5^, ^6^] but focusing on the significant regioselectivity challenges associated with this chemistry, we recently developed an *ortho*‐selective amination of aniline‐derived sulfamate salts.^[7]^ In that work, *O*‐acyl hydroxylamine reagents were used as the nitrogen source and we proposed that the excellent *ortho* selectivity arose due to attractive non‐covalent interactions between the anionic substrate and the incoming aminium radical. During optimization, we also evaluated the related *O*‐benzenesulfonyl hydroxylamines and observed an unexpected byproduct when the triflate salt of *O*‐benzenesulfonyl hydroxylamine (**1**) was evaluated.^[4e]^ Scrutinization of this by‐product suggested that it occurred through an apparent rearrangement process involving “self‐amination” and we were surprised to discover that simply stirring **1** in hexafluoroisopropanol (HFIP) at 60 °C led to aminated product **2 a** in good yield and with outstanding selectivity for the *ortho* isomer over all others (Figure 1D). The product exists as a zwitterionic salt and could be purified by simple precipitation. Isolated arenesulfonate‐derived *N*‐*O* reagents are established to be prohibitively unstable if the nitrogen atom is unsubstituted with only a few exceptions such as *O*‐mesitylenesulfonyl hydroxylamine (MSH), which remains challenging to handle.^[8]^ In most cases spontaneous decomposition occurs, presumably through facile cleavage of the weak N−O bond. *N*‐Protonation, as in **1**, typically increases stability to some degree and related triflate salts have been investigated as reagents in a handful of recent studies.[^4e^, ^9^] We found **1**, and closely related analogues, not to be reliably stable and that their synthesis could be capricious. We therefore investigated their formation in situ from the stable *N*‐Boc precursors, which are trivially accessed through the coupling of *N*‐Boc hydroxylamine with aryl sulfonyl chlorides. Gratifyingly, after optimization (see Supporting Information for details) we found that we could simply stir the *N*‐Boc precursor **1 a** in CH_2_Cl_2_ at 40 °C with five equiv. of trifluoroacetic acid (TFA) and the same rearrangement product **2 a** could be obtained in 70 % NMR yield as a single regioisomer (Figure 1E). This could be increased to 80 % by addition of 1 mol % of FeSO_4_ and the zwitterionic product was isolated in 74 % yield by simple precipitation.

We then proceeded to evaluate the scope of the rearrangement, the starting materials all obtained in a single step from commercially available benzenesulfonyl chlorides (Scheme 1). We were pleased to find that the reaction could be successfully applied to arenes bearing a broad range of functionalities. One or two alkyl substituents were well tolerated at various positions of the ring (**2 b**–**2 h**), providing the *ortho* isomer(s) of the desired products with high selectivities over *meta* and *para*. Substrates bearing halogens could also undergo the aminative rearrangement in moderate to good yields, including fluorines (**2 i**–**2 k**), chlorine (**2 l**–**2 q**) and bromine (**2 r**, **2 s**). In cases where the substrate has two available *ortho* positions, the product was obtained as a mixture of these, typically with some preference for functionalization at the less hindered position. In some cases, on purification only the major regioisomer was obtained due to solubility differences and we display the crude isomeric ratio in parentheses alongside the isolated ratio and yield. One (**2 t**, **2 u**) or two (**2 v**) electron‐donating methoxy groups could be incorporated. Interestingly, in the case of **2 t**, an approximately 1 : 1 ratio of regioisomers was obtained wherein the reaction also occurred adjacent to the methoxy group, suggesting that strong electron donating groups are able to override the *ortho*‐selectivity (see later discussion). A similar effect was observed with **2 u**. A naphthalene‐based substrate also worked well and a single *ortho* regioisomer could be isolated after precipitation of the zwitterion, despite a 1 : 1 mixture of the two possible *ortho* regioisomers being initially obtained (**2 w**). Importantly, the rearrangement is not limited to electron‐rich or neutral substrates: as well as a trifluoromethoxy group (**2 x**), variously trifluoromethyl‐substituted (**2 y**, **2 za**) substrates and an ester‐substituted (**2 z**) precursor smoothly participated in the rearrangement reaction, as did a substrate bearing a sulfone (**2 zb**). Finally, we discovered that even a nitro group could be accommodated, although to get appreciable yield in this case it was necessary to use the triflate salt of the arenesulfonyl N−O reagent under the conditions described in Figure 1D, rather than the Boc‐protected precursor (**2 zc**, see Supporting Information for details). In examples where yields are moderate this is typically due to incomplete conversion and not due to formation of isomers other than those depicted.

**Scheme 1:**
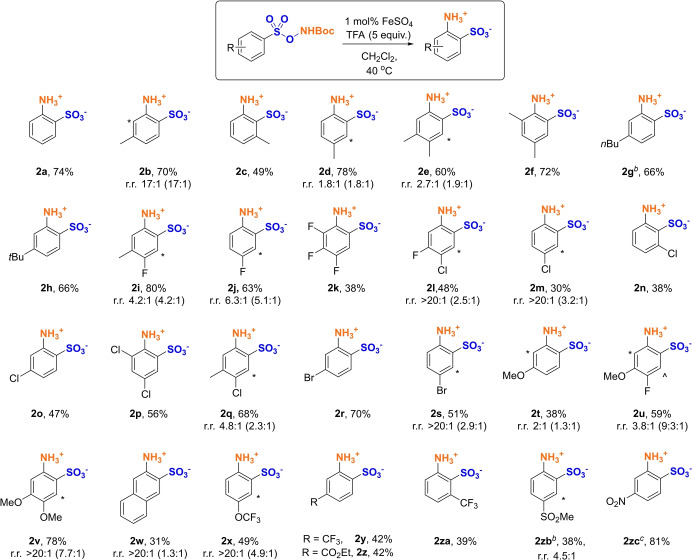
[a] Yields are isolated. Ratio (r.r.) refers to regioisomeric ratio of two observed regioisomers, minor indicated with (*), in isolated material. Ratio in parenthesis refers to regioisomeric ratio determined from crude material, prior to purification. For **2 u** a third regioisomer indicated with ( ) was identified in the crude material only. [b] Reaction run without iron catalyst. [c] Starting material was isolated triflate salt of N−O reagent, reaction conditions HFIP (0.2 M), 60 °C (see Supporting Information for details)

We next questioned whether the reaction would tolerate the addition of a methylene between the arene and the sulfonate group (Scheme 2, **3**). If successful, this would allow access to substituted anilines with a methylenesulfonyl linkage at the *ortho* position, compounds that can be directly transformed to 5‐membered sultams, which have important applications in medicinal compounds.^[10]^ Gratifyingly, these substrates also proved to be amenable to the aminative rearrangement, giving exclusively the *ortho* product(s) under the same conditions, the unsubstituted example giving 83 % yield (**4 a**). Chlorine (**4 b**, **4 c**), electron‐donating groups (**4 d**, **4 e**), bromines (**4 f**, **4 g**), fluorine (**4 h**) as well as an electron‐withdrawing trifluoromethyl group (**4 i**) were all tolerated and gave access to the desired products. As previously, if two inequivalent *ortho* positions are present, both isomers are obtained (as in **4 g**).

**Scheme 2:**
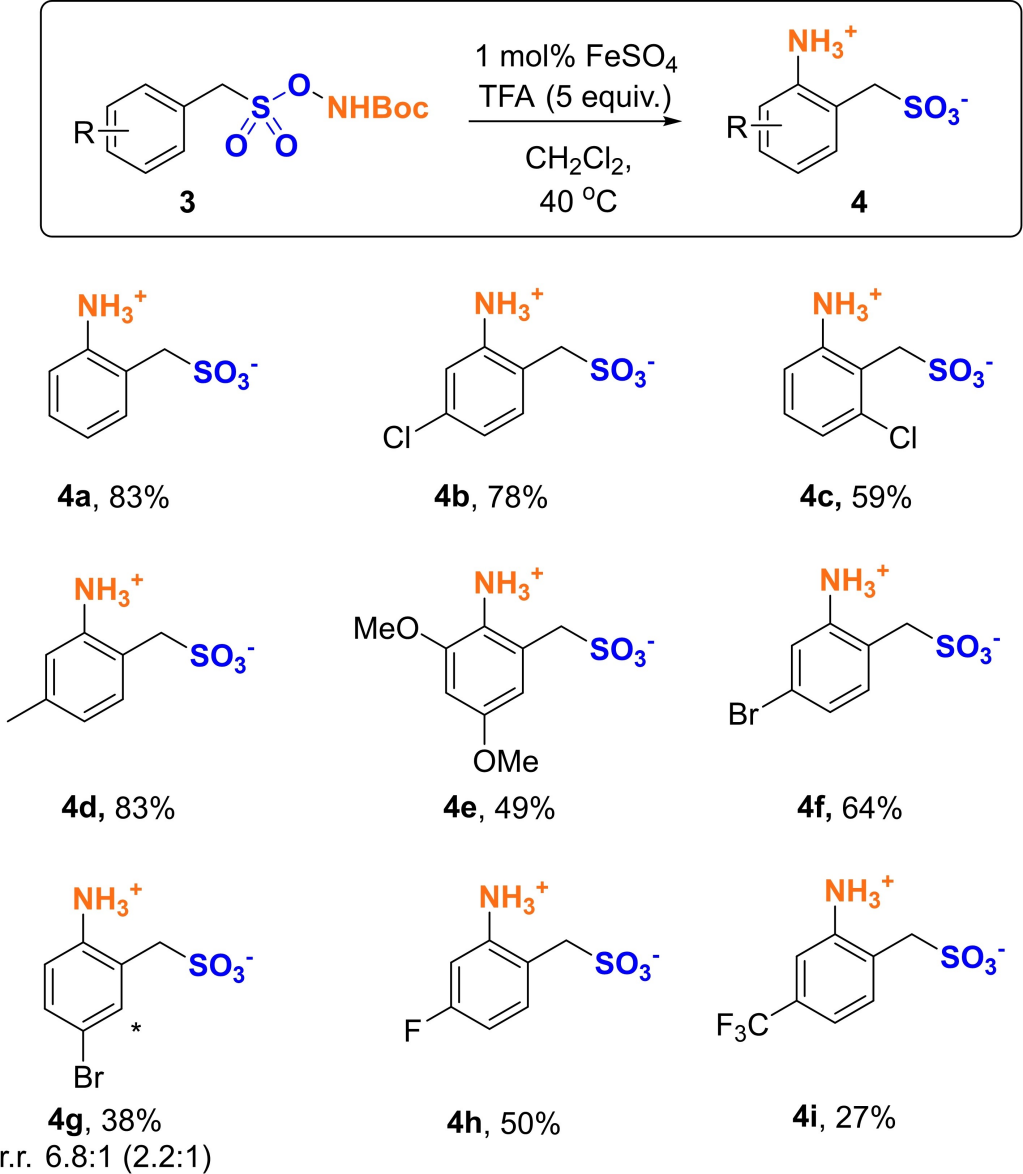
[a] Yields are isolated. Ratio (r.r.) refers to regioisomeric ratio of two observed regioisomers, minor indicated with (*), in isolated material. Ratio in parenthesis refers to regioisomeric ratio determined from crude material, prior to purification.

An important mechanistic question relating to aromatic rearrangements is whether they occur by an intermolecular or intramolecular mechanism, often with important implications for the origins of regioselectivity.^[11]^ One potential explanation for the excellent observed *ortho*‐selectivity in our reaction would be that the rearrangement proceeds via an intramolecular mechanism and so results in proximal arene functionalization. A crossover experiment would be the most effective way to address this question but in order to achieve this, two different nitrogen‐based groups would need to be transferred to two different aryl sulfonates.^[12]^ To this end we first established that transfer of NHMe is viable from *N*‐methylated starting material **5 a**,^[9b]^ although the yield of product was low (see Supporting Information for details). Nevertheless, it provided a basis for the crossover experiment in which **5 a** and **1 b** are subjected to the standard reaction conditions and the product distribution is analyzed (Scheme 3A). The outcome was that all four possible product combinations were indeed observed, showing that there is crossover between the two reagents and strongly suggesting that the reaction proceeds via an intermolecular mechanism. To gain further support for this, we reacted an independently synthesized tetrabutylammoium sulfonate salt **6** with separate mesityl aminating agent **7 a**, which is blocked at the *ortho* positions and so should not undergo self‐amination (Scheme 3B). This gave amination of **6** in low yield (11 %), which could be improved by use of the methanesulfonyl aminating agent **7 b** (49 %).[^4d^, ^4f^] In both cases, the *ortho* isomer was produced exclusively, providing further support for the rearrangement proceeding through an intermolecular pathway, given the same unprecedented selectivity outcome observed in both cases.

**Scheme 3:**
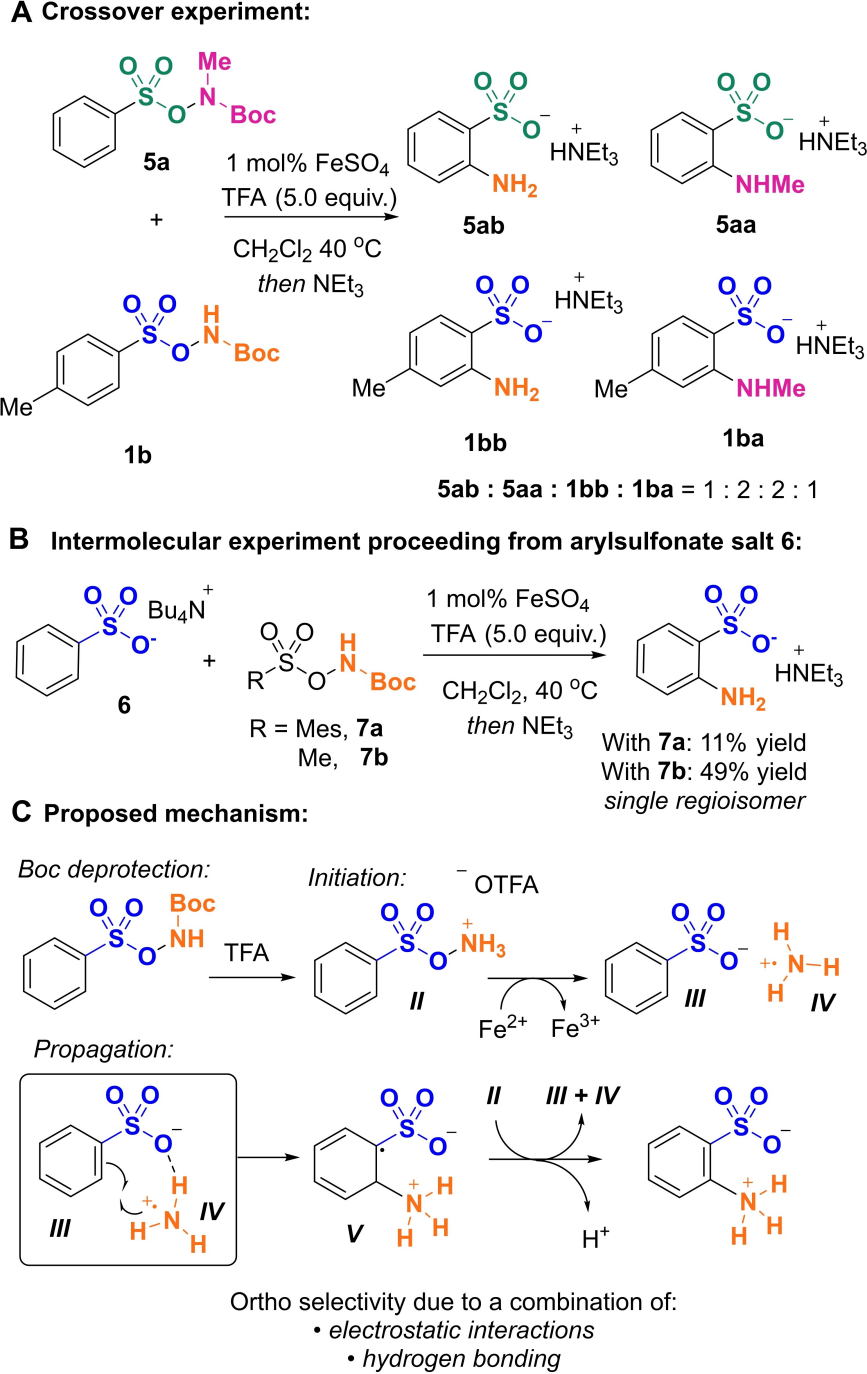
Experiments to probe mechanism.

Based on precedent we envisage that the reaction proceeds via a radical mechanism[^4d^, ^4e^, ^4f^, ^5g^, ^9b^] and tentatively propose that the most likely form of this is a radical chain process,^[13]^ initiated by cleavage of the weak N−O bond that intermediate ***II*** possesses once the Boc group is removed (Scheme 3C).[^5c^, ^5d^, ^5e^] Thermal homolytic cleavage under relatively mild conditions has been shown to be viable for related *O*‐methanesulfonyl hydroxylamines.^[4f]^ For the *O*‐arylsulfonyl hydroxylamines used in our study, the barrier to this cleavage is likely to be even lower, as implied by their lower stability in isolated triflate salt form. It is likely that in most cases sufficient initiation for productive chain propagation can occur without the addition of iron. However, in some cases addition of iron facilitates the N−O bond cleavage and ensures a productive chain, increasing product yield.^[14]^ We evaluated a selection of substrates from the scope with and without iron added found that the beneficial effect of adding iron varied from substrate to substrate (see Supporting Information). N−O bond cleavage in ***II*** produces the key electrophilic aminyl radical ***IV*** which can enter the putative propagation cycle through reaction with sulfonate anion ***III***. The resulting cyclohexadienyl radical ***V*** can then regenerate the starting components of the propagation sequence, ***III*** and ***IV***, by electron transfer to ***II***, prior to rearomatization to give the product. We anticipate that a combination of ion‐pairing and hydrogen bonding interactions between the sulfonate group and the incoming radical would direct it to the *ortho* position in a manner analogous to that proposed in our previous work on the amination of aniline‐derived sulfamate salts.^[7]^ Our observation that a *para*‐methoxy substituent results in a 1.3 : 1 mixture of aminated isomers (*ortho* and *meta*) suggests that these non‐covalent interactions are not strong enough to override directing effects from the most strongly electron‐donating substituents (Scheme 1, **2 t** and **2 u**).

The *ortho*‐amino benzene sulfonates obtained in Scheme 1 can be further transformed in a variety of useful ways and we demonstrate these on the parent 2‐aminobenzenesulfonic acid (Figure 2A). The sulfonate can be transformed to a sulfonyl chloride, even in the presence of the aniline (**8 a**), and then on to a sulfonamide (**8 b**). The amine functionality can be protected with an acetyl group (**8 e**) or can be transformed to an iodide through a Sandmeyer reaction (**8 d**), both in the presence of the sulfonic acid. An important elaboration of the zwitterionic sulfonates is that they can be converted to sodium sulfinate salts, nucleophilic at sulfur, which can then undergo a range of further chemistry.^[15]^ This proceeded in two steps to give sulfinate salt **8 f** which could then be arylated with a diaryliodonium salt to give diarylsulfone **8 g**.^[16]^ Analogously, the sulfonyl chloride could be converted in two steps, via the sulfinate salt, to the methyl sulfone through alkylation (**8 c**). For the *ortho*‐amino benzyl sulfonates obtained in Scheme 2, these can be readily converted to the corresponding sultams and we demonstrate this on **4 f** to highlight the complementarity of our method to existing approaches to these molecules (Figure 2B).^[10]^ The resulting 6‐bromosultam **9** has not been reported in the literature, in contrast to the 5‐isomer which has been used in a number of medicinal chemistry campaigns (inset box).^[17]^ The 5‐isomer can be readily obtained through electrophilic bromination and seems likely that poor synthetic accessibility has prevented the 6‐isomer from being explored.^[18]^ Finally, to further stress the practical utility of our method, we compare synthetic access to all four methylated regioisomers of 2‐aminobenzenesulfonic acid using our approach versus aniline sulfonation (Figure 2C). Whilst aniline sulfonation only allows access to the *para*‐methyl isomer,[^3a^, ^3b^] our rearrangement reaction allows access to the each of the two *meta‐*methyl isomers as single compounds, depending on the starting material used. The remaining *ortho*‐methyl isomer is accessible using our method but as a mixture of isomers, a mixture that presumably could be separated by prep‐HPLC if required.

**Figure 2:**
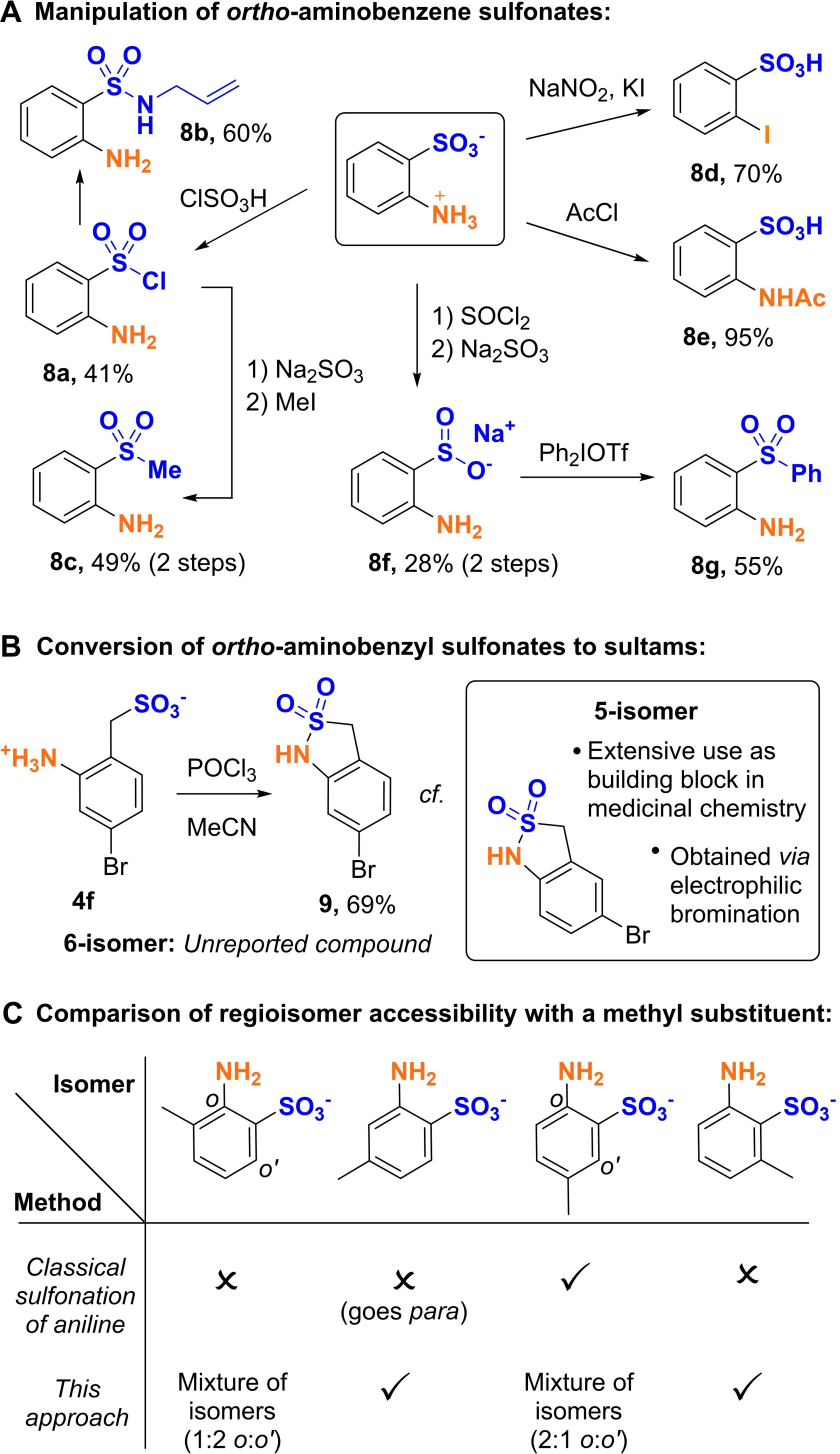
Product manipulations and comparison of isomer outcomes with aniline sulfonation

In summary, we describe the discovery of an aromatic rearrangement reaction of *O*‐benzene and *O*‐benzyl sulfonyl hydroxylamines. An iron catalyst improves yields in many cases and the reaction provides valuable *ortho*‐sulfonyl aniline products under simple reaction conditions. Our aminative approach is complementary to the main route presently used, through formation of the C−S bond during arene sulfonation. Mechanistic experiments suggest an intermolecular mechanism for the rearrangement, and we propose that the excellent regioselectivity for the *ortho* position is the result of a combination of ionic and hydrogen bonding interactions between the incoming radical cation and the anionic aryl sulfonate substrate. A limitation to overcome is that selectivity cannot currently be obtained if there are two available *ortho* positions that lead to different regioisomers. This study serves to highlight the powerful ability of attractive non‐covalent interactions to control regioselectivity in radical reactions.

## Conflict of interest

The authors declare no conflict of interest.

## Supporting information

As a service to our authors and readers, this journal provides supporting information supplied by the authors. Such materials are peer reviewed and may be re‐organized for online delivery, but are not copy‐edited or typeset. Technical support issues arising from supporting information (other than missing files) should be addressed to the authors.

Supporting Information
