# Supplementary material for: An Aminative Rearrangement of O‐(Arenesulfonyl)hydroxylamines: Facile Access to ortho‐Sulfonyl Anilines
Source: Angew Chem Int Ed Engl. 2022 Jul 7;61(33):e202204025. doi: 10.1002/anie.202204025 (PMC9546328; doi:10.1002/anie.202204025)

Angewandte Chemie
Eine Zeitschrift der Gesellschaft Deutscher Chemiker

Supporting Information

An Aminative Rearrangement of *O*-(Arenesulfonyl)hydroxylamines: Facile Access to *ortho*-Sulfonyl Anilines

*C. Morrill, J. E. Gillespie, R. J. Phipps**

Contents

1. **General information**
2. **Optimisation**
3. **Comparison of metal-free and Fe-catalysed procedures**
4. **General procedures**
5. **Synthesis of sulfonyloxycarbamate substrates**
6. **Fe-catalysed amination of sulfonyloxycarbamates**
7. **Product manipulations**
8. **Mechanistic investigations**
9. **References**
10. **NMR spectra**

1. General Information

NMR spectra: ^{1}H NMR spectra were recorded on a 700 MHz TXO Cryoprobe spectrometer, a 500 MHz Avance III Smart probe spectrometer, a 400 MHz Avance III HD Smart probe spectrometer, and a 400 MHz QNP Cryoprobe spectrometer. Chemical shifts are reported in parts per million (ppm) and the spectra are calibrated to the resonance resulting from incomplete deuteration of the solvent ($CDCl_3$: 7.26 ppm, CD_3OD: 3.31 ppm, $(CD_3)_2SO$: 2.50 ppm). ^{13}C NMR spectra were recorded with the same spectrometers with complete proton decoupling. Chemical shifts are reported in ppm with the solvent resonance as the internal standard ($^{13}CDCl_3$: 77.16 ppm, t; $^{13}CD_3OD$: 49.00, sept; $^{13}(CD_3)_2SO$: 39.52, sept). Data are reported as follows: chemical shift δ/ppm, integration (^{1}H only), multiplicity (s = singlet, d = doublet, t = triplet, q = quartet, sept = septet, br = broad, m = multiplet or combinations thereof; ^{13}C signals are singlets unless stated otherwise), coupling constants *J* in Hz. ^{1}H-COSY, DEPT-135, HSQC and HMBC were used where appropriate to facilitate structural determination of regioisomers. ^{19}F NMR spectra were recorded on a 400 MHz Avance III HD Smart probe spectrometer and were proton decoupled.

High Resolution Mass Spectrometry (HRMS): Samples were recorded on a Waters Micromass LCT Premier or a Waters Xevo G2-S or a Waters Vion QTof spectrometer using a positive electrospray ionization (ESI+). The measured values are reported to 4 decimal places and are within ±5 ppm of the calculated value. The calculated values are based on the most abundant isotope.

Chromatography: Analytical thin layer chromatography was performed using precoated Merck glass backed silica gel plates (Silicagel 60 F254). Visualisation was by ultraviolet fluorescence (λ = 254 nm) and/or staining potassium permanganate ($KMnO_4$). Flash column chromatography was performed using silica gel 60 (0.040-0.063 µm) from Material Harvest.

Reagents: Unless stated otherwise were used as supplied from commercial sources without further purification. CH_2Cl_2, THF, Et_2O, MeCN and MeOH were purified by distillation on site under an inert atmosphere via the following processes: THF and Et_2O were pre-dried over sodium wire then distilled from calcium hydride and lithium aluminium hydride; MeCN, MeOH and CH_2Cl_2 were distilled from calcium hydride.

2. Optimisation

General procedure for screening reactions: To a solution of *tert*-butyl ((phenylsulfonyl)oxy)carbamate (27 mg, 0.1 mmol, 1.0 eq.) in the given solvent was added acid (and additive). The reaction was heated to the specified temperature and stirred for 18 h. Solvent was removed under a stream of compressed air and then MeOH (0.5 mL) and NEt_3 (0.2 mL) were added. The solvent was removed under a stream of compressed air and the reaction was analysed by ^{1}H NMR.

The products formed in the crude reaction mixture during optimisation of the amination procedures were deprotonated using NEt_3 following reaction completion, in order to improve solubility and facilitate removal of iron where necessary; reactions containing iron were filtered through a plug of silica using CH_2Cl_2/MeOH (9:1), which was essential for analysis by ^{1}H NMR.

Table S1: Evaluation of acids

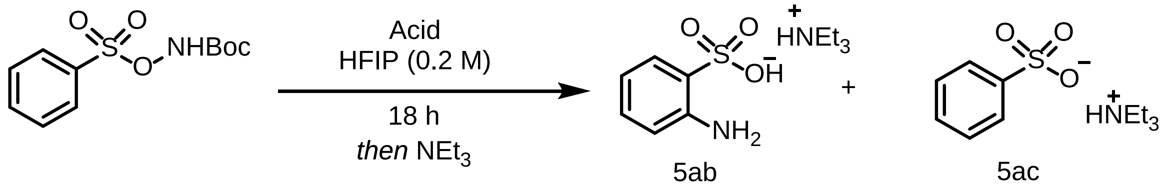

Entry	Acid (eq.)	NMR yield 5ab (%)	NMR yield 5ac (%)
**1**	HCl/MeOH (2)	0	97
**2**	HCl/MeOH (5)	0	92
**3**	HCl/dioxane (2)	0	101
**4**	HCl/dioxane (5)	0	105
**5**	*p*TSA (2)	0	97
**6**	*p*TSA (5)	0	105
**7**	TfOH (2)	11	82
**8**	TfOH (5)	0	13
**9**	TFA (1.1)	32	61
**10**	TFA (2)	35	61
**11**	TFA (5)	51	45
**12**	TFA (10)	30	70

Yields were determined by ^{1}H NMR analysis of the crude reaction mixture using 1,2-dimethoxyethane as an internal standard.

Table S2: Evaluation of solvent

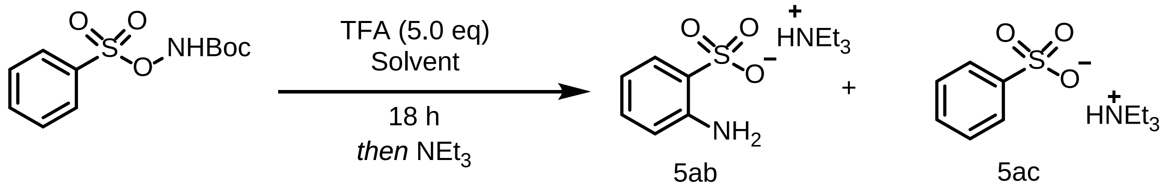

Entry	Solvent	Concentration (M)	Temperature (°C)	NMR yield 5ab (%)	NMR yield 5ac (%)
**1**	HFIP	0.2	60	51	45
**2**	HFIP/CH_2Cl_2 (1:1)	0.2	60	27	65
**3**	HFIP/H_2O (9:1)	0.2	60	48	42
**4**	EtOAc	0.2	60	15	80
**5**	MeCN	0.2	60	14	70
**6**	THF	0.2	60	14	81
**7**	MeOH	0.2	60	7	89
**8**	$CHCl_3$	0.2	60	10	81
**9**	DCE	0.2	60	26	64
**10**	CH_2Cl_2	0.2	40	53	38
**11**	CH_2Cl_2	0.5	40	23	64
**12**	CH_2Cl_2	0.1	40	62	28
**13**	CH_2Cl_2	0.05	40	70	28
**14***	CH_2Cl_2	0.05	40	80	17

Yields were determined by ^1H NMR analysis of the crude reaction mixture using 1,2-dimethoxyethane as an internal standard; *$FeSO_4.7H_2O$ (1mol%) additive

3. Comparison of metal-free and Fe-catalysed protocols

As detailed in the manuscript, the reaction was found to proceed in the absence of a Fe(II) catalyst for some substrates. In some cases adding Fe(II) made little difference to the product NMR yield but in other cases the yield was significantly improved. Adding the Fe(II) catalyst did not have a detrimental effect on reactivity for any substrates. Thus it was decided that the addition of the Fe(II) catalyst for all substrates was appropriate.

Table S3: Comparison of metal free and Fe-catalysed processes

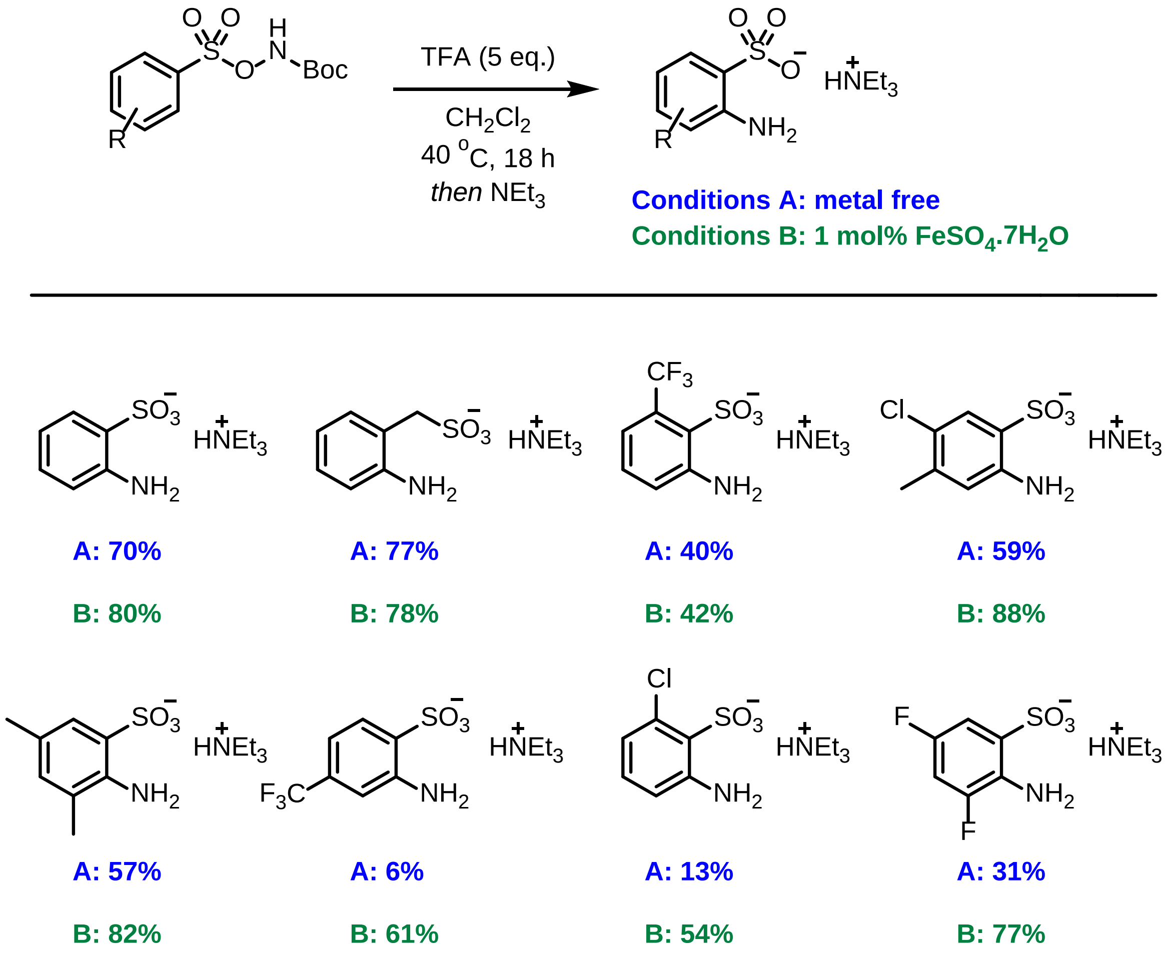

Yields are determined by ^{1}H NMR analysis of the crude reaction mixture in comparison with 1,2-dimethoxyethane as an internal standard

4. General procedures

General Procedure 1 (GP-1): Synthesis of sulfonyloxycarbamates

To a solution of *tert*-butyl *N*-hydroxycarbamate (1.0 eq.) and sulfonyl chloride (1.1 eq.) in dry Et_2O at 0 °C under N_2 was added NEt_3 (1.1 eq.). The reaction was stirred at rt for 2-16 h. Solids were removed by filtration and then the organics were washed with aq. $NaHCO_3$, dried ($MgSO_4$) and concentrated. The crude products were purified by recrystallisation or silica gel column chromatography.

General Procedure 2 (GP-2): Synthesis of tetrabutylammonium benzyl sulfonate salts

A solution of substituted benzyl bromide (1.0 eq.) and Na_2SO_3 (1.0 eq.) in acetone/H_2O (2:3) was heated to 80 °C for 2 h. The reaction was cooled to rt and the solvent was removed *in vacuo*. The residue was redissolved in H_2O and the aqueous was washed with CH_2Cl_2. Tetrabutylammoniumhydrogen sulfate (0.8 eq.) was added and the aqueous was extracted with CH_2Cl_2. The organics were washed with H_2O, dried ($MgSO_4$) and concentrated to yield the desired tetrabutylammonium salt, which was used in the following step without further purification.

General Procedure 3 (GP-3): Synthesis of sulfonyl chlorides

To a solution of tetrabutylammonium benzyl sulfonate salt in MeCN under N_2 was added $POCl_3$ (2.0 eq.). The reaction was heated to 55 °C for 2 h, then was cooled to rt and the solvent was removed under a stream of compressed air. Et_2O was added, inducing formation of a solid which was removed by filtration. The organics were concentrated to yield the sulfonyl chloride product.

General Procedure 4 (GP-4): Iron-catalysed amination of sulfonyloxycarbamates

To a solution of sulfonyloxycarbamate (1.0 eq.) and $FeSO_4{\cdot}7H_2O$ (1 mol%) in CH_2Cl_2 (0.05 M) under air was added trifluoracetic acid (5.0 eq.). The reaction vial was sealed and heated to 40 °C for 18 h, then cooled to rt and volatiles were removed under a stream of compressed air. The resulting solid was washed with a given solvent to yield the desired amination product.

Note: It was often necessary to concentrate the washings and resubject to the washing protocol to increase the isolated yield of the product.

Note: In cases where the washing solvent was not an alcohol, the addition of a few drops of MeOH into the washing solvent was required in order to remove residual $FeSO_4 \cdot 7H_2O$, which was essential for characterisation of the final products by NMR.

5. Synthesis of sulfonyloxycarbonate substrates

tert-Butyl ((phenylsulfonyl)oxy)carbamate 1a

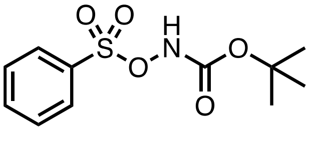

Prepared according to **GP-1** using benzenesulfonyl chloride (1.4 mL, 11 mmol, 1.1 eq.), *tert*-butyl *N*-hydroxycarbamate (1.33 g, 10 mmol, 1.0 eq.), NEt_3 (1.5 mL, 11 mmol, 1.1 eq.) and Et_2O (80 mL). The crude product was purified by recrystallisation from Pet. Ether/Et_2O to yield the title product as white crystals (1.68 g, 6.2 mmol, 62%).

^{1}H NMR (400 MHz, $CDCl_3$) δ 8.04 (d, *J* = 7.7 Hz, 2H), 7.76 – 7.67 (m, 2H), 7.60 (t, *J* = 7.7 Hz, 2H), 1.31 (s, 9H) ppm; ^{13}C NMR (101 MHz, $CDCl_3$) δ 154.0, 134.6, 133.7, 129.7, 129.0, 84.0, 27.7 ppm; HRMS calcd. for $C_{11}H_{15}NO_5SNa$ $[M+Na]^+$ 296.0.563, found 296.0566.

***O*-(Phenylsulfonyl)hydroxylammonium trifluoromethanesulfonate 1**

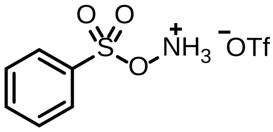

The title compound was prepared according to a reported procedure and the spectral data was consistent with reported values.[1]

tert-Butyl (*p*-tosyloxy)carbamate 1b

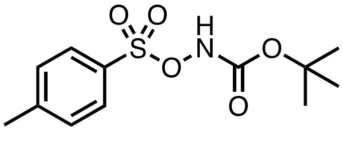

Prepared according to **GP-1** using tosyl chloride (0.95 g, 5.5 mmol, 1.1 eq.), *tert*-butyl *N*-hydroxycarbamate (0.67 g, 5.5 mmol, 1.0 eq.), NEt_3 (0.75 mL, 5.5 mmol, 1.1 eq.) and Et_2O (40 mL). The crude product was purified by recrystallisation from Pet. Ether/Et_2O to yield the title product as white crystals (833 mg, 2.9 mmol, 58%).

^{1}H NMR (400 MHz, $CDCl_3$) δ 7.91 (d, *J* = 8.1 Hz, 2H), 7.66 (br s, 1H), 7.39 (d, *J* = 8.1 Hz, 2H), 2.48 (s, 3H), 1.33 (s, 9H) ppm; ^{13}C NMR (101 MHz, $CDCl_3$) δ 154.1, 145.9, 130.6, 129.7, 129.6, 83.9, 27.7, 21.7 ppm.

Data is consistent with reported values.[2]

***tert*-Butyl ((*o*-tolylsulfonyl)oxy)carbamate 1c**

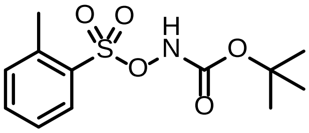

Prepared according to **GP-1** using *o*-tosyl chloride (80% purity, 1.91 g, 10.0 mmol, 1.0 eq.), *tert*-butyl *N*-hydroxycarbamate (1.33 g, 10 mmol, 1.0 eq.), NEt_3 (1.5 mL, 11 mmol, 1.1 eq.) and Et_2O (80 mL). The crude product was purified by silica gel column chromatography (Pet. Ether/EtOAc 100:0 – 88:12) to yield the title product as a white solid (1.15 g, 4.0 mmol, 50%).

^{1}H NMR (400 MHz, $CDCl_3$) δ 8.10 (s, 1H), 8.01 (dd, *J* = 8.0, 1.3 Hz, 1H), 7.56 (td, *J* = 7.5, 1.4 Hz, 1H), 7.41 – 7.32 (m, 2H), 2.74 (s, 3H), 1.30 (s, 9H). ^{13}C NMR (101 MHz, $CDCl_3$) δ 154.2, 140.3, 134.6, 132.6, 132.0, 132.0, 125.9, 83.9, 27.7, 20.9 ppm; HRMS calcd. for $C_{12}H_{16}NO_5S_2$ $[M-H]^-$ 286.0755, found 286.0764.

***tert*-Butyl ((*m*-tolylsulfonyl)oxy)carbamate 1d**

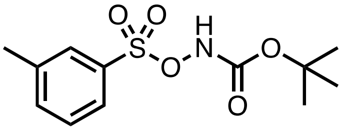

Prepared according to **GP-1** using 3-methylbenzenesulfonyl chloride (0.8 mL, 5.5 mmol, 1.1 eq.), *tert*-butyl *N*-hydroxycarbamate (0.67 g, 5.0 mmol, 1.0 eq.), NEt_3 (0.75 mL, 5.5 mmol, 1.1 eq.) and Et_2O (40 mL). The crude product was purified by recrystallisation from Pet. Ether/Et_2O to yield the title product as white crystals (1.00 g, 3.5 mmol, 70%).

^{1}H NMR (400 MHz, $CDCl_3$) δ 7.85 – 7.79 (m, 2H), 7.76 (br s, 1H), 7.55 – 7.50 (m, 1H), 7.50 – 7.44 (m, 1H), 2.47 (s, 3H), 1.32 (s, 9H) ppm; ^{13}C NMR (101 MHz, $CDCl_3$) δ 154.2, 139.4, 135.4, 133.5, 129.8, 128.9, 126.8, 83.9, 27.7, 21.2 ppm; HRMS calcd. for $C_7H_7O_3S$ $[M-NHBoc]^-$ 171.0121, found 171.0118.

tert-Butyl (((3,4-dimethylphenyl)sulfonyl)oxy)carbamate 1e

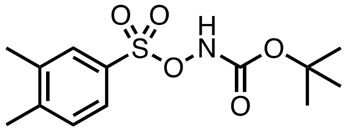

Prepared according to **GP-1** using 3,4-dimethylbenzenesulfonyl chloride (0.45 g, 2.2 mmol, 1.1 eq.), *tert*-butyl *N*-hydroxycarbamate (0.27 g, 2.0 mmol, 1.0 eq.), NEt_3 (0.25 mL, 2.2 mmol, 1.1 eq.) and Et_2O (16 mL). The crude product was purified by recrystallisation from Pet. Ether/Et_2O to yield the title product as white crystals (0.27 g, 0.90 mmol, 45%).

^{1}H NMR (400 MHz, $CDCl_3$) δ 7.78 – 7.72 (m, 2H), 7.68 (d, *J* = 2.8 Hz, 1H), 7.33 (d, *J* = 7.9 Hz, 1H), 2.38 (s, 3H), 2.37 (s, 3H), 1.32 (s, 9H) ppm; ^{13}C NMR (101 MHz, $CDCl_3$) δ 154.2, 144.6, 137.9, 130.7, 130.2, 130.2, 127.2, 83.8, 27.7, 20.1, 19.7 ppm; HRMS calcd. for $C_8H_9O_3S$ [M-NHBoc]$^-$ 185.0277, found 185.0278.

tert-Butyl (((3,5-dimethylphenyl)sulfonyl)oxy)carbamate 1f

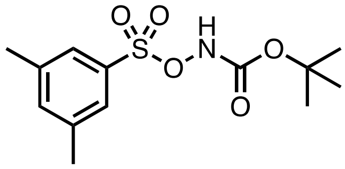

Prepared according to **GP-1** using 3,5-dimethylbenzenesulfonyl chloride (2.05 g, 11 mmol, 1.1 eq.), *tert*-butyl *N*-hydroxycarbamate (1.33 g, 10 mmol, 1.0 eq.), NEt_3 (1.5 mL, 11 mmol, 1.1 eq.) and Et_2O (80 mL). The crude product was purified by recrystallisation from Pet. Ether/Et_2O to yield the title product as white crystals (2.05 g, 0.68 mmol, 68%).

^{1}H NMR (400 MHz, $CDCl_3$) δ 7.69 (s, 1H), 7.63 (s, 2H), 7.33 (br s, 1H), 2.42 (s, 6H), 1.33 (s, 9H) ppm; ^{13}C NMR (101 MHz, $CDCl_3$) δ 154.2, 139.2, 136.3, 133.4, 127.0, 83.8, 27.7, 21.1 ppm; HRMS calcd. for $C_8H_9O_3S$ [M-NHBoc]$^-$ 185.0278, found 185.0277.

tert-Butyl (((4-butylphenyl)sulfonyl)oxy)carbamate 1g

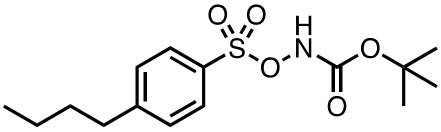

Prepared according to **GP-1** using 4-*n*-butylbenzenesulfonyl chloride (1.1 mL, 5.5 mmol, 1.1 eq.), *tert*-butyl *N*-hydroxycarbamate (0.67 g, 5.0 mmol, 1.0 eq.), NEt_3 (0.75 mL, 5.5 mmol, 1.1 eq.) and Et_2O (40 mL). The crude product was purified by recrystallisation from Pet. Ether/Et_2O to yield the title product as white crystals (905 mg, 2.8 mmol, 55%).

^{1}H NMR (500 MHz, $CDCl_3$) δ 7.92 (d, *J* = 8.3 Hz, 2H), 7.61 (br s, 1H), 7.39 (d, *J* = 8.3 Hz, 2H), 2.74 (t, *J* = 7.5 Hz, 2H), 1.69 – 1.59 (m, 2H), 1.38 (dq, *J* = 14.7, 7.5 Hz, 2H), 1.32 (s, 9H), 0.95 (t, *J* = 7.3 Hz, 3H) ppm; ^{13}C NMR (126 MHz, $CDCl_3$) δ 154.0, 150.8, 130.7, 129.8, 129.0, 83.9, 35.7, 33.2, 27.7, 22.2, 13.8 ppm; HRMS calcd. for $C_{10}H_{13}O_3S$ [M-NHBoc]$^-$ 213.0591, found 213.0594.

***tert*-Butyl (((4-(*tert*-butyl)phenyl)sulfonyl)oxy)carbamate 1h**

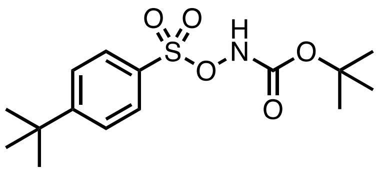

Prepared according to **GP-1** using 4-*tert*-butylbenzenesulfonyl chloride (1.28 g, 5.5 mmol, 1.1 eq.), *tert*-butyl *N*-hydroxycarbamate (0.67 g, 5.0 mmol, 1.0 eq.), NEt_3 (0.75 mL, 5.5 mmol, 1.1 eq.) and Et_2O (40 mL). The crude product was purified by recrystallisation from Pet. Ether/Et_2O to yield the title product as white crystals (0.62 g, 2.8 mmol, 56%).

^{1}H NMR (400 MHz, $CDCl_3$) δ 7.92 (d, *J* = 8.5 Hz, 2H), 7.63 (br s, 1H), 7.57 (d, *J* = 8.5 Hz, 2H), 1.35 (s, 9H), 1.27 (s, 9H) ppm; ^{13}C NMR (101 MHz, $CDCl_3$) δ 158.8, 154.0, 130.4, 129.6, 126.0, 83.8, 35.4, 31.0, 27.7 ppm; HRMS calcd. for $C_{15}H_{23}NO_5SNa$ [M+Na]$^+$ 352.1189, found 352.1186.

***tert*-Butyl (((3-fluoro-4-methylphenyl)sulfonyl)oxy)carbamate 1i**

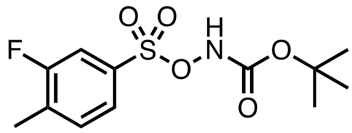

Prepared according to **GP-1** using 3-fluoro-4-methylbenzenesulfonyl chloride (1.15 g, 5.5 mmol, 1.1 eq.), *tert*-butyl *N*-hydroxycarbamate (0.67 g, 5.0 mmol, 1.0 eq.), NEt_3 (0.75 mL, 5.5 mmol, 1.1 eq.) and Et_2O (40 mL). The crude product was purified by recrystallisation from Pet. Ether/Et_2O to yield the title product as yellow crystals (1.08 g, 3.5 mmol, 71%).

^{1}H NMR (400 MHz, $CDCl_3$) δ 7.72 (dd, *J* = 8.0, 1.9 Hz, 1H), 7.67 (dd, *J* = 8.6, 1.9 Hz, 1H), 7.62 (br s, 1H), 7.42 (ddd, *J* = 8.0, 7.1, 0.9 Hz, 1H), 2.41 (d, *J* = 2.1 Hz, 3H), 1.36 (s, 9H) ppm; ^{13}C NMR

(101 MHz, $CDCl_3$) δ 161.8 (d, *J* = 250.3 Hz), 156.6, 133.2 (d, *J* = 17.2 Hz), 132.4 (d, *J* = 7.7 Hz), 132.0 (d, *J* = 5.0 Hz), 125.2 (d, *J* = 3.9 Hz), 116.5 (d, *J* = 26.1 Hz), 84.2, 27.7, 15.0 (d, *J* = 3.5 Hz) ppm; ^{19}F NMR (376 MHz, $CDCl_3$) δ -114.6 ppm; HRMS calcd. for $C_7H_6FO_3S$ [M-NHBoc]$^-$ 182.0027, found 182.0028.

tert-Butyl (((3-fluorophenyl)sulfonyl)oxy)carbamate 1j

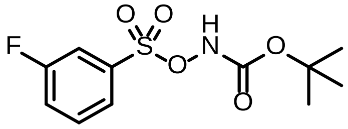

Prepared according to **GP-1** using 3-fluorobenzenesulfonyl chloride (1.03 g, 5.5 mmol, 1.1 eq.), *tert*-butyl *N*-hydroxycarbamate (0.67 g, 5.0 mmol, 1.0 eq.), NEt_3 (0.75 mL, 5.5 mmol, 1.1 eq.) and Et_2O (40 mL). The crude product was purified by recrystallisation from Pet. Ether/Et_2O to yield the title product as a white solid (796 mg, 2.7 mmol, 55%).

^1H NMR (500 MHz, $CDCl_3$) δ 8.18 (br s, 1H), 7.83 (ddd, *J* = 7.9, 1.7, 1.0 Hz, 1H), 7.71 (ddd, *J* = 7.9, 2.6, 1.7 Hz, 1H), 7.59 (td, *J* = 8.1, 5.2 Hz, 1H), 7.43 (tdd, *J* = 8.3, 2.6, 1.0 Hz, 1H), 1.32 (s, 9H) ppm; ^{13}C NMR (101 MHz, $CDCl_3$) δ 162.1 (d, *J* = 251.9 Hz), 154.3, 135.5 (d, *J* = 7.5 Hz), 132.5 – 123.9 (m), 121.9 (d, *J* = 21.2 Hz), 116.9 (d, *J* = 25.0 Hz), 84.3, 27.7 ppm; ^{19}F NMR (471 MHz, $CDCl_3$) δ -110.4 ppm; HRMS calcd. for $C_{11}H_{13}FNO_5S$ [M-H]$^-$ 290.0504, found 290.0504.

tert-Butyl (((3,4,5-trifluorophenyl)sulfonyl)oxy)carbamate 1k

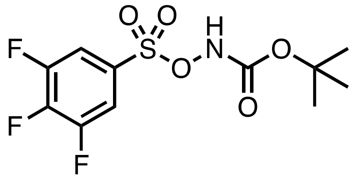

Prepared according to **GP-1** using 3,4,5-trifluorobenzenesulfonyl chloride (0.31 mL, 2.2 mmol, 1.1 eq.), *tert*-butyl *N*-hydroxycarbamate (0.27 g, 2.0 mmol, 1.0 eq.), NEt_3 (0.25 mL, 2.2 mmol, 1.1 eq.) and Et_2O (16 mL). The crude product was purified by recrystallisation from Pet. Ether/Et_2O to yield the title product as white crystals (0.40 g, 1.2 mmol, 61%).

^1H NMR (400 MHz, $CDCl_3$) δ 7.77 (br s, 1H), 7.70 (t, *J* = 6.2 Hz, 2H), 1.38 (s, 9H) ppm; ^{13}C NMR (101 MHz, $CDCl_3$) δ 153.7, 152.2 (dd, *J* = 10.7, 3.3 Hz), 149.7 (dd, *J* = 10.7, 3.3 Hz), 144.1 (d, *J* = 263.5 Hz), 129.5 (dd, *J* = 7.4, 4.9 Hz), 118.7 – 109.3 (m), 84.8, 27.7 ppm; ^{19}F NMR (376 MHz, $CDCl_3$) δ -129.4, -148.2 ppm; HRMS calcd. for $C_{11}H_{12}F_3NO_5SNa$ [M+Na]$^+$ 350.0280, found 350.0276.

tert-Butyl (((3-chloro-4-fluorophenyl)sulfonyl)oxy)carbamate 1l

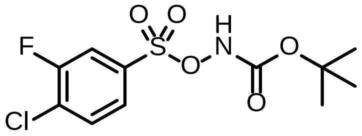

Prepared according to **GP-1** using 3-chloro-4-fluorobenzenesulfonyl chloride (1.42 mL, 10 mmol, 1.0 eq.), *tert*-butyl *N*-hydroxycarbamate (1.33 g, 10 mmol, 1.0 eq.), NEt_3 (1.53 mL, 11 mmol, 1.1 eq.) and Et_2O (80 mL). The crude product was purified by silica gel column chromatography (Pet. Ether/EtOAc 95:5 to 85:15) to yield the title product as a white solid (1.37 g, 4.2 mmol, 42%).

^{1}H NMR (400 MHz, $CDCl_3$) δ 8.12 (br s, 1H), 8.09 (dd, *J* = 6.6, 2.2 Hz, 1H), 7.99 – 7.90 (m, 1H), 7.36 (t, *J* = 8.5 Hz, 1H), 1.35 (s, 9H) ppm; ^{13}C NMR (101 MHz, $CDCl_3$) δ 161.9 (d, *J* = 260.1 Hz), 154.1, 132.5 (d, *J* = 1.5 Hz), 130.5 (d, *J* = 3.9 Hz), 130.4 (d, *J* = 9.0 Hz), 122.5 (d, *J* = 19.0 Hz), 117.4 (d, *J* = 22.6 Hz), 84.5, 27.7 ppm; ^{19}F NMR (471 MHz, $CDCl_3$) δ -104.6 ppm; HRMS calcd. for $C_{11}H_{13}ClFNO_5SNa$ $[M+Na]^+$ 348.0069, found 348.0066.

tert-Butyl (((3-chlorophenyl)sulfonyl)oxy)carbamate 1m

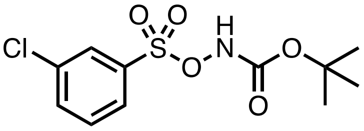

Prepared according to **GP-1** using 3-chlorobenzenesulfonyl chloride (0.77 mL, 5.5 mmol, 1.1 eq.), *tert*-butyl *N*-hydroxycarbamate (0.67 g, 5.0 mmol, 1.0 eq.), NEt_3 (0.75 mL, 5.5 mmol, 1.1 eq.) and Et_2O (40 mL). The crude product was purified by recrystallisation from Pet. Ether/Et_2O to yield the title product as yellow crystals (614 mg, 2.0 mmol, 40%).

^{1}H NMR (400 MHz, $CDCl_3$) δ 8.01 (t, *J* = 1.9 Hz, 1H), 7.93 (ddd, *J* = 7.9, 1.7, 1.0 Hz, 1H), 7.72 – 7.67 (m, 2H), 7.55 (t, *J* = 7.9 Hz, 1H), 1.35 (s, 9H) ppm; ^{13}C NMR (101 MHz, $CDCl_3$) δ 153.9, 151.8, 135.3, 134.7, 130.3, 129.5, 127.8, 84.4, 27.7 ppm; HRMS calcd. for $C_6H_4ClO_3S$ $[M-NHBoc]^-$ 190.9575, found 190.9578.

tert-Butyl (((2-chlorophenyl)sulfonyl)oxy)carbamate 1n

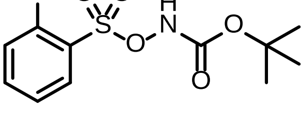

Prepared according to **GP-1** using 2-chlorobenzenesulfonyl chloride (1.03 g, 5.5 mmol, 1.1 eq.), *tert*-butyl *N*-hydroxycarbamate (0.67 g, 5.0 mmol, 1.0 eq.), NEt_3 (0.75 mL, 5.5 mmol, 1.1 eq.) and Et_2O (40 mL). The crude product was purified by recrystallisation from Pet. Ether/Et_2O to yield the title product as white crystals (698 mg, 2.3 mmol, 45%).

^{1}H NMR (400 MHz, $CDCl_3$) δ 8.16 (br s, 1H), 8.12 (dd, *J* = 8.0, 1.5 Hz, 1H), 7.66 – 7.55 (m, 2H), 7.45 (ddd, *J* = 8.0, 6.8, 1.9 Hz, 1H), 1.35 (s, 9H) ppm; ^{13}C NMR (101 MHz, $CDCl_3$) δ 154.3, 135.5, 134.2, 133.6, 132.2, 131.9, 126.9, 84.3, 27.8 ppm; HRMS calcd. for $C_{11}H_{13}ClNO_5S$ $[M-H]^-$ 306.0208, found 306.0213.

***tert*-Butyl (((4-chlorophenyl)sulfonyl)oxy)carbamate 1o**

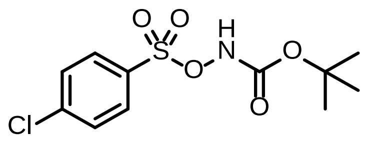

Prepared according to **GP-1** using 4-chlorobenzenesulfonyl chloride (1.03 g, 5.5 mmol, 1.1 eq.), *tert*-butyl *N*-hydroxycarbamate (0.67 g, 5.0 mmol, 1.0 eq.), NEt_3 (0.75 mL, 5.5 mmol, 1.1 eq.) and Et_2O (40 mL). The crude product was purified by recrystallisation from Pet. Ether/Et_2O to yield the title product as a white solid (727 mg, 2.4 mmol, 47%).

^{1}H NMR (400 MHz, $CDCl_3$) δ 7.96 (d, *J* = 8.3 Hz, 2H), 7.88 (br s, 1H), 7.57 (d, *J* = 8.3 Hz, 2H), 1.34 (s, 9H) ppm; ^{13}C NMR (101 MHz, $CDCl_3$) δ 154.1, 141.6, 132.0, 131.1, 129.4, 84.3, 27.7 ppm; HRMS calcd. for $C_{11}H_{13}ClNO_5S$ $[M-H]^-$ 306.0208, found 306.0202.

***tert*-Butyl (((3,5-dichlorophenyl)sulfonyl)oxy)carbamate 1p**

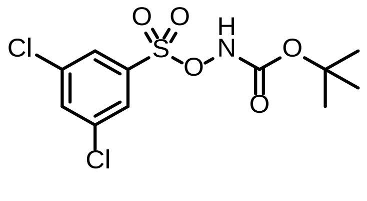

Prepared according to **GP-1** using 3,5-dichlorobenzenesulfonyl chloride (1.03 g, 5.5 mmol, 1.1 eq.), *tert*-butyl *N*-hydroxycarbamate (0.67 g, 5.0 mmol, 1.0 eq.), NEt_3 (0.75 mL, 5.5 mmol, 1.1 eq.) and Et_2O (40 mL). The crude product was purified by recrystallisation from Pet. Ether/Et_2O to yield the title product as white solid (1.02 g, 3.0 mmol, 60%).

^{1}H NMR (400 MHz, $CDCl_3$) δ 8.03 (s, 1H), 7.90 (s, 2H), 7.69 (br s, 1H), 1.37 (s, 9H) ppm; ^{13}C NMR (101 MHz, $CDCl_3$) δ 154.0, 136.4, 136.1, 134.5, 127.8, 84.7, 27.7 ppm; HRMS calcd. for $C_{11}H_{12}Cl_2NO_5S$ $[M-H]^-$ 339.9819, found 339.9820.

***tert*-Butyl (((3-chloro-4-methylphenyl)sulfonyl)oxy)carbamate 1q**

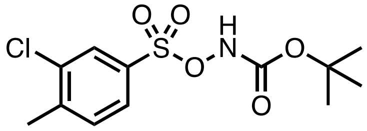

Prepared according to **GP-1** using 3-chloro-4-metylbenzenesulfonyl chloride (1.24 g, 5.5 mmol, 1.1 eq.), *tert*-butyl *N*-hydroxycarbamate (0.67 g, 5.0 mmol, 1.0 eq.), NEt_3 (0.75 mL, 5.5 mmol, 1.1 eq.) and Et_2O (40 mL). The crude product was purified by recrystallisation from Pet. Ether/Et_2O to yield the title product as white crystals (0.69 g, 2.1 mmol, 43%).

^{1}H NMR (400 MHz, $CDCl_3$) δ 7.98 (s, 1H), 7.86 – 7.78 (m, 2H), 7.46 (d, *J* = 8.0 Hz, 1H), 2.50 (s, 3H), 1.34 (s, 9H) ppm; ^{13}C NMR (101 MHz, $CDCl_3$) δ 154.1, 144.0, 135.2, 132.4, 131.4, 129.9, 127.7, 84.2, 27.7, 20.5 ppm; HRMS calcd. for $C_7H_6ClO_3S$ $[M-NHBoc]^-$ 204.9732, found 204.9735.

***tert*-Butyl (((4-bromophenyl)sulfonyl)oxy)carbamate 1r**

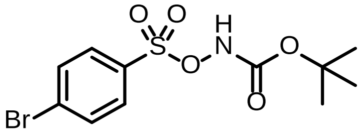

Prepared according to **GP-1** using 4-bromobenzenesulfonyl chloride (1.03 g, 5.5 mmol, 1.1 eq.), *tert*-butyl *N*-hydroxycarbamate (0.67 g, 5.0 mmol, 1.0 eq.), NEt_3 (0.75 mL, 5.5 mmol, 1.1 eq.) and Et_2O (40 mL). The crude product was purified by recrystallisation from Pet. Ether/Et_2O to yield the title product as white crystals (2.22 g, 6.3 mmol, 63%).

^{1}H NMR (500 MHz, $CDCl_3$) δ 8.15 (br s, 1H), 7.89 – 7.81 (m, 2H), 7.75 – 7.69 (m, 2H), 1.32 (s, 9H) ppm; ^{13}C NMR (126 MHz, $CDCl_3$) δ 154.3, 132.5, 132.7, 131.1, 130.2, 84.3, 27.7 ppm; HRMS calcd. for $C_6H_4BrO_3S$ $[M-NHBoc]^-$ 234.9070, found 234.9070.

tert-Butyl (((3-bromophenyl)sulfonyl)oxy)carbamate 1s

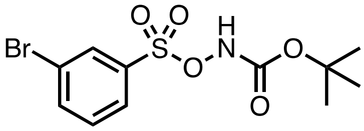

Prepared according to **GP-1** using 3-bromobenzenesulfonyl chloride (1.60 mL, 11.0 mmol, 1.1 eq.), *tert*-butyl *N*-hydroxycarbamate (1.33 g, 10.0 mmol, 1.0 eq.), NEt_3 (1.50 mL, 11.0 mmol, 1.1 eq.) and Et_2O (80 mL). The crude product was purified by silica gel column chromatography (Pet. Ether/EtOAc 95:5 – 80:20) to yield the title product as a pale yellow oil (1.60 g, 4.5 mmol, 45%).

^{1}H NMR (400 MHz, $CDCl_3$) δ 8.16 (s, 1H), 7.98 (d, *J* = 8.0 Hz, 1H), 7.85 (d, *J* = 8.0 Hz, 1H), 7.68 (br s, 1H), 7.48 (t, *J* = 8.0 Hz, 1H), 1.35 (s, 9H) ppm; ^{13}C NMR (101 MHz, $CDCl_3$) δ 154.1, 137.7, 135.4, 132.3, 130.5, 128.2, 122.9, 84.3, 27.7 ppm; HRMS calcd. for $C_6H_4BrO_3S$ $[M\text{-}NHBoc]^-$ 234.9070, found 234.9067.

tert-Butyl (((4-methoxyphenyl)sulfonyl)oxy)carbamate 1t

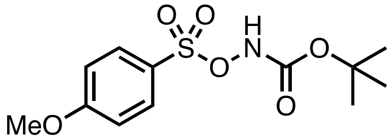

Prepared according to **GP-1** using 4-methoxybenzenesulfonyl chloride (1.03 g, 5.5 mmol, 1.1 eq.), *tert*-butyl *N*-hydroxycarbamate (0.67 g, 5.0 mmol, 1.0 eq.), NEt_3 (0.75 mL, 5.5 mmol, 1.1 eq.) and Et_2O (40 mL). The crude product was purified by silica gel column chromatography (Pet. Ether/EtOAc 95:5 to 85:15) to yield the title product as a white solid (1.43 g, 4.7 mmol, 94%).

^{1}H NMR (700 MHz, $CDCl_3$) δ 7.95 (d, *J* = 8.6 Hz, 2H), 7.68 (br s, 1H), 7.04 (d, *J* = 8.6 Hz, 2H), 3.91 (s, 3H), 1.34 (s, 9H) ppm; ^{13}C NMR (101 MHz, $CDCl_3$) δ 164.6, 154.1, 132.0, 124.7, 114.3, 83.8, 55.8, 27.8 ppm; HRMS calcd. for $C_{12}H_{17}NO_6SK$ $[M+K]^+$ 342.0408, found 342.0407.

tert-Butyl (((3-fluoro-4-methoxyphenyl)sulfonyl)oxy)carbamate 1u

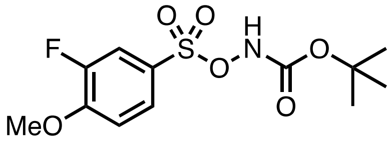

Prepared according to **GP-1** using 3-fluoro-4-methoxybenzenesulfonyl chloride (449 mg, 2.0 mmol, 1.0 eq.), *tert*-butyl *N*-hydroxycarbamate (266 mg, 2.0 mmol, 1.0 eq.), NEt_3 (0.22 mL, 2.2 mmol, 1.1 eq.) and Et_2O (16 mL). The crude product was purified by silica gel column chromatography (CH_2Cl_2) to yield the title product as a white solid (275 mg, 0.86 mmol, 43%).

^{1}H NMR (400 MHz, $CDCl_3$) δ 7.81 (br s, 1H), 7.78 (ddd, *J* = 8.7, 2.3, 1.3 Hz, 1H), 7.69 (dd, *J* = 10.1, 2.3 Hz, 1H), 7.09 (dd, *J* = 8.7, 7.8 Hz, 1H), 3.98 (s, 3H), 1.33 (s, 9H) ppm; ^{13}C NMR (101 MHz, $CDCl_3$) δ 154.1, 153.2 (d, *J* = 10.3 Hz), 151.4 (d, *J* = 252.0 Hz), 127.4 (d, *J* = 3.8 Hz), 124.9 (d, *J* = 6.5 Hz), 117.5 (d, *J* = 21.7 Hz), 112.7 (d, *J* = 2.1 Hz), 84.1, 56.6, 27.7 ppm; ^{19}F NMR (376 MHz, $CDCl_3$) δ -131.8 ppm; HRMS calcd. for $C_{12}H_{16}FNO_6SNa$ $[M+Na]^+$ 344.0575, found 345.0572.

***tert*-Butyl (((3,4-dimethoxyphenyl)sulfonyl)oxy)carbamate 1v**

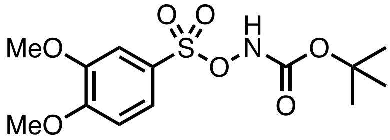

Prepared according to **GP-1** using 3,4-dimethoxybenzenesulfonyl chloride (1.30 g, 5.5 mmol, 1.1 eq.), *tert*-butyl *N*-hydroxycarbamate (0.67 g, 5.0 mmol, 1.0 eq.), NEt_3 (0.75 mL, 5.5 mmol, 1.1 eq.) and Et_2O (40 mL). The crude product was purified by silica gel column chromatography (Pet. Ether/EtOAc 85:15) and then recrystallised from Pet. Ether/Et_2O to yield the title product as white crystals (435 mg, 1.3 mmol, 26%).

^{1}H NMR (400 MHz, $CDCl_3$) δ 7.83 (s, 1H), 7.64 (dd, *J* = 8.6, 2.2 Hz, 1H), 7.43 (t, *J* = 2.2 Hz, 1H), 6.99 (dd, *J* = 8.6, 2.2 Hz, 1H), 3.97 (s, 3H), 3.95 (s, 3H), 1.33 (s, 9H) ppm; ^{13}C NMR (101 MHz, $CDCl_3$) δ 154.3, 154.2, 149.1, 124.8, 124.2, 111.5, 110.4, 83.8, 56.4 (2C), 27.8 ppm; HRMS calcd. for $C_{13}H_{19}NO_7SNa$ $[M+Na]^+$ 356.0774, found 356.0771.

***tert*-Butyl ((naphthalen-2-ylsulfonyl)oxy)carbamate 1w**

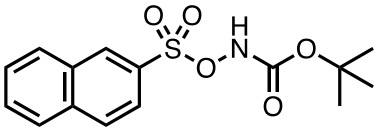

Prepared according to **GP-1** using 2-napthalenesulfonyl chloride (1.25 g, 5.5 mmol, 1.1 eq.), *tert*-butyl *N*-hydroxycarbamate (0.67 g, 5.0 mmol, 1.0 eq.), NEt_3 (0.75 mL, 5.5 mmol, 1.1 eq.) and Et_2O (40 mL). The crude product was purified by recrystallisation from Pet. Ether/Et_2O to yield the title product as white crystals (1.22 g, 3.8 mmol, 75%).

^{1}H NMR (400 MHz, $CDCl_3$) δ 8.61 (s, 1H), 8.02 (d, *J* = 8.7 Hz, 2H), 8.00 – 7.93 (m, 2H), 7.84 (br s, 1H), 7.72 (t, *J* = 7.5 Hz, 1H), 7.66 (t, *J* = 7.5 Hz, 1H), 1.19 (s, 9H) ppm; ^{13}C NMR (101 MHz, $CDCl_3$) δ 154.2, 135.7, 131.9, 131.8, 130.5, 129.8, 129.5, 129.3, 128.0, 127.8, 123.7, 84.0, 27.6 ppm; HRMS calcd. for $C_{15}H_{16}NO_5S$ $[M-H]^-$ 322.0755, found 322.0760.

tert-Butyl (((3-(trifluoromethoxy)phenyl)sulfonyl)oxy)carbamate 1x

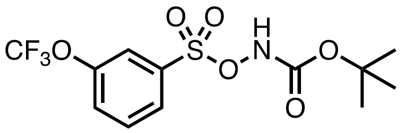

Prepared according to **GP-1** using 3-(trifluoromethoxy)benzenesulfonyl chloride (0.37 mL, 2.2 mmol, 1.1 eq.), *tert*-butyl *N*-hydroxycarbamate (0.27 g, 2.0 mmol, 1.0 eq.), NEt_3 (0.30 mL, 2.2 mmol, 1.1 eq.) and Et_2O (16 mL). The crude product was purified by recrystallisation from Pet. Ether/Et_2O to yield the title product as white crystals (498 mg, 1.4 mmol, 70%).

^{1}H NMR (400 MHz, $CDCl_3$) δ 7.97 (ddd, *J* = 7.9, 1.7, 1.1 Hz, 1H), 7.85 (s, 1H), 7.68 (br s, 1H), 7.64 (t, *J* = 7.9 Hz, 1H), 7.59 – 7.54 (m, 1H), 1.31 (s, 9H) ppm; ^{13}C NMR (101 MHz, $CDCl_3$) δ 153.8, 149.1 (d, *J* = 2.0 Hz), 135.5, 130.7, 127.9, 127.0 (d, *J* = 1.1 Hz), 122.3 (d, *J* = 1.1 Hz), 120.2 (q, *J* = 259.4 Hz), 84.4, 27.6 ppm; ^{19}F NMR (376 MHz, $CDCl_3$) δ -58.0 ppm; HRMS calcd. for $C_7H_4F_3O_4S$ $[M-NHBoc]^-$ 240.9788, found 240.9789.

tert-Butyl (((4-(trifluoromethyl)phenyl)sulfonyl)oxy)carbamate 1y

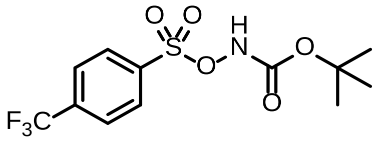

Prepared according to **GP-1** using 4-(trifluoromethyl)benzenesulfonyl chloride (1.03 g, 5.5 mmol, 1.1 eq.), *tert*-butyl *N*-hydroxycarbamate (0.67 g, 5.0 mmol, 1.0 eq.), NEt_3 (0.75 mL, 5.5 mmol, 1.1 eq.) and Et_2O (40 mL). The crude product was purified by recrystallisation from Pet. Ether/Et_2O to yield the title product as yellow crystals (1.26 g, 3.7 mmol, 74%).

^{1}H NMR (500 MHz, $CDCl_3$) δ 8.18 (dt, *J* = 8.2, 0.8 Hz, 2H), 7.96 (br s, 1H), 7.87 (dt, *J* = 8.2, 0.7 Hz, 2H), 1.30 (s, 9H) ppm; ^{13}C NMR (126 MHz, $CDCl_3$) δ 154.0, 137.2 (q, *J* = 1.5 Hz), 136.2 (q, *J* = 33.3 Hz), 130.3, 126.1 (q, *J* = 3.7 Hz), 123.0 (q, *J* = 273.2 Hz), 84.5, 27.7 ppm; ^{19}F NMR (471 MHz, $CDCl_3$) δ -64.4 ppm; HRMS calcd. for $C_{12}H_{13}F_3NO_5S$ $[M-H]^-$ 340.0472, found 340.0472.

Methyl 4-((((tert-butoxycarbonyl)amino)oxy)sulfonyl)benzoate 1z

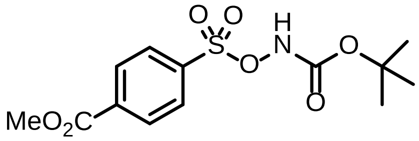

Prepared according to **GP-1** methyl-(4-chlorosulfonyl)benzoate (0.47 g, 2.0 mmol, 1 eq.), *tert*-butyl *N*-hydroxycarbamate (0.27 g, 2.0 mmol, 1.0 eq.), NEt_3 (0.30 mL, 2.2 mmol, 1.1 eq.) and Et_2O (10 mL). The crude product was purified by silica gel column chromatography (CH_2Cl_2) to yield the title product as a white solid (0.32 g, 0.97 mmol, 48%).

^1H NMR (400 MHz, $CDCl_3$) δ 8.21 (m, 3H), 8.09 (d, *J* = 8.6 Hz, 2H), 3.98 (s, 3H), 1.30 (s, 9H); ^{13}C NMR (101 MHz, $CDCl_3$) δ 165.4, 154.1, 137.6, 135.5, 130.0, 129.7, 84.2, 52.8, 27.7; HRMS calcd. for $C_{13}H_{16}NO_7S$ $[M-H]^-$ 330.0653, found 330.0662.

***tert*-Butyl (((2-(trifluoromethyl)phenyl)sulfonyl)oxy)carbamate 1za**

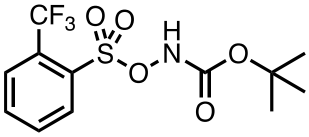

Prepared according to **GP-1** using 2-(trifluoromethyl)benzenesulfonyl chloride (0.85 mL, 5.5 mmol, 1.1 eq.), *tert*-butyl *N*-hydroxycarbamate (0.67 g, 5.0 mmol, 1.0 eq.), NEt_3 (0.75 mL, 5.5 mmol, 1.1 eq.) and Et_2O (40 mL). The crude product was purified by silica gel column chromatography (Pet.Ether/EtOAc 9:1) to yield the title product as a white solid (1.19 g, 3.5 mmol, 70%).

^1H NMR (400 MHz, $CDCl_3$) δ 8.31 (dd, *J* = 7.8, 1.5 Hz, 1H), 7.97 (dd, *J* = 7.8, 1.5 Hz, 1H), 7.83 (td, *J* = 7.5, 1.4 Hz, 1H), 7.76 (td, *J* = 7.5, 1.4 Hz, 1H), 7.69 (br s, 1H) 1.31 (s, 9H) ppm; ^{13}C NMR (101 MHz, $CDCl_3$) δ 153.9, 134.6, 134.2, 132.2, 132.0, 129.8 (q, *J* = 34.0 Hz), 128.5 (q, *J* = 6.1 Hz), 122.0 (q, *J* = 274.5 Hz), 84.4, 27.7 ppm; ^{19}F NMR (376 MHz, $CDCl_3$) δ -59.3 ppm; HRMS calcd. for $C_{12}H_{13}F_3NO_5S$ $[M-H]^-$ 340.0472, found 340.0478.

***tert*-Butyl (((3-(methylsulfonyl)phenyl)sulfonyl)oxy)carbamate 1zb**

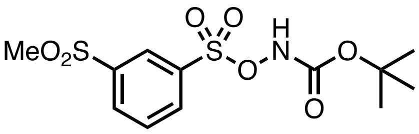

Prepared according to **GP-1** using 3-(methylsulfonyl)benzenesulfonyl chloride (0.56 g, 2.2 mmol, 1.1 eq.), *tert*-butyl *N*-hydroxycarbamate (0.27 g, 2.0 mmol, 1.0 eq.), NEt_3 (0.22 mL, 2.2 mmol, 1.1 eq.) and Et_2O (16 mL). The crude product was purified by recrystallisation from Pet. Ether/Et_2O to yield the title product as yellow crystals (216 mg, 0.61 mmol, 31%).

^{1}H NMR (400 MHz, $CDCl_3$) δ 8.61 (s, 1H), 8.31 (t, *J* = 8.4 Hz, 2H), 7.84 (t, *J* = 8.4 Hz, 1H), 7.78 (s, 1H), 3.14 (s, 3H), 1.33 (s, 9H) ppm; ^{13}C NMR (101 MHz, $CDCl_3$) δ 153.7, 142.0, 135.6, 134.4, 133.0, 130.3, 129.0, 84.7, 44.4, 27.7 ppm; HRMS calcd. for $C_{12}H_{16}NO_7S_2$ $[M-H]^-$ 350.0374, found 350.0368.

tert-Butyl (((4-nitrophenyl)sulfonyl)oxy)carbamate

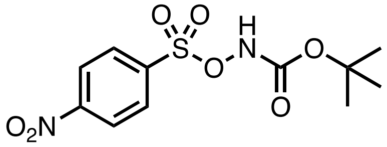

Prepared according to **GP-1** using nosyl chloride (2.44 g, 11 mmol, 1.1 eq.), *tert*-butyl *N*-hydroxycarbamate (1.33 g, 10 mmol, 1.0 eq.), NEt_3 (1.5 mL, 11 mmol, 1.1 eq.) and Et_2O (80 mL). The crude product was purified by recrystallisation from Pet. Ether/Et_2O to yield the title product as yellow crystals (2.40 g, 7.5 mmol, 75%).

^{1}H NMR (400 MHz, $CDCl_3$) δ 8.43 (d, *J* = 8.6 Hz, 2H), 8.24 (d, *J* = 8.6 Hz, 2H), 7.72 (br s, 1H), 1.34 (s, 9H) ppm; ^{13}C NMR (101 MHz, $CDCl_3$) δ 153.6, 151.2, 139.4, 131.1, 124.0, 84.8, 27.8 ppm.

Data is consistent with reported values.[3]

***O*-((4-Nitrophenyl)sulfonyl)hydroxylammonium trifluoromethanesulfonate 1zc**

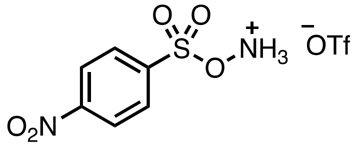

tert-Butyl (((4-nitrophenyl)sulfonyl)oxy)carbamate (2.40 g, 7.54 mmol, 1.0 eq.), was dissolved in Et_2O (40 mL) and triflic acid (0.73 mL, 8.29 mmol, 1.1 eq.) was added at 0 °C. The reaction was stirred at rt for 2 h, then the solid was collected by filtration to yield the title compound as a yellow solid (2.20 g, 6.0 mmol, 81%).

^{1}H NMR (400 MHz, DMSO-d_6) δ 9.18 (s, 1H), 8.21 (d, *J* = 8.3 Hz, 2H), 7.84 (d, *J* = 8.3 Hz, 2H) ppm; ^{13}C NMR (101 MHz, DMSO-d_6) δ 154.4, 147.8, 127.4, 123.9, 121.1 (q, *J* = 322.2 Hz); ^{19}F NMR (376 MHz, DMSO-d_6) δ -78.7 ppm.

Data is consistent with reported values.[3]

tert-Butyl ((benzylsulfonyl)oxy)carbamate 3a

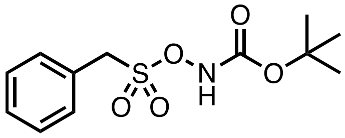

Prepared according to **GP-1** using phenylmethanesulfonyl chloride (2.09 g, 11.0 mmol, 1.1 eq.), *tert*-butyl *N*-hydroxycarbamate (1.33 g, 10.0 mmol, 1.0 eq.), NEt_3 (1.5 mL, 11.0 mmol, 1.1 eq.) and Et_2O (80 mL). The crude product was purified by recrystallisation from Pet. Ether/Et_2O to yield the title product as white crystals (927 mg, 3.22 mmol, 32%).

^{1}H NMR (400 MHz, $CDCl_3$) δ 7.82 (br s, 1H), 7.53 – 7.47 (m, 2H), 7.46 – 7.40 (m, 3H), 4.60 (s, 2H), 1.56 (s, 9H) ppm; ^{13}C NMR (101 MHz, $CDCl_3$) δ 154.8, 130.9, 129.4, 129.1, 126.5, 84.8, 55.6, 28.1 ppm; HRMS calcd. for $C_{12}H_{16}NO_5S$ $[M-H]^-$ 286.0755, found 286.0757.

tert-Butyl (((4-chlorobenzyl)sulfonyl)oxy)carbamate 3b

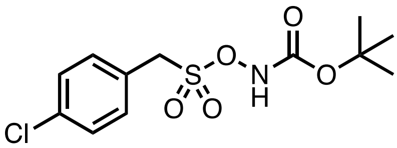

Prepared according to **GP-1** using (4-chlorophenyl)methanesulfonyl chloride (1.24 g, 5.5 mmol, 1.1 eq.), *tert*-butyl *N*-hydroxycarbamate (0.67 g, 5.0 mmol, 1.0 eq.), NEt_3 (0.75 mL, 5.5 mmol, 1.1 eq.) and Et_2O (40 mL). The crude product was purified by silica gel column chromatography (Pet. Ether/EtOAc 8:2) to yield the title product as colourless crystals (588 mg, 1.8 mmol, 37%).

^{1}H NMR (400 MHz, $CDCl_3$) δ 7.90 (s, 1H), 7.45 (d, *J* = 8.5 Hz, 2H), 7.41 (d, *J* = 8.5 Hz, 2H), 4.57 (s, 2H), 1.54 (s, 9H) ppm; ^{13}C NMR (101 MHz, $CDCl_3$) δ 154.6, 135.7, 132.2, 129.3, 125.1, 85.0, 55.0, 28.0 ppm; HRMS calcd. for $C_{12}H_{15}ClNO_5S$ $[M-H]^-$ 320.0365, found 320.0366.

(2-Chlorophenyl)methanesulfonyl chloride

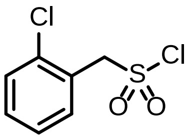

2-Chlorobenzyl bromide (1.25 g, 5.0 mmol, 1.0 eq.), Na_2SO_3 (0.76 g, 6.0 mmol, 1.2 eq.), acetone/H_2O (2:3, 25 mL) and then tetratbutylammoniumhydrogen sulfate (1.36 g, 4.0 mmol, 0.8 eq.) were subjected to **GP-2**. The resulting tetrabutylammonium sulfonate salt was subjected to **GP-3** using $POCl_3$ (0.80 mL, 8.0 mmol, 2.0 eq.) and MeCN (20 mL) to yield the title product. The product was used directly in the following step without further purification.

***tert*-Butyl (((2-chlorobenzyl)sulfonyl)oxy)carbamate 3c**

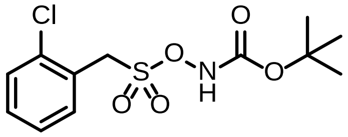

Prepared according to **GP-1** using (2-chlorophenyl)methanesulfonyl chloride (1.67 g, 7.4 mmol, 1.0 eq.), *tert*-butyl *N*-hydroxycarbamate (823 mg, 7.4 mmol, 1.0 eq.), NEt_3 (1.13 mL, 8.2 mmol, 1.1 eq.) and Et_2O. The crude product was purified by silica gel column chromatography (Pet. Ether/EtOAc 95:5 to 80:20) to yield the title product as a white solid (285 mg, 0.87 mmol, 12%).

^{1}H NMR (400 MHz, $CDCl_3$) δ 7.97 (s, 1H), 7.65 (dd, *J* = 7.1, 2.3 Hz, 1H), 7.49 (dd, *J* = 7.2, 2.1 Hz, 1H), 7.45 – 7.29 (m, 2H), 4.84 (s, 2H), 1.57 (s, 9H) ppm; ^{13}C NMR (101 MHz, $CDCl_3$) δ 154.7, 135.5, 132.8, 130.8, 130.1, 127.4, 125.2, 84.8, 52.7, 28.0 ppm; HRMS calcd. for $C_{12}H_{15}ClNO_5S$ $[M-H]^-$ 320.0365, found 320.0375

***p*-Tolylmethanesulfonyl chloride**

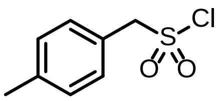

4-Methylbenzyl bromide (1.25 g, 5.0 mmol, 1.0 eq.), Na_2SO_3 (0.76 g, 6.0 mmol, 1.2 eq.), acetone/H_2O (2:3, 25 mL) and then tetratbutylammoniumhydrogen sulfate (1.36 g, 4.0 mmol, 0.8 eq.) were subjected to **GP-2**. The resulting tetrabutylammonium sulfonate salt was subjected to **GP-3** using $POCl_3$ (0.80 mL, 8.0 mmol, 2.0 eq.) and MeCN (20 mL) to yield the title product. The product was used directly in the following step without further purification.

tert-Butyl (((4-methylbenzyl)sulfonyl)oxy)carbamate 3d

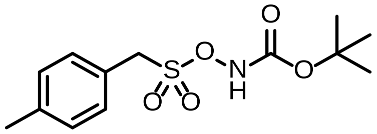

Prepared according to **GP-1** using *p*-tolylmethanesulfonyl chloride (225 mg, 1.1 mmol, 1.1 eq.), *tert*-butyl *N*-hydroxycarbamate (0.13 g, 1.0 mmol, 1.0 eq.), NEt_3 (0.15 mL, 1.1 mmol, 1.1 eq.) and Et_2O (8 mL). The crude product was purified by silica gel column chromatography (Pet. Ether/EtOAc 80:20) to yield the title product as a white solid (147 mg, 0.49 mmol, 49%).

^{1}H NMR (500 MHz, $CDCl_3$) δ 7.88 (br s, 1H), 7.38 (d, *J* = 8.0 Hz, 2H), 7.24 (d, *J* = 8.0 Hz, 2H), 4.56 (s, 2H), 2.39 (s, 3H), 1.56 (s, 9H) ppm; ^{13}C NMR (126 MHz, $CDCl_3$) δ 154.7, 139.4, 130.8, 129.8, 123.3, 84.7, 55.4, 28.1, 21.3 ppm; HRMS calcd. for $C_{13}H_{18}NO_5S$ $[M-H]^-$ 300.0911, found 300.0914.

(3,5-Dimethoxyphenyl)methanesulfonyl chloride

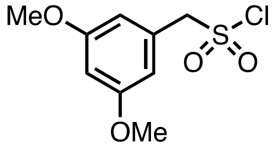

3,5-Dimethoxybenzyl bromide (1.16 g, 5.0 mmol, 1.0 eq.), Na_2SO_3 (0.76 g, 6.0 mmol, 1.0 eq.), acetone/H_2O (2:3, 25 mL) and then tetratbutylammoniumhydrogen sulfate (1.36 g, 4.0 mmol, 0.8 eq.) were subjected to **GP-2**. The resulting tetrabutylammonium sulfonate salt was subjected to **GP-3** using $POCl_3$ (0.80 mL, 8.0 mmol, 2.0 eq.) and MeCN (20 mL) to yield the title product as a brown oil. The product was used directly in the following step without further purification.

tert-Butyl (((3,5-dimethoxybenzyl)sulfonyl)oxy)carbamate 3e

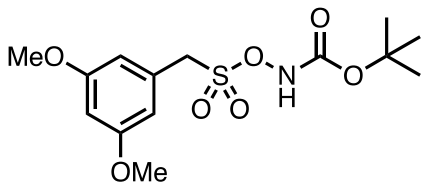

Prepared according to **GP-1** using (3,5-dimethoxyphenyl)methanesulfonyl chloride (414 mg, 1.65 mmol, 1.1 eq.), *tert*-butyl *N*-hydroxycarbamate (200 mg, 1.5 mmol, 1.0 eq.), NEt_3 (0.22 mL, 1.65 mmol, 1.1 eq.) and Et_2O (11 mL). The crude product was purified by silica gel column chromatography (Pet. Ether/EtOAc 75:25) to yield the title product as a yellow oil (220 mg, 0.63 mmol, 42%).

^1H NMR (400 MHz, $CDCl_3$) δ 7.83 (br s, 1H), 6.64 (d, *J* = 2.2 Hz, 1H), 6.51 (t, *J* = 2.2 Hz, 2H), 4.52 (s, 2H), 3.82 (s, 6H), 1.56 (s, 9H) ppm; ^{13}C NMR (101 MHz, $CDCl_3$) δ 161.1, 154.6, 128.3, 108.9, 101.5, 84.8, 55.8, 55.5, 28.0 ppm; HRMS calcd. for $C_{14}H_{20}NO_7S$ $[M-H]^-$ 346.0966, found 346.0958.

(4-Bromophenyl)methanesulfonyl chloride

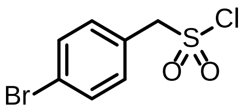

4-Bromobenzyl bromide (1.25 g, 5.0 mmol, 1.0 eq.), Na_2SO_3 (0.76 g, 6.0 mmol, 1.2 eq.), acetone/H_2O (2:3, 25 mL) and then tetratbutylammoniumhydrogen sulfate (1.36 g, 4.0 mmol, 0.8 eq.) were subjected to **GP-2**. The resulting tetrabutylammonium sulfonate salt was subjected to **GP-3** using $POCl_3$ (0.80 mL, 8.0 mmol, 2.0 eq.) and MeCN (20 mL) to yield the title product. The product was used directly in the following step without further purification.

tert-Butyl (((4-bromobenzyl)sulfonyl)oxy)carbamate 3f

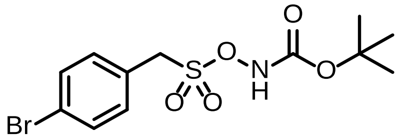

Prepared according to **GP-1** using (4-bromophenyl)methanesulfonyl chloride (1.08 g, 4.0 mmol, 1.0 eq.), *tert*-butyl *N*-hydroxycarbamate (588 mg, 4.0 mmol, 1.0 eq.), NEt_3 (0.61 mL, 4.4 mmol, 1.1 eq.) and Et_2O (30 mL). The crude product was purified by silica gel column chromatography (Pet. Ether/EtOAc 95:5 to 80:20) to yield the title product as a white solid (177 mg, 0.48 mmol, 12%).

^1H NMR (500 MHz, $CDCl_3$) δ 8.03 (br s, 1H), 7.56 (d, *J* = 8.5 Hz, 2H), 7.38 (d, *J* = 8.4 Hz, 2H), 4.54 (s, 2H), 1.56 (s, 9H) ppm; ^{13}C NMR (126 MHz, $CDCl_3$) δ 154.7, 132.5, 132.3, 125.6, 123.9, 84.9, 55.0, 28.0 ppm; HRMS calcd. for $C_{12}H_{15}BrNO_5S$ $[M-H]^-$ 363.9860, found 363.9862.

(3-Bromophenyl)methanesulfonyl chloride

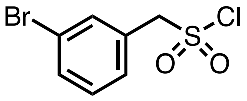

3-Bromobenzyl bromide (1.25 g, 5.0 mmol, 1.0 eq.), Na_2SO_3 (0.76 g, 6.0 mmol, 1.2 eq.), acetone/H_2O (2:3, 25 mL) and then tetratbutylammoniumhydrogen sulfate (1.36 g, 4.0 mmol, 0.8 eq.) were subjected to **GP-2**. The resulting tetrabutylammonium sulfonate salt was subjected to **GP-3** using $POCl_3$ (0.80 mL, 8.0 mmol, 2.0 eq.) and MeCN (20 mL) to yield the title product as white crystals. The product was used directly in the following step without further purification.

***tert*-Butyl (((3-bromobenzyl)sulfonyl)oxy)carbamate 3g**

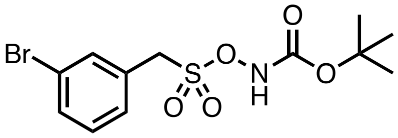

Prepared according to **GP-1** using (3-bromophenyl)methanesulfonyl chloride (296 mg, 1.1 mmol, 1.1 eq.), *tert*-butyl *N*-hydroxycarbamate (0.13 g, 1.0 mmol, 1.0 eq.), NEt_3 (0.15 mL, mmol, 1.1 eq.) and Et_2O (8 mL). The crude product was purified by silica gel column chromatography (Pet. Ether/EtOAc 80:20) to yield the title product as a yellow oil (242 mg, 0.66 mmol, 66%).

^{1}H NMR (400 MHz, $CDCl_3$) δ 7.90 (br s, 1H), 7.66 (t, *J* = 1.5 Hz, 1H), 7.57 (d, *J* = 8.0 Hz, 1H), 7.46 (dt, *J* = 8.0, 1.5 Hz, 1H), 7.31 (t, *J* = 8.0 Hz, 1H), 4.56 (s, 2H), 1.56 (s, 9H) ppm; ^{13}C NMR (101 MHz, $CDCl_3$) δ 154.6, 133.8, 132.6, 130.5, 129.5, 128.7, 122.9, 85.0, 55.0, 28.0 ppm; HRMS calcd. for $C_{12}H_{15}BrNO_5S$ $[M-H]^-$ 363.9860, found 363.9857.

(4-Fluorophenyl)methanesulfonyl chloride

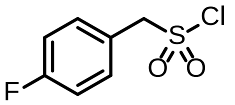

4-Fluorobenzyl bromide (2.84 g, 15.0 mmol, 1.0 eq.), Na_2SO_3 (1.89 g, 15.0 mmol, 1.2 eq.), acetone/H_2O (2:3, 50 mL) and then tetratbutylammoniumhydrogen sulfate (4.58 g, 13.5 mmol, 0.9 eq.) were subjected to **GP-2**. The resulting tetrabutylammonium sulfonate salt was

subjected to **GP-3** using $POCl_3$ (2.80 mL, 30.0 mmol, 2.0 eq.) and MeCN (60 mL) to yield the title product. The product was used directly in the following step without further purification.

tert-Butyl (((4-fluorobenzyl)sulfonyl)oxy)carbamate 3h

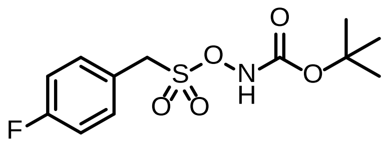

Prepared according to **GP-1** using (4-fluorophenyl)methanesulfonyl chloride (778 mg, 3.7 mmol, 1.0 eq.), *tert*-butyl *N*-hydroxycarbamate (496 mg, 3.7 mmol, 1.0 eq.), NEt_3 (0.57 mL, 4.1 mmol, 1.1 eq.) and Et_2O (15 mL). The crude product was purified by silica gel column chromatography (Pet. Ether/EtOAc 95:5 - 80:20) to yield the title product as a white solid (275 mg, 0.90 mmol, 24%).

^{1}H NMR (400 MHz, $CDCl_3$) δ 8.33 (s, 1H), 7.48 (dd, *J* = 8.4, 5.2 Hz, 2H), 7.10 (t, *J* = 8.6 Hz, 2H), 4.56 (s, 2H), 1.54 (s, 9H) ppm; ^{13}C NMR (101 MHz, $CDCl_3$) δ 163.3 (d, *J* = 249.1 Hz), 154.9, 132.8 (d, *J* = 8.6 Hz), 122.4 (d, *J* = 3.2 Hz), 116.1 (d, *J* = 21.9 Hz), 84.8, 54.7, 28.0 ppm; ^{19}F NMR (376 MHz, $CDCl_3$) δ -112.87 ppm; HRMS calcd. for $C_{12}H_{15}FNO_5S$ $[M-H]^-$ 304.0660, found 304.0660.

tert-Butyl (((4-(trifluoromethyl)benzyl)sulfonyl)oxy)carbamate 3i

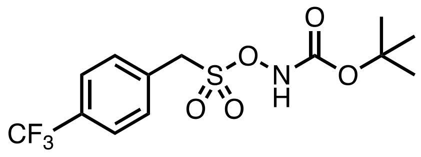

Prepared according to **GP-1** using (4-(trifluoromethyl)phenyl)methanesulfonyl chloride chloride (1.42 g, 5.5 mmol, 1.1 eq.), *tert*-butyl *N*-hydroxycarbamate (0.67 g, 5.0 mmol, 1.0 eq.), NEt_3 (0.75 mL, 5.5 mmol, 1.1 eq.) and Et_2O (40 mL). The crude product was purified by recrystallisation from Pet. Ether/Et_2O to yield the title product as white crystals (742 mg, 2.1 mmol, 42%).

^{1}H NMR (400 MHz, $CDCl_3$) δ 7.87 (s, 1H), 7.70 (d, *J* = 8.2 Hz, 2H), 7.66 (d, *J* = 8.2 Hz, 2H), 4.66 (s, 2H), 1.57 (s, 9H) ppm; ^{13}C NMR (126 MHz, $CDCl_3$) δ 154.6, 131.4, 131.3 (q, *J* = 43.8 Hz), 130.6, 126.0 (q, *J* = 3.7 Hz), 124.9, 85.1, 55.2, 28.0 ppm; ^{19}F NMR (376 MHz, $CDCl_3$) δ -62.9 ppm; HRMS calcd. for $C_{13}H_{15}F_3NO_5S$ $[M-H]^-$ 354.0629, found 354.0629.

tert-Butyl hydroxy(methyl)carbamate

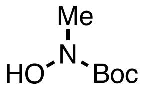

Potassium carbonate (0.73 g, 5.0 mmol, 0.5 eq.) was added to a solution of *N*-methylhydroxylamine hydrochloride (0.84 g, 10 mmol, 1.0 eq.) in THF/H_2O (1:1, 4 mL) at 0 °C. A solution of di-*tert*-butyldicarbonate (2.4 g, 10 mmol, 1.0 eq.) in THF (3 mL) was added dropwise then the reaction was stirred at rt for 5 h. The solution was concentrated and the residue was redissolved in CH_2Cl_2 (20 mL), washed with H_2O (3 x 10 mL) and brine (10 mL), dried ($MgSO_4$) and concentrated. The crude product was used directly in the following step without further purification.

tert-Butyl methyl((phenylsulfonyl)oxy)carbamate 5a

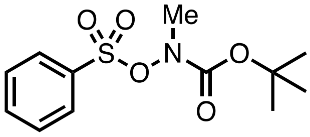

Prepared according to a modified version of **GP-1** using benzenesulfonyl chloride (1.40 mL, 11 mmol, 1.1 eq.), *tert*-butyl hydroxy(methyl)carbamate (1.33 g, 10 mmol, 1.0 eq.) and NEt_3 (1.50 mL, 11 mmol, 1.1 eq.) in Et_2O (80 mL). The crude product was purified by silica gel column chromatography (Pet. Ether/EtOAc 95:5 to 9:1) to yield the title product as white crystals (1.42 g, 4.98 mmol, 99%).

^1H NMR (400 MHz, $CDCl_3$) δ 8.02 (d, *J* = 8.1 Hz, 2H), 7.72 (t, *J* = 7.5 Hz, 1H), 7.59 (t, *J* = 7.4 Hz, 2H), 3.29 (s, 3H), 1.23 (s, 9H) ppm; ^{13}C NMR (101 MHz, $CDCl_3$) δ 156.1, 134.4, 134.2, 129.7, 129.0, 83.4, 40.3, 27.6 ppm; HRMS calcd. for $C_{12}H_{17}NO_5SNa$ $[M+Na]^+$ 310.0720, found 310.0723.

tert-Butyl methyl(tosyloxy)carbamate

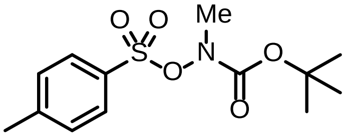

Prepared according to a modified version of **GP-1** using 4-toluenesulfonyl chloride (381 mg, 2.0 mmol, 1.0 eq.), *tert*-butyl hydroxy(methyl)carbamate (294 mg, 2.0 mmol, 1.0 eq.) and NEt_3 (0.30 mL, 2.2 mmol, 1.1 eq.) in Et_2O (16 mL). The crude product was purified by silica gel

column chromatography (Pet. Ether/EtOAc 100:0 to 95:5) to yield the title product as a white solid (378 mg, 1.3 mmol, 63%).

^{1}H NMR (400 MHz, $CDCl_3$) δ 7.85 (d, *J* = 8.0 Hz, 2H), 7.35 (d, *J* = 8.0 Hz, 2H), 3.23 (s, 3H), 2.45 (s, 3H), 1.21 (s, 9H) ppm; ^{13}C NMR (101 MHz, $CDCl_3$) δ 156.0, 145.7, 131.1, 129.7, 129.5, 83.3, 40.1, 27.5, 21.7 ppm; HRMS calcd. for $C_{13}H_{19}NO_5SNa$ $[M+Na]^+$ 324.0876, found 324.0882.

6. Amination of sulfonyloxycarbamates

2-Aminobenzenesulfonic acid 2a

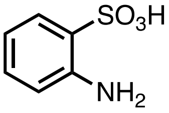

Prepared according to **GP-4** using *tert*-butyl ((phenylsulfonyl)oxy)carbamate **1a** (68 mg, 0.25 mmol, 1.0 eq.), $FeSO_4{\cdot}7H_2O$ (0.70 mg, 2.5 µmol, 0.01 eq.), TFA (0.1 mL, 1.25 mmol, 5.0 eq.) and CH_2Cl_2 (5 mL). The crude residue was washed with acetone to yield the title product as a pink solid (32.1 mg, 0.185 mmol, 74%) as a >20:1 mixture of regioisomers.

^{1}H NMR (500 MHz, DMSO-*d*$_6$) δ 8.66 (s, 3H), 7.78 (d, *J* = 7.5 Hz, 1H), 7.48 (t, *J* = 7.6 Hz, 1H), 7.41 (t, *J* = 7.4 Hz, 1H), 7.33 (d, *J* = 7.7 Hz, 1H) ppm; ^{13}C NMR (126 MHz, DMSO-*d*$_6$) δ 140.5, 131.0, 128.9, 128.1, 128.0, 124.2 ppm.

Data is consistent with reported values.[4]

Alternative procedure:

A solution of *O*-(phenylsulfonyl)hydroxylammonium trifluoromethanesulfonate **1** (32 mg, 0.1 mmol, 1.0 eq.) in HFIP (0.2 mL) was heated to 60 °C for 18 h. The solvent was removed under a stream of compressed air and the crude residue was washed with acetone to yield the title product as a pink solid (10.3 mg, 0.06 mmol, 60%) as a >20:1 mixture of regioisomers.

2-Amino-4-methylbenzenesulfonic acid 2b

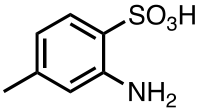

Prepared according to **GP-4** using *tert*-butyl (*p*-tosyloxy)carbamate **1b** (72 mg, 0.25 mmol, 1.0 eq.), $FeSO_4{\cdot}7H_2O$ (0.70 mg, 2.5 µmol, 0.01 eq.), TFA (0.1 mL, 1.25 mmol, 5.0 eq.) and CH_2Cl_2 (5 mL). The crude residue was washed with MeOH and acetone to yield the title product as a pink solid (30.0 mg, 0.160 mmol, 64%) as a 17:1 mixture of *o/m* regioisomers.

^{1}H NMR (400 MHz, DMSO-*d*$_6$) δ 8.17 (br s, 3 H), 7.65 (d, *J* = 7.9 Hz, 1H), 7.21 (d, *J* = 7.9 Hz, 1H), 7.10 (s, 1H), 2.33 (s, 3H) ppm; ^{13}C NMR (101 MHz, DMSO-*d*$_6$) δ 140.9, 137.9, 128.7, 128.6, 128.0, 124.3, 21.1 ppm; HRMS calcd. for $C_7H_8NO_3S$ [M-H]$^-$ 186.0225, found 186.0226.

2-Amino-6-methylbenzenesulfonic acid 2c

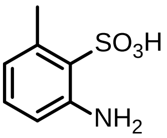

Prepared according to **GP-4** using *tert*-Butyl ((*o*-tolylsulfonyl)oxy)carbamate **1c** (72 mg, 0.25 mmol, 1.0 eq.), $FeSO_4{\cdot}7H_2O$ (0.70 mg, 2.5 µmol, 0.01 eq.), TFA (0.1 mL, 1.25 mmol, 5.0 eq.) and CH_2Cl_2 (5 mL). The crude residue was washed with acetone to yield the title product as a pale brown solid (22.1 mg, 0.121 mmol, 47%) as a >20:1 mixture of regioisomers.

^{1}H NMR (500 MHz, DMSO-*d*$_6$) δ 7.36 (t, *J* = 7.6 Hz, 1H), 7.28 (d, *J* = 7.5 Hz, 1H), 7.14 (d, *J* = 7.8 Hz, 1H), 2.63 (s, 3H) ppm; ^{13}C NMR (126 MHz, DMSO-*d*$_6$) δ 139.3, 138.6, 131.8, 129.9, 128.4, 122.5, 21.7 ppm; HRMS calcd. for $C_7H_{10}NO_3S$ [M+H]$^+$ 188.0376, found 188.0373.

2-Amino-5-methylbenzenesulfonic acid and 2-amino-3-methylbenzenesulfonic acid 2d

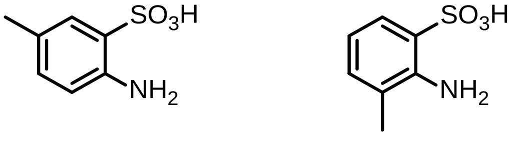

major

minor

Prepared according to **GP-4** using *tert*-butyl ((*m*-tolylsulfonyl)oxy)carbamate **1d** (72 mg, 0.25 mmol, 1.0 eq.), $FeSO_4{\cdot}7H_2O$ (0.70 mg, 2.5 µmol, 0.01 eq.), TFA (0.1 mL, 1.25 mmol, 5.0 eq.)

and CH_2Cl_2 (5 mL). The crude residue was washed with acetone to yield the title products as a white solid (32.0 mg, 0.171 mmol, 68%) as a 1.8:1 mixture of regioisomers.

Major isomer: ^{1}H NMR (400 MHz, DMSO-d_6) δ 8.90 (br s, 3H), 7.59 (s, 1H), 7.28 (m, 1H), 7.21 (d, J = 8.2 Hz, 1H), 2.34 (s, 3H) ppm; ^{13}C NMR (101 MHz, DMSO-d_6) δ 140.4, 137.1, 132.5, 131.3, 128.4, 124.1, 20.9 ppm; *Minor isomer:* ^{1}H NMR (400 MHz, DMSO-d_6) δ 8.90 (br s, 3H), 7.59 (s, 1H), 7.30 (m, 1H), 7.17 (d, J = 7.7 Hz, 1H), 2.34 (s, 3H) ppm; ^{13}C NMR (101 MHz, DMSO-d_6) δ 139.1, 132.4, 131.5, 129.6, 125.9, 125.7, 17.5 ppm; HRMS calcd. for $C_7H_8NO_3S$ [M-H]$^-$ 186.0225, found 186.0228.

2-Amino-4,5-dimethylbenzenesulfonic acid and 2-amino-3,4-dimethylbenzenesulfonic acid 2e

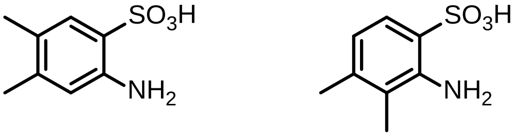

major minor

Prepared according to **GP-4** using *tert*-butyl (((3,4-dimethylphenyl)sulfonyl)oxy)carbamate **1e** (75 mg, 0.25 mmol, 1.0 eq.), $FeSO_4 \cdot 7H_2O$ (0.70 mg, 2.5 µmol, 0.01 eq.), TFA (0.1 mL, 1.25 mmol, 5.0 eq.) and CH_2Cl_2 (5 mL). The crude residue was washed with acetone to yield the title products as a white solid (30.1 mg, 0.150 mmol, 60%) as a 2.7:1 mixture of regioisomers (crude ratio 1.9:1).

Major isomer: ^{1}H NMR (500 MHz, DMSO-d_6) δ 7.53 (s, 1H), 7.07 (s, 1H), 2.23 (s, 6H) ppm; ^{13}C NMR (126 MHz, DMSO-d_6) δ 139.7, 137.8, 136.8, 128.8, 125.7, 124.8, 19.6, 19.3 ppm; *Minor isomer:* ^{1}H NMR (500 MHz, DMSO-d_6) δ 7.53 (d, J = 7.9 Hz, 1H), 7.19 (d, J = 7.9 Hz, 1H), 2.28 (s, 3H), 2.17 (s, 3H) ppm; ^{13}C NMR (126 MHz, DMSO-d_6) δ 140.2, 137.6, 131.0, 128.6, 127.6, 123.4, 20.4, 13.9 ppm; HRMS calcd. for $C_8H_{10}NO_3S^-$ [M-H]$^-$ 200.0381, found 200.0384.

2-Amino-3,5-dimethylbenzenesulfonic acid 2f

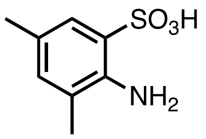

Prepared according to **GP-4** using *tert*-butyl (((3,5-dimethyl)phenyl)sulfonyl)oxy)carbamate **1f** (75 mg, 0.25 mmol, 1.0 eq.), $FeSO_4{\cdot}7H_2O$ (0.70 mg, 2.5 µmol, 0.01 eq.), TFA (0.1 mL, 1.25 mmol, 5.0 eq.) and CH_2Cl_2 (5 mL). The crude residue was washed with acetone to yield the title product as a white solid (36.3 mg, 0.180 mmol, 72%) as a >20:1 mixture of regioisomers.

^{1}H NMR (400 MHz, DMSO-d_6) δ 7.44 (s, 1H), 7.17 (s, 1H), 2.29 (s, 3H), 2.28 (s, 3H) ppm; ^{13}C NMR (101 MHz, DMSO-d_6) δ 140.2, 137.2, 133.2, 132.6, 126.0, 124.7, 20.8, 17.3 ppm; HRMS calcd. for $C_8H_{10}NO_3S$ $[M-H]^-$ 200.0381, found 200.0385.

2-Amino-4-butylbenzenesulfonic acid 2g

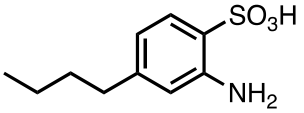

Prepared according to a modified version of **GP-4** using *tert*-butyl (((4-butylphenyl)sulfonyl)oxy)carbamate **1g** (82 mg, 0.25 mmol, 1.0 eq.), TFA (0.1 mL, 1.25 mmol, 5.0 eq.) and CH_2Cl_2 (5 mL). The crude residue was washed with EtOAc to yield the title product as a white solid (39.1 mg, 0.171 mmol, 68%) as a 17:1 mixture of *o/m* regioisomers.

^{1}H NMR (500 MHz, DMSO-d_6) δ 7.85 (br s, 3H), 7.68 (d, J = 7.9 Hz, 1H), 7.25 (dd, J = 8.0, 1.7 Hz, 1H), 7.14 (d, J = 1.6 Hz, 1H), 2.61 (t, J = 7.6 Hz, 2H), 1.63 – 1.49 (m, 2H), 1.29 (h, J = 7.3 Hz, 2H), 0.89 (t, J = 7.4 Hz, 3H) ppm; ^{13}C NMR (126 MHz, DMSO-d$_6$) δ 145.7, 138.3, 128.3, 128.3, 128.1, 123.9, 34.6, 33.1, 22.0, 14.2 ppm; HRMS calcd. for $C_{10}H_{16}NO_3S$ $[M+H]^+$ 230.0845, found 230.0853.

2-amino-4-(*tert*-butyl)benzenesulfonic acid 2h

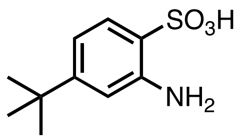

Prepared according to **GP-4** using *tert*-butyl (((4-(*tert*-butyl)phenyl)sulfonyl)oxy)carbamate **1h** (82 mg, 0.25 mmol, 1.0 eq.), $FeSO_4·7H_2O$ (0.70 mg, 2.5 µmol, 0.01 eq.), TFA (0.1 mL, 1.25 mmol, 5.0 eq.) and CH_2Cl_2 (5 mL). The crude residue was washed with Et_2O to yield the title product as a pink solid (38.0 mg, 0.166 mmol, 66%) as a >20:1 mixture of regioisomers.

^{1}H NMR (400 MHz, DMSO-d_6) δ 7.69 (dd, J = 8.1, 1.8 Hz, 1H), 7.43 (d, J = 8.1 Hz, 1H), 7.32 (d, J = 1.8 Hz, 1H), 1.28 (s, 9H) ppm; ^{13}C NMR (101 MHz, DMSO-d_6) δ 153.9, 137.8, 128.7, 128.0, 124.8, 120.8, 35.0, 31.2 ppm; HRMS calcd. for $C_{10}H_{14}NO_3S$ [M-H]$^-$ 228.0694, found 228.0696.

2-Amino-5-fluoro-4-methylbenzenesulfonic acid and 2-amino-3-fluoro-4-methylbenzenesulfonic acid 2i

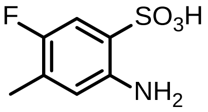

major

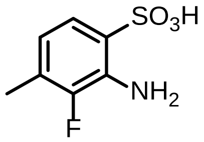

minor

Prepared according to **GP-4** using *tert*-butyl (((3-fluoro-4-methylphenyl)sulfonyl)oxy)carbamate **1i** (76 mg, 0.25 mmol, 1.0 eq.), $FeSO_4·7H_2O$ (0.70 mg, 2.5 µmol, 0.01 eq.), TFA (0.1 mL, 1.25 mmol, 5.0 eq.) and CH_2Cl_2 (5 mL). The crude residue was washed with acetone to yield the title products as a yellow solid (40.3 mg, 0.199 mmol, 79%) as a 4.2:1 mixture of *o/o'* regioisomers (crude ratio 4.2:1).

Major Isomer: ^{1}H NMR (700 MHz, DMSO-d_6) δ 8.40 (br s, 3H), 7.42 (d, J = 9.0 Hz, 1H), 7.24 (d, J = 6.4 Hz, 1H), 2.26 (d, J = 1.7 Hz, 3H) ppm; ^{13}C NMR (126 MHz, DMSO-d_6) δ 159.2 (d, J = 246.1 Hz), 140.4, 127.6 (d, J = 18.9 Hz), 127.2, 124.4, 114.3 (d, J = 25.3 Hz), 14.4 ppm; ^{19}F NMR (471 MHz, DMSO-d_6) δ -121.5 ppm. *Minor Isomer*: ^{1}H NMR (700 MHz, DMSO-d_6) δ 8.40 (br s, 3H), 7.24 (m, 1H), 6.66 (t, J = 7.2 Hz, 1H), 2.18 (d, J = 1.9 Hz, 3H) ppm; ^{13}C NMR (126 MHz, DMSO-d_6) δ 151.2 (d, J = 240.8 Hz), 134.2, 128.1 (d, J = 14.6 Hz), 125.3 (d, J = 15.0 Hz), 122.6, 120.8, 14.6 ppm; ^{19}F NMR (471 MHz, DMSO-d_6) δ -139.0 ppm; HRMS calcd. for $C_7H_9FNO_3S$ [M+H]$^+$ 206.0285, found 206.0282.

2-Amino-5-fluorobenzenesulfonic acid and 2-amino-3-fluorobenzenesulfonic acid 2j

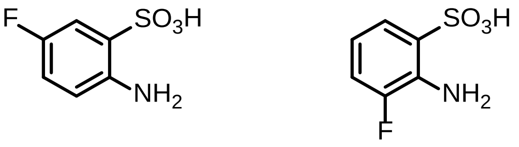

major minor

Prepared according to **GP-4** using *tert*-butyl (((3-fluorophenyl)sulfonyl)oxy)carbamate **1j** (73 mg, 0.25 mmol, 1.0 eq.), $FeSO_4 \cdot 7H_2O$ (0.70 mg, 2.5 μmol, 0.01 eq.), TFA (0.1 mL, 1.25 mmol, 5.0 eq.) and CH_2Cl_2 (5 mL). The crude residue was washed with acetone to yield the title products as a white solid (30.1 mg, 0.158 mmol, 63%) as a 6.3:1 mixture of regioisomers (crude ratio 5.1:1).

Major isomer: ^1H NMR (400 MHz, DMSO-d_6) δ 8.47 (br s, 3H), 7.50 (dd, *J* = 8.4, 2.6 Hz, 1H), 7.43 – 7.30 (m, 2H) ppm; ^{13}C NMR (101 MHz, DMSO-d_6) δ 160.6 (d, *J* = 247.0 Hz), 142.6, 126.4 (d, *J* = 8.3 Hz), 125.6, 117.8 (d, *J* = 23.1 Hz), 114.7 (d, *J* = 24.4 Hz) ppm; ^{19}F NMR (471 MHz, DMSO-d_6) δ -114.4 ppm; HRMS calcd. for $C_6H_7FNO_3S$ $[M+H]^+$ 192.0125, found 192.0122.

2-Amino-3,4,5-trifluorobenzenesulfonic acid 2k

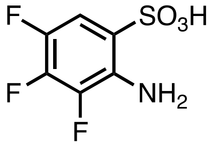

Prepared according to **GP-4** using *tert*-butyl (((3,4,5-trifluorophenyl)sulfonyl)oxy)carbamate **1 k** (82 mg, 0.25 mmol, 1.0 eq.), $FeSO_4 \cdot 7H_2O$ (0.70 mg, 2.5 μmol, 0.01 eq.), TFA (0.1 mL, 1.25 mmol, 5.0 eq.) and CH_2Cl_2 (5 mL). The crude residue was washed with EtOAc to yield the title product as a white solid (21.6 mg, 0.095 mmol, 38%).

^1H NMR (400 MHz, DMSO-d_6) δ 7.21 (ddd, *J* = 10.7, 8.4, 2.3 Hz, 1H), 5.98 (br s, 3H) ppm; ^{13}C NMR (101 MHz, DMSO-d_6) δ 141.3 – 140.4 (m), 139.4 – 138.9 (m), 138.6 (dd, *J* = 11.9, 2.6 Hz), 131.5 (dt, *J* = 10.7, 1.9 Hz), 127.9 – 126.2 (m), 110.0 (dd, *J* = 18.6, 3.2 Hz) ppm; ^{19}F NMR (471 MHz, DMSO-d_6) δ -154.7 (dd, *J* = 22.9, 3.7 Hz), -156.2 (dd, *J* = 20.3, 3.7 Hz), -162.1 (dd, *J* = 22.9, 20.3 Hz) ppm; HRMS calcd. for $C_6H_8F_3N_2O_3S$ $[M+NH_4]^+$ 245.0202, found 245.0192.

2-Amino-5-chloro-4-fluorobenzenesulfonic acid 2l

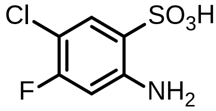

Prepared according to **GP-4** using *tert*-butyl (((3-chloro-4-fluorophenyl)sulfonyl)oxy)carbamate **1l** (81 mg, 0.25 mmol, 1.0 eq.), $FeSO_4{\cdot}7H_2O$ (0.70 mg, 2.5 µmol, 0.01 eq.), TFA (0.1 mL, 1.25 mmol, 5.0 eq.) and CH_2Cl_2 (5 mL). The crude residue was washed with acetone to yield the title product as a pink solid (27.2 mg, 0.121 mmol, 48%) as a >20:1 mixture of regioisomers (crude ratio 2.5:1).

^{1}H NMR (500 MHz, DMSO-d_6) δ 7.52 (d, J = 8.3 Hz, 1H), 6.72 (d, J = 11.4 Hz, 1H), 6.55 (br s, 3H) ppm; ^{13}C NMR (126 MHz, DMSO-d_6) δ 158.0 (d, J = 244.7 Hz), 143.3 (d, J = 10.8 Hz), 130.1 (d, J = 2.4 Hz), 129.3, 106.4 (d, J = 18.1 Hz), 104.7 (d, J = 24.1 Hz) ppm; ^{19}F NMR (471 MHz, DMSO-d_6) δ -116.9 ppm; HRMS calcd. for $C_6H_6ClFNO_3S$ $[M+H]^+$ 225.9741, found 225.9740.

Note: the crude ^{1}H NMR indicated a 2.5:1 mixture of regioisomers.

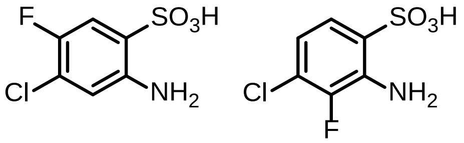

r.r. 2.5:1

2-Amino-5-chlorobenzenesulfonic acid 2m

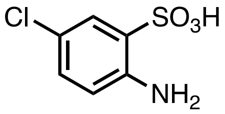

Prepared according to **GP-4** using *tert*-butyl (((3-chlorophenyl)sulfonyl)oxy)carbamate **1m** (77 mg, 0.25 mmol, 1.0 eq.), $FeSO_4{\cdot}7H_2O$ (0.70 mg, 2.5 µmol, 0.01 eq.), TFA (0.1 mL, 1.25 mmol, 5.0 eq.) and CH_2Cl_2 (5 mL). The crude residue was washed with acetone to yield the title product as a white solid (15.4 mg, 0.0742 mmol, 30%) as a >20:1 mixture of *o/o'* regioisomers (crude ratio 7.0:1).

^{1}H NMR (400 MHz, DMSO-d_6) δ 7.58 (d, J = 2.7 Hz, 1H), 7.38 (dd, J = 8.6, 2.7 Hz, 1H), 7.11 (d, J = 8.6 Hz, 1H) ppm; ^{13}C NMR (101 MHz, DMSO-d_6) δ 138.9, 133.2, 130.4, 127.4, 123.5, 123.3 ppm.

Data is consistent with reported values.[5]

Note: the crude ^{1}H NMR indicated a 7.0:1 mixture of regioisomers.

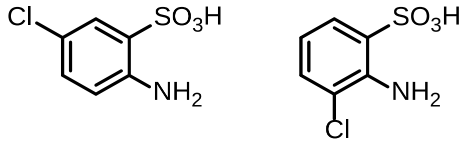

r.r. 7.0:1

2-Amino-6-chlorobenzenesulfonic acid 2n

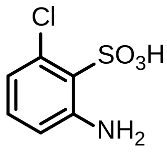

Prepared according to **GP-4** using *tert*-butyl (((2-chlorophenyl)sulfonyl)oxy)carbamate **1n** (77 mg, 0.25 mmol, 1.0 eq.), $FeSO_4 \cdot 7H_2O$ (0.70 mg, 2.5 µmol, 0.01 eq.), TFA (0.1 mL, 1.25 mmol, 5.0 eq.) and CH_2Cl_2 (5 mL). The crude residue was washed with methanol to yield the title product as a white solid (19.8 mg, 0.095 mmol, 38%) as a >20:1 mixture of regioisomers.

^{1}H NMR (500 MHz, DMSO-d_6) δ 7.26 (t, *J* = 7.9 Hz, 1H), 7.17 (dd, *J* = 7.9, 1.3 Hz, 1H), 7.04 (dd, *J* = 8.0, 1.3 Hz, 1H) ppm; ^{13}C NMR (126 MHz, DMSO-d_6) δ 136.0, 133.7, 133.4, 130.8, 129.2, 122.6 ppm; HRMS calcd. for $C_6H_7ClNO_3S$ $[M+H]^+$ 207.9835, found 207.9835.

2-Amino-4-chlorobenzenesulfonic acid 2o

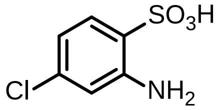

Prepared according to **GP-4** using *tert*-butyl (((4-chlorophenyl)sulfonyl)oxy)carbamate **1o** (77 mg, 0.25 mmol, 1.0 eq.), $FeSO_4 \cdot 7H_2O$ (0.70 mg, 2.5 µmol, 0.01 eq.), TFA (0.1 mL, 1.25 mmol, 5.0 eq.) and CH_2Cl_2 (5 mL). The crude residue was washed with acetone to yield the title product as a pink solid (24.3 mg, 0.117 mmol, 47%) as a >20:1 mixture of regioisomers.

^{1}H NMR (500 MHz, DMSO-d_6) δ 7.61 (d, *J* = 8.0 Hz, 1H), 7.49 (br s, 3H), 7.07 (s, 1H), 7.05 (d, *J* = 8.2 Hz, 1H) ppm; ^{13}C NMR (126 MHz, DMSO-d_6) δ 137.6, 135.2, 134.4, 129.7, 122.2, 119.9 ppm; HRMS calcd. for $C_6H_7ClNO_3S$ $[M+H]^+$ 207.9835, found 207.9835.

2-Amino-3,5-dichlorobenzenesulfonic acid 2p

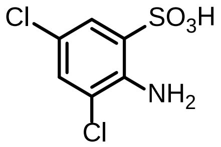

Prepared according to **GP-4** using *tert*-butyl (((3,5-dichlorophenyl)sulfonyl)oxy)carbamate **1p** (86 mg, 0.25 mmol, 1.0 eq.), $FeSO_4\cdot7H_2O$ (0.70 mg, 2.5 µmol, 0.01 eq.), TFA (0.1 mL, 1.25 mmol, 5.0 eq.) and CH_2Cl_2 (5 mL). The crude residue was washed with acetone to yield the title product as a brown solid (33.6 mg, 0.139 mmol, 56%) as a >20:1 mixture of regioisomers.

^{1}H NMR (400 MHz, DMSO-d_6) δ 7.40 (d, J = 2.5 Hz, 1H), 7.34 (d, J = 2.5 Hz, 1H), 6.60 (br s, 3H) ppm; ^{13}C NMR (101 MHz, DMSO-d_6) δ 140.6, 133.4, 129.1, 126.2, 119.3, 118.2 ppm; HRMS calcd. for $C_6H_6Cl_2NO_3S$ $[M+H]^+$ 241.9440, found 241.9431.

2-Amino-5-chloro-4-methylbenzenesulfonic acid and 2-amino-3-chloro-4-methylbenzenesulfonic acid 2q

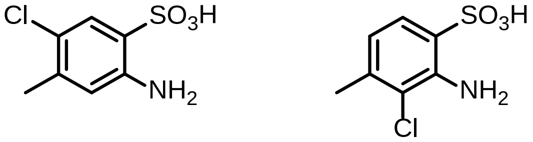

major minor

Prepared according to **GP-4** using *tert*-butyl (((3-chloro-4-methylphenyl)sulfonyl)oxy)carbamate **1q** (80 mg, 0.25 mmol, 1.0 eq.), $FeSO_4\cdot7H_2O$ (0.70 mg, 2.5 µmol, 0.01 eq.), TFA (0.1 mL, 1.25 mmol, 5.0 eq.) and CH_2Cl_2 (5 mL). The crude residue was washed with acetone and then methanol to yield the title products as a white solid (37.6 mg, 0.170 mmol, 68%) as a 5.1:1 mixture of *o/o'* regioisomers (crude ratio 4.8:1).

Major isomer: ^{1}H NMR (500 MHz, DMSO-d_6) δ 7.62 (s, 1H), 7.14 (s, 1H), 2.32 (s, 3H) ppm; ^{13}C NMR (126 MHz, DMSO-d_6) δ 138.2, 137.1, 130.9, 128.0, 127.9, 125.0, 19.9 ppm; *Minor isomer:* ^{1}H NMR (500 MHz, DMSO-d_6) δ 7.35 (dd, J = 7.8, 1.7 Hz, 1H), 6.56 (d, J = 7.7 Hz, 1H), 2.26 (d, J = 1.8 Hz, 3H) ppm; ^{13}C NMR (126 MHz, DMSO-d_6) δ 140.1, 138.0, 130.9, 129.8, 119.8, 118.0, 20.8 ppm; HRMS calcd for $C_7H_9ClNO_3S$ $[M+H]^+$ 221.9986, found 221.9992.

2-Amino-4-bromobenzenesulfonic acid 2r

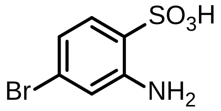

Prepared according to **GP-4** using *tert*-butyl (((4-bromorophenyl)sulfonyl)oxy)carbamate **1r** (88 mg, 0.25 mmol, 1.0 eq.), $FeSO_4 \cdot 7H_2O$ (0.70 mg, 2.5 μmol, 0.01 eq.), TFA (0.1 mL, 1.25 mmol, 5.0 eq.) and CH_2Cl_2 (5 mL). The crude residue was washed with MeOH to yield the title product as a white solid (44.0 mg, 0.175 mmol, 70%) as a >20:1 mixture of regioisomers.

^{1}H NMR (400 MHz, DMSO-d_6) δ 8.26 (br s, 3H), 7.55 (d, *J* = 8.6 Hz, 1H), 7.22 (s, 1H), 7.19 (d, *J* = 8.6 Hz, 1H) ppm; ^{13}C NMR (101 MHz, DMSO-d_6) δ 137.8, 135.6, 129.8, 125.1, 122.9, 122.8; HRMS calcd. for $C_6H_7BrNO_3S$ $[M+H]^+$ 251.9325, found 251.9327.

2-Amino-5-bromobenzenesulfonic acid 2s

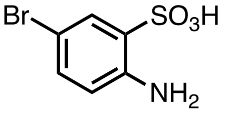

Prepared according to **GP-4** using *tert*-butyl (((3-bromophenyl)sulfonyl)oxy)carbamate **1s** (88 mg, 0.25 mmol, 1.0 eq.), $FeSO_4 \cdot 7H_2O$ (0.70 mg, 2.5 μmol, 0.01 eq.), TFA (0.1 mL, 1.25 mmol, 5.0 eq.) and CH_2Cl_2 (5 mL). The crude residue was washed with MeOH to yield the title product as a pale pink solid (32.1 mg, 0.128 mmol, 51%) as a >20:1 mixture of *o/o'* regioisomers (3.7:1 crude ratio).

^{1}H NMR (400 MHz, DMSO-d_6) δ 8.00 (br s, 3H), 7.76 (d, *J* = 2.4 Hz, 1H), 7.55 (dd, *J* = 8.5, 2.4 Hz, 1H), 7.10 (d, *J* = 8.5 Hz, 1H) ppm; ^{13}C NMR (101 MHz, DMSO-d_6) δ 139.7, 133.4, 133.0, 130.3, 124.2, 116.3 ppm.

Data is consistent with reported values.[5]

Note: the crude ^{1}H NMR indicated a 3.7:1 mixture of regioisomers

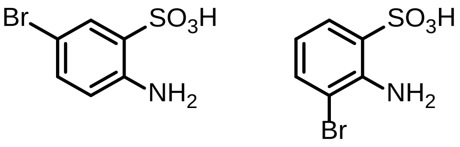

r.r. 3.7:1

2-Amino-4-methoxybenzenesulfonic acid and 3-amino-4-methoxybenzenesulfonic acid 2t

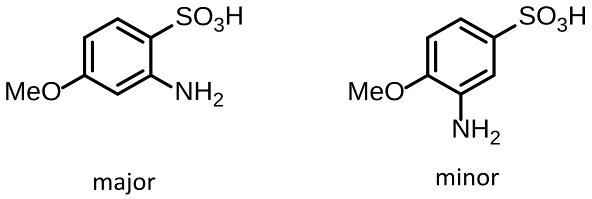

Prepared according to **GP-4** using *tert*-butyl (((4-methoxyphenyl)sulfonyl)oxy)carbamate **1t** (76 mg, 0.25 mmol, 1.0 eq.), $FeSO_4{\cdot}7H_2O$ (0.70 mg, 2.5 µmol, 0.01 eq.), TFA (0.1 mL, 1.25 mmol, 5.0 eq.) and CH_2Cl_2 (5 mL). The crude residue was washed with MeOH to yield the title products as a brown solid (19.4 mg, 0.096 mmol, 38%) as a 2.0:1 mixture of regioisomers (crude ratio 1.3:1).

Major isomer: ^{1}H NMR (500 MHz, DMSO-d_6) δ 7.59 (d, *J* = 8.6 Hz, 1H), 6.75 (dd, *J* = 8.6, 2.5 Hz, 1H), 6.67 (d, *J* = 2.5 Hz, 1H), 3.74 (s, 3H); ^{13}C NMR (101 MHz, DMSO-d_6) δ 160.6, 132.2, 131.3, 129.5, 110.3, 107.7, 55.9 ppm; *Minor isomer:* ^{1}H NMR (500 MHz, DMSO-d_6) δ 7.29 – 7.22 (m, 2H), 7.03 (dd, *J* = 8.7, 3.0 Hz, 1H), 3.78 (s, 3H); ^{13}C NMR (101 MHz, DMSO-d_6) δ 158.8, 142.3, 125.9, 120.5, 116.4, 112.6, 56.1 ppm; HRMS calcd. for $C_7H_9NNaO_4S$ $[M+Na]^+$ 226.0150, found 226.0148.

2-Amino-5-fluoro-4-methoxybenzenesulfonic acid 3-amino-5-fluoro-4-methoxybenzenesulfonic acid 2u

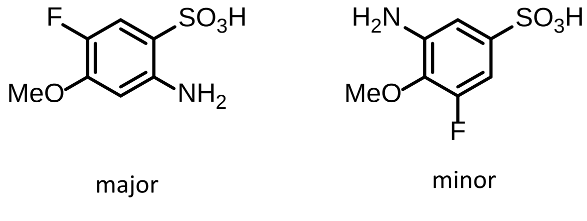

Prepared according to **GP-4** using *tert*-butyl (((3-fluoro-4-methoxyphenyl)sulfonyl)oxy)carbamate **1u** (80 mg, 0.25 mmol, 1.0 eq.), $FeSO_4{\cdot}7H_2O$ (0.70 mg, 2.5 µmol, 0.01 eq.), TFA (0.1 mL, 1.25 mmol, 5.0 eq.) and CH_2Cl_2 (5 mL). The crude residue was washed with acetone to yield the title products as a pale pink solid (32.5 mg, 0.147 mmol, 59%) as a 3.8:1 mixture of regioisomers (crude ratio 8.8:3:1).

Major isomer: ^{1}H NMR (500 MHz, DMSO-d_6) δ 7.44 (d, *J* = 11.3 Hz, 1H), 6.99 (d, *J* = 7.3 Hz, 1H), 3.85 (s, 3H) ppm; ^{13}C NMR (126 MHz, DMSO-d_6) δ 148.9 (d, *J* = 245.5 Hz), 148.6 (d, *J* = 11.5

Hz), 131.8 (d, J = 4.4 Hz), 127.5, 115.2 (d, J = 20.5 Hz), 108.5, 56.9 ppm; ^{19}F NMR (471 MHz, DMSO-d_6) δ -132.1 ppm; *Minor isomer:* ^{1}H NMR (500 MHz, DMSO-d_6) δ 7.44 (m, 1H), 7.18 (d, J = 11.5 Hz, 1H) 3.87 (s, 1H) ppm; ^{13}C NMR (126 MHz, DMSO-d_6) δ 151.6 (d, J = 248.0 Hz), 145.9 (d, J = 10.5 Hz), 136.9 (d, J = 3.8 Hz), 122.2 (d, J = 9.0 Hz), 112.9 (d, J = 2.7 Hz), 111.8 (d, J = 22.2 Hz), 56.3 ppm; ^{19}F NMR (471 MHz, DMSO-d_6) -138.5 ppm; HRMS calcd. for $C_7H_7FNO_4S^-$ [M-H]$^-$ 220.0080, found 220.0082.

Note: the crude ^{1}H NMR indicated a 8.8:3:1 mixture of regioisomers

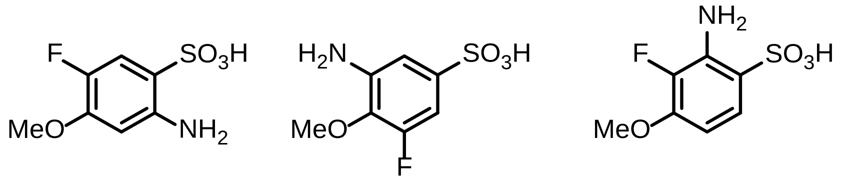

r.r. 8.8:3:1

2-Amino-4,5-dimethoxybenzenesulfonic acid 2v

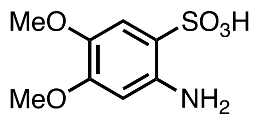

Prepared according to **GP-4** using *tert*-butyl (((3,4-dimethoxyphenyl)sulfonyl)oxy)carbamate **1v** (83 mg, 0.25 mmol, 1.0 eq.), $FeSO_4{\cdot}7H_2O$ (0.70 mg, 2.5 µmol, 0.01 eq.), TFA (0.1 mL, 1.25 mmol, 5.0 eq.) and CH_2Cl_2 (5 mL). The crude residue was washed with acetone to yield the title product as a pale pink solid (45.2 mg, 0.194 mmol, 78%) as a >20:1 mixture of *o/o'* regioisomers (crude ratio 7.7:1).

Major isomer: ^{1}H NMR (400 MHz, DMSO-d_6) δ 7.24 (s, 1H), 6.89 (s, 1H), 3.79 (s, 3H), 3.78 (s, 3H) ppm; ^{13}C NMR (101 MHz, DMSO-d_6) δ 150.0, 148.0, 133.2, 120.8, 110.5, 107.6, 56.4, 56.2 ppm; HRMS calcd. for $C_8H_{10}NO_5S^-$ [M-H]$^-$ 232.0280, found 232.0285.

Note: the crude ^{1}H NMR indicated a 7.7:1 mixture of regioisomers.

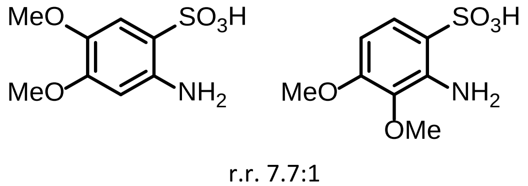

r.r. 7.7:1

3-Aminonaphthalene-2-sulfonic acid 2w

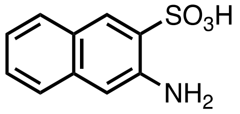

Prepared according to **GP-4** using *tert*-butyl ((naphthalen-2-ylsulfonyl)oxy)carbamate **1w** (81 mg, 0.25 mmol, 1.0 eq.), $FeSO_4{\cdot}7H_2O$ (0.70 mg, 2.5 µmol, 0.01 eq.), TFA (0.1 mL, 1.25 mmol, 5.0 eq.) and CH_2Cl_2 (5 mL). The crude residue was washed with MeOH to yield the title product as a pink solid (20.0 mg, 0.090 mmol, 31%) as a >20:1 mixture of regioisomers (1.3:1 crude ratio).

^{1}H NMR (500 MHz, DMSO-d_6) δ 8.36 (s, 1H), 8.08 (d, J = 8.0, 1.5 Hz, 1H), 7.97 (d, J = 8.0, 1.5 Hz, 1H), 7.82 (s, 1H), 7.67 – 7.49 (m, 2H), 7.01 (br s, 3H) ppm; ^{13}C NMR (126 MHz, DMSO-d_6) δ 138.4, 133.3, 131.3, 129.0, 128.4, 127.9, 127.7, 127.4, 127.3, 121.7 ppm; HRMS calcd. for $C_{10}H_8NO_3S^-$ [M-H]$^-$ 222.0225, found 222.0227.

Note: the crude ^{1}H NMR indicated a 1.3:1 mixture of regioisomers.

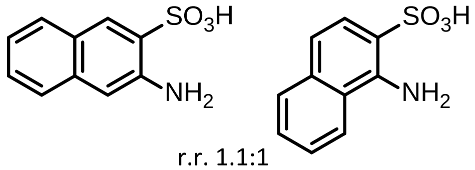

r.r. 1.1:1

2-Amino-5-(trifluoromethoxy)benzenesulfonic acid 2x

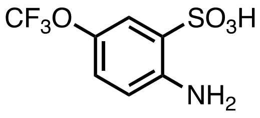

Prepared according to **GP-4** using *tert*-butyl (((3-(trifluoromethoxy)phenyl)sulfonyl)oxy)carbamate **1x** (89 mg, 0.25 mmol, 1.0 eq.), $FeSO_4{\cdot}7H_2O$ (0.70 mg, 2.5 µmol, 0.01 eq.), TFA (0.1 mL, 1.25 mmol, 5.0 eq.) and CH_2Cl_2 (5 mL). The crude residue was washed with acetone to yield the title product as a pink solid (31.5 mg, 0.122 mmol, 49%) as a >20:1 mixture of regioisomers (crude ratio 4.9:1).

^{1}H NMR (500 MHz, DMSO-d_6) δ 7.54 (d, J = 2.8 Hz, 1H), 7.36 (dd, J = 8.7, 2.8 Hz, 1H), 7.22 (d, J = 8.7 Hz, 1H) ppm; ^{13}C NMR (126 MHz, DMSO-d_6) δ 144.0, 138.8, 133.6, 123.5, 123.3, 120.6 (q, J = 256.3 Hz), 120.2 ppm; ^{19}F NMR (376 MHz, DMSO-d_6) δ -58.1 ppm; HRMS calcd. for $C_7H_5F_3NO_4S^-$ [M-H]$^-$ 225.9891, found 225.9896.

Note: the crude ^{1}H NMR indicated a 4.9:1 mixture of regioisomers.

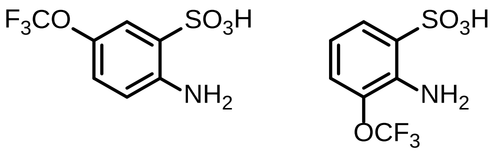

r.r. 4.9:1

2-Amino-4-(trifluoromethyl)benzenesulfonic acid 2y

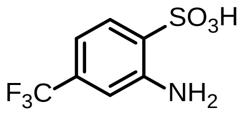

Prepared according to **GP-4** using *tert*-butyl (((4-(trifluoromethyl)phenyl)sulfonyl)oxy)carbamate **1y** (85 mg, 0.25 mmol, 1.0 eq.), $FeSO_4·7H_2O$ (0.70 mg, 2.5 µmol, 0.01 eq.), TFA (0.1 mL, 1.25 mmol, 5.0 eq.) and CH_2Cl_2 (5 mL). The crude residue was washed with acetone to yield the title product as a white solid (25.2 mg, 0.105 mmol, 42%) as a >20:1 mixture of regioisomers.

^{1}H NMR (500 MHz, DMSO-d_6) δ 7.63 (d, *J* = 7.8 Hz, 1H), 7.03 (s, 1H), 6.90 (d, *J* = 7.8 Hz, 1H) ppm; ^{13}C NMR (126 MHz, DMSO-d_6) δ 143.2, 135.5, 130.5 (q, *J* = 31.3 Hz), 128.7, 124.6 (q, *J* = 272.2 Hz), 113.4, 113.2 ppm; ^{19}F NMR (471 MHz, DMSO-d_6) δ -62.4 ppm; HRMS calcd. for $C_7H_6F_3KNO_3S$ $[M+K]^+$ 279.9652, found 279.9643.

2-Amino-4-(methoxycarbonyl)benzenesulfonic acid 2z

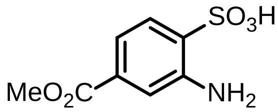

Prepared according to **GP-4** using methyl 4-((((tert-butoxycarbonyl)amino)oxy)sulfonyl)benzoate **1z** (66 mg, 0.20 mmol, 1.0 eq.), $FeSO_4·7H_2O$ (0.56mg, 2.0 µmol, 0.01 eq.), TFA (0.08 mL, 1.0 mmol, 5.0 eq.) and CH_2Cl_2 (4 mL). The crude residue was washed with methanol to yield the title product as a white solid (19.3 mg, 0.084 mmol, 42%) as a >20:1 mixture of regioisomers.

^{1}H NMR (500 MHz, DMSO-d_6) δ 7.75 (d, *J* = 8.0 Hz, 1H), 7.63 (s, 1H), 7.57 (d, *J* = 7.9 Hz, 1H), 3.86 (s, 3H); ^{13}C NMR (126 MHz, DMSO-d_6) δ 166.1, 138.8, 138.1, 131.4, 128.4, 121.8, 120.5, 52.8; HRMS calcd. for $C_8H_8NO_5S$ $[M-H]^-$ 230.0129, found 230.0128.

2-Amino-6-(trifluoromethyl)benzenesulfonic acid 2za

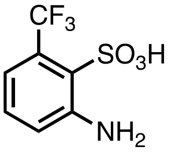

Prepared according to **GP-4** using *tert*-butyl (((2-(trifluoromethyl)phenyl)sulfonyl)oxy)carbamate **1za** (85 mg, 0.25 mmol, 1.0 eq.), $FeSO_4 \cdot 7H_2O$ (0.70 mg, 2.5 µmol, 0.01 eq.), TFA (0.1 mL, 1.25 mmol, 5.0 eq.) and CH_2Cl_2 (5 mL). The crude residue was washed with acetone to yield the title product as a white solid (23.5 mg, 0.097 mmol, 39%) as a >20:1 mixture of regioisomers.

^{1}H NMR (500 MHz, DMSO-d_6) δ 7.57 (d, J = 7.8 Hz, 1H), 7.53 (t, J = 7.8 Hz, 1H), 7.45 (d, J = 7.8 Hz, 1H), 6.97 (br s, 3H) ppm; ^{13}C NMR (126 MHz, DMSO-d_6) δ 136.7, 136.3, 130.3, 127.9 (q, J = 31.4 Hz), 127.1, 123.8 (q, J = 274.4 Hz), 123.5 (q, J = 7.4 Hz) ppm; ^{19}F NMR (471 MHz, DMSO-d_6) δ -55.6 ppm; HRMS calcd. for $C_7H_7F_3NO_3S$ $[M+H]^+$ 242.0093, found 242.0099.

2-Amino-5-(methylsulfonyl)benzenesulfonic acid 2zb

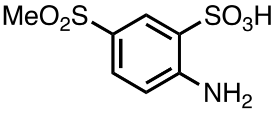

Prepared according to a modified version of **GP-4** using *tert*-butyl (((3-(methylsulfonyl)phenyl)sulfonyl)oxy)carbamate **1zb** (88 mg, 0.25 mmol, 1.0 eq.), TFA (0.1 mL, 1.25 mmol, 5.0 eq.) and CH_2Cl_2 (5 mL). The crude residue was washed with acetone to yield the title product as a white solid (23.9 mg, 0.095 mmol, 38%) as a 16:1 mixture of regioisomers (crude ratio 4.5:1).

^{1}H NMR (400 MHz, DMSO-d_6) δ 7.91 (d, J = 2.4 Hz, 1H), 7.51 (dd, J = 8.6, 2.4 Hz, 1H), 7.08 (br s, 3H), 6.77 (d, J = 8.6 Hz, 1H), 3.05 (s, 3H) ppm; ^{13}C NMR (101 MHz, DMSO-d_6) δ 149.7, 129.9, 129.3, 127.6, 125.6, 115.6, 45.0 ppm; HRMS calcd. for $C_7H_8NO_5S_2^-$ $[M-H]^-$ 249.9844, found 249.9848.

Note: the crude ^{1}H NMR indicated a 4.5:1 mixture of regioisomers.

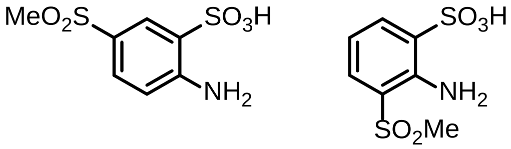

r.r. 4.5:1

2-Amino-4-nitrobenzenesulfonic acid 2zc

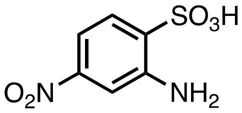

A solution of *O*-((4-nitrophenyl)sulfonyl)hydroxylammonium trifluoromethanesulfonate **1zc** (92 mg, 0.25 mmol, 1.0 eq.) in HFIP (1.25 mL) was heated to 60 °C for 18 h. The reaction was cooled to rt and the solvent was removed under a stream of compressed air. The crude residue was washed with acetone to yield the title product as a yellow solid (44.0 mg, 0.202 mmol, 81%) as a >20:1 mixture of regioisomers.

^{1}H NMR (400 MHz, DMSO-*d*$_6$) δ 7.68 (d, *J* = 8.5 Hz, 1H), 7.58 (d, *J* = 2.1 Hz, 1H), 7.39 (dd, *J* = 8.5, 2.1 Hz, 1H), 7.33 (br s, 3H) ppm; ^{13}C NMR (101 MHz, DMSO-*d*$_6$) δ 148.7, 144.4, 137.2, 129.0, 111.0, 110.9 ppm; HRMS calcd. for $C_6H_5N_2O_5S$ [M-H]$^-$ 216.9919, found 216.9922.

(2-Aminophenyl)methanesulfonic acid 4a

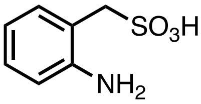

Prepared according to **GP-4** using *tert*-butyl ((benzylsulfonyl)oxy)carbamate **3a** (72 mg, 0.25 mmol, 1.0 eq.), $FeSO_4{\cdot}7H_2O$ (0.70 mg, 2.5 µmol, 0.01 eq.), TFA (0.1 mL, 1.25 mmol, 5.0 eq.) and CH_2Cl_2 (5 mL). The crude residue was washed with acetone to yield the title product as a pink solid (38.9 mg, 0.208 mmol, 83%) as a >20:1 mixture of regioisomers.

^{1}H NMR (400 MHz, DMSO-*d*$_6$) δ 9.87 (br s, 3H), 7.35 (m, 4H), 4.01 (s, 2H) ppm; ^{13}C NMR (101 MHz, DMSO-*d*$_6$) δ 133.7, 131.7, 129.6, 128.7, 128.6, 123.9, 54.3 ppm; HRMS calcd. for $C_7H_8NO_3S$ [M-H]$^-$ 186.0225, found 186.0228.

(2-Amino-4-chlorophenyl)methanesulfonic acid 4b

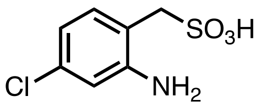

Prepared according to **GP-4** using *tert*-butyl (((4-chlorobenzyl)sulfonyl)oxy)carbamate **3b** (72 mg, 0.25 mmol, 1.0 eq.), $FeSO_4·7H_2O$ (0.70 mg, 2.5 µmol, 0.01 eq.), TFA (0.1 mL, 1.25 mmol, 5.0 eq.) and CH_2Cl_2 (5 mL). The crude residue was washed with acetone to yield the title product as a brown solid (43.2 mg, 0.195 mmol, 78%) as a >20:1 mixture of regioisomers.

^{1}H NMR (500 MHz, DMSO-*d*$_6$) δ 7.34 (d, *J* = 8.9 Hz, 1H), 7.30 – 7.26 (m, 2H), 6.35 (br s, 3H), 3.99 (s, 2H) ppm; ^{13}C NMR (101 MHz, DMSO-*d*$_6$) δ 135.1, 135.0, 132.4, 127.9, 127.1, 122.8, 53.8 ppm; HRMS calcd. for $C_7H_9ClNO_3S$ [M+H]$^+$ 221.9986, found 221.9994.

(2-Amino-6-chlorophenyl)methanesulfonic acid 4c

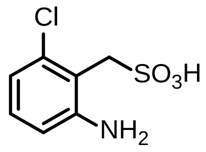

Prepared according to **GP-4** using *tert*-butyl (((2-chlorobenzyl)sulfonyl)oxy)carbamate **3c** (64 mg, 0.20 mmol, 1.0 eq.), $FeSO_4·7H_2O$ (0.56 mg, 2.0 µmol, 0.01 eq.), TFA (0.08 mL, 1.00 mmol, 5.0 eq.) and CH_2Cl_2 (4 mL). The crude residue was washed with methanol to yield the title product as a white solid (26.1 mg, 0.118 mmol, 59%) as a >20:1 mixture of regioisomers.

^{1}H NMR (400 MHz, DMSO-*d*$_6$) δ 7.44 (d, *J* = 7.9 Hz, 1H), 7.35 (t, *J* = 7.9 Hz, 1H), 7.29 (d, *J* = 7.8 Hz, 1H), 4.19 (s, 2H). ^{13}C NMR (126 MHz, DMSO-*d*$_6$) δ 136.0, 135.6, 129.5, 128.4, 126.6, 122.2, 50.6 ppm; HRMS calcd. for $C_7H_9ClNO_3S$ [M+H]$^+$ 221.9986, found 221.9990.

(2-Amino-4-methylphenyl)methanesulfonic acid 4d

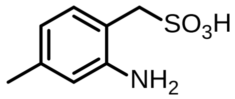

Prepared according to **GP-4** using *tert*-butyl (((4-methylbenzyl)sulfonyl)oxy)carbamate **3d** (60 mg, 0.20 mmol, 1.0 eq.), $FeSO_4·7H_2O$ (0.56 mg, 2.0 µmol, 0.01 eq.), TFA (0.08 mL, 1.00 mmol, 5.0 eq.) and CH_2Cl_2 (4 mL). The crude residue was washed with acetone to yield the title product as a white solid (33.4 mg, 0.166 mmol, 83%) as a >20:1 mixture of regioisomers.

^{1}H NMR (400 MHz, DMSO-d_6) δ 7.25 (d, J = 7.7 Hz, 1H), 7.19 (d, J = 8.1 Hz, 1H), 7.16 (s, 1H), 3.99 (s, 2H), 2.32 (s, 3H) ppm; ^{13}C NMR (101 MHz, DMSO-d_6) δ 138.5, 133.5, 131.1, 129.4, 126.5, 124.3, 53.9, 21.0 ppm; HRMS calcd. for $C_8H_{12}NO_3S$ $[M+H]^+$ 202.0532, found 202.0538.

(2-Amino-3,5-dimethoxyphenyl)methanesulfonic acid 4e

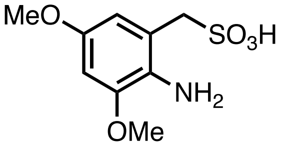

Prepared according to **GP-4** using *tert*-butyl (((3,5-dimethoxybenzyl)sulfonyl)oxy)carbamate **3e** (87 mg, 0.25 mmol, 1.0 eq.), $FeSO_4{\cdot}7H_2O$ (0.70 mg, 2.5 µmol, 0.01 eq.), TFA (0.1 mL, 1.25 mmol, 5.0 eq.) and CH_2Cl_2 (5 mL). The crude residue was washed with acetone to yield the title product as a pink solid (30.6 mg, 0.120 mmol, 49%) as a >20:1 mixture of regioisomers.

^{1}H NMR (500 MHz, DMSO-d_6) δ 6.70 (d, J = 2.6 Hz, 1H), 6.56 (d, J = 2.6 Hz, 1H), 4.00 (s, 2H), 3.89 (s, 3H), 3.78 (s, 3H) ppm; ^{13}C NMR (126 MHz, DMSO-d_6) δ 159.8, 153.6, 131.1, 112.4, 109.9, 98.7, 57.0, 56.1, 54.5 ppm; HRMS calcd. for $C_9H_{14}NO_5S$ $[M+H]^+$ 248.0587, found 248.0585.

(2-Amino-4-bromophenyl)methanesulfonic acid 4f

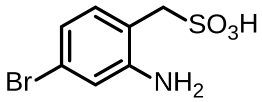

Prepared according to **GP-4** using *tert*-butyl (((4-bromobenzyl)sulfonyl)oxy)carbamate **3f** (110 mg, 0.30 mmol, 1.0 eq.), $FeSO_4{\cdot}7H_2O$ (0.84 mg, 3.0 µmol, 0.01 eq.), TFA (0.12 mL, 1.5 mmol, 5.0 eq.) and CH_2Cl_2 (6 mL). The crude residue was washed with methanol to yield the title product as a white solid (51.4 mg, 0.193 mmol, 64%) as a >20:1 mixture of regioisomers.

^{1}H NMR (400 MHz, DMSO-d_6) δ 7.54 – 7.47 (m, 2H), 7.38 (br s, 3H), 7.31 (d, J = 8.1 Hz, 1H), 3.99 (s, 2H) ppm; ^{13}C NMR (101 MHz, DMSO-d_6) δ 135.3, 134.8, 130.4, 128.5, 125.9, 120.5, 53.8 ppm; HRMS calcd. for $C_7H_9BrNO_3S$ $[M+H]^+$ 265.9481, found 265.9486.

(2-Amino-5-bromophenyl)methanesulfonic acid and (2-amino-3-bromophenyl)methanesulfonic acid 4g

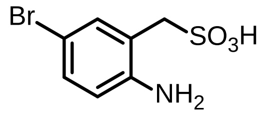

major

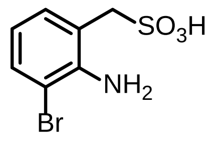

minor

Prepared according to **GP-4** using *tert*-butyl (((4-bromobenzyl)sulfonyl)oxy)carbamate **3g** (92 mg, 0.25 mmol, 1.0 eq.), $FeSO_4 \cdot 7H_2O$ (0.70 mg, 2.5 µmol, 0.01 eq.), TFA (0.1 mL, 1.25 mmol, 5.0 eq.) and CH_2Cl_2 (5 mL). The crude residue was washed with acetone to yield the title products as a white solid (25.6 mg, 0.096 mmol, 38%) as a 6.8:1 mixture of regioisomers (crude ratio 5.0:1).

Major isomer: ^{1}H NMR (500 MHz, DMSO-d_6) δ 7.61 (s, 1H), 7.58 (d, J = 8.4 Hz, 1H), 7.29 (d, J = 8.4 Hz, 1H), 4.03 (s, 2H) ppm; ^{13}C NMR (126 MHz, DMSO) δ 143.5, 135.9, 132.0, 131.9, 131.4, 125.8, 53.7 ppm; *Minor isomer:* ^{1}H NMR (500 MHz, DMSO-d_6) δ 7.32 (dd, J = 7.8, 1.5 Hz, 1H), 7.01 (dd, J = 7.8, 1.5 Hz, 1H), 6.61 (t, J = 7.8 Hz, 1H), 3.81 (s, 2H) ppm; ^{13}C NMR (126 MHz, DMSO) δ 145.7, 132.1, 131.0, 124.0, 119.8, 111.0, 55.7; HRMS calcd. for $C_7H_9BrNO_3S$ $[M+H]^+$ 265.9481, found 265.9481.

(2-Amino-4-fluorophenyl)methanesulfonic acid 4h

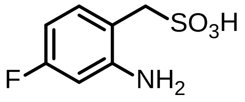

Prepared according to **GP-4** using *tert*-butyl (((4-fluorobenzyl)sulfonyl)oxy)carbamate **3h** (87 mg, 0.25 mmol, 1.0 eq.), $FeSO_4 \cdot 7H_2O$ (0.70 mg, 2.5 µmol, 0.01 eq.), TFA (0.1 mL, 1.25 mmol, 5.0 eq.) and CH_2Cl_2 (5 mL). The crude residue was washed with methanol to yield the title product as a white solid (25.8 mg, 0.126 mmol, 50%) as a >20:1 mixture of regioisomers.

^{1}H NMR (500 MHz, DMSO-d_6) δ 7.34 (dd, J = 9.2, 5.9 Hz, 1H), 7.14 – 6.98 (m, 2H), 3.95 (s, 2H) ppm; ^{13}C NMR (126 MHz, DMSO-d_6) δ 161.4 (d, J = 244.4 Hz), 135.1 (d, J = 8.9 Hz), 134.6 (d, J = 10.3 Hz), 125.3 (d, J = 3.3 Hz), 114.3 (d, J = 20.7 Hz), 110.6 (d, J = 25.3 Hz), 53.6 ppm; ^{19}F NMR (471 MHz, DMSO-d_6) δ -114.74 ppm; HRMS calcd. for $C_7H_{10}FNO_3S$ $[M+H]^+$ 206.0282, found 206.0276.

(2-Amino-4-(trifluoromethyl)phenyl)methanesulfonic acid 4i

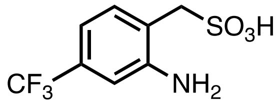

Prepared according to **GP-4** using *tert*-butyl (((2-chlorobenzyl)sulfonyl)oxy)carbamate **3i** (71 mg, 0.20 mmol, 1.0 eq.), $FeSO_4 \cdot 7H_2O$ (0.56 mg, 2.0 µmol, 0.01 eq.), TFA (0.08 mL, 1.00 mmol, 5.0 eq.) and CH_2Cl_2 (4 mL). The crude residue was washed with acetone to yield the title product as a white solid (13.8 mg, 0.054 mmol, 27%) as a >20:1 mixture of regioisomers.

^{1}H NMR (500 MHz, DMSO-d_6) δ 7.54 – 7.38 (m, 3H), 4.02 (s, 2H) ppm; ^{13}C NMR (126 MHz, DMSO-d_6) δ 137.5, 134.3, 131.8, 128.7 (q, J = 32.0 Hz), 124.3 (q, J = 272.1 Hz), 121.7, 118.2, 54.4 ppm; ^{19}F NMR (471 MHz, DMSO-d_6) δ -62.2 ppm; HRMS calcd. for $C_8H_9F_3NO_3S$ $[M+H]^+$ 256.0250, found 256.0258.

7. Product manipulations

2-Aminobenzenesulfonyl chloride 8a

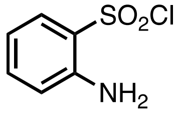

To a vial containing chlorosulfonic acid (0.5 mL) at 0 °C was added portionwise 2-aminobenzenesulfonic acid (87 mg, 0.5 mmol, 1.0 eq.). The reaction was stirred at 0 °C for 1 h and then heated to 80 °C for a further 2 h. The solution was poured onto crushed ice and the aqueous was extracted with CH_2Cl_2 (3 x 10 mL), the organics were combined, dried ($MgSO_4$) and concentrated. The crude product was purified by silica gel column chromatography (Pet. Ether/EtOAc 8:2) to yield the title product as a yellow solid (39.3 mg, 0.205 mmol, 41%).

^{1}H NMR (400 MHz, $CDCl_3$) δ 7.81 (d, *J* = 8.4 Hz, 1H), 7.45 (t, *J* = 7.8 Hz, 1H), 6.89 – 6.73 (m, 2H), 5.26 (br s, 2H); ^{13}C NMR (101 MHz, $CDCl_3$) δ 145.7, 136.9, 129.0, 125.6, 118.0, 117.4 ppm; HRMS calcd. for $C_6H_7ClNO_2S$ $[M+H]^+$ 191.9881, found 191.9882.

***N*-Allyl-2-aminobenzenesulfonamide 8b**

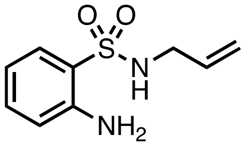

To a solution of 2-aminobenzenesulfonyl chloride **8a** (38 mg, 0.2 mmol, 1.0 eq.) in Et_2O (1 mL) at 0 °C was added NEt_3 (31 µL, 0.22 mmol, 1.1 eq.) and allyl amine (45 µL, 0.6 mmol, 3.0 eq.). The reaction was stirred at room temperature for 16 h, then filtered to remove solids. The organic layer was washed with $NaHCO_3$ aq. (5 mL), dried ($MgSO_4$) and concentrated. Purification by silica gel column chromatography (Pet. Ether/EtOAc 8:2) yielded the title product as a yellow oil (25.5 mg, 0.120 mmol, 60%).

^{1}H NMR (500 MHz, $CDCl_3$) δ 7.73 (dd, *J* = 8.0, 1.6 Hz, 1H), 7.35 (ddd, *J* = 8.1, 7.3, 1.6 Hz, 1H), 6.83 (ddd, *J* = 8.2, 7.2, 1.0 Hz, 1H), 6.79 (dd, *J* = 8.2, 1.0 Hz, 1H), 5.70 (ddt, *J* = 17.1, 10.2, 5.8 Hz, 1H), 5.18 (dq, *J* = 17.1, 1.6 Hz, 1H), 5.09 (dq, *J* = 10.2, 1.3 Hz, 1H), 4.88 (br s, 3H), 3.53 (td, *J* = 6.1, 3.0 Hz, 2H) ppm; ^{13}C NMR (126 MHz, $CDCl_3$) δ 145.1, 134.3, 132.9, 129.7, 121.7, 118.0, 117.9, 117.7, 45.8 ppm.

Data is consistent with reported values.[6]

2-(Methylsulfonyl)aniline 8c

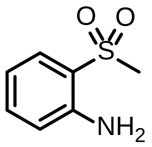

A suspension of 2-aminobenzenesulfonyl chloride **8a** (192 mg, 1.0 mmol, 1 eq), sodium sulfite (252 mg, 2.0 mmol, 2 eq) and sodium hydrogen carbonate (168 mg, 2.0 mmol, 2 eq) in distilled water (1 mL) was stirred at 80 °C for 4 h. The resulting yellow solution was then diluted with aqueous sodium hydroxide (2.5 M, 5 mL). Tetrabutylammoniumhydrogen sulfate (339 mg, 1.0 mmol, 1 eq) was then added. The aqueous layer was then extracted with chloroform (3 x 5 mL). The combined organics were then dried ($MgSO_4$), filtered and concentrated *in vacuo*. The resulting residue was then dissolved in acetone (1 mL) and toluene (1 mL) and methyl iodide (114 mg, 0.80 mmol, 0.8 eq) added. The reaction was then stirred at 80 ℃ for 18 h. The reaction was then concentrated *in vacuo*. The resulting residue was then purified by silica gel chromatography (Pet. Ether/EtOAc 100:0 – 70:30) to give the title compound (67.5 mg, 0.39 mmol, 49%) as a white solid.

^{1}H NMR (400 MHz, $CDCl_3$) δ 7.74 (dd, *J* = 8.0, 1.6 Hz, 1H), 7.37 (t, *J* = 8.5 Hz, 1H), 6.83 (t, *J* = 7.5 Hz, 1H), 6.78 (dd, *J* = 8.2, 1.0 Hz, 1H), 5.05 (s, 2H), 3.07 (s, 3H) ppm; ^{13}C NMR (101 MHz, $CDCl_3$) δ 146.2, 135.2, 129.4, 121.9, 118.0, 117.6, 42.2 ppm.

Data is consistent with reported values.[7]

2-Iodobenzenesulfonic acid 8d

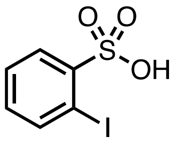

To a solution of 2-aminobenzenesulfonic acid (174 mg, 1.0 mmol, 1.0 eq.) in water (1.7 mL) was added slowly $NaHCO_3$ (52 mg, 0.5 mmol, 0.5 eq.). Once gas evolution had ceased, the reaction was cooled to 0 °C and $NaNO_2$ (76 mg, 1.1 mmol, 1.1 eq.) was added in portions. After 30 minutes, HCl (37%, 0.2 mL, 2.0 mmol, 2.0 eq.) was added and after a further 30 minutes a solution of KI (200 mg, 1.2 mmol, 1.2 eq.) in water was added dropwise. The reaction mixture was warmed to room temperature and stirred for 19 h. The solvent was removed under a stream of compressed air and the crude product was recrystallised from hot water to yield the title product as brown crystals (200 mg, 0.70 mmol, 70%).

^{1}H NMR (400 MHz, D_2O) δ 8.07 (d, *J* = 7.8 Hz, 1H), 7.95 (d, *J* = 7.8 Hz, 1H), 7.46 (t, *J* = 7.8 Hz, 1H), 7.17 (t, *J* = 7.8 Hz, 1H) ppm; ^{13}C NMR (101 MHz, D_2O) δ 144.8, 142.1, 132.4, 128.5, 128.3, 90.9 ppm.

Data is consistent with reported values.[8]

2-Acetamidobenzenesulfonic acid 8e

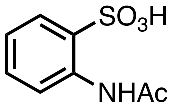

To a solution of 2-aminobenzenesulfonic acid (87 mg, 0.5 mmol, 1.0 eq.) in acetic acid (6 mL) was added slowly acetyl chloride (0.05 mL, 0.6 mmol, 1.1 eq.). The reaction was heated to 100 °C for 18 h and then cooled to room temperature. The solvent was evaporated to yield the title product as a white solid (102 mg, 0.474 mmol, 95%).

^{1}H NMR (400 MHz, DMSO-d_6) δ 10.42 (br s, 1H), 8.28 (d, *J* = 8.2 Hz, 1H), 7.66 (d, *J* = 7.7 Hz, 1H), 7.30 (t, *J* = 7.8 Hz, 1H), 7.02 (t, *J* = 7.5 Hz, 1H), 2.06 (s, 3H) ppm; ^{13}C NMR (126 MHz, DMSO-d_6) δ 168.0, 135.7, 135.6, 130.0, 127.3, 122.6, 119.9, 25.4 ppm.

Data is consistent with reported values.[9]

Sodium 2-aminobenzenesulfinate 8f

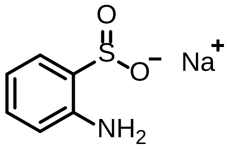

A suspension of 2-aminobenzenesulfonyl chloride **8a** (480mg, 2.5 mmol, 1 eq), sodium sulfite (630 mg, 5.0 mmol, 2 eq) and sodium hydrogen carbonate (420 mg, 5.0 mmol, 2 eq) in distilled water (2.5 mL) was stirred at 80 °C for 4 h. The resulting solution was then concentrated *in vacuo*. Ethanol (5 mL) was then added to the resulting residue and the suspension was concentrated *in vacuo*. This was repeated three times. The resulting solid was then washed with ethanol (25 mL) and the resulting suspension filtered. The filtrate was concentrated in vacuo to give the title compound (124 mg, 0.693 mmol, 28%) as a white solid.

^{1}H NMR (400 MHz, D_2O) δ 7.42 – 7.33 (m, 1H), 7.24 – 7.16 (m, 1H), 6.85 (t, *J* = 7.5 Hz, 1H), 6.77 (d, *J* = 8.1 Hz, 1H) ppm; ^{13}C NMR (101 MHz, D_2O) δ 143.8, 136.9, 131.4, 124.1, 119.1, 117.9 ppm; HRMS calcd. for $C_6H_6NO_2S$ $[M - Na^+]^-$ 156.0125, found 156.0124.

2-(Phenylsulfonyl)aniline 8g

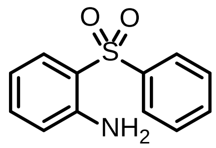

Under an atmosphere of nitrogen, sodium 2-aminobenzenesulfinate **8f** (18.0 mg, 0.10 mmol, 1 eq) and diphenyliodonium triflate (47.3 mg, 0.11 mmol, 1.1 eq) were stirred in dry DMF at 90 °C for 18 h. The reaction was quenched with aqueous sodium hydrogen carbonate (2.5 M, 3 mL). The aqueous layer was then extracted with chloroform (3 x 2 mL) and the combined organics were dried ($MgSO_4$), filtered and concentrated *in vacuo*. The resulting residue was then purified by silica gel chromatography (Pet. Ether/EtOAc 100:0 – 60:40) to give the title compound (12.9 mg, 0.055 mmol, 55%) as a white solid.

^{1}H NMR (400 MHz, $CDCl_3$) δ 8.02 – 7.88 (m, 2H), 7.86 (dd, *J* = 8.1, 1.6 Hz, 1H), 7.58 (t, *J* = 7.4 Hz, 1H), 7.51 (t, *J* = 7.5 Hz, 2H), 7.39 – 7.28 (m, 1H), 6.81 (td, *J* = 7.6, 7.0, 1.2 Hz, 1H), 6.67 (dd, *J* = 8.2, 1.2 Hz, 1H), 5.15 (s, 2H) ppm; ^{13}C NMR (126 MHz, $CDCl_3$) δ 146.2, 141.8, 135.0, 133.1, 130.0, 129.0, 126.9, 121.9, 117.8, 117.7 ppm.

Data is consistent with reported values.[10]

6-Bromo-1,3-dihydrobenzo[c]isothiazole 2,2-dioxide 9

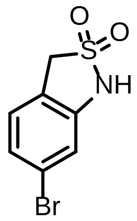

Phosphorous oxychloride (47 µL, 0.50 mmol, 5 eq) was added to a stirred suspension of (2-amino-4-bromophenyl)methanesulfonic acid **4f** (26.6 mg, 0.10 mmol, 1 eq) in dry acetonitrile (1 mL). The reaction was heated at 55 °C for 18 h. The reaction was then concentrated under a stream of air and then water (2 mL) was added. The aqueous layer was then extracted with chloroform (3 x 2 mL) and the combined organics were dried ($MgSO_4$), filtered and concentrated *in vacuo*. The resulting residue was then purified by silica gel chromatography (Pet. Ether/EtOAc 100:0 – 70:30) to give the title compound (17.1 mg, 0.69 mmol, 69%) as a white solid.

^{1}H NMR (400 MHz, $CDCl_3$) δ 7.21 (dd, *J* = 8.2, 1.8 Hz, 1H), 7.13 (d, *J* = 8.1 Hz, 1H), 7.05 (d, *J* = 1.7 Hz, 1H), 6.88 (s, 2H), 4.35 (s, 1H) ppm; ^{13}C NMR (101 MHz, $CDCl_3$) δ 139.9, 126.9, 126.2, 123.0, 118.1, 115.9, 52.0 ppm; HRMS calcd. for $C_7H_5BrNO_2S$ $[M-H]^-$ 245.9230, found 245.9221.

8. Mechanistic experiments

Establishing viability of NHMe transfer:

Triethylammonium 2-(methylamino)benzenesulfonate 5aa

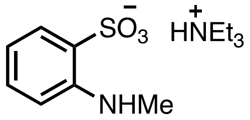

tert-Butyl methyl((phenylsulfonyl)oxy)carbamate **5a** (72 mg, 0.25 mmol, 1.0 eq.), TFA (0.1 mL, 1.25 mmol, 5.0 eq.) and CH_2Cl_2 (5 mL) were subjected to the conditions described in **GP-4**. After 18 h, the solvent was removed under a stream of compressed air, the crude residue was dissolved in MeOH and NEt_3 (0.2 mL) was added. The solvent was removed under a stream of compressed air and the reaction was analysed by ^{1}H NMR in comparison with 1,2-dimethoxyethane as an internal standard (20% NMR yield). A pure sample for analysis could be obtained by silica gel column chromatography (CH_2Cl_2/MeOH/NEt_3 97.5:2.5:0.5).

^{1}H NMR (500 MHz, $CDCl_3$) δ 10.87 (br s, 1H), 7.80 (dd, *J* = 7.7, 1.6 Hz, 1H), 7.33 – 7.29 (m, 1H), 6.77 (dd, *J* = 8.3, 1.1 Hz, 1H), 6.73 (td, *J* = 7.5, 1.1 Hz, 1H), 3.16 (m, 6H), 2.93 (s, 3H), 1.39 (t, *J* = 7.0 Hz, 12H) ppm; ^{13}C NMR (126 MHz, $CDCl_3$) δ 145.0, 131.7, 128.9, 127.7, 127.7, 116.4, 112.1, 46.2, 31.2, 8.7 ppm; HRMS calcd. for $C_7H_8NO_3S$ $[M\text{-}HNEt_3]^-$ 186.0230, found 186.0232.

Triethylammonium 4-methyl-2-(methylamino)benzenesulfonate 1ba

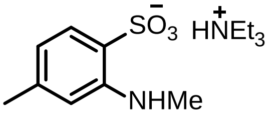

tert-Butyl methyl(tosyloxy)carbamate (75 mg, 0.25 mmol, 1.0 eq.), TFA (0.1 mL, 1.25 mmol, 5.0 eq.) and CH_2Cl_2 (5 mL) were subjected to the conditions described in **GP-4**. After 18 h, the solvent was removed under a stream of compressed air, the crude residue was dissolved in MeOH and NEt_3 (0.2 mL) was added. The solvent was removed under a stream of compressed air and the reaction was analysed by ^{1}H NMR in comparison with 1,2-dimethoxyethane as an internal standard (25% NMR yield). A pure sample for analysis could be obtained by silica gel column chromatography (CH_2Cl_2/MeOH/NEt_3 97.5:2:0.5 to 96:3.5:0.5).

^{1}H NMR (500 MHz, MeOD-d_4) δ 7.58 (d, *J* = 7.9 Hz, 1H), 6.60 (s, 1H), 6.54 (d, *J* = 7.8 Hz, 1H), 3.20 (q, *J* = 7.3 Hz, 6H), 2.89 (s, 3H), 2.31 (s, 3H), 1.31 (t, *J* = 7.3 Hz, 9H) ppm; ^{13}C NMR (126

MHz, MeOD-d_4) δ 144.8, 142.0, 127.9, 127.5, 116.9, 112.2, 46.5, 29.9, 20.3, 7.8 ppm; HRMS calcd. for $C_8H_{12}NO_3S$ $[M+2H]^+$ 202.0532, found 202.0534.

Crossover experiment (Scheme 3A):

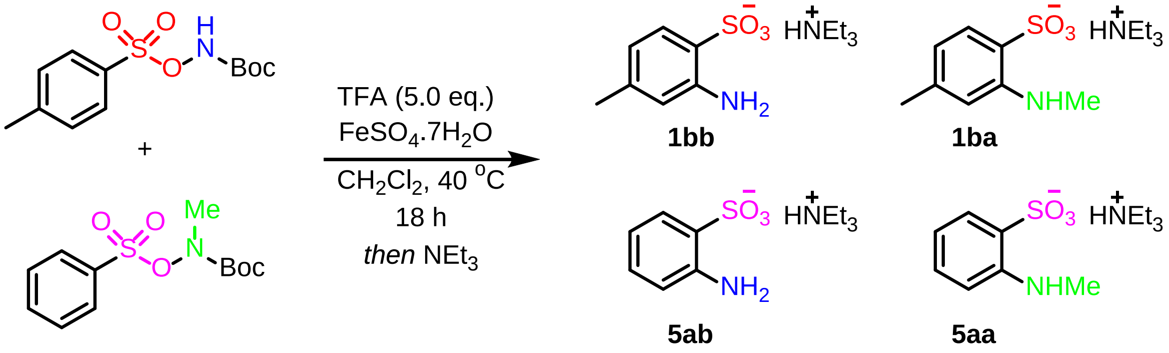

The crossover experiment was performed according to **GP-4** using *tert*-butyl (tosyloxy)carbamate **1b** (36 mg, 0.05 mmol, 0.5 eq.), *tert*-butyl methyl((phenylsulfonyl)oxy)carbamate **5a** (36 mg, 0.05 mmol, 0.5 eq.), $FeSO_4{\cdot}7H_2O$ (0.70 mg, 2.5 µmol, 0.01 eq.), TFA (0.1 mL, 1.25 mmol, 5.0 eq.) and CH_2Cl_2 (5 mL). The crude reaction mixture was deprotonated using NEt_3 (0.2 mL), filtered through a plug of silica with the aid of CH_2Cl_2/MeOH (9:1) and analysed by ^1H NMR.

The crude reaction mixture indicated the formation of products **5aa, 5ab, 1ba** and **1bb** in a ratio of 2:1:1:2, indicating that crossover had occurred and suggesting an intermolecular reaction mechanism. The identities of the products formed in the crossover experiment were determined by comparison with the ^1H NMR spectra of known authentic isolated samples. The remaining mass balance consisted of sulfonic acid derived from both starting materials which had not undergone amination.

Crude NMR spectrum (400 MHz, MeOD-d_4):

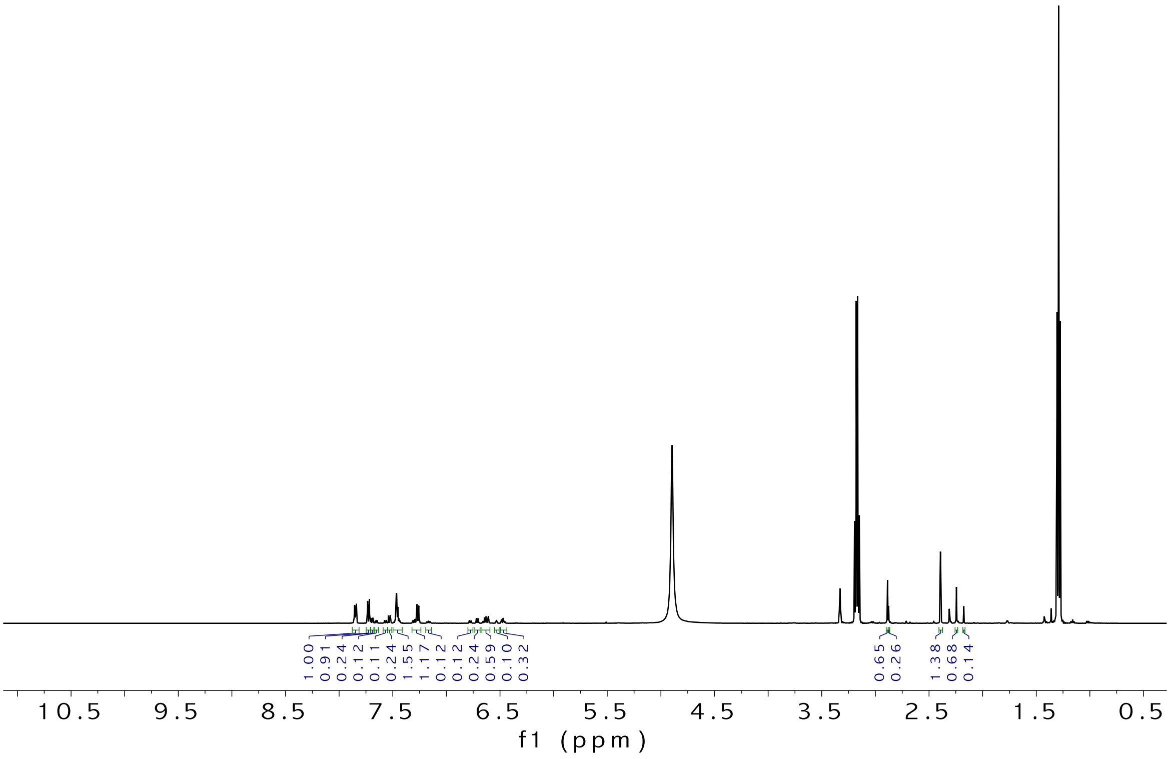

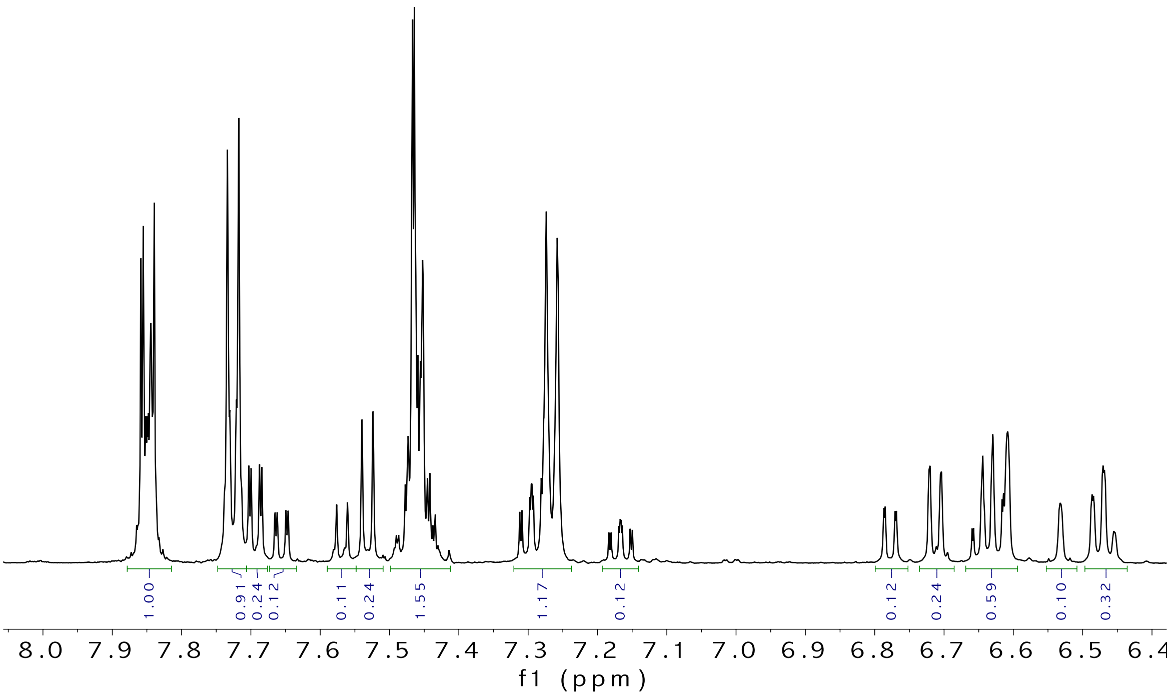

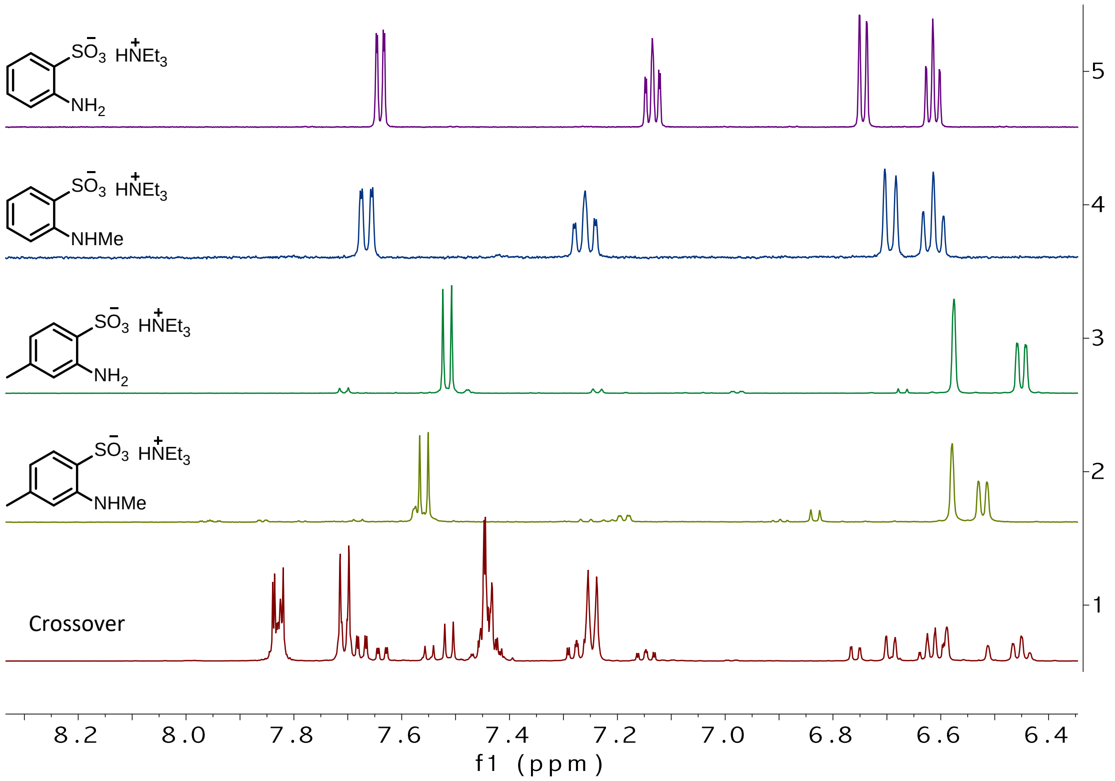
SO3 HNEt3
NH2
SO3 HNEt3
NHMe
SO3 HNEt3
NH2
SO3 HNEt3
NHMe
Crossover
5
4
3
2
1
8.2
8.0
7.8
7.6
7.4
7.2
7.0
6.8
6.6
6.4
f1 (ppm)

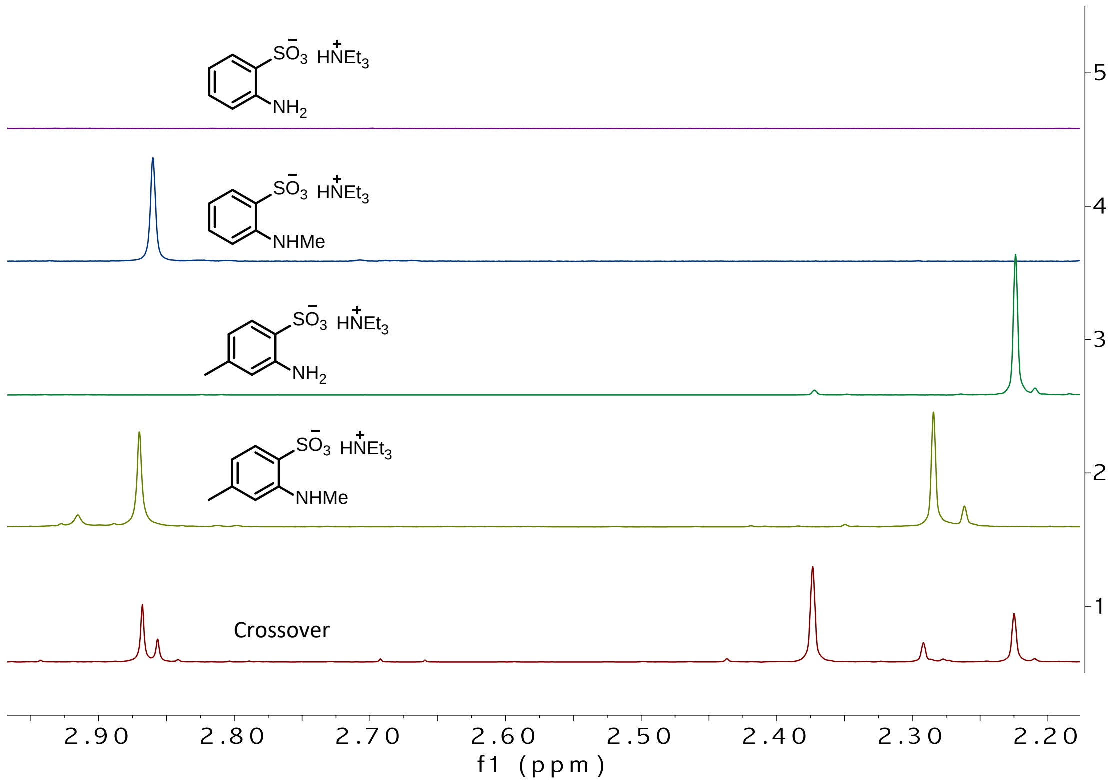
SO3 HNEt3
NH2
SO3 HNEt3
NHMe
SO3 HNEt3
NH2
SO3 HNEt3
NHMe
Crossover
5
4
3
2
1
2.90
2.80
2.70
2.60
2.50
2.40
2.30
2.20
f1 (ppm)

Intermolecular experiment (Scheme 3B):

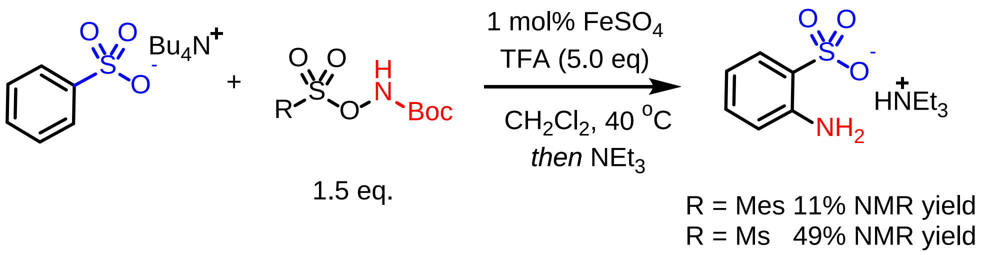

To a solution of tetrabutylammonium benzenesulfonate **6** (40.0 mg, 0.1 mmol, 1.0 eq.) and aminating agent (0.15 mmol, 1.5 eq.) in CH_2Cl_2 (2 mL) was added TFA (0.04 mL, 0.5 mmol, 5.0 eq.). The reaction was heated to 40 °C for 18 h, then cooled to room temperature and the volatiles were removed under a stream of compressed air. To the crude reaction mixture was added MeOH (0.5 mL) and NEt_3 (0.2 mL). The solvent was removed under a stream of compressed air and the reaction mixture was analysed by ^1H NMR in comparison with 1,2-dimethoxyethane as an internal standard (NMR yields; when R = Mes 11%, when R = Ms 49%).

Tetrabutylammonium benzenesulfonate 6

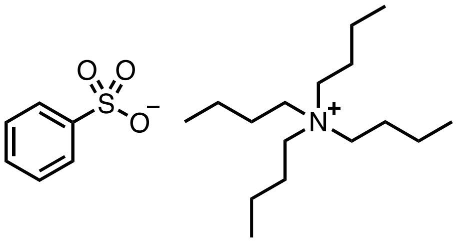

Prepared according to **GP-5** using benzenesulfonyl chloride (0.38 mL, 3.0 mmol, 1.0 eq.), tetrabutylammonium hydroxide (1.0 M in MeOH, 3.3 mL, 3.3 mmol, 1.1 eq.) and MeOH (3 mL) to yield the title product as colourless crystals (0.679 g, 1.70 mmol, 57%).

^{1}H NMR (400 MHz, $CDCl_3$) δ 7.97 – 7.89 (m, 2H), 7.36 – 7.31 (m, 3H), 3.39 – 3.23 (m, 8H), 1.73 – 1.58 (m, 8H), 1.45 (q, *J* = 7.4 Hz, 8H), 1.01 (t, *J* = 7.3 Hz, 12H) ppm; ^{13}C NMR (101 MHz, $CDCl_3$) δ 146.8, 129.0, 127.8, 126.2, 58.8, 24.1, 19.7, 13.7 ppm.

Data is consistent with reported values.[11]

***tert*-Butyl ((mesitylsulfonyl)oxy)carbamate 7a**

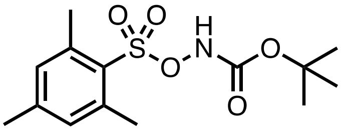

Prepared according to **GP-1** using 2,4,6-trimethylbenzenesulfonyl chloride (1.20 g, 5.5 mmol, 1.1 eq.), *tert*-butyl *N*-hydroxycarbamate (0.67 g, 5.0 mmol, 1.0 eq.), NEt_3 (0.75 mL, 5.5 mmol, 1.1 eq.) and Et_2O (40 mL). The crude product was purified by recrystallisation from Pet. Ether/Et_2O to yield the title product as white crystals (0.70 g, 2.2 mmol, 44%).

^{1}H NMR (400 MHz, $CDCl_3$) δ 7.59 (br s, 1H), 6.99 (s, 2H), 2.68 (s, 6H), 2.32 (s, 3H), 1.32 (s, 9H) ppm; ^{13}C NMR (101 MHz, $CDCl_3$) δ 154.1, 144.4, 142.0, 131.7, 128.5, 83.8, 27.7, 23.1, 21.1 ppm.

Data is consistent with reported values.[12]

tert-Butyl ((methylsulfonyl)oxy)carbamate 7b

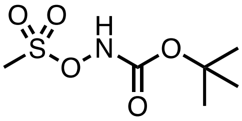

Prepared according to **GP-1** using mesyl chloride (0.86 mL, 11.0 mmol, 1.1 eq.), *tert*-butyl *N*-hydroxycarbamate (1.33 g, 10.0 mmol, 1.0 eq.), NEt_3 (1.5 mL, 11.0 mmol, 1.1 eq.) and Et_2O (80 mL). The crude product was purified by recrystallisation from Pet. Ether/Et_2O to yield the title product as colourless crystals (1.34 g, 6.4 mmol, 64%).

^{1}H NMR (400 MHz, $CDCl_3$) δ 7.92 (br s, 1H), 3.20 (s, 3H), 1.54 (s, 9H) ppm; ^{13}C NMR (101 MHz, $CDCl_3$) δ 154.6, 84.8, 36.4, 28.0 ppm.

Data is consistent with reported values.[13]

9. References

[1] J. Liu, K. Wu, T. Shen, Y. Liang, M. Zou, Y. Zhu, X. Li, X. Li, N. Jiao, *Chem. – A Eur. J.* **2017**, *23*, 563–567.

[2] X. Ma, I. R. Hazelden, T. Langer, R. H. Munday, J. F. Bower, *J. Am. Chem. Soc.* **2019**, *141*, 3356–3360.

[3] J. E. Gillespie, C. Morrill, R. J. Phipps, *J. Am. Chem. Soc.* **2021**, *143*, 9355–9360.

[4] R. E. Moreira, G. S. Sinclair, D. J. Schipper, *Can. J. Chem.* **2019**, *97*, 360–365.

[5] H.-Z. Li, L.-W. Xiao, H.-Y. Li, K.-F. Wang, X. Li, *J. Chem. Res.* **2003**, *2003*, 493–494.

[6] A. L. J. Beckwith, G. F. Meijs, *J. Org. Chem.* **1987**, *52*, 1922–1930.

[7] J. B. Gordon, J. P. McGale, J. R. Prendergast, Z. Shirani-Sarmazeh, M. A. Siegler, G. N. L. Jameson, D. P. Goldberg, *J. Am. Chem. Soc.* **2018**, *140*, 14807–14822.

[8] N. Radhoff, A. Studer, *Angew. Chem. Int. Ed.* **2021**, *60*, 3561–3565.

[9] M. R. Jafari, J. Lakusta, R. J. Lundgren, R. Derda, *Bioconjug. Chem.* **2016**, *27*, 509–514.

[10] J. Zhao, S. Niu, X. Jiang, Y. Jiang, X. Zhang, T. Sun, D. Ma, *J. Org. Chem.* **2018**, *83*, 6589–6599.

[11] P. G. M. Wuts, K. E. Wilson, *Synthesis (Stuttg).* **1998**, *11*, 1593–1595.

[12] Masruri, A. C. Willis, M. D. McLeod, *J. Org. Chem.* **2012**, *77*, 8480–8491.

[13] E. M. D’Amato, J. Börgel, T. Ritter, *Chem. Sci.* **2019**, *10*, 2424–2428.

10.NMR Spectra

tert-Butyl ((phenylsulfonyl)oxy)carbamate 1a

400 MHz, $CDCl_3$

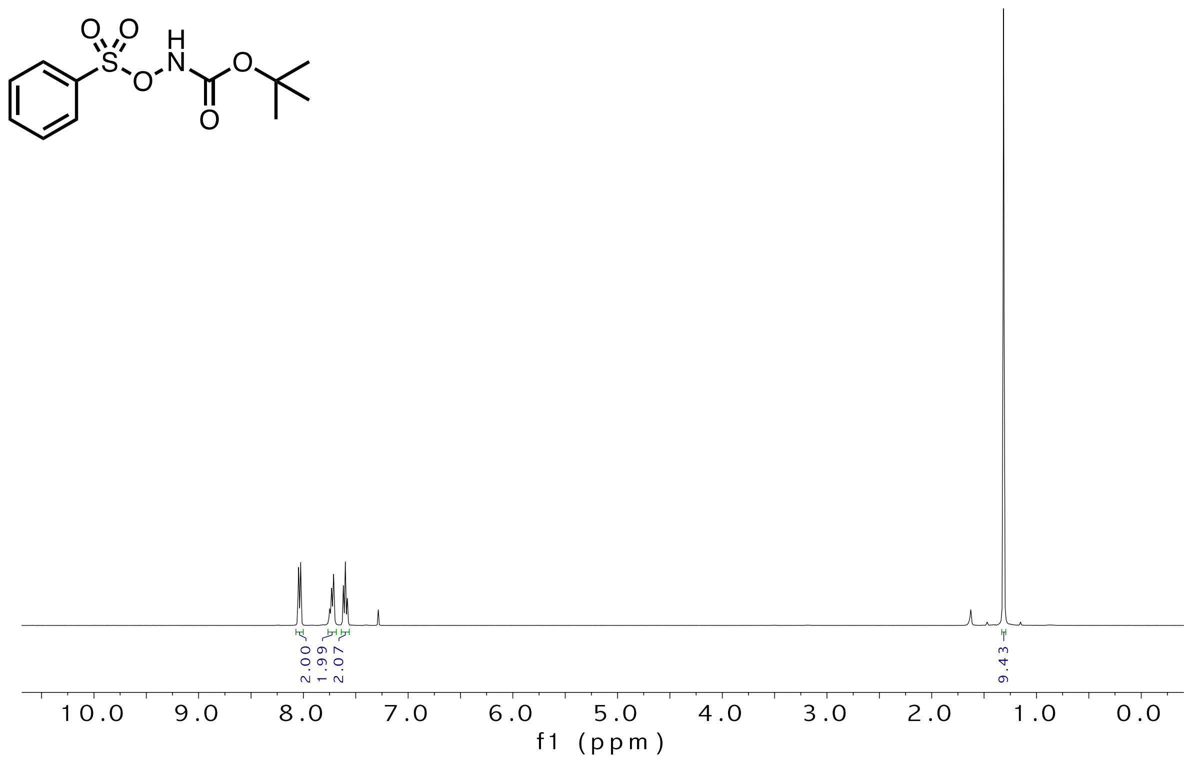

101 MHz, $CDCl_3$

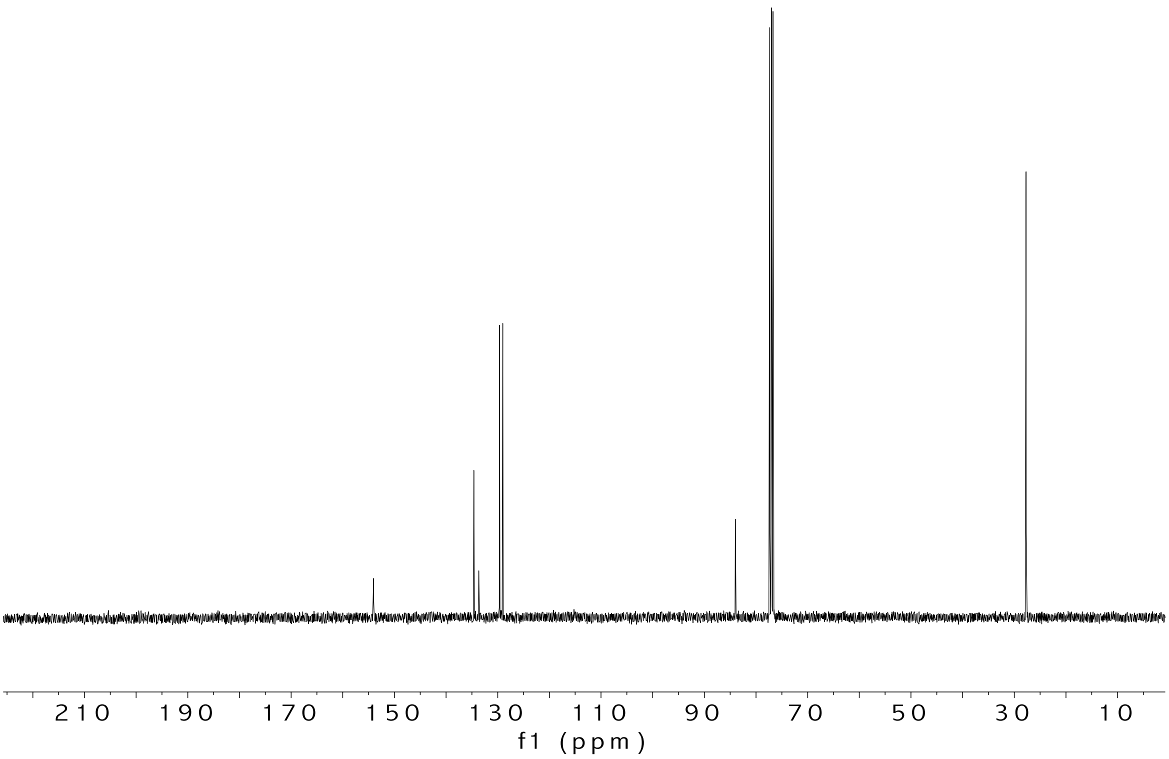

***tert*-Butyl (tosyloxy)carbamate 1b**

400 MHz, $CDCl_3$

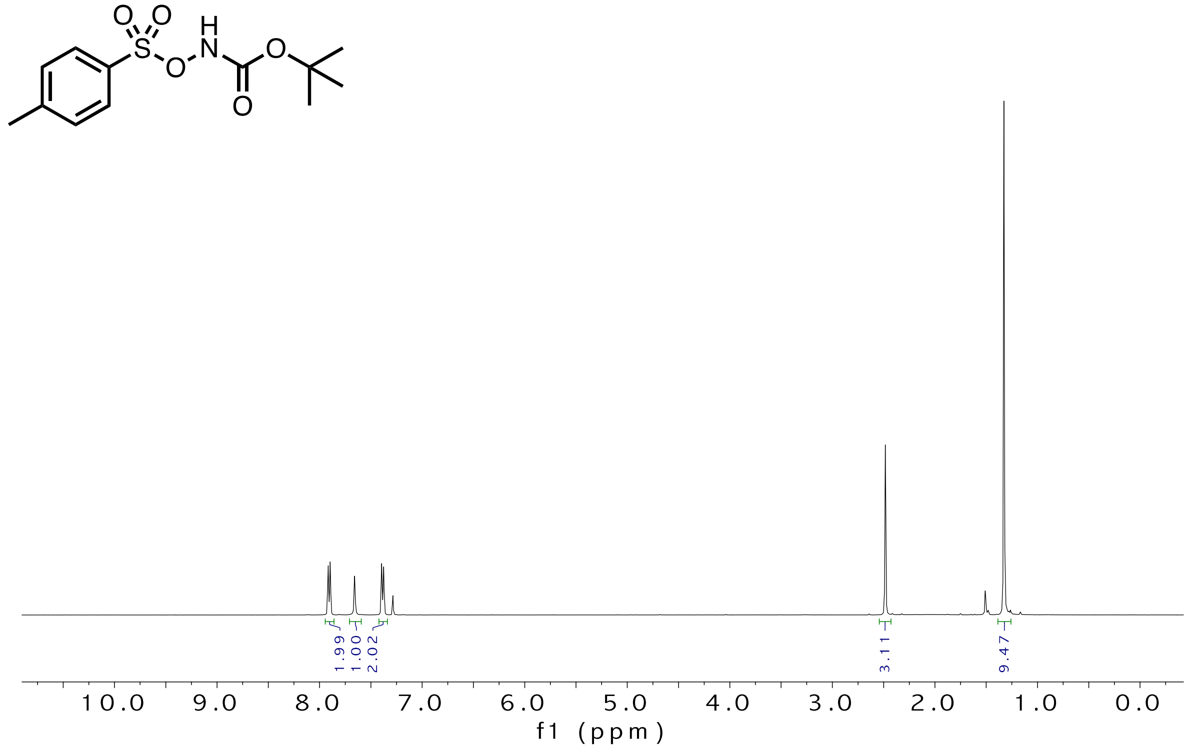

101 MHz, $CDCl_3$

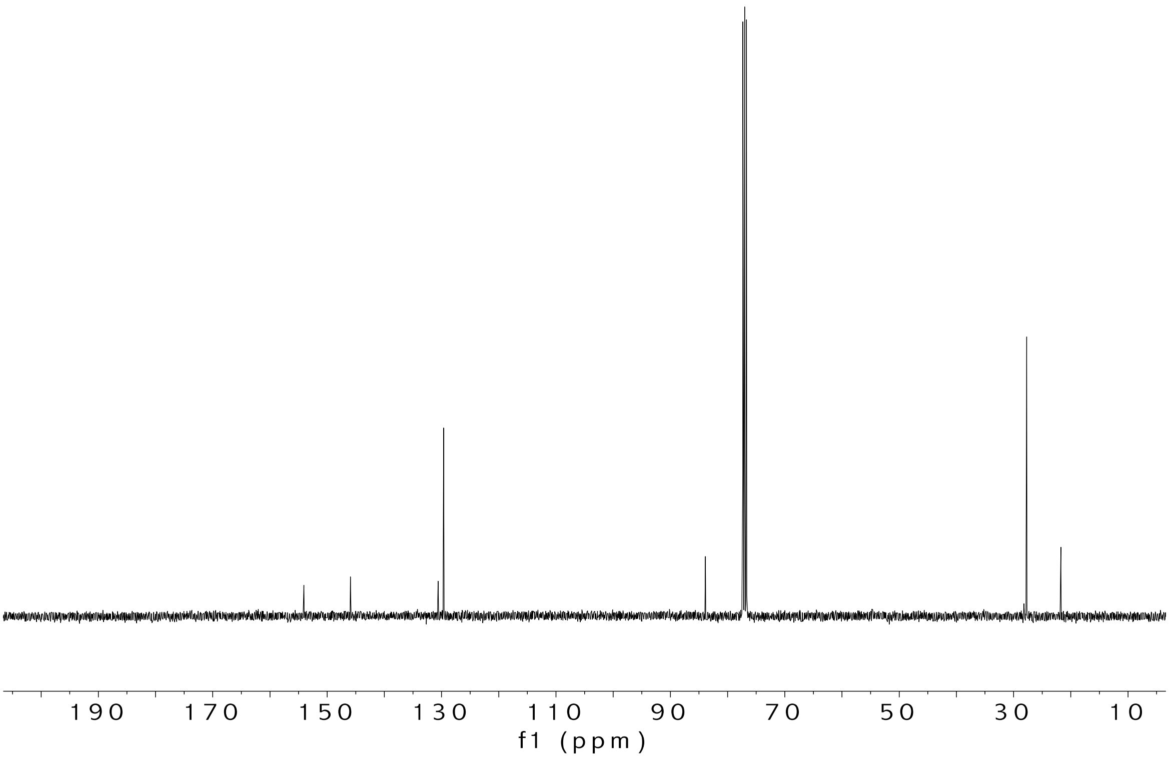

tert-Butyl((o-tolylsulfonyl)oxy)carbamate 1c

400 MHz, CDCl$_3$

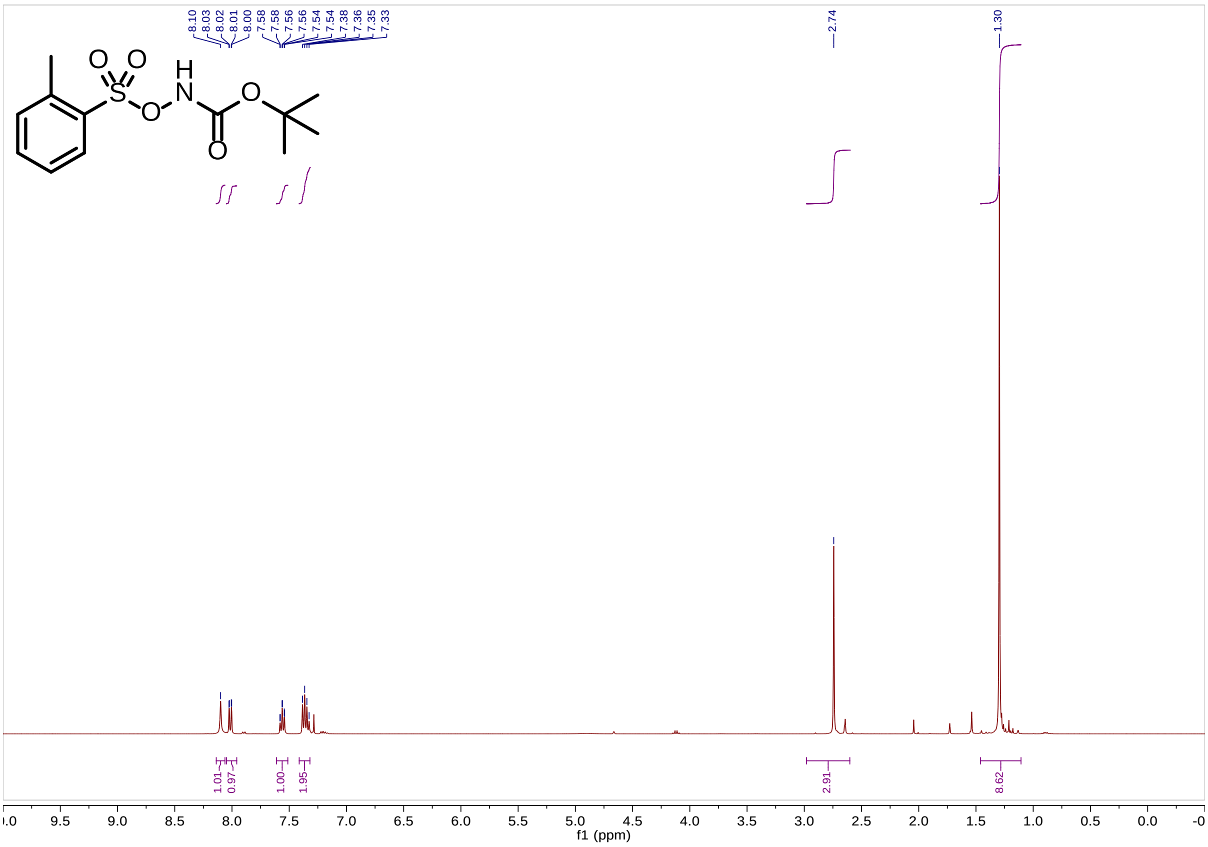

101 MHz, CDCl$_3$

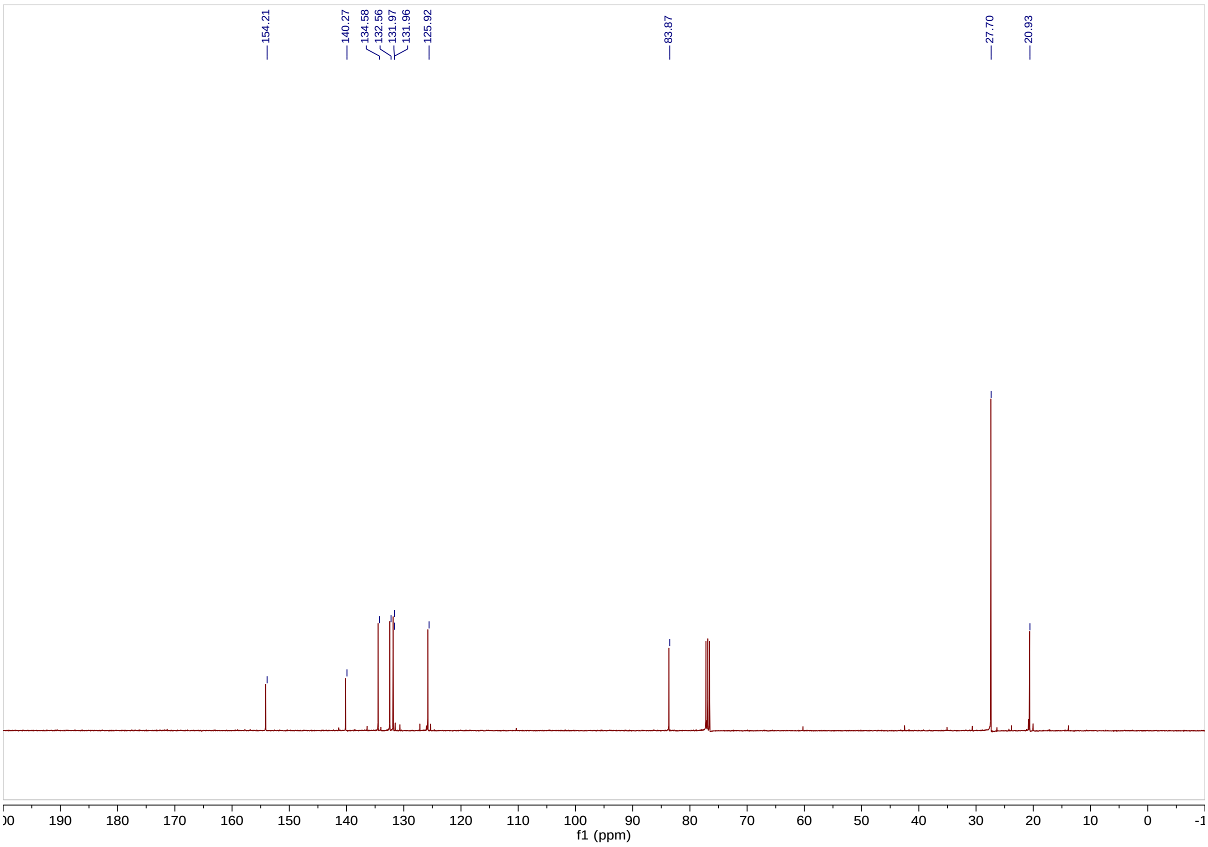

tert-Butyl ((_m_-tolylsulfonyl)oxy)carbamate 1d

400 MHz, $CDCl_3$

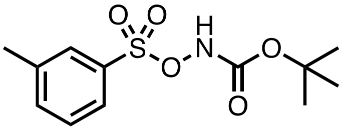

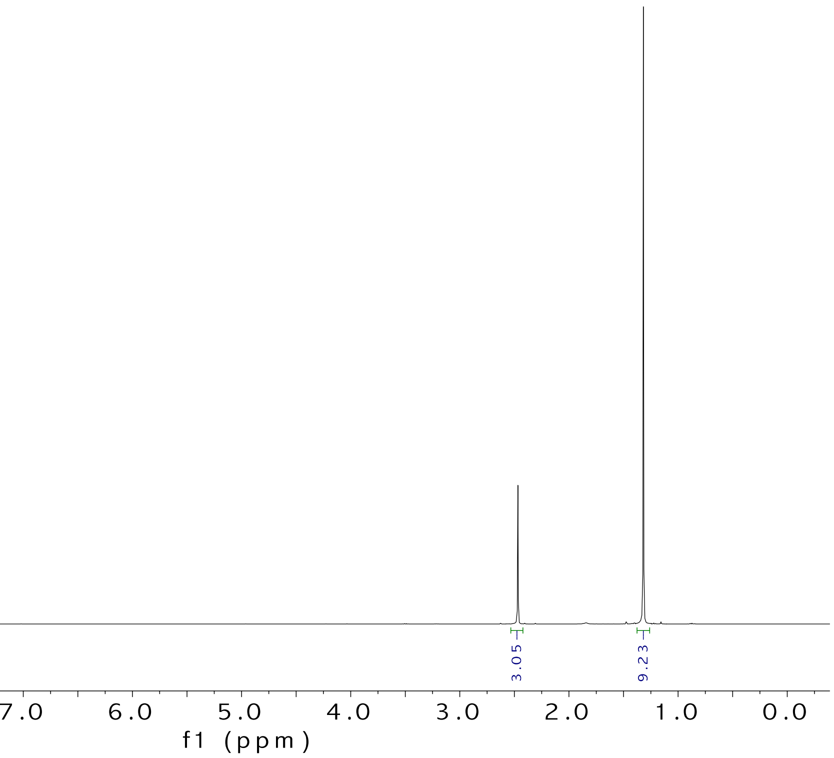

101 MHz, $CDCl_3$

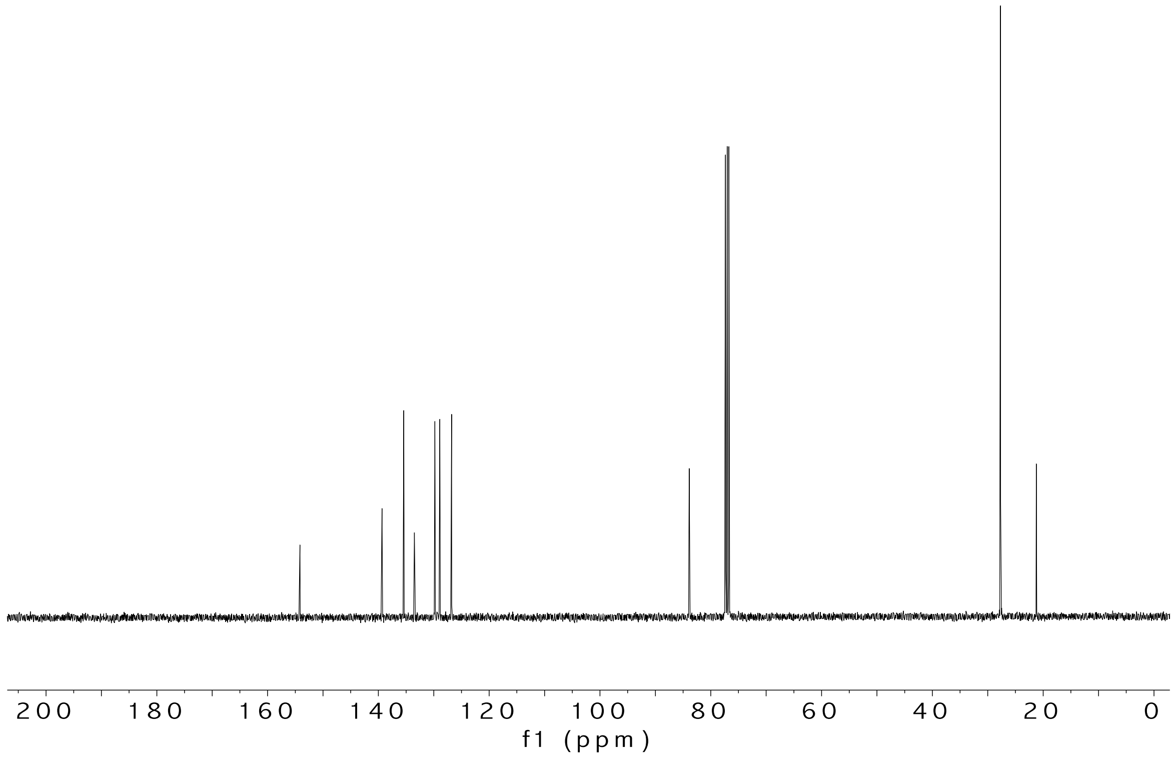

tert-Butyl (((3,4-dimethylphenyl)sulfonyl)oxy)carbamate 1e

400 MHz, $CDCl_3$

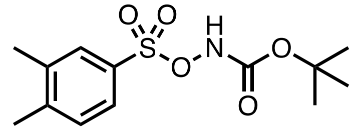

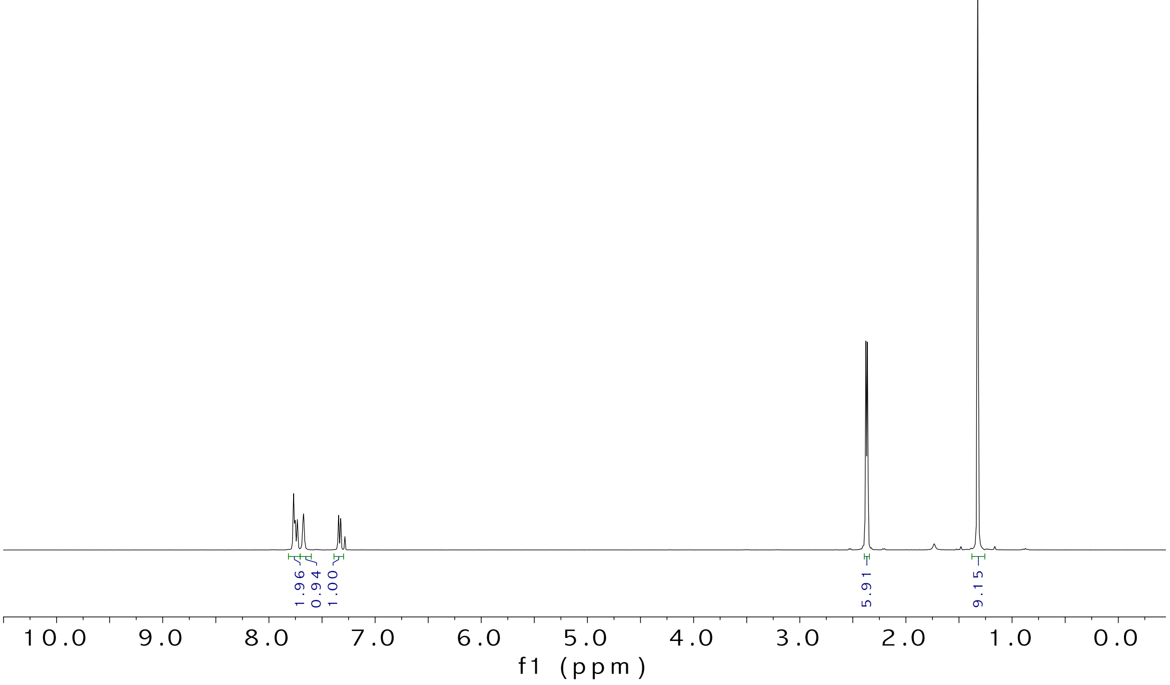

101 MHz, $CDCl_3$

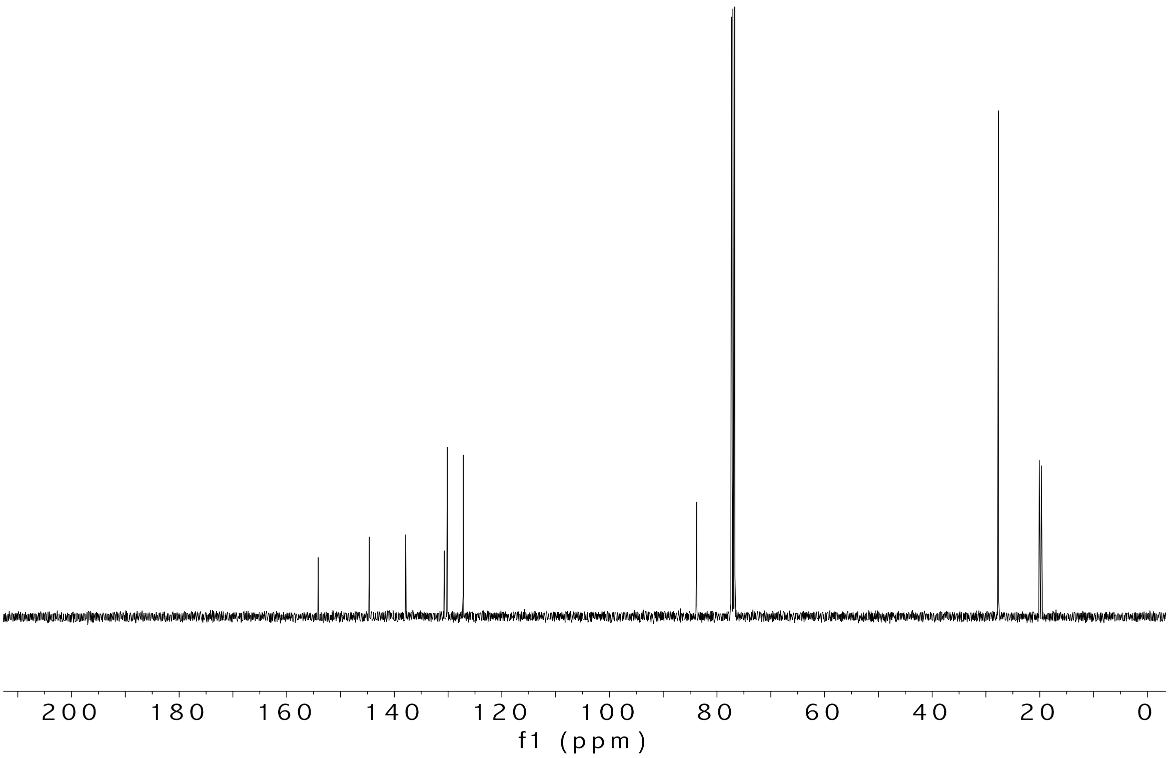

tert-Butyl (((3,5-dimethylphenyl)sulfonyl)oxy)carbamate 1f

400 MHz, $CDCl_3$

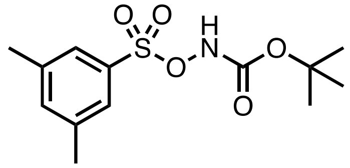

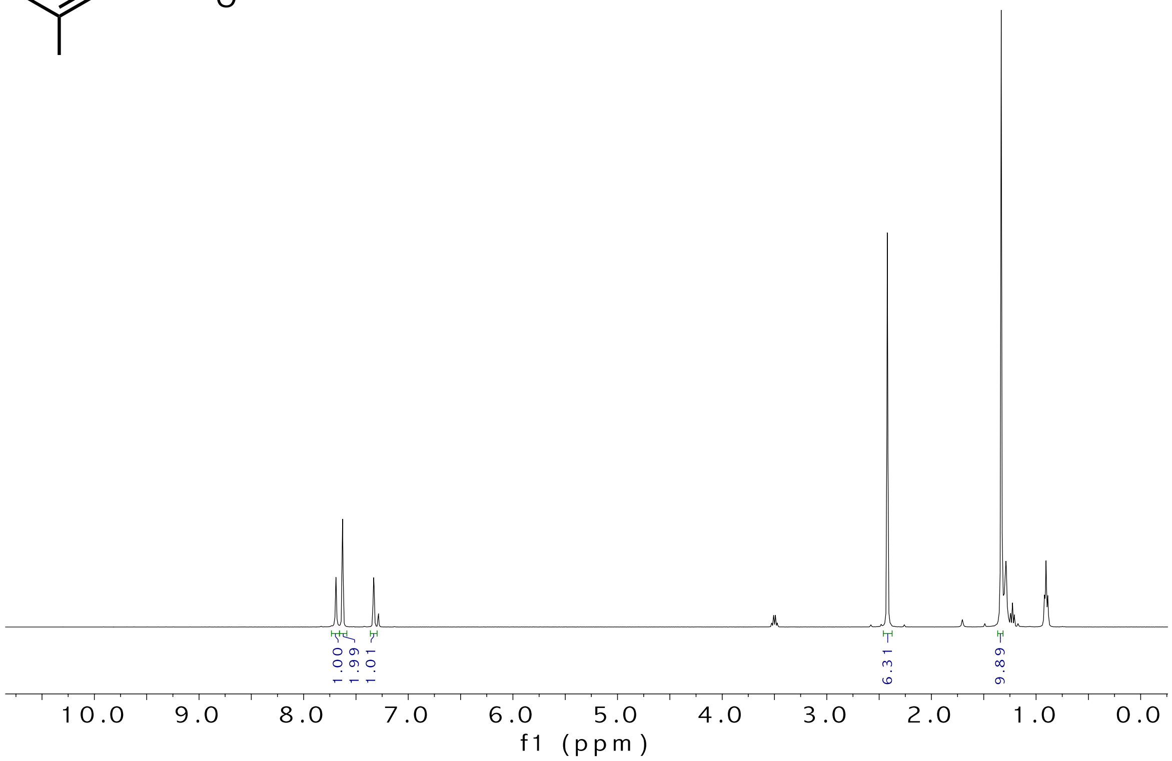

101 MHz, $CDCl_3$

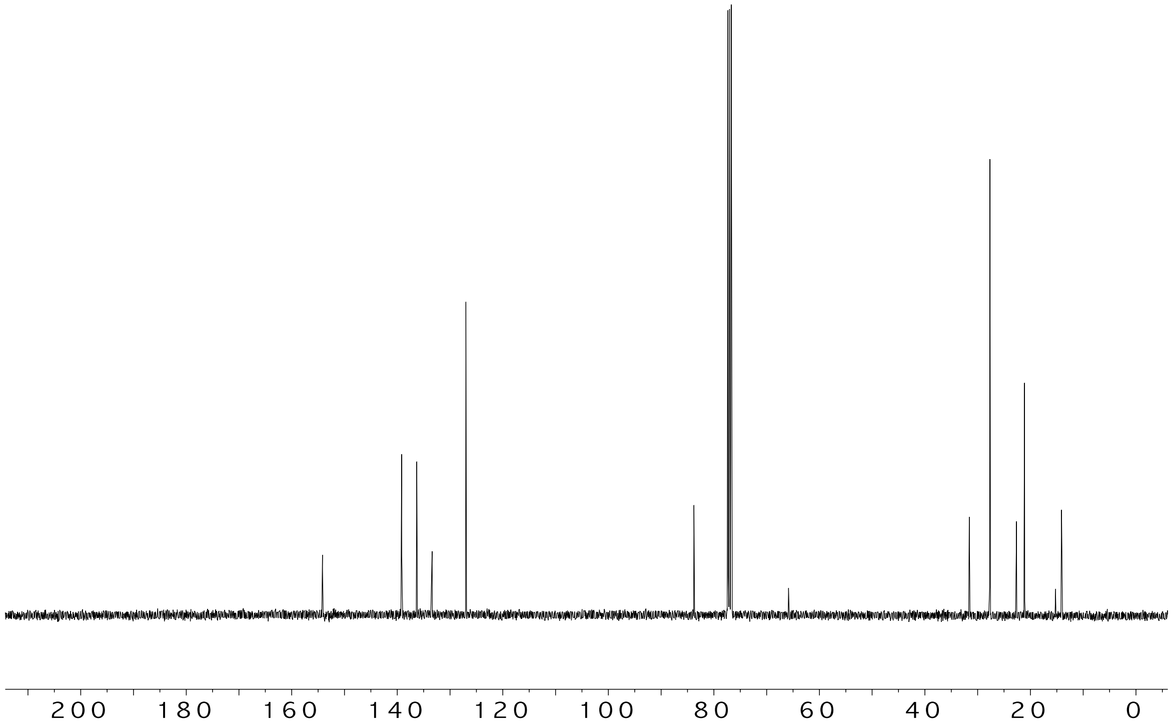

***tert*-Butyl (((4-butylphenyl)sulfonyl)oxy)carbamate 1g**

500 MHz, $CDCl_3$

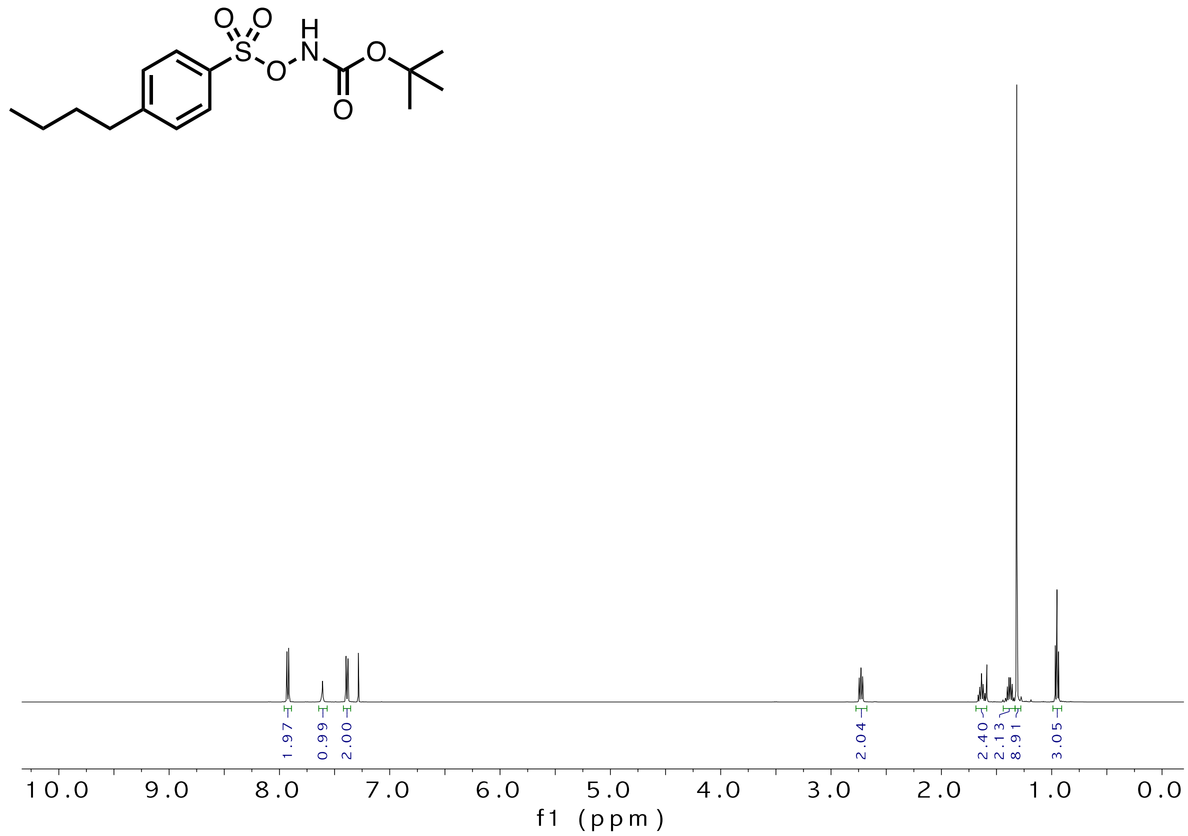

126 MHz, $CDCl_3$

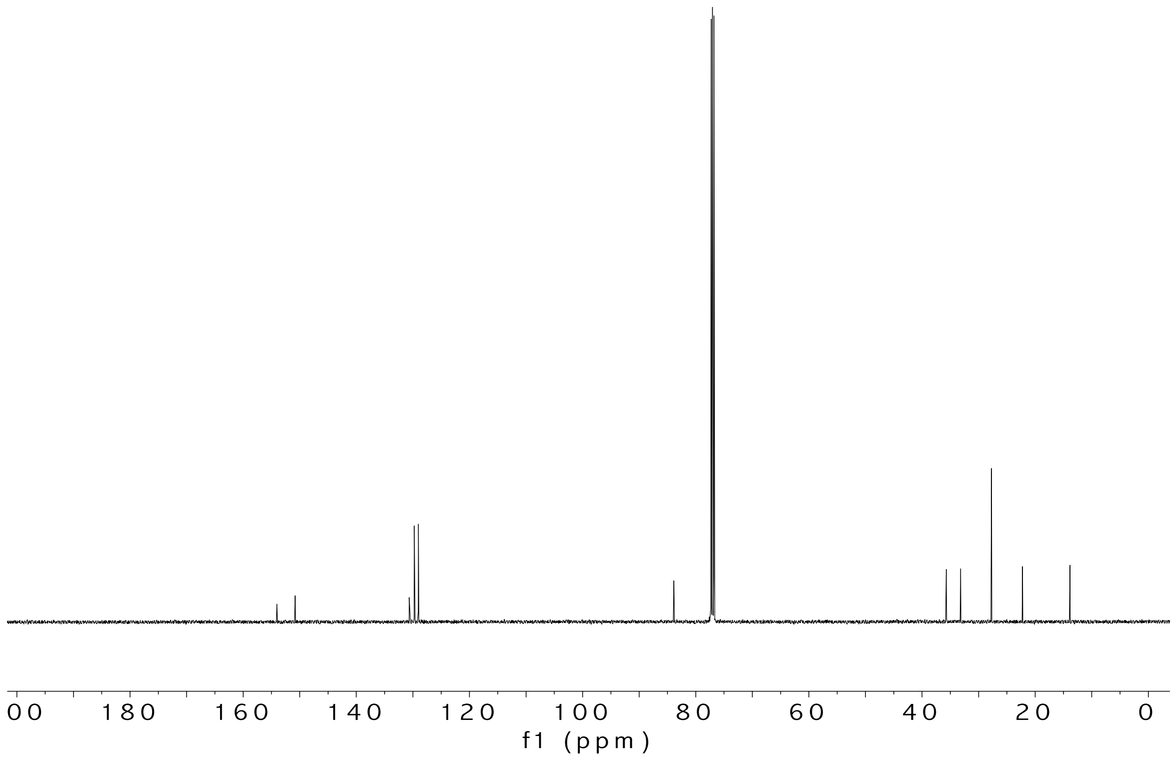

tert-Butyl (((4-(_tert_-butyl)phenyl)sulfonyl)oxy)carbamate 1h

400 MHz, $CDCl_3$

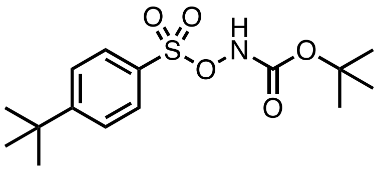

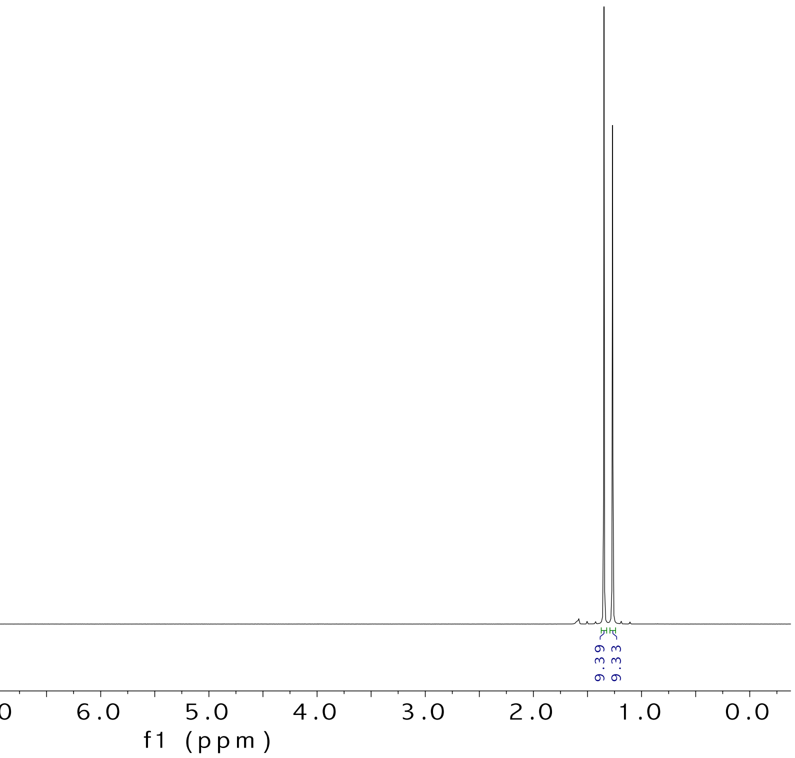

101 MHz, $CDCl_3$

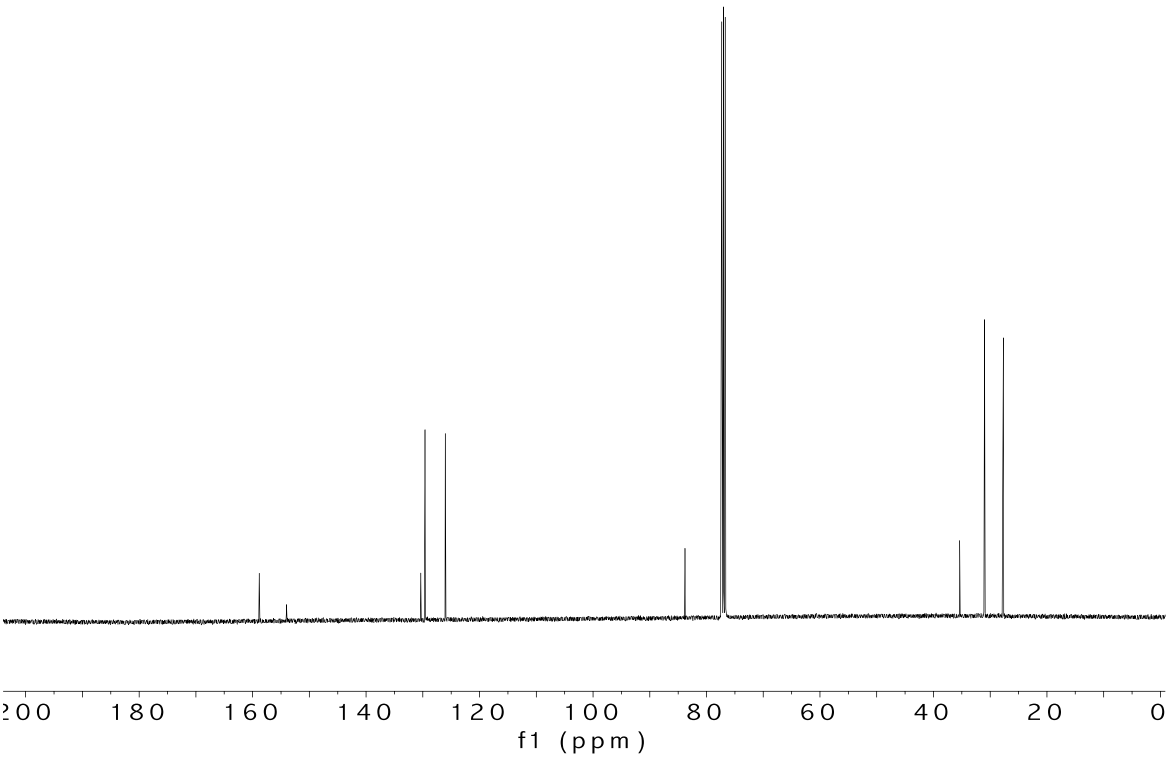

tert-Butyl (((3-fluoro-4-methylphenyl)sulfonyl)oxy)carbamate 1i

400 MHz, $CDCl_3$

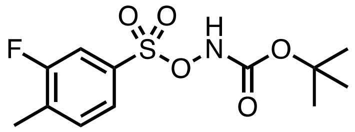

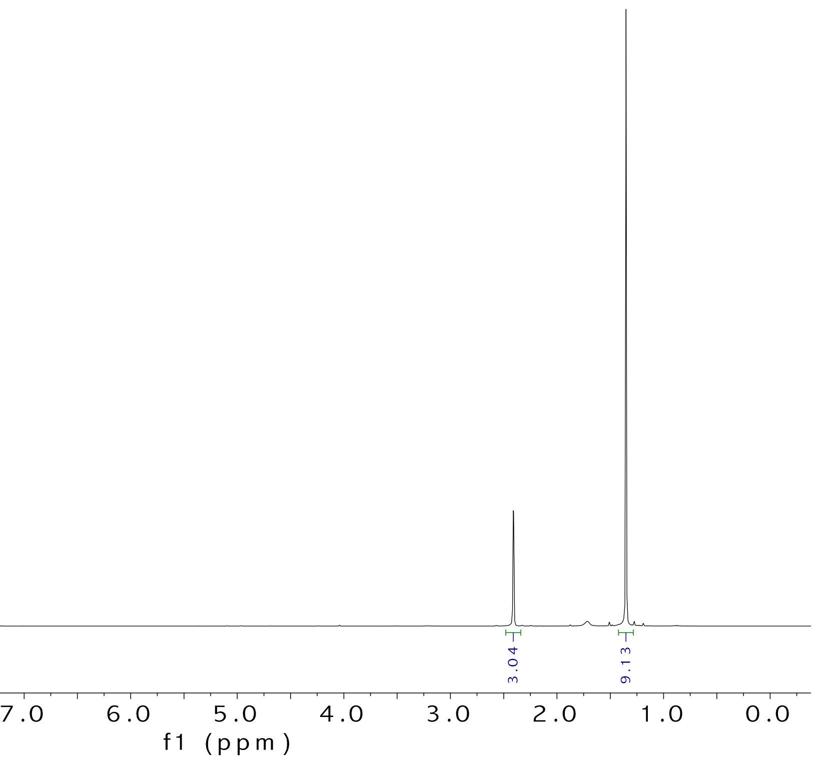

101 MHz, $CDCl_3$

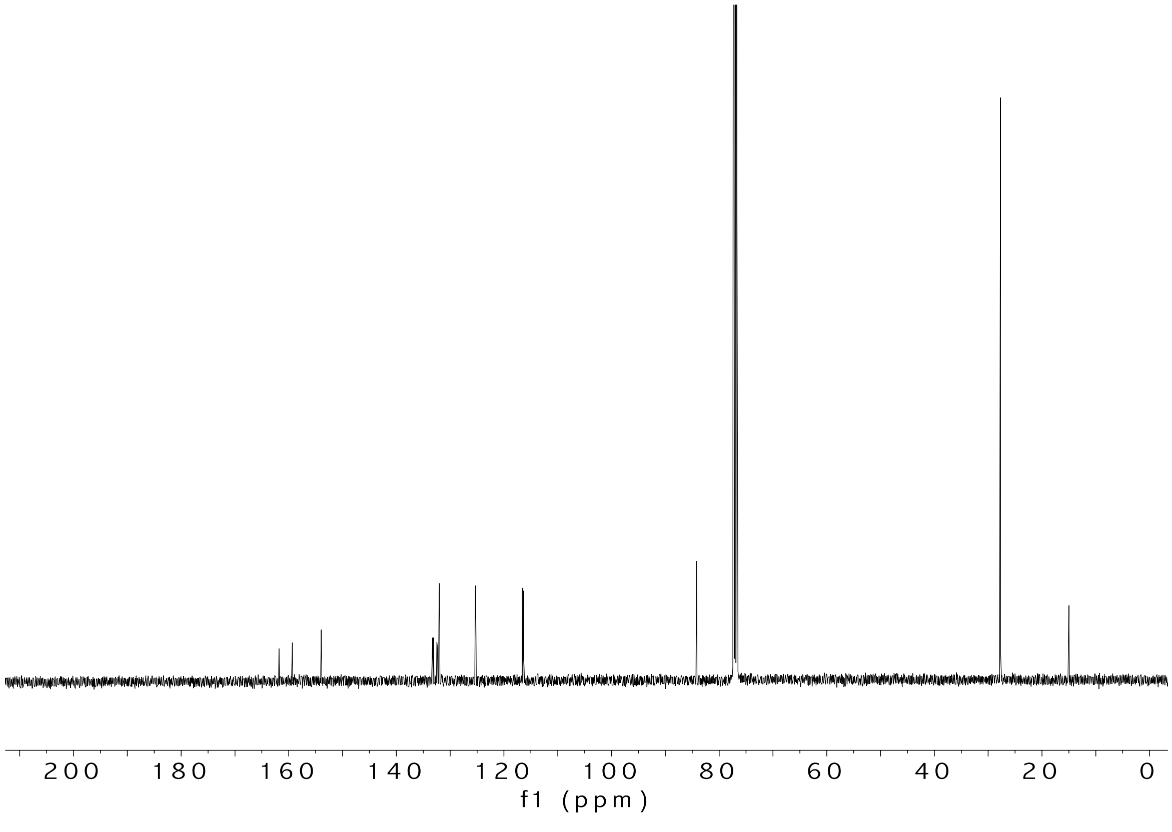

376 MHz, $CDCl_3$

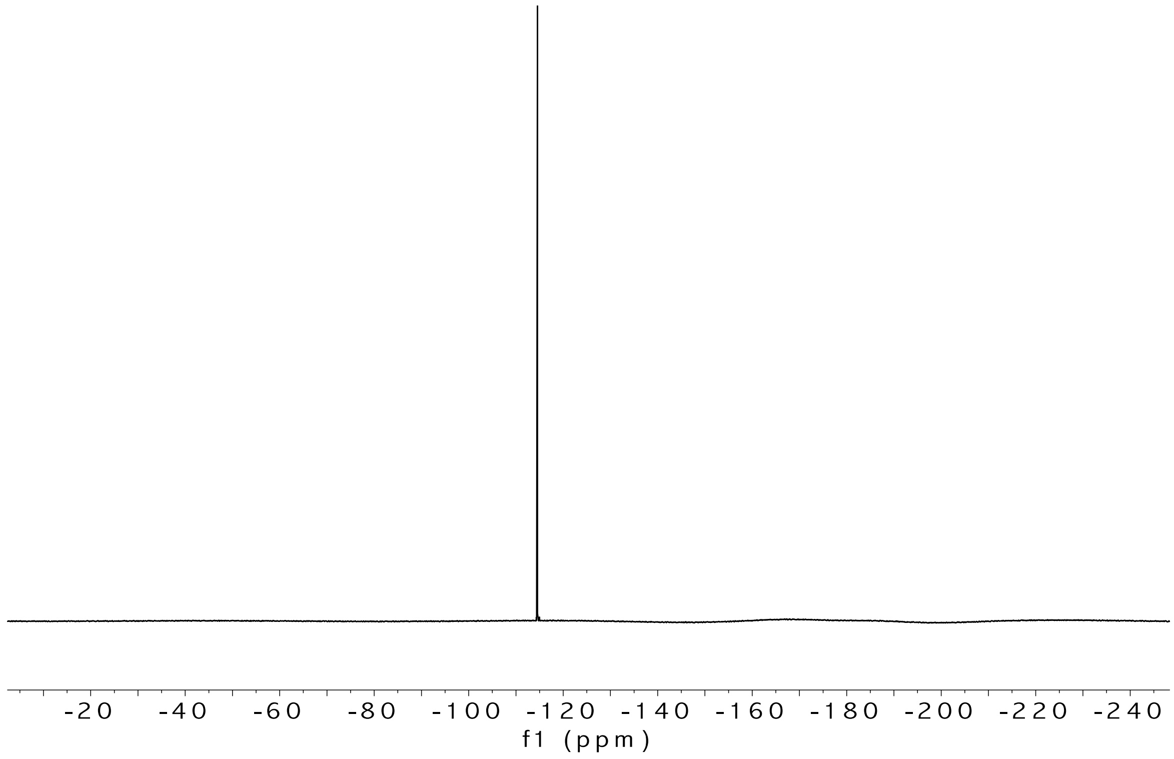

***tert*-Butyl (((3-fluorophenyl)sulfonyl)oxy)carbamate 1j**

400 MHz, $CDCl_3$

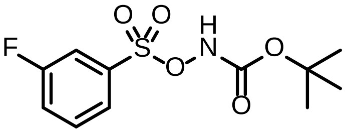

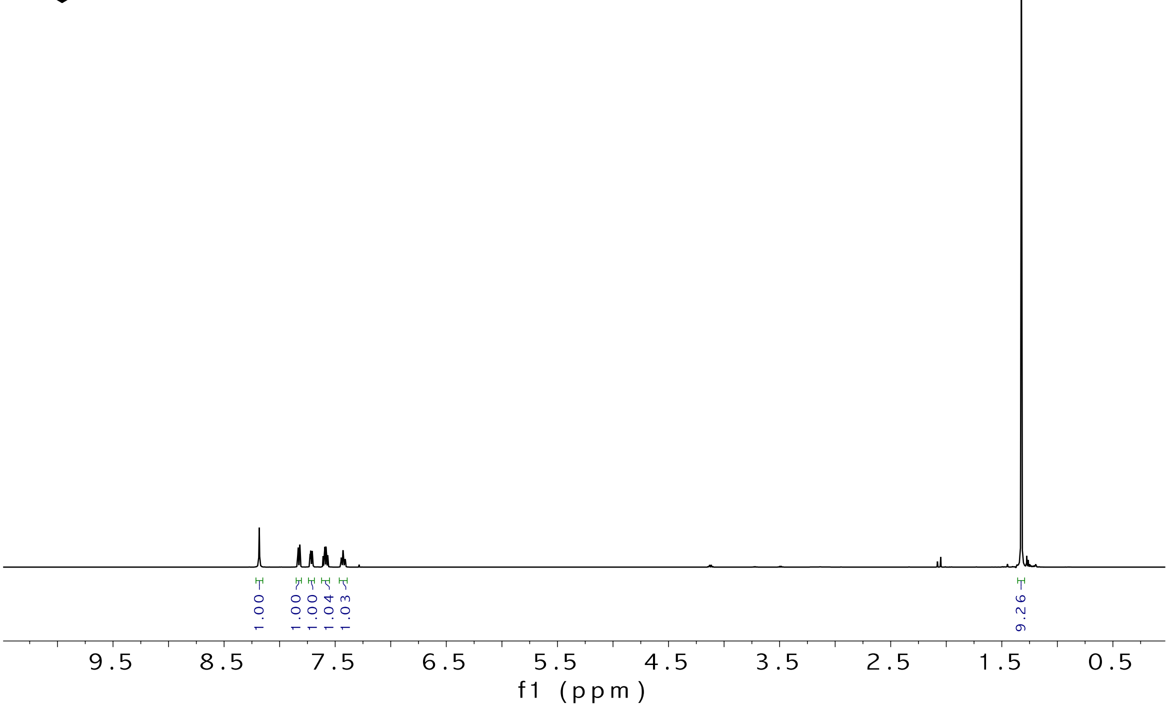

101 MHz, $CDCl_3$

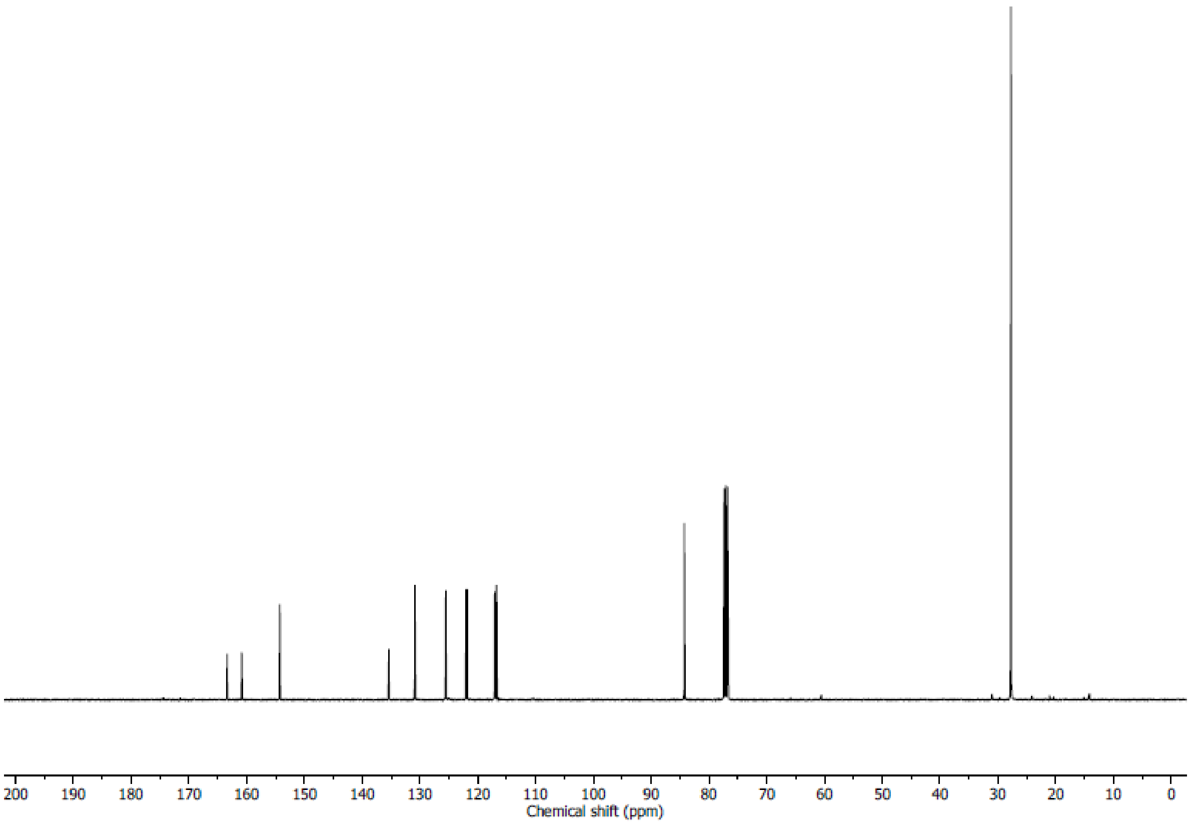

471 MHz, $CDCl_3$

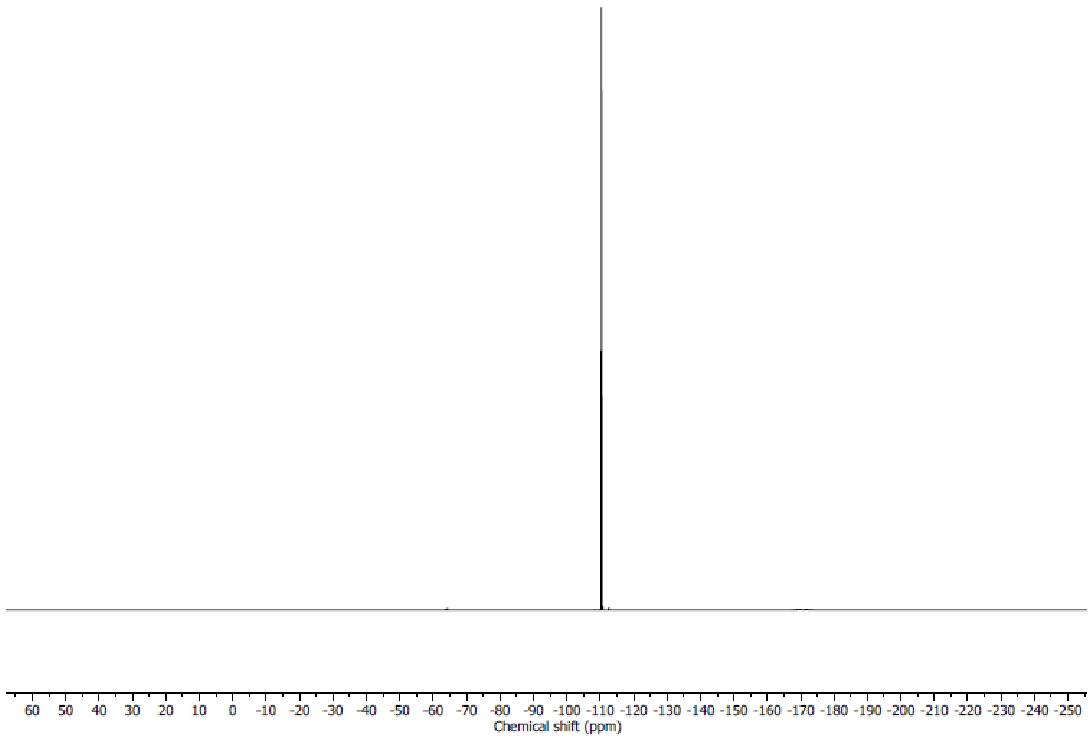

tert-Butyl (((3,4,5-trifluorophenyl)sulfonyl)oxy)carbamate 1k

400 MHz, $CDCl_3$

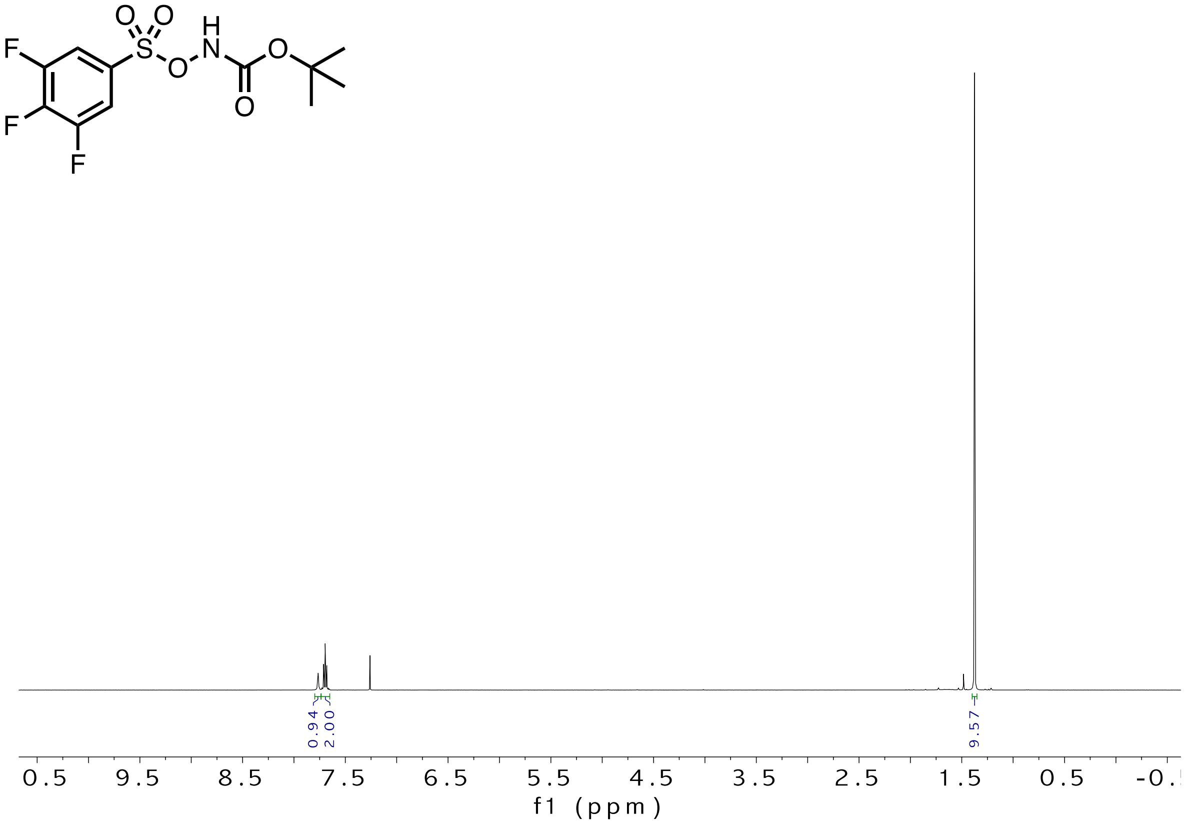

101 MHz, $CDCl_3$

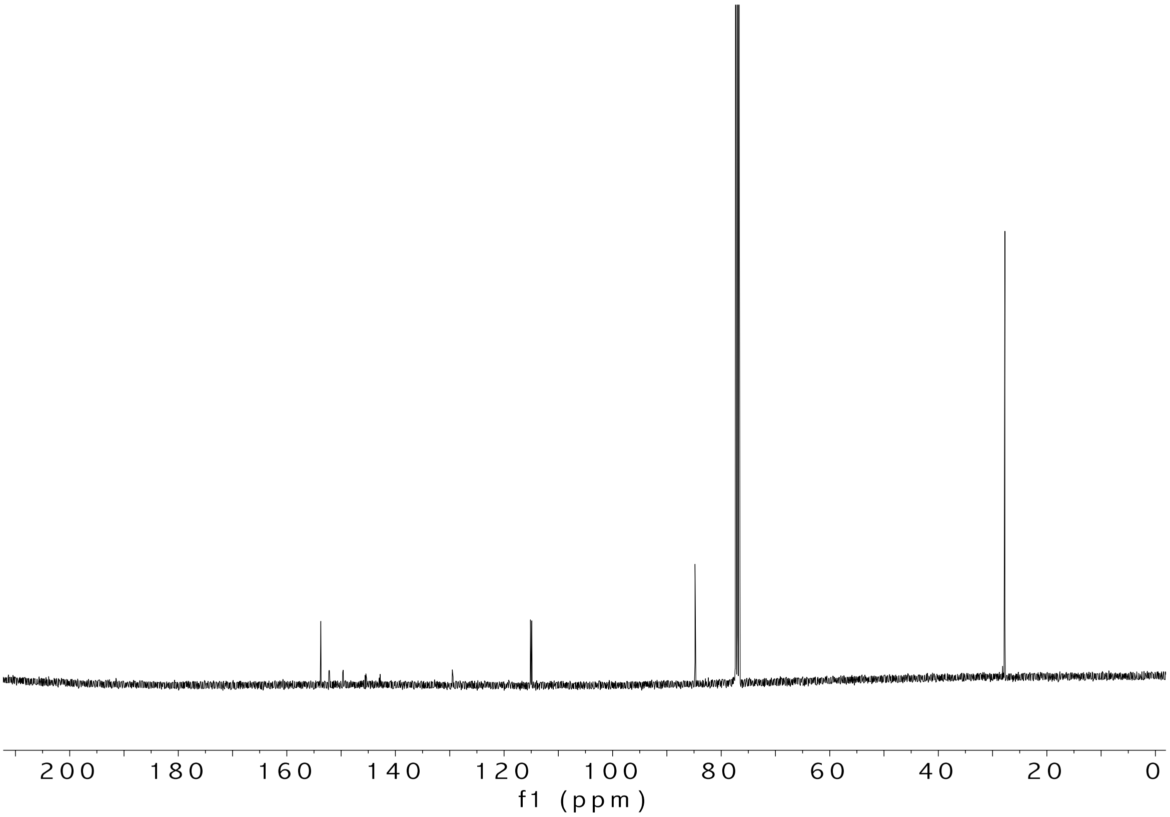

376 MHz, $CDCl_3$

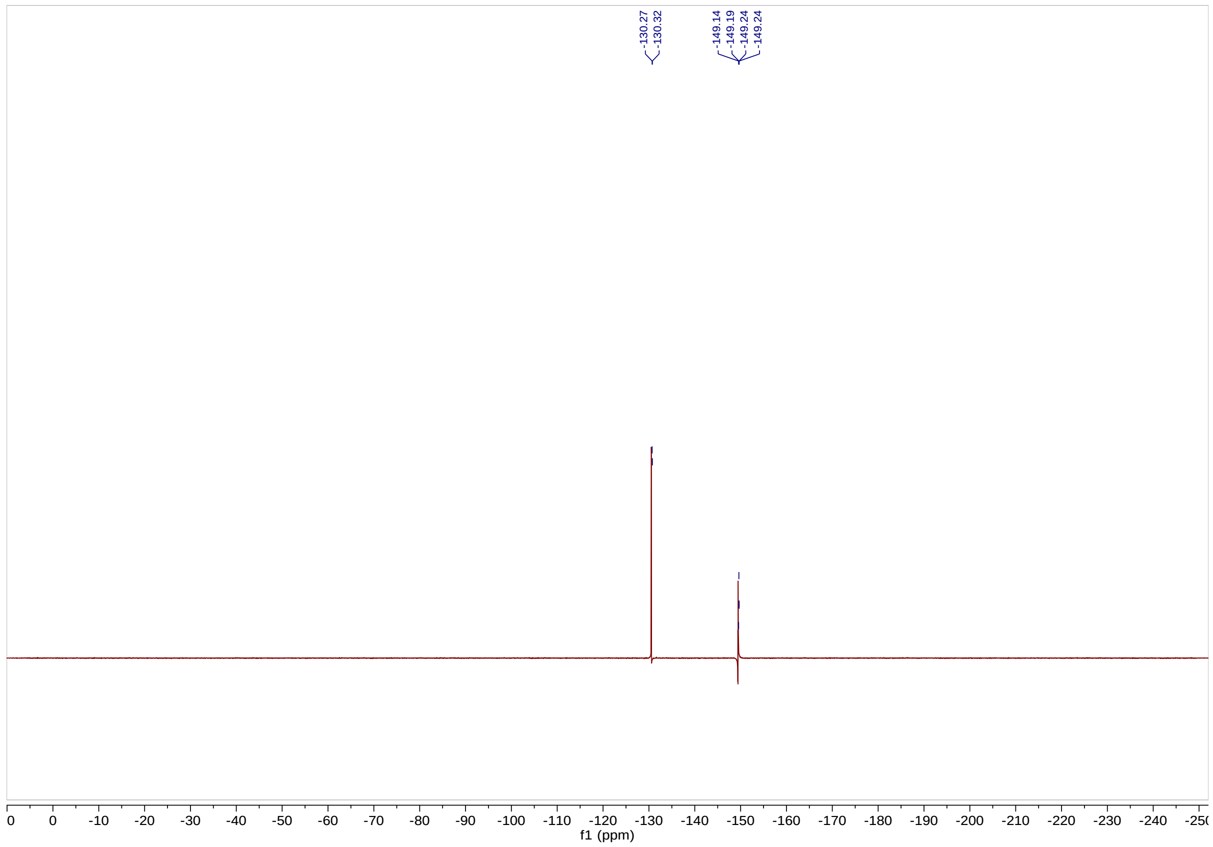

tert-Butyl (((3-chloro-4-fluorophenyl)sulfonyl)oxy)carbamate 1l

400 MHz, $CDCl_3$

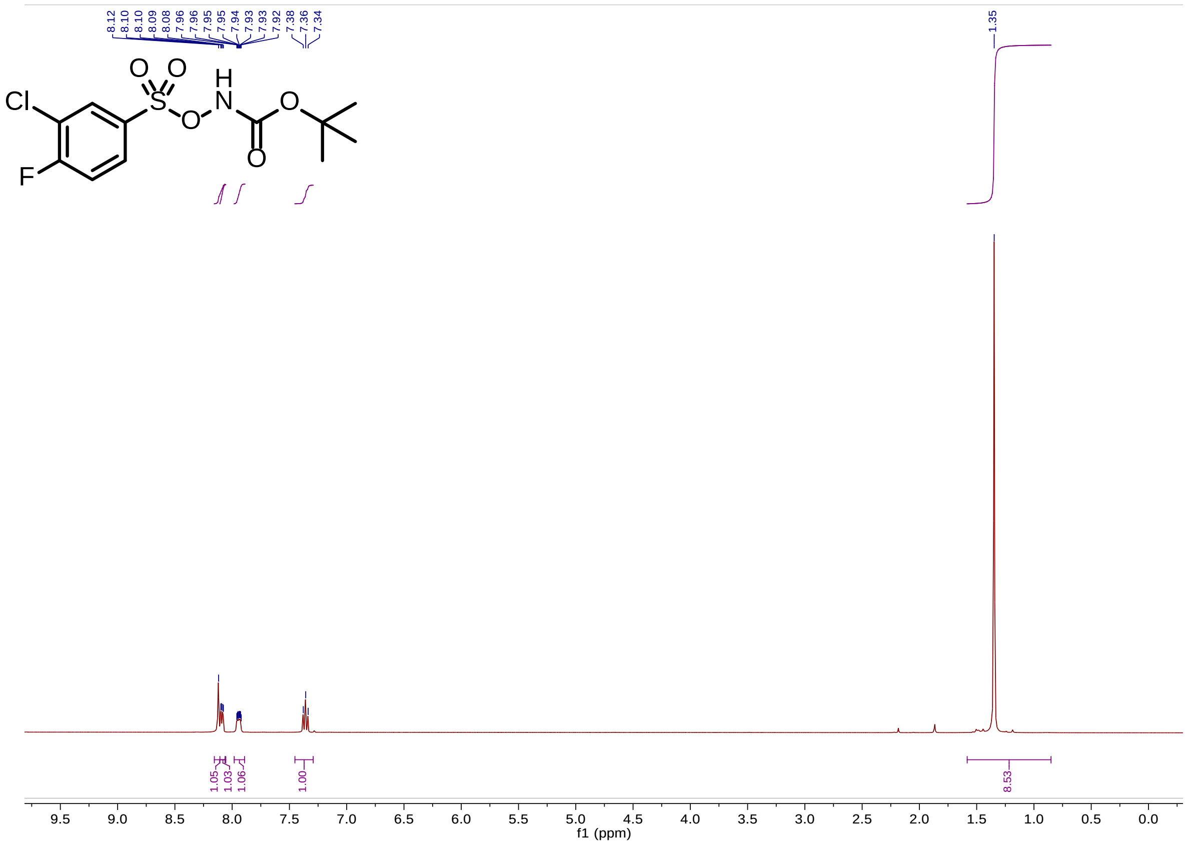

101 MHz, $CDCl_3$

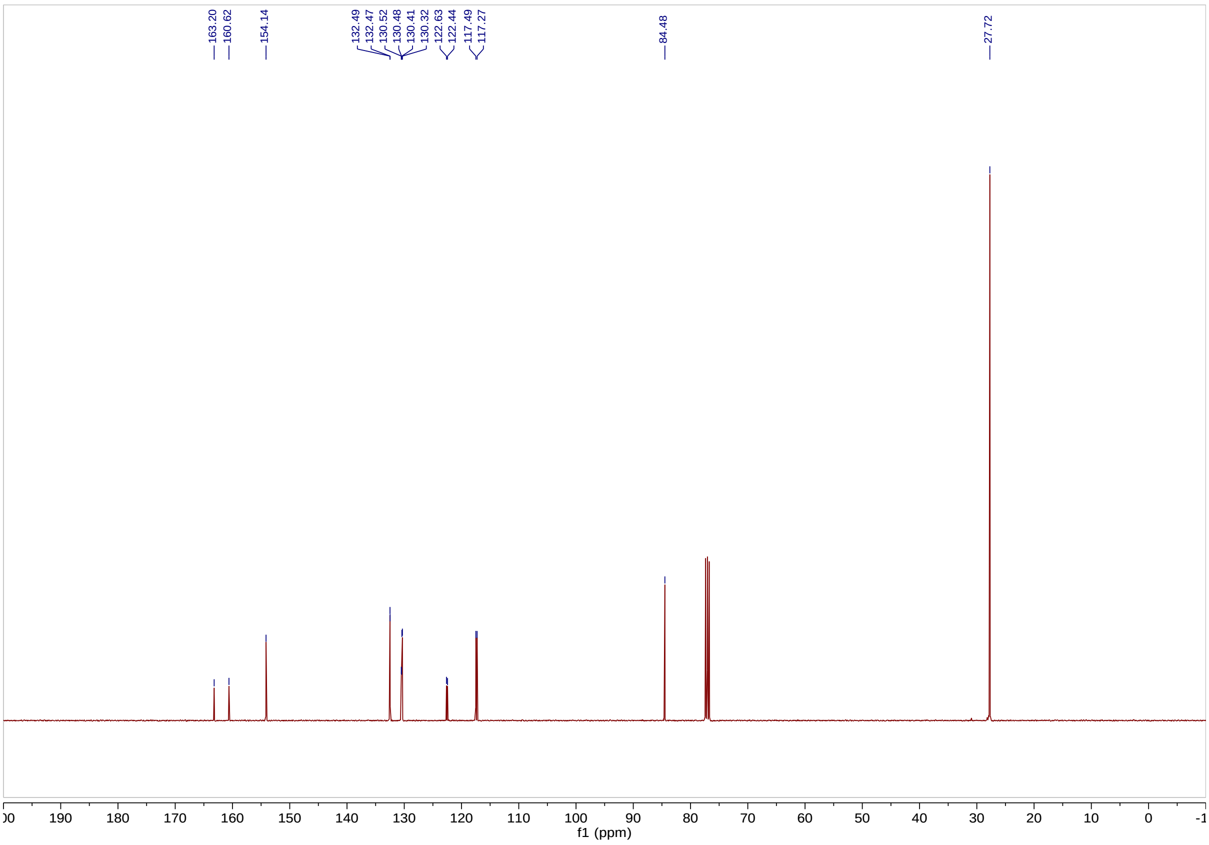

471 MHz, $CDCl_3$

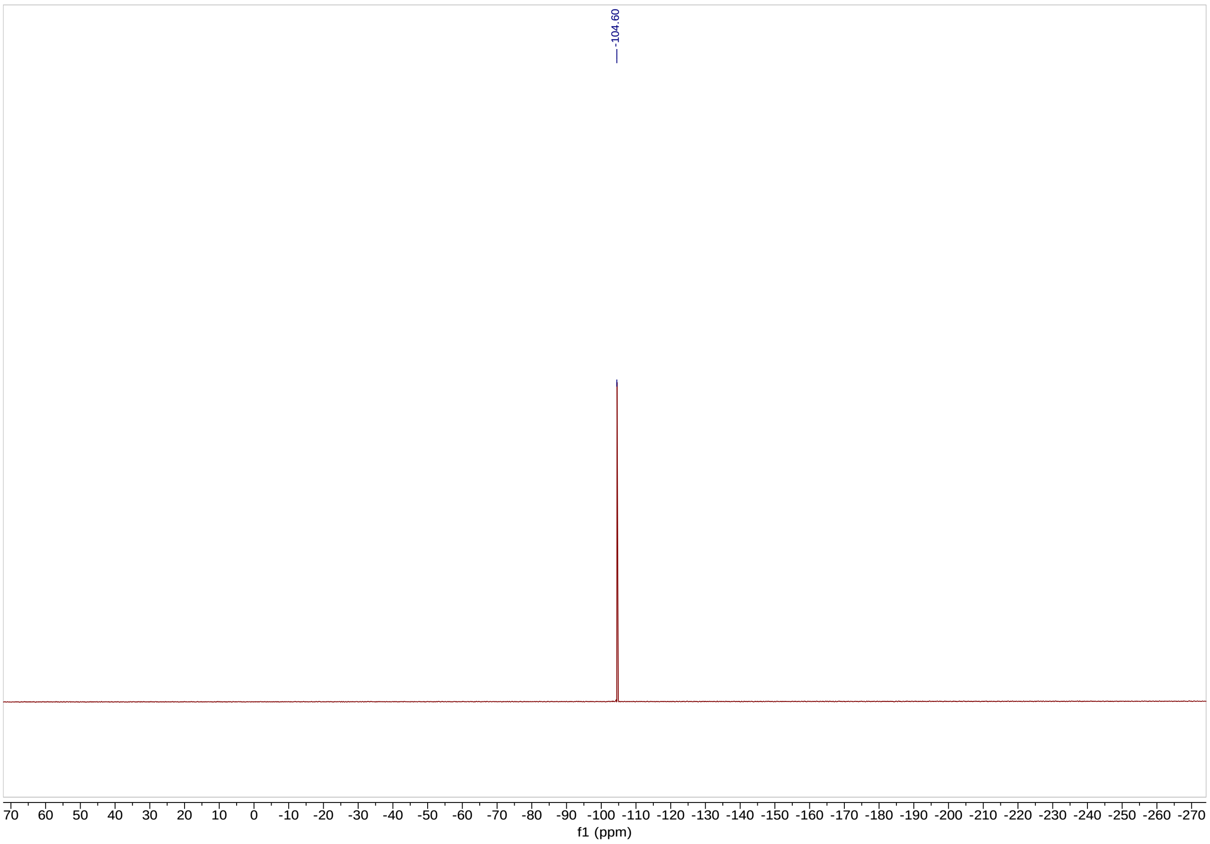

***tert*-Butyl (((3-chlorophenyl)sulfonyl)oxy)carbamate 1m**

400 MHz, $CDCl_3$

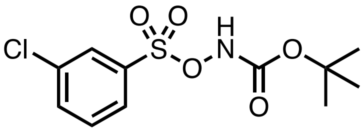

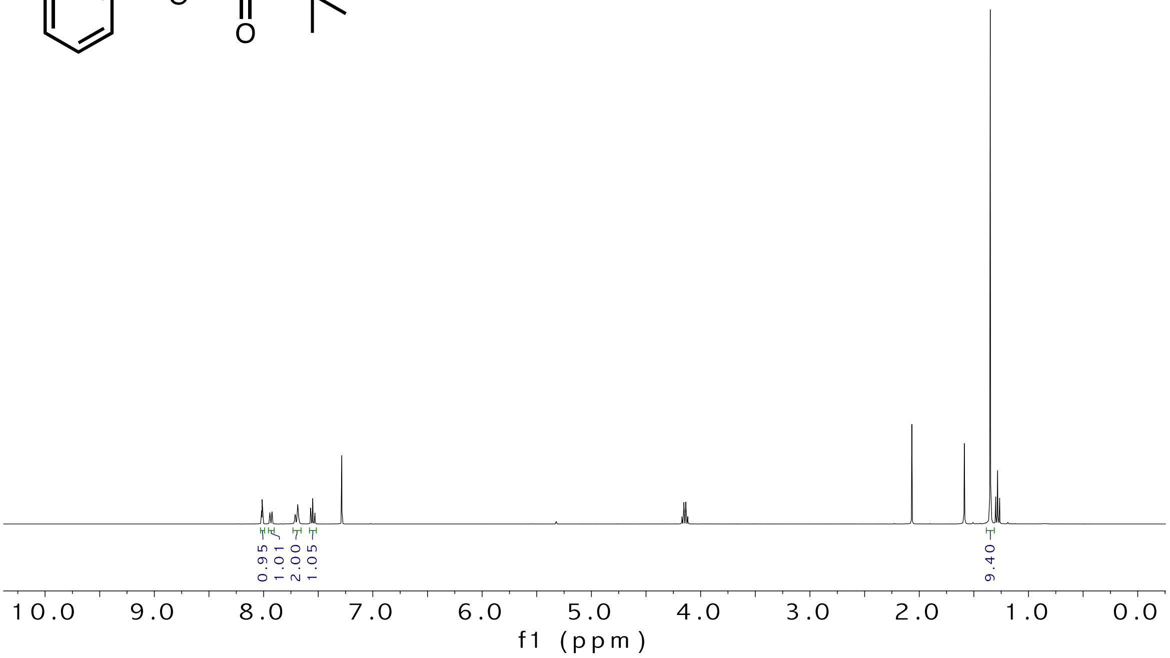

101 MHz, $CDCl_3$

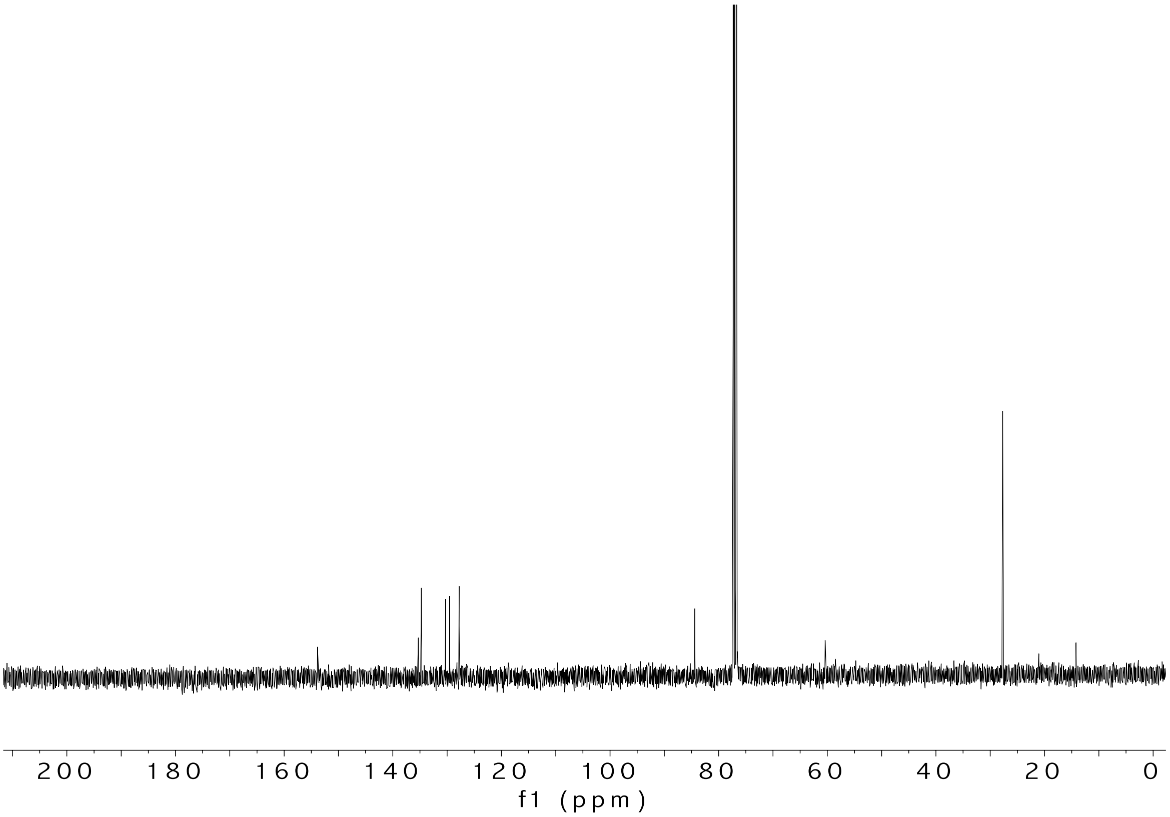

***tert*-Butyl (((2-chlorophenyl)sulfonyl)oxy)carbamate 1n**

400 MHz, $CDCl_3$

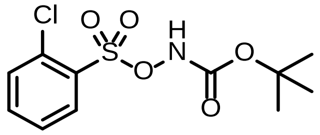

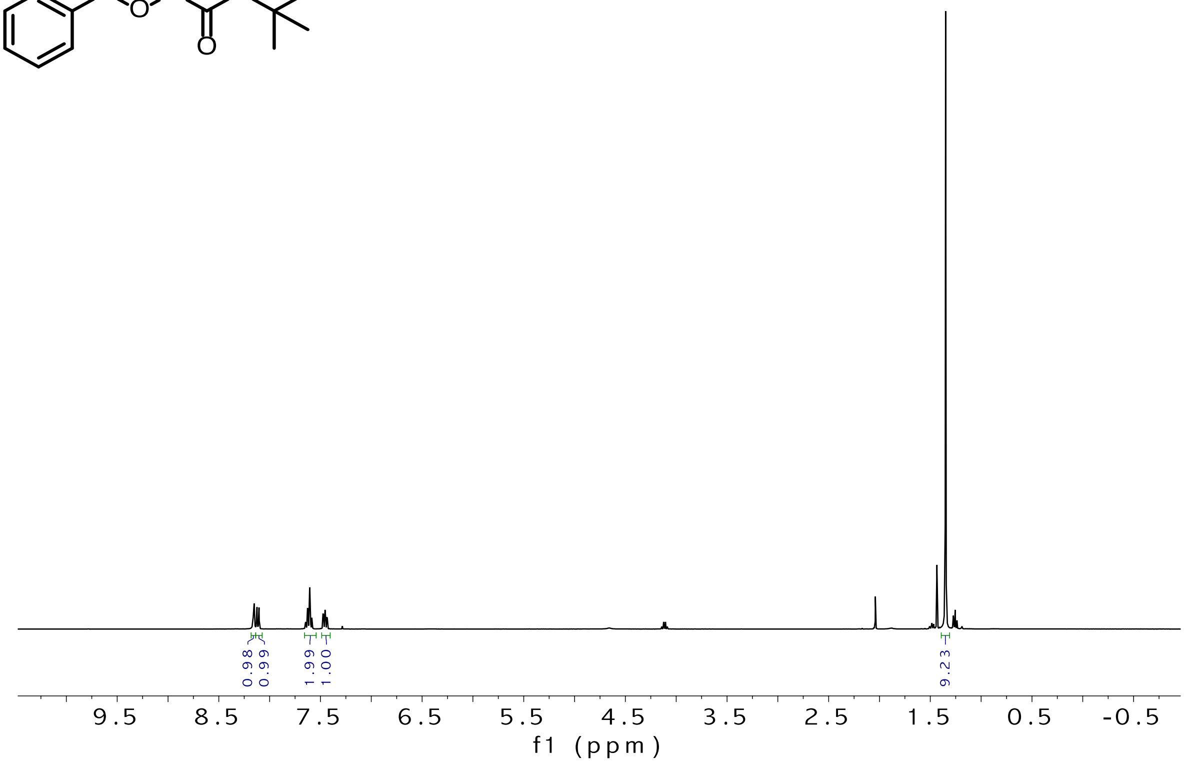

101 MHz, $CDCl_3$

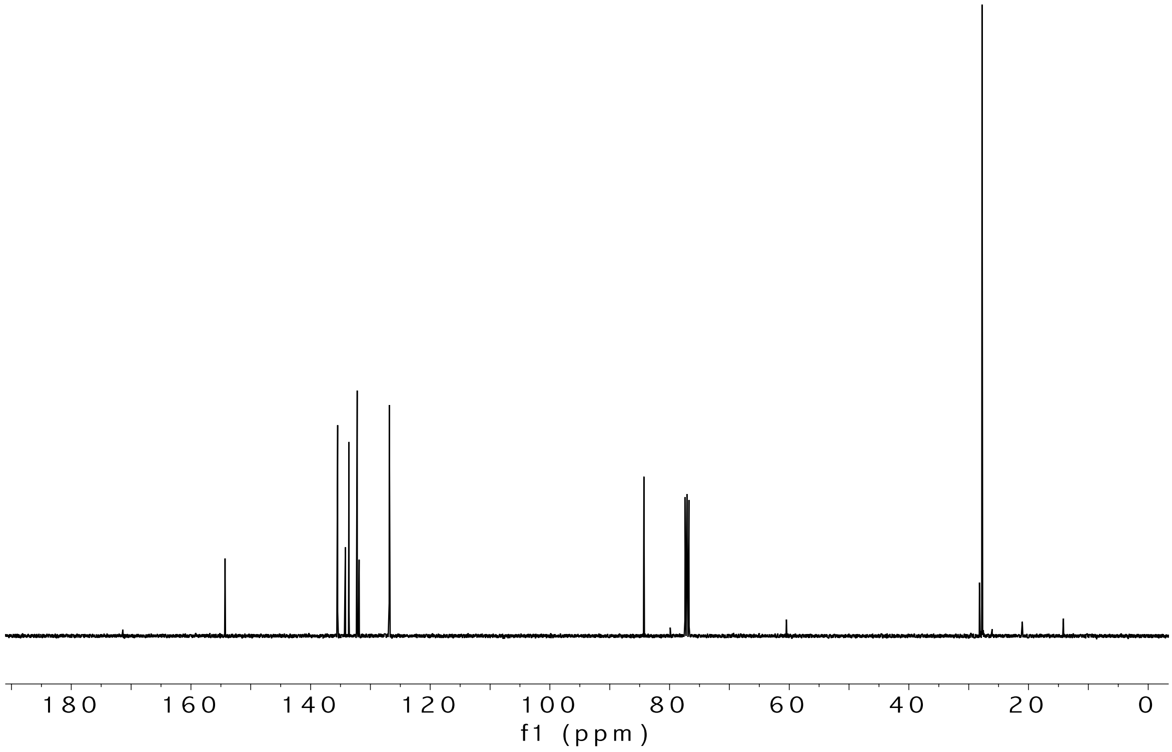

tert-Butyl (((4-chlorophenyl)sulfonyl)oxy)carbamate 1o

400 MHz, $CDCl_3$

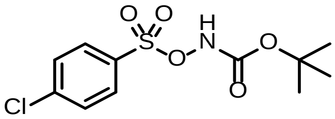

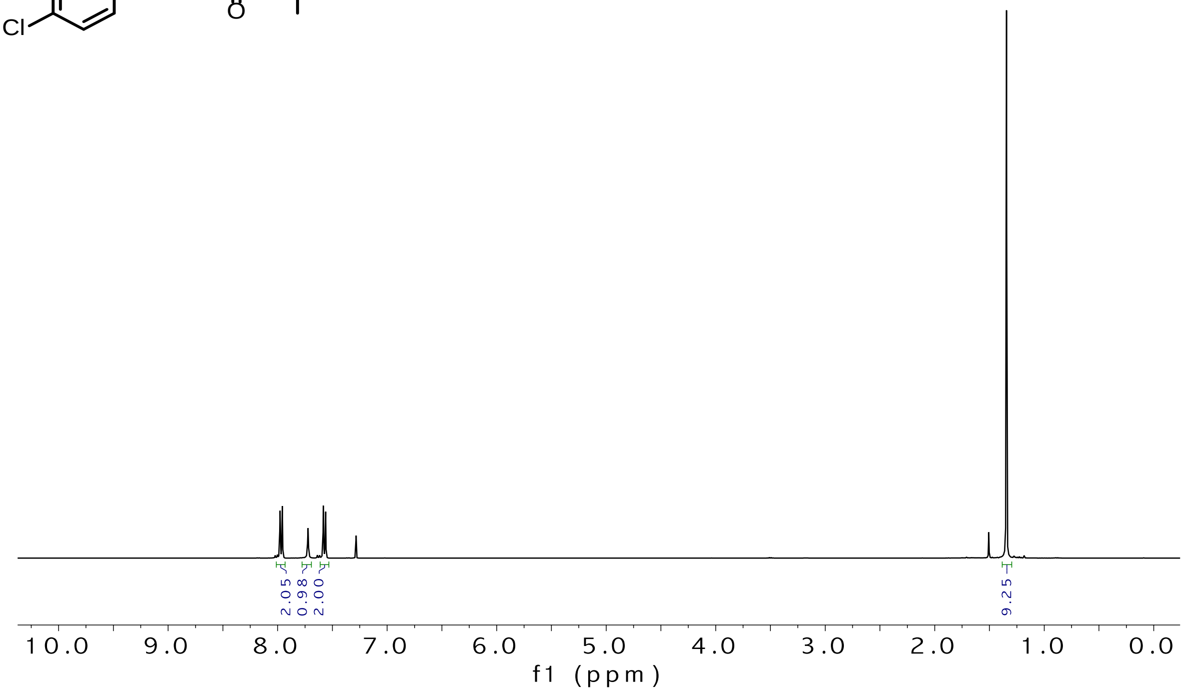

101 MHz, $CDCl_3$

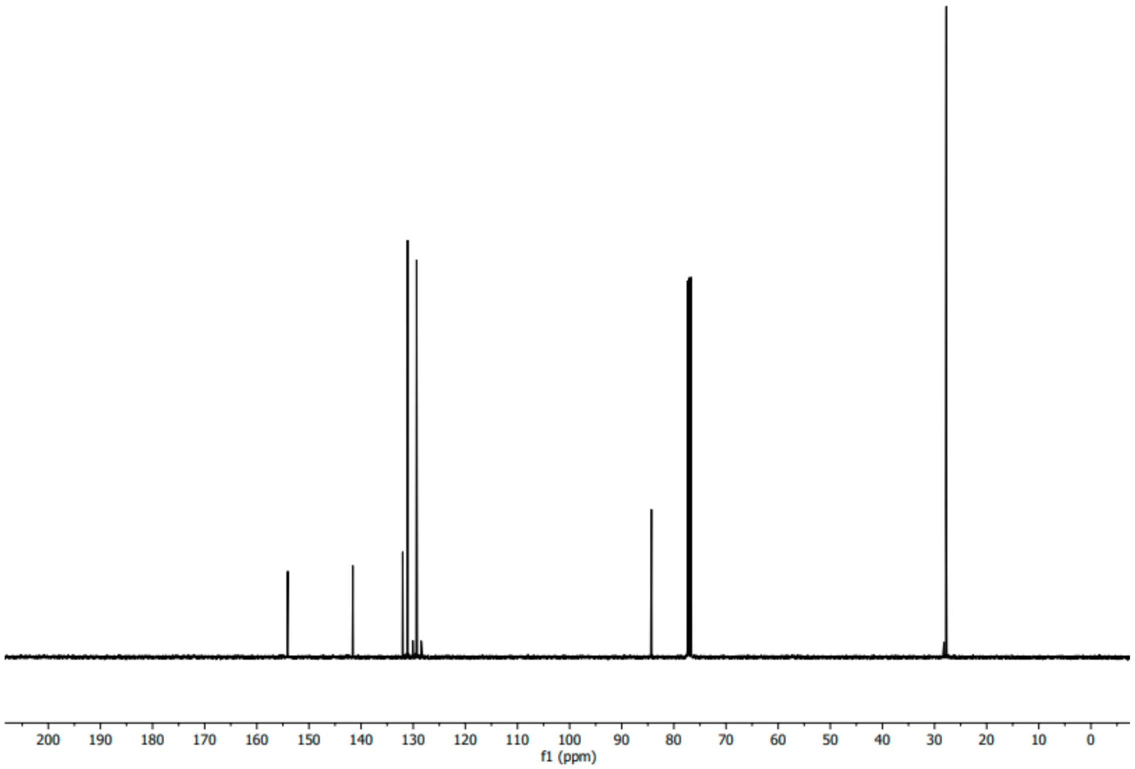

tert-Butyl (((3,5-dichlorophenyl)sulfonyl)oxy)carbamate 1p

400 MHz, $CDCl_3$

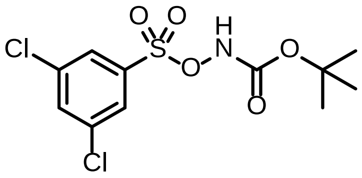

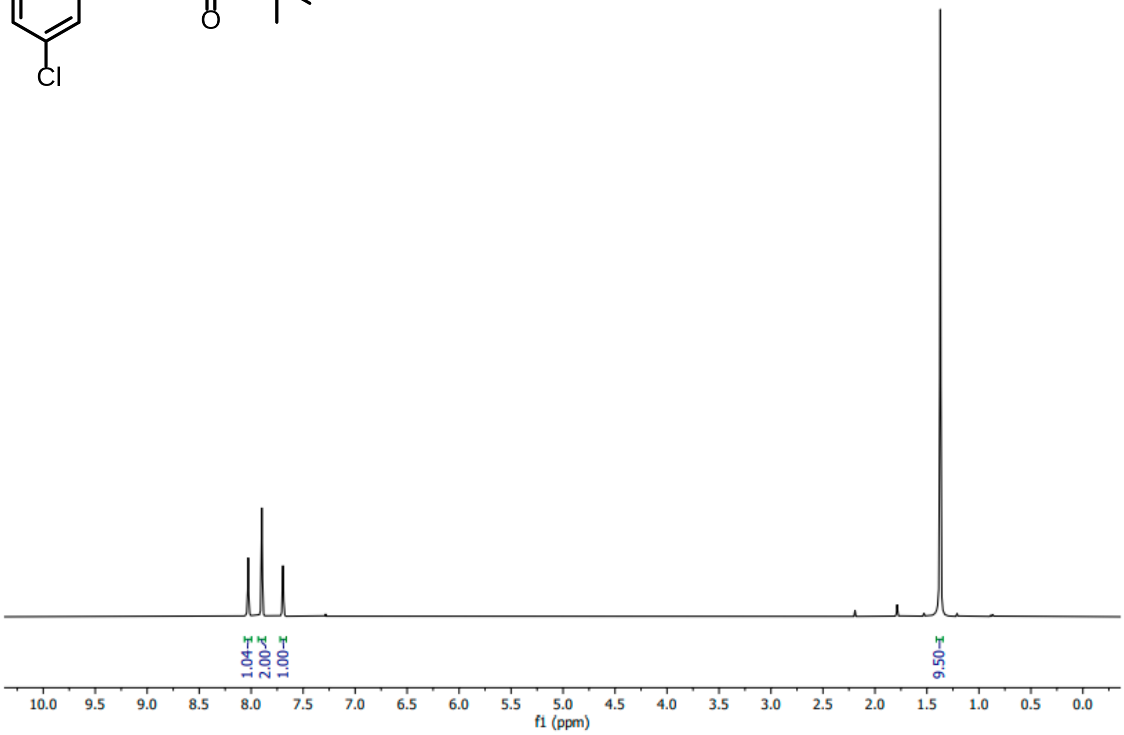

101 MHz, $CDCl_3$

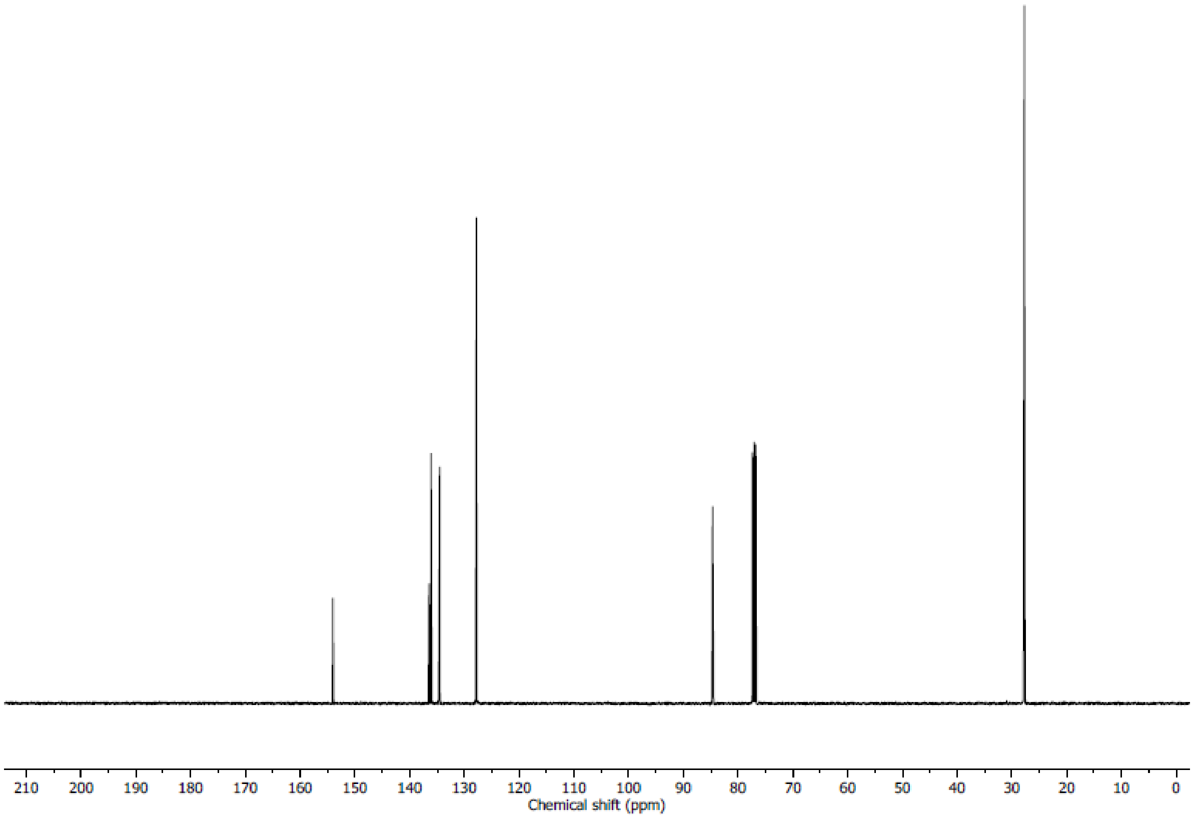

tert-Butyl (((3-chloro-4-methylphenyl)sulfonyl)oxy)carbamate 1q

400 MHz, $CDCl_3$

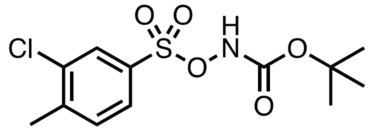

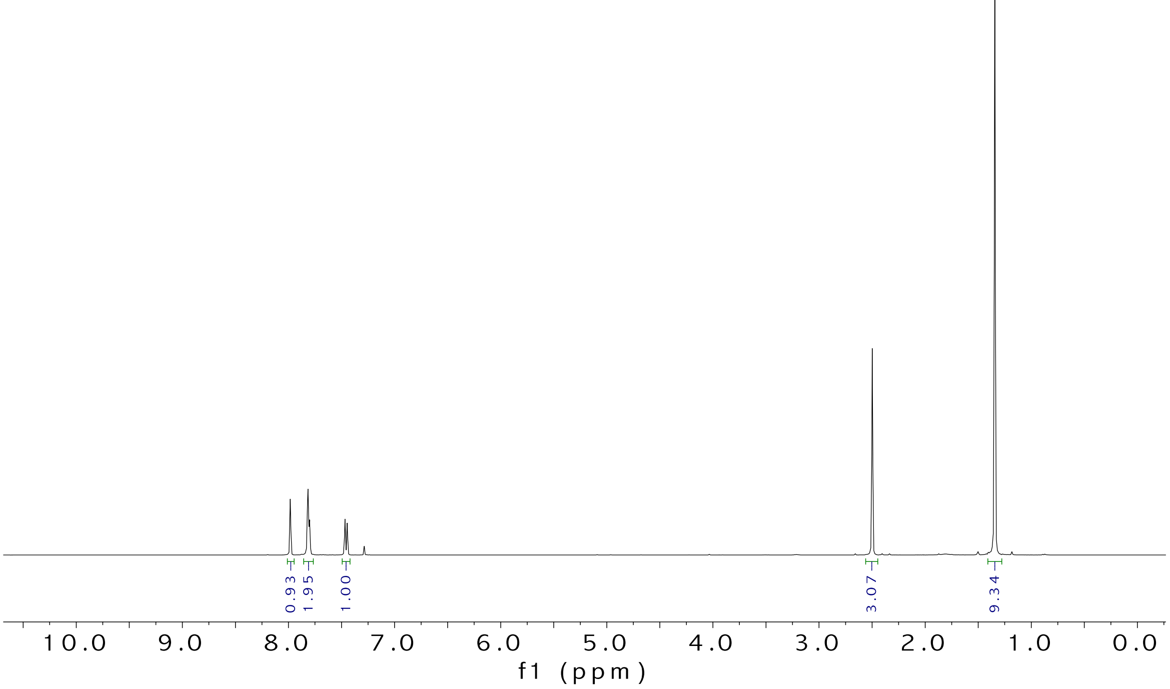

101 MHz, $CDCl_3$

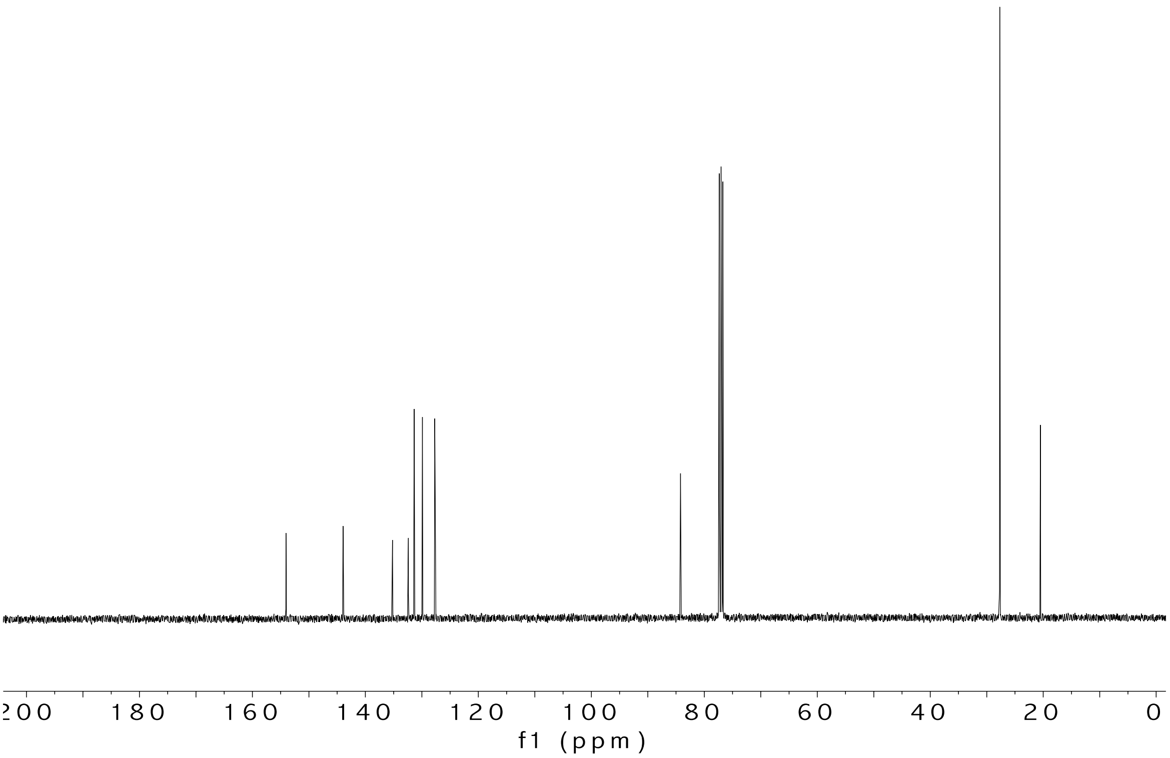

tert-Butyl (((4-bromophenyl)sulfonyl)oxy)carbamate 1r

500 MHz, $CDCl_3$

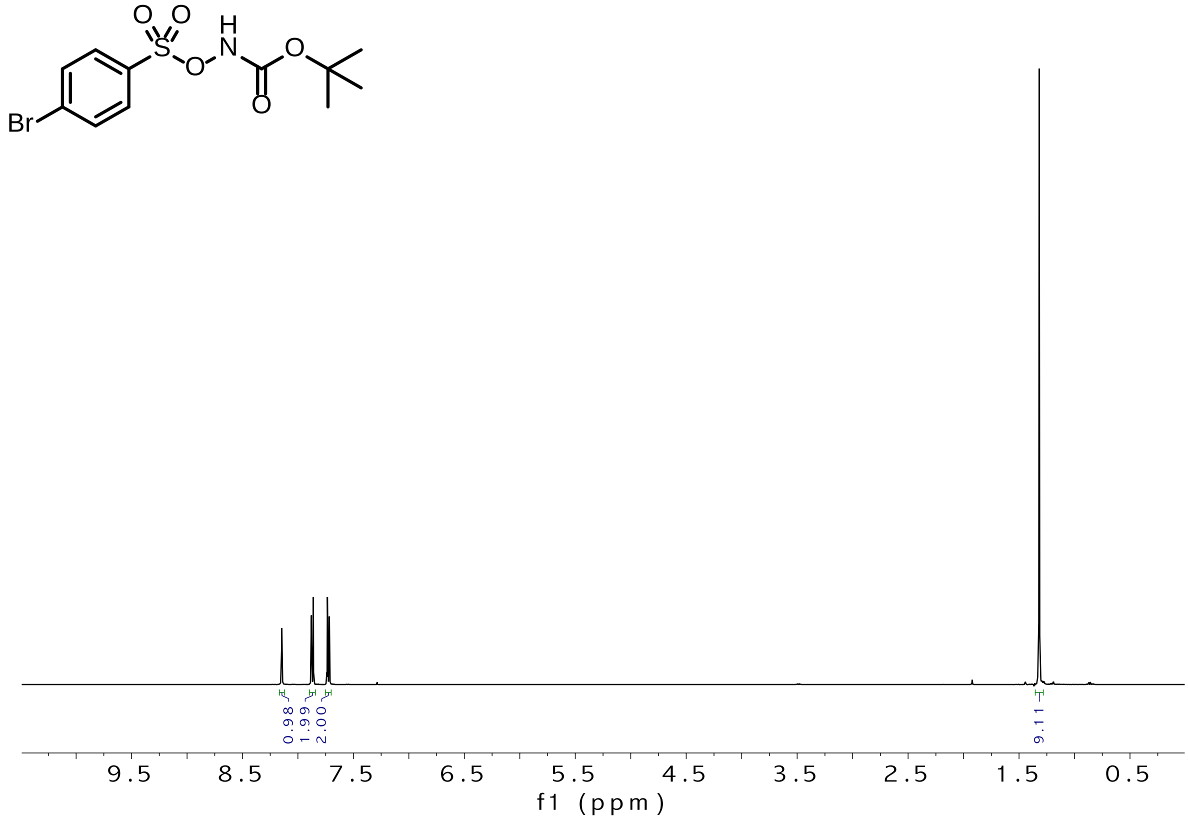

126 MHz, $CDCl_3$

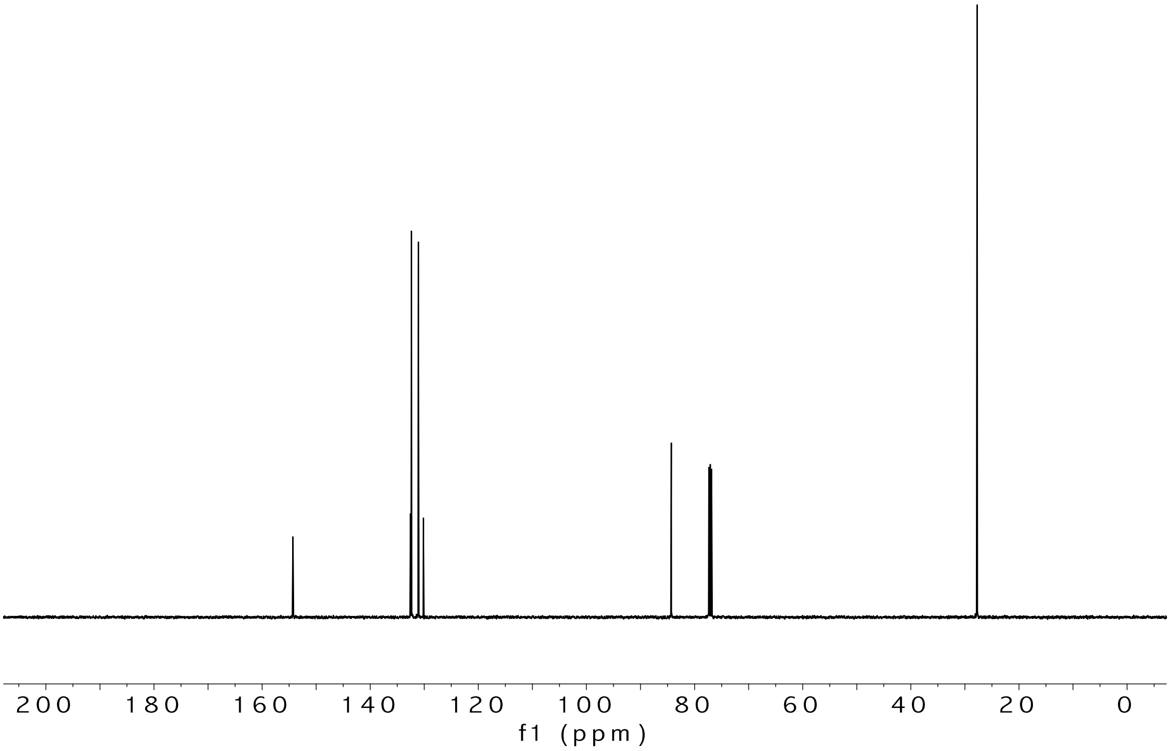

tert-Butyl (((3-bromophenyl)sulfonyl)oxy)carbamate 1s

400 MHz, $CDCl_3$

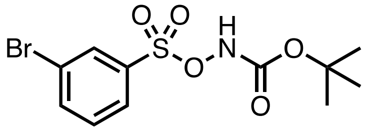

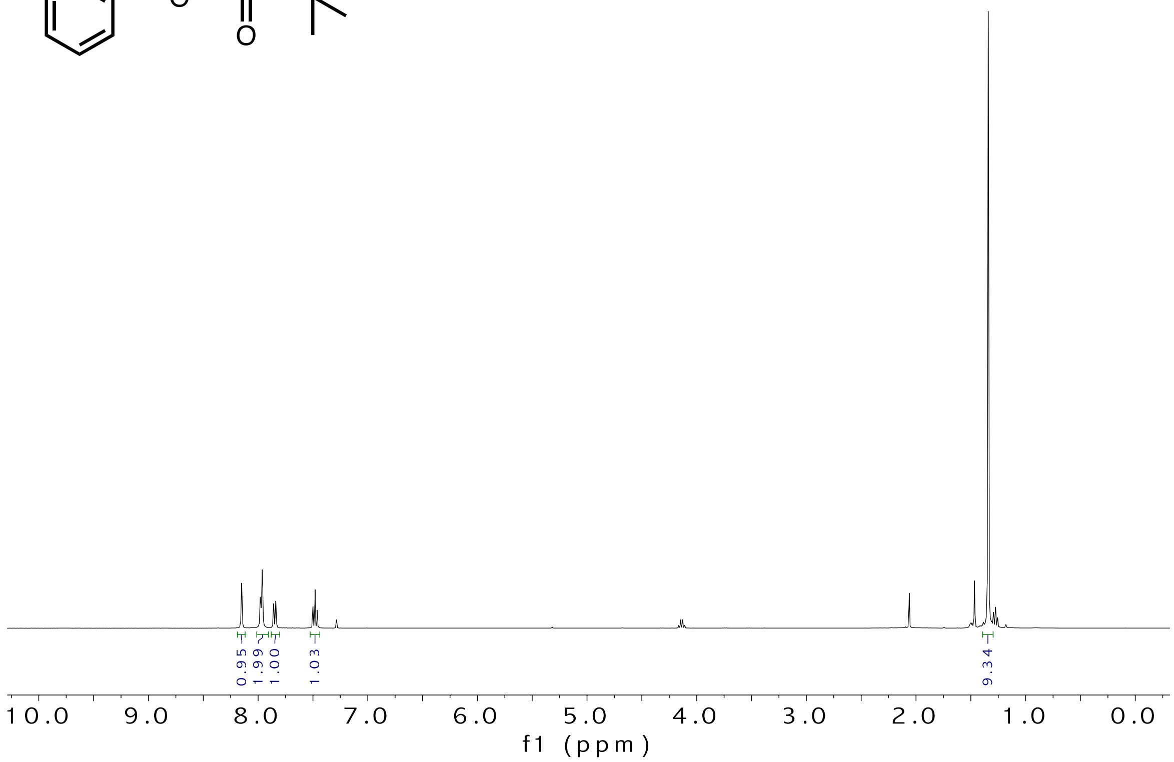

101 MHz, $CDCl_3$

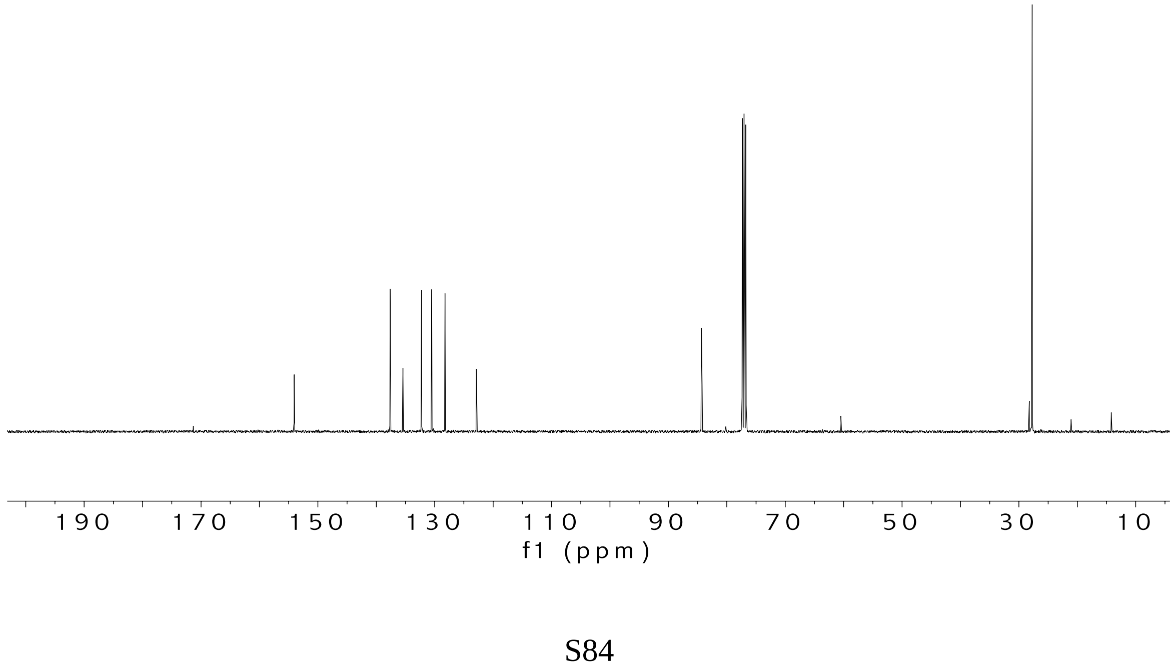

tert-Butyl (((4-methoxyphenyl)sulfonyl)oxy)carbamate 1t

700 MHz, $CDCl_3$

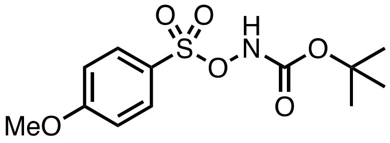

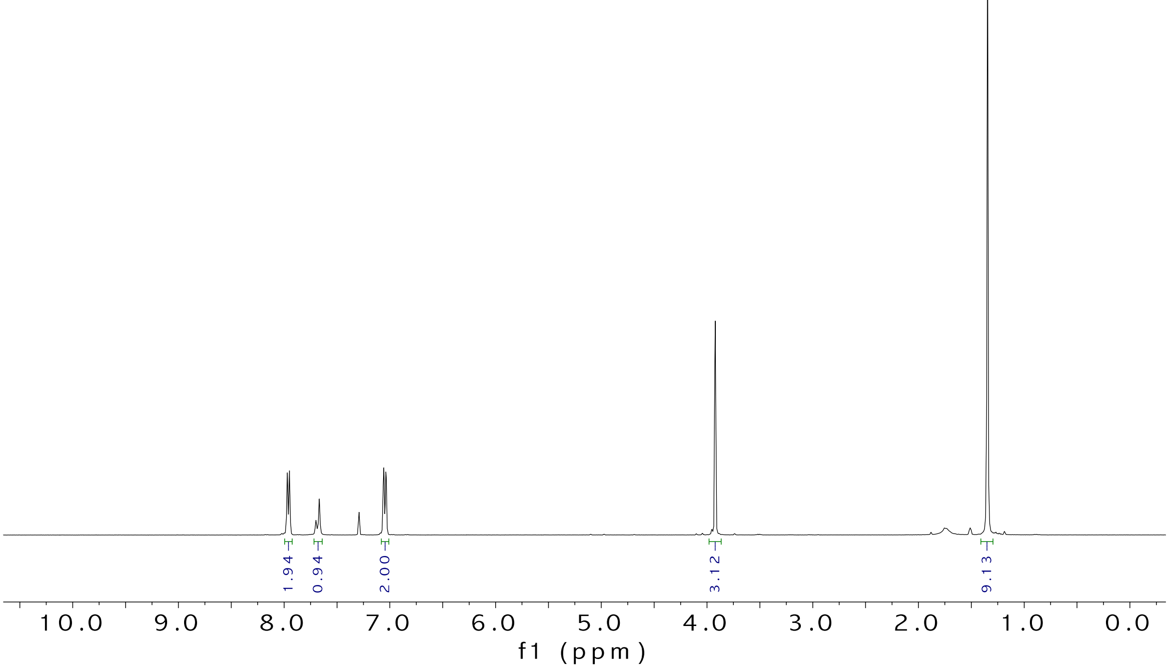

101 MHz, $CDCl_3$

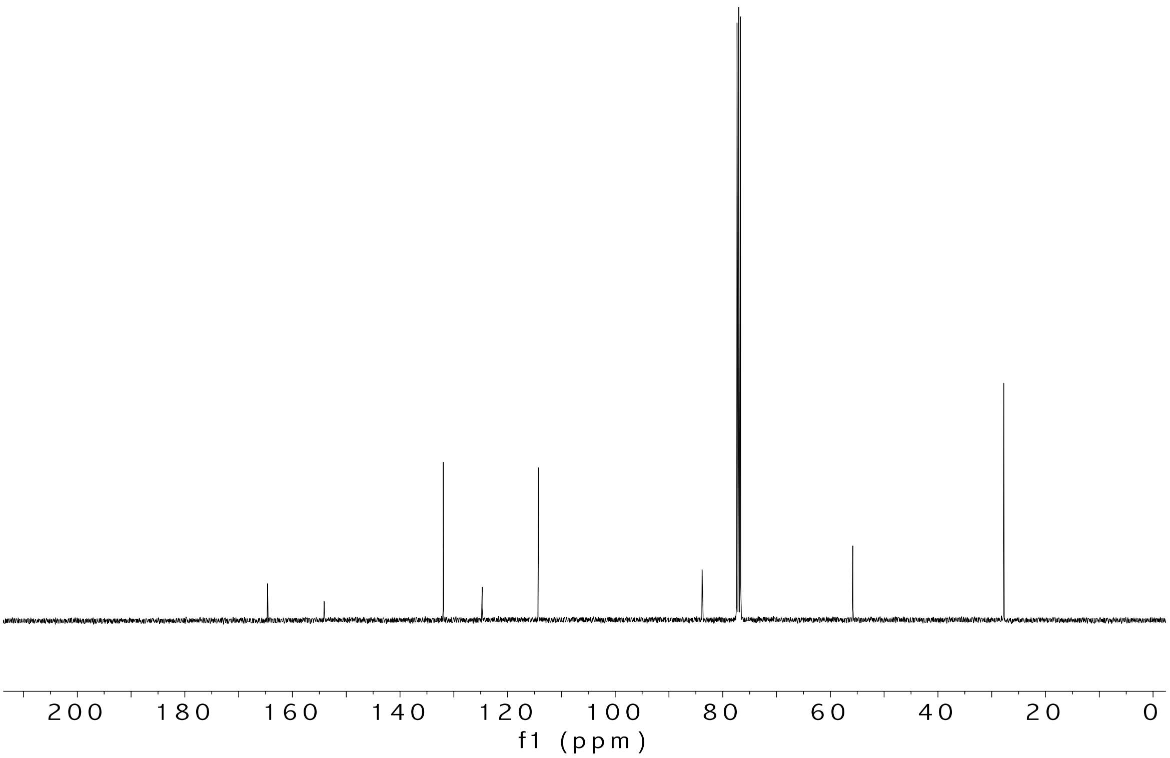

tert-Butyl (((3-fluoro-4-methoxyphenyl)sulfonyl)oxy)carbamate 1u

400 MHz, $CDCl_3$

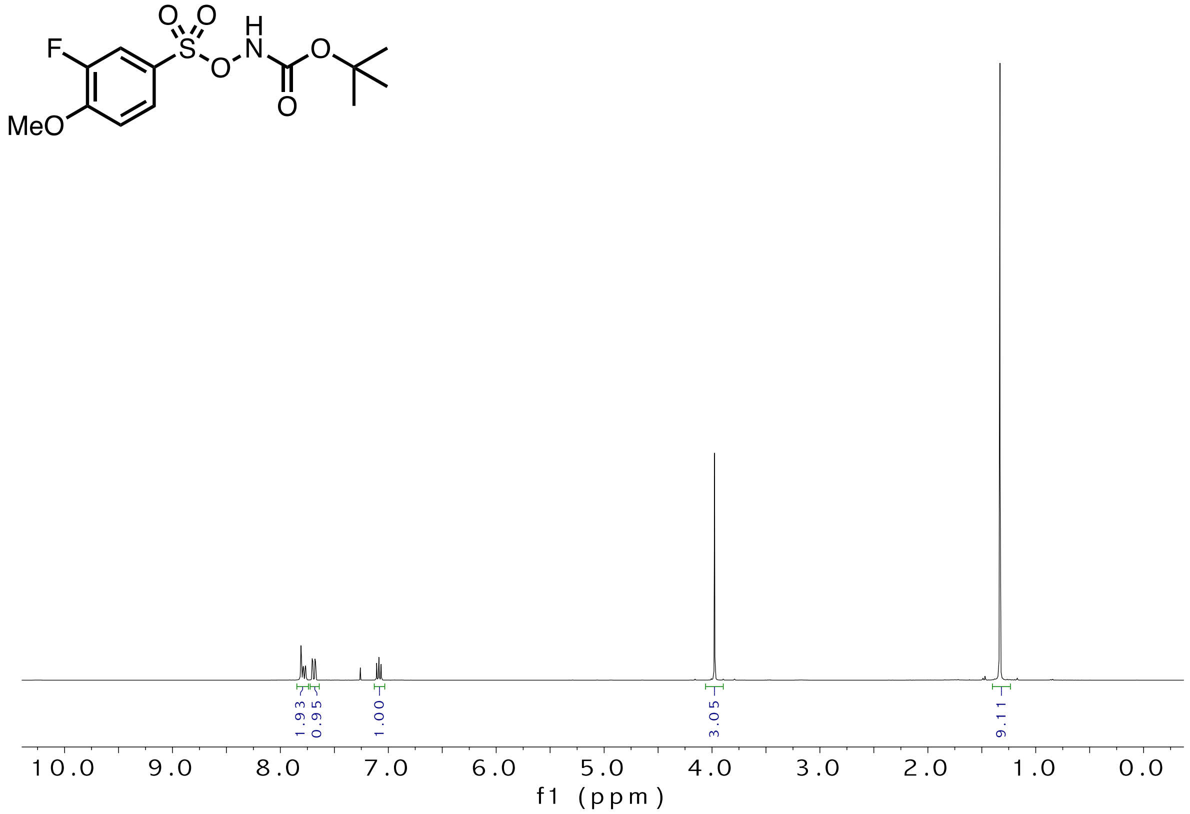

101 MHz, $CDCl_3$

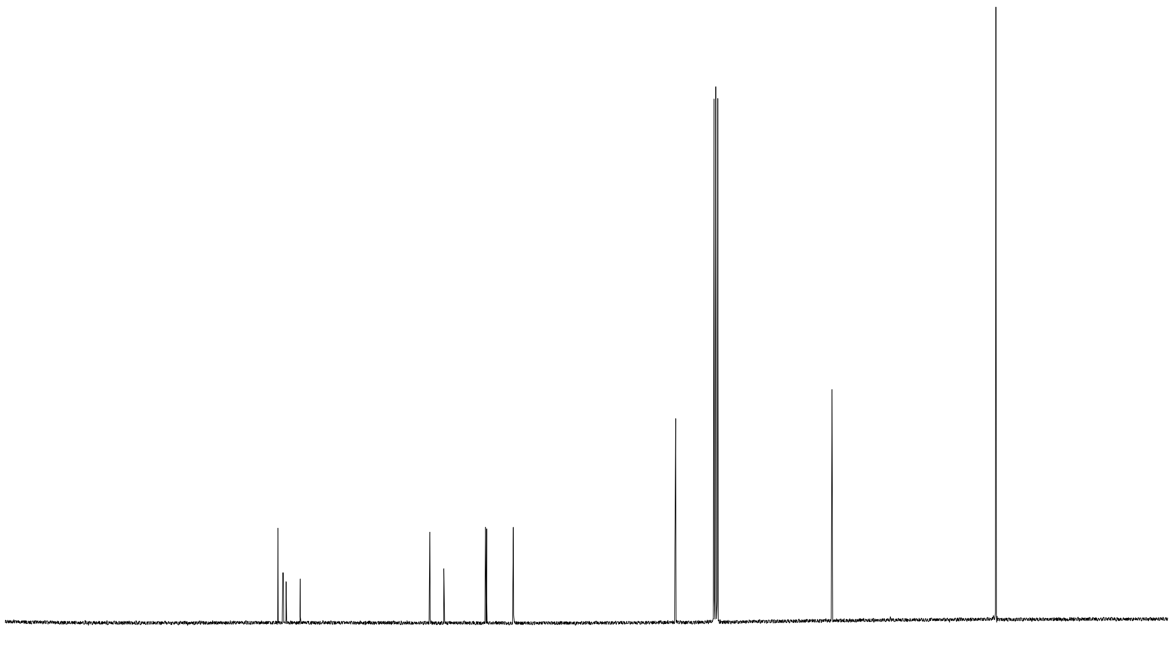

376 MHz, $CDCl_3$

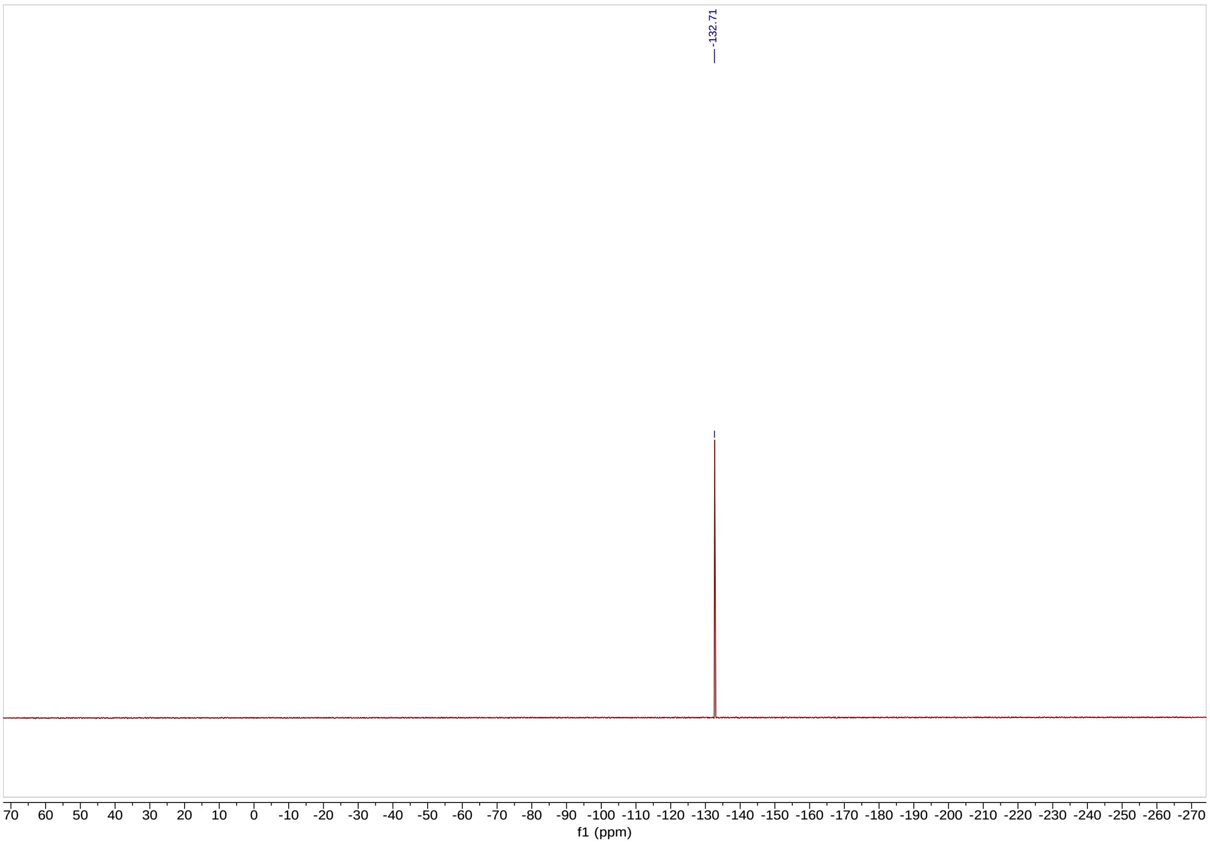

tert-Butyl (((3,4-dimethoxyphenyl)sulfonyl)oxy)carbamate 1v

400 MHz, $CDCl_3$

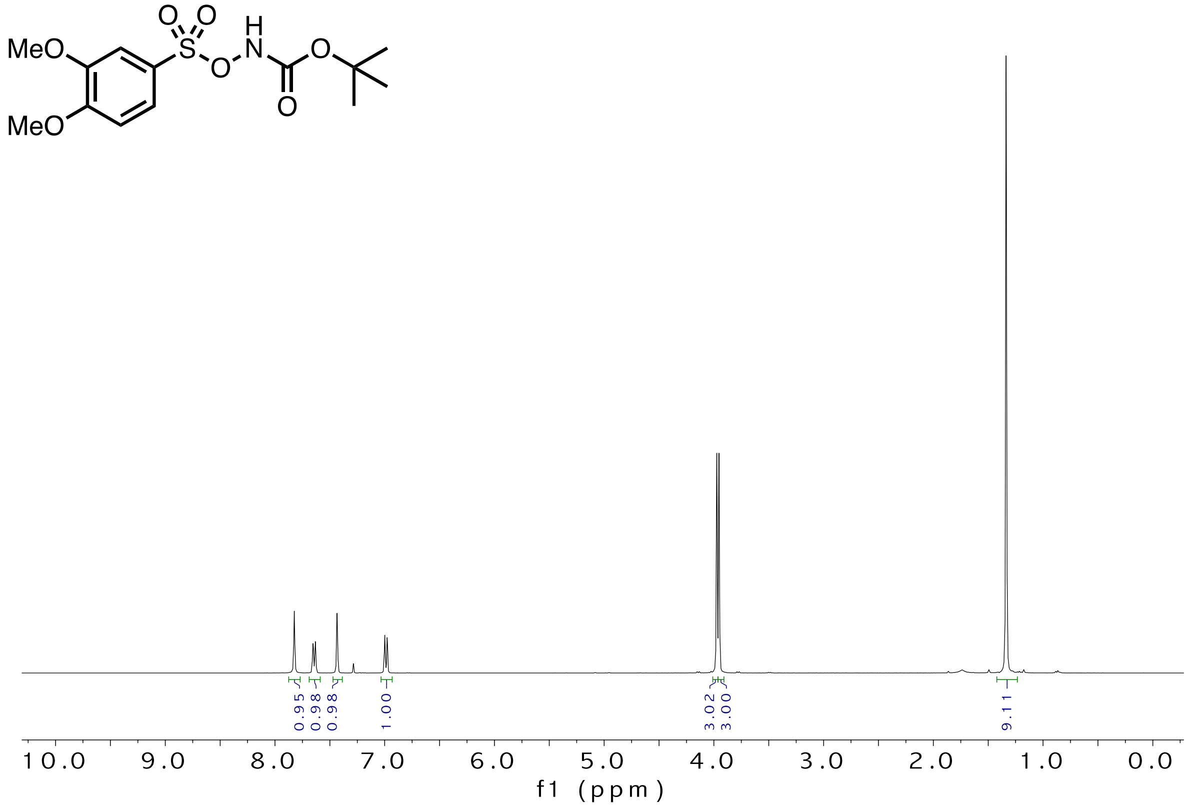

101 MHz, $CDCl_3$

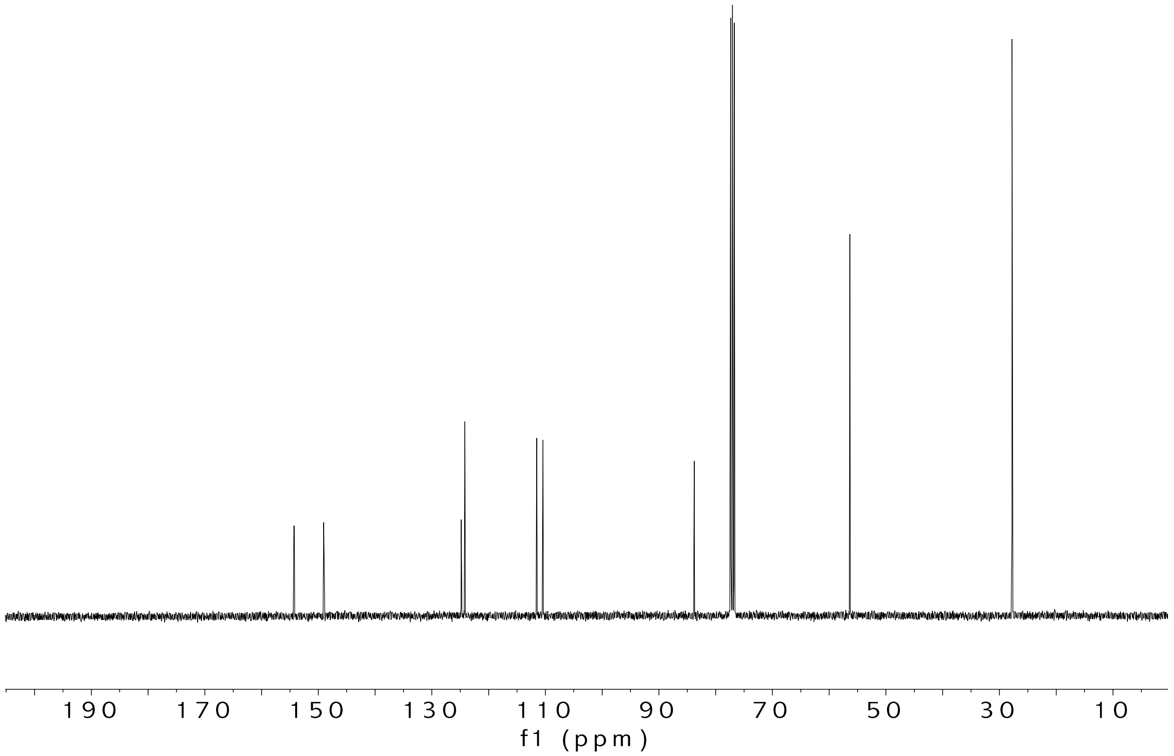

tert-Butyl ((naphthalen-2-ylsulfonyl)oxy)carbamate 1w

400 MHz, $CDCl_3$

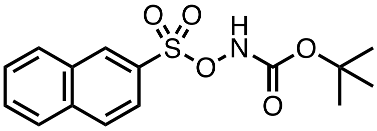

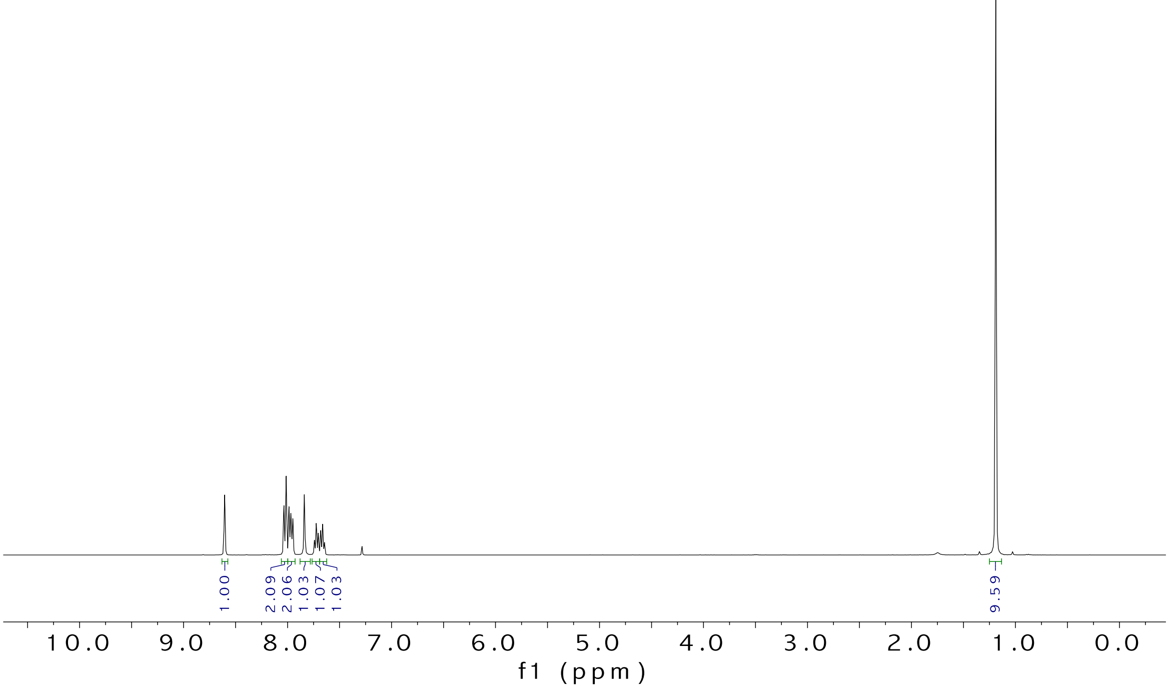

101 MHz, $CDCl_3$

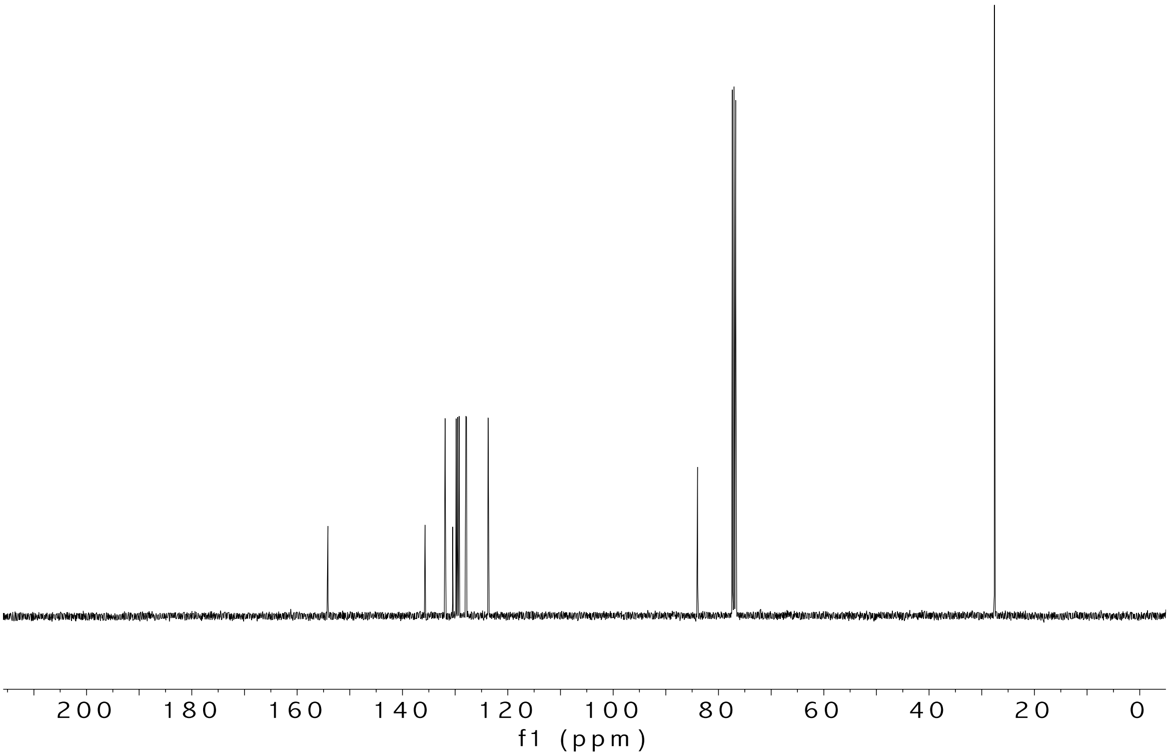

***tert*-Butyl (((3-(trifluoromethoxy)phenyl)sulfonyl)oxy)carbamate 1x**

400 MHz, $CDCl_3$

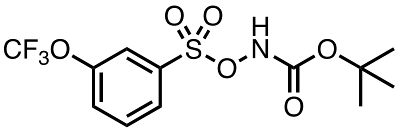

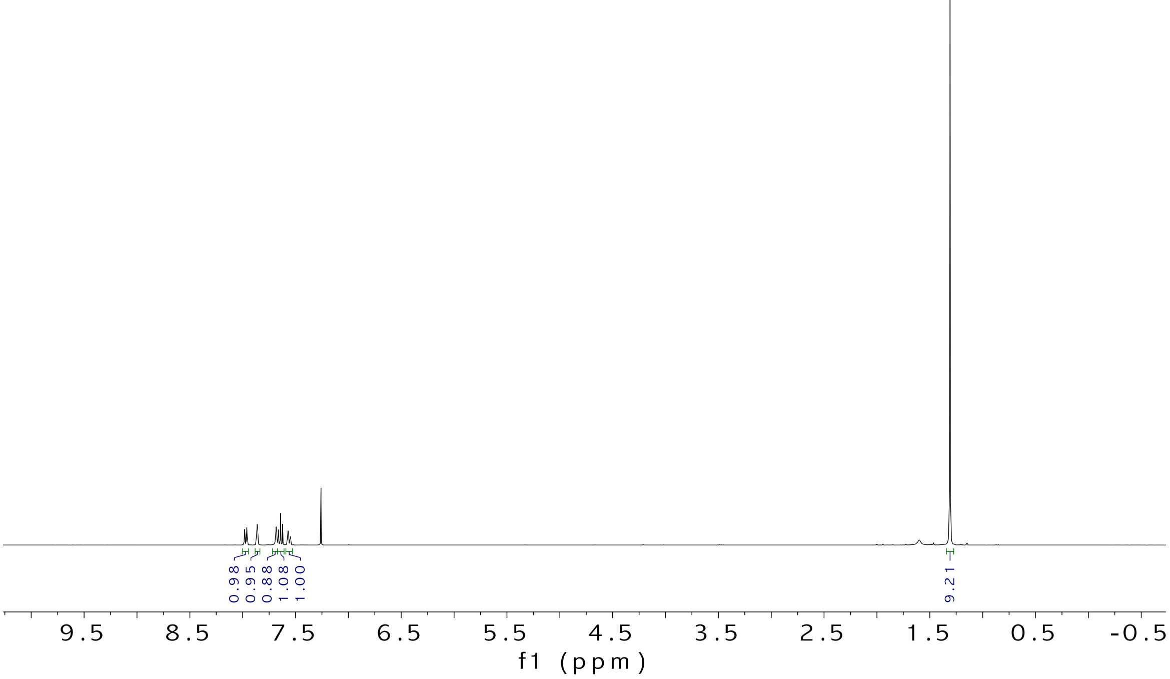

101 MHz, $CDCl_3$

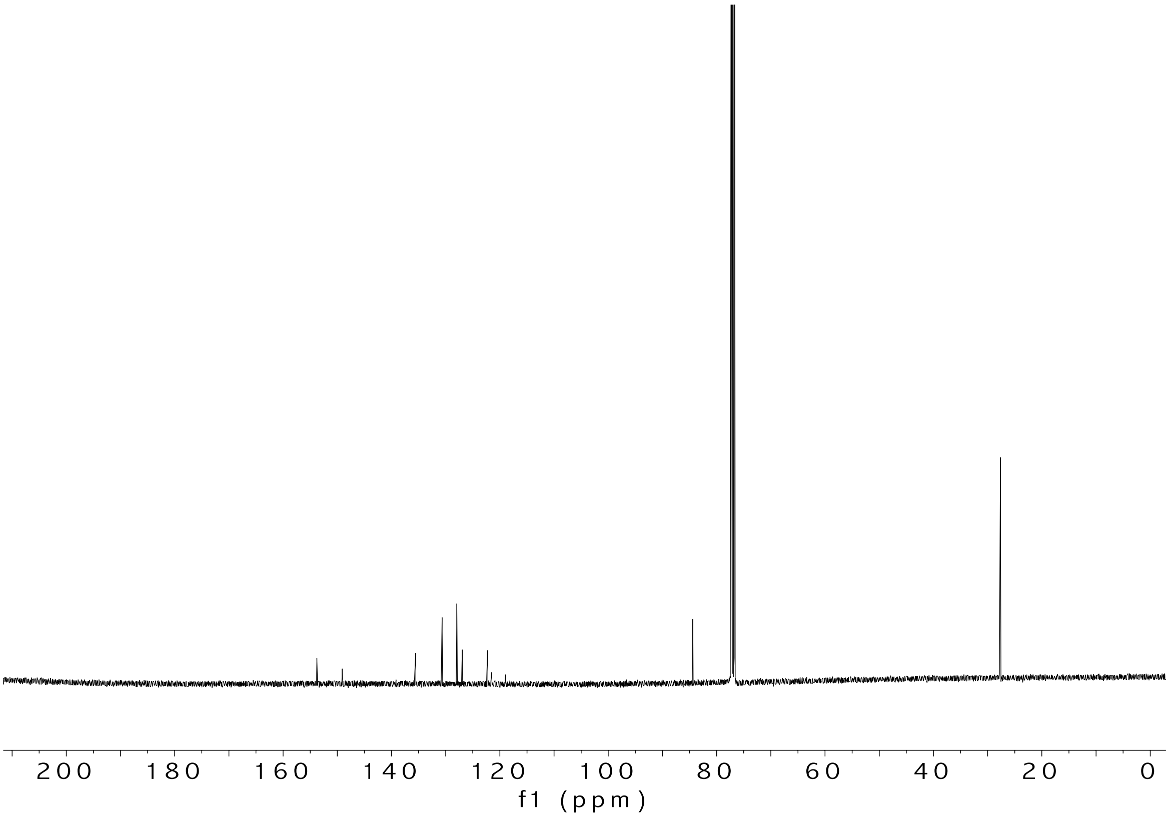

376 MHz, $CDCl_3$

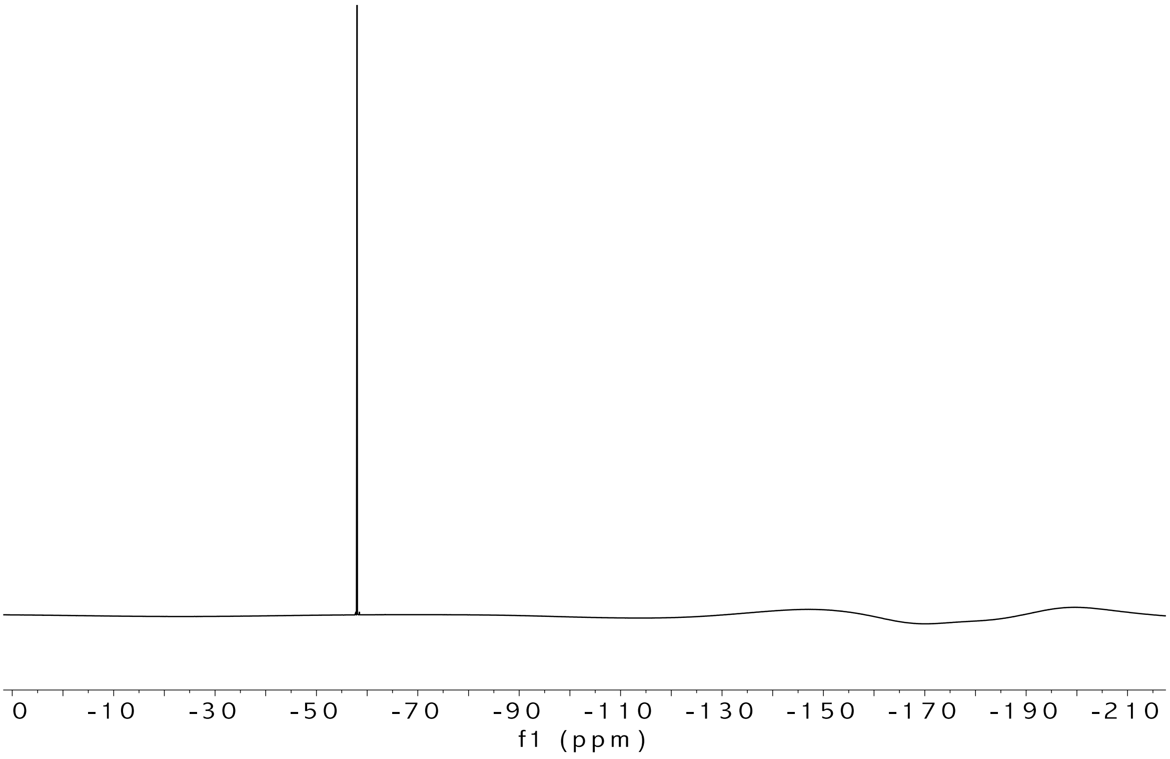

***tert*-Butyl (((4-(trifluoromethyl)phenyl)sulfonyl)oxy)carbamate 1y**

400 MHz, $CDCl_3$

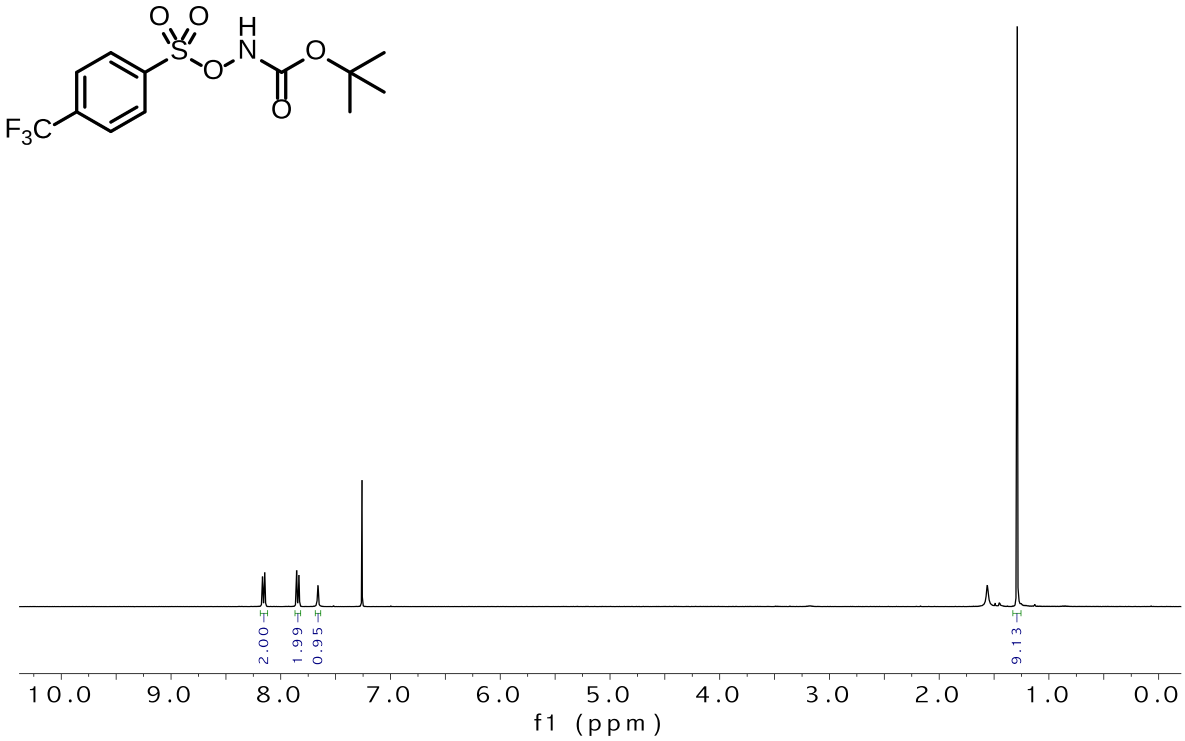

101 MHz, $CDCl_3$

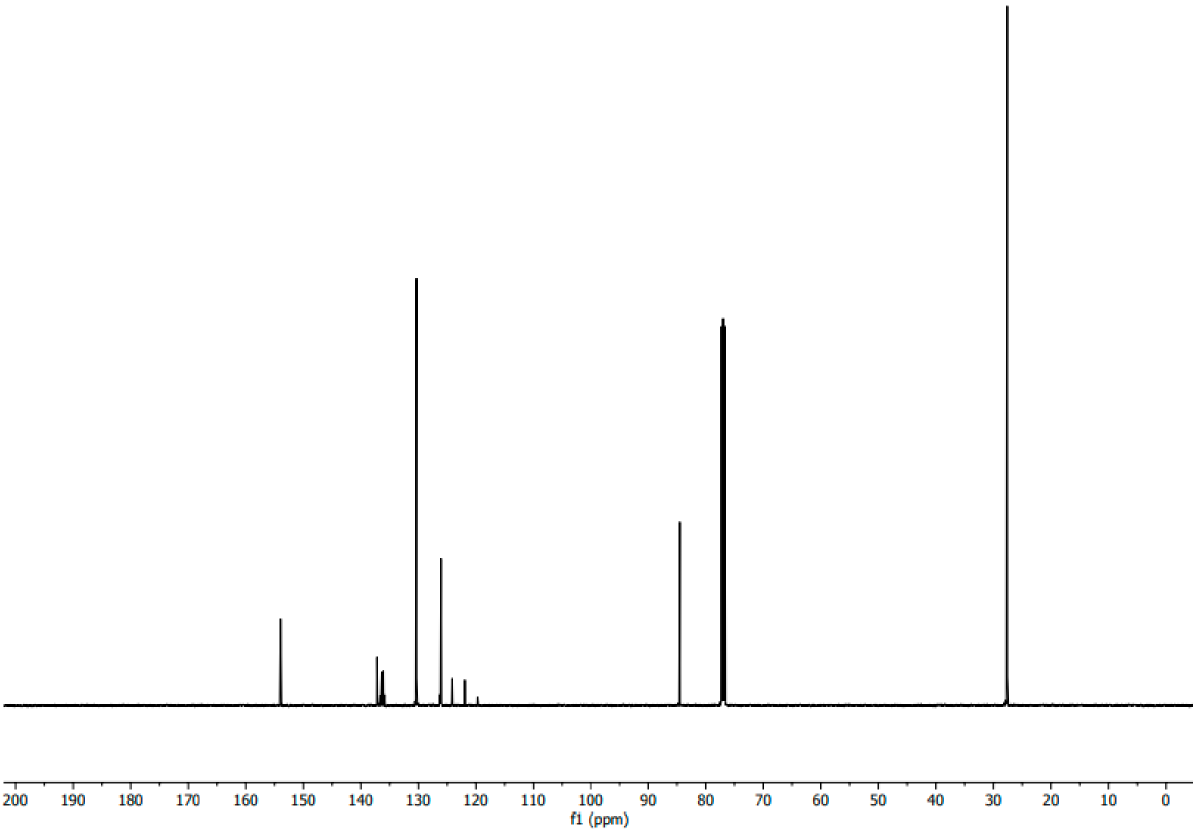

471 MHz, $CDCl_3$

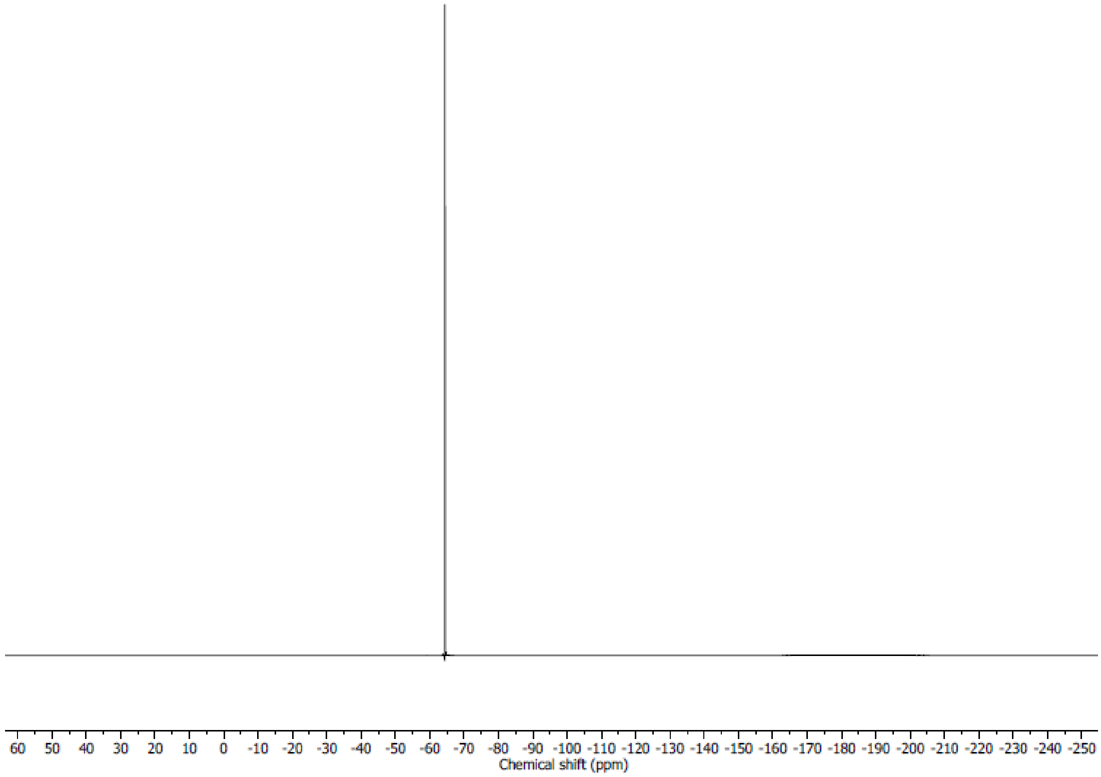

Methyl 4-((((*tert*-butoxycarbonyl)amino)oxy)sulfonyl)benzoate 1z

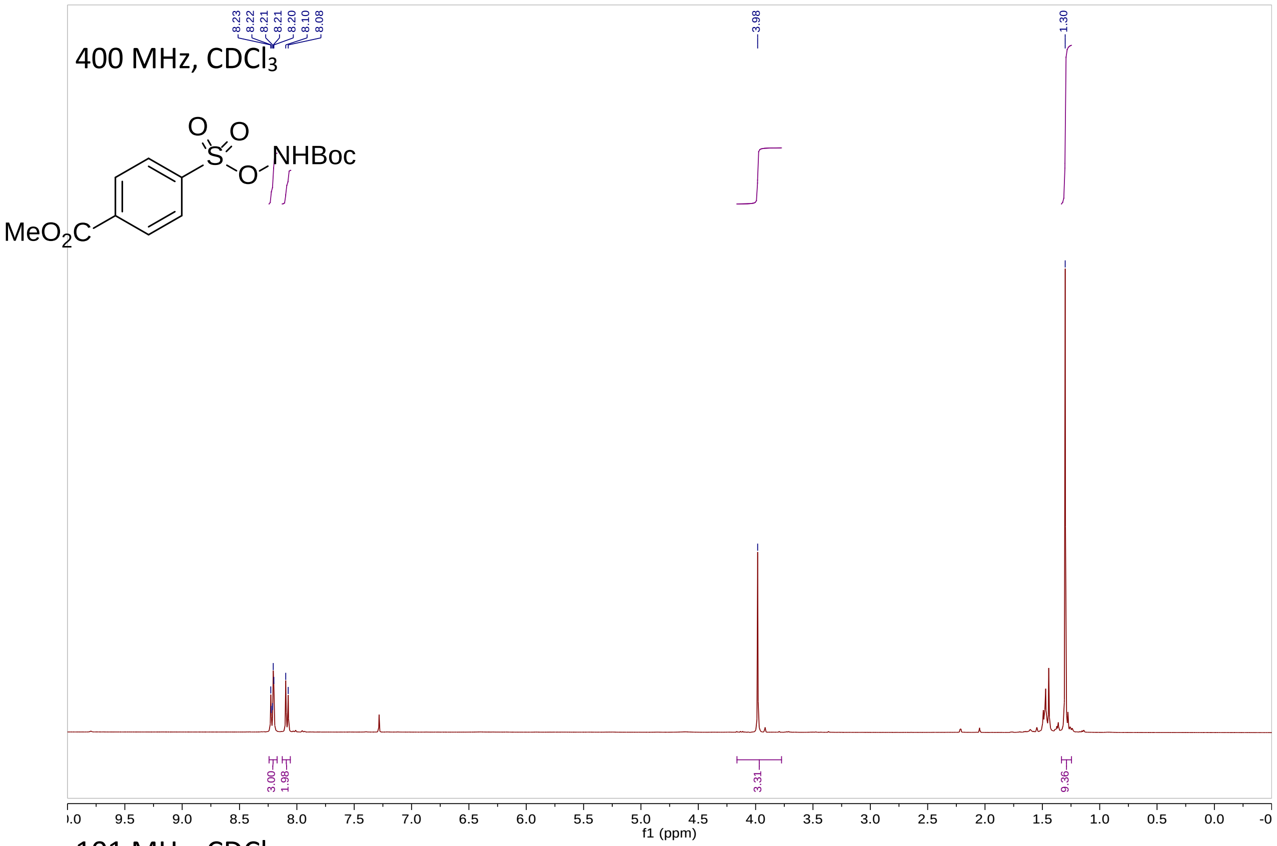

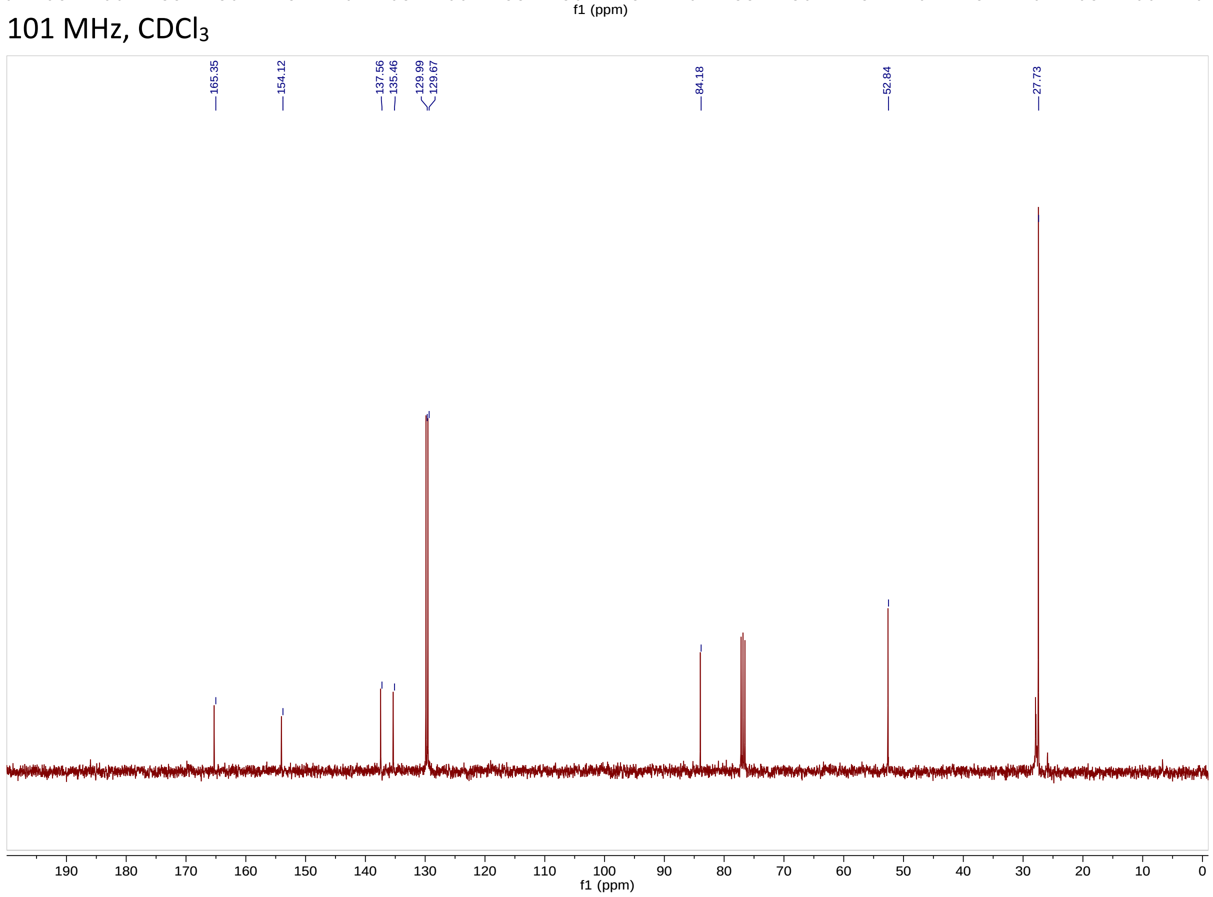

***tert*-Butyl (((2-(trifluoromethyl)phenyl)sulfonyl)oxy)carbamate 1za**

400 MHz, $CDCl_3$

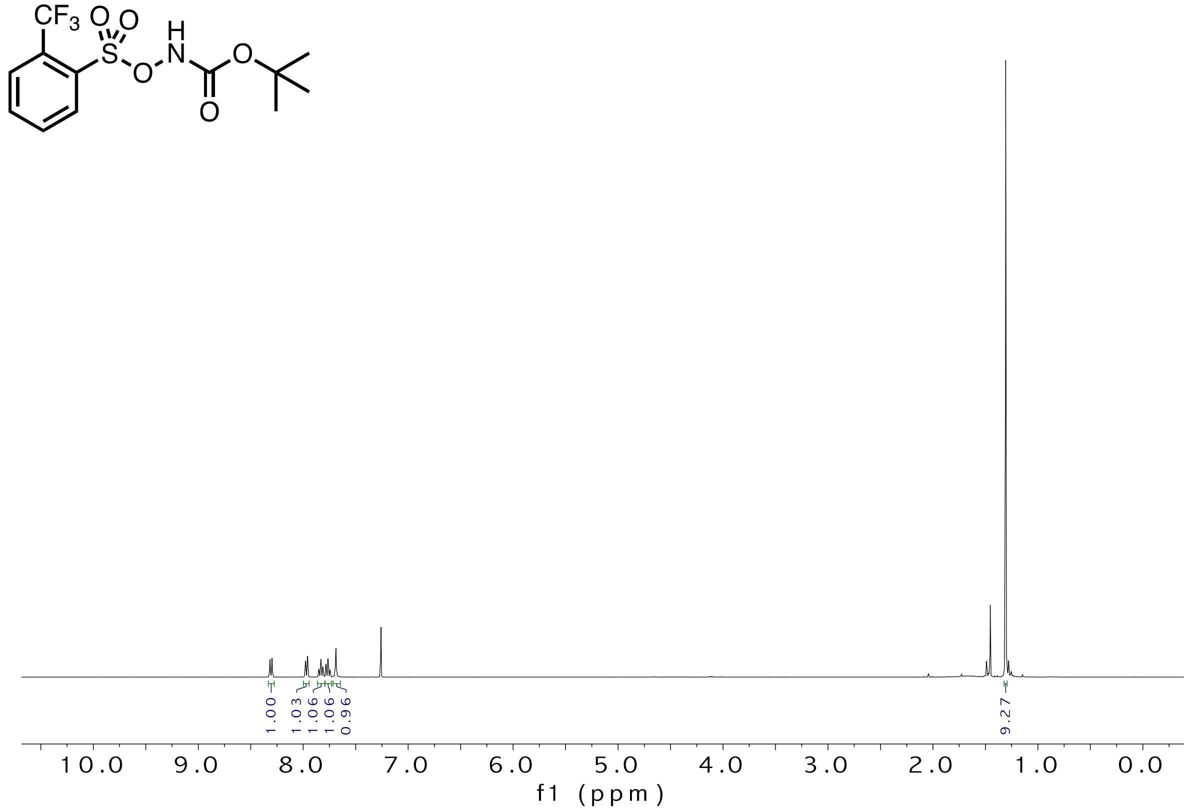

101 MHz, $CDCl_3$

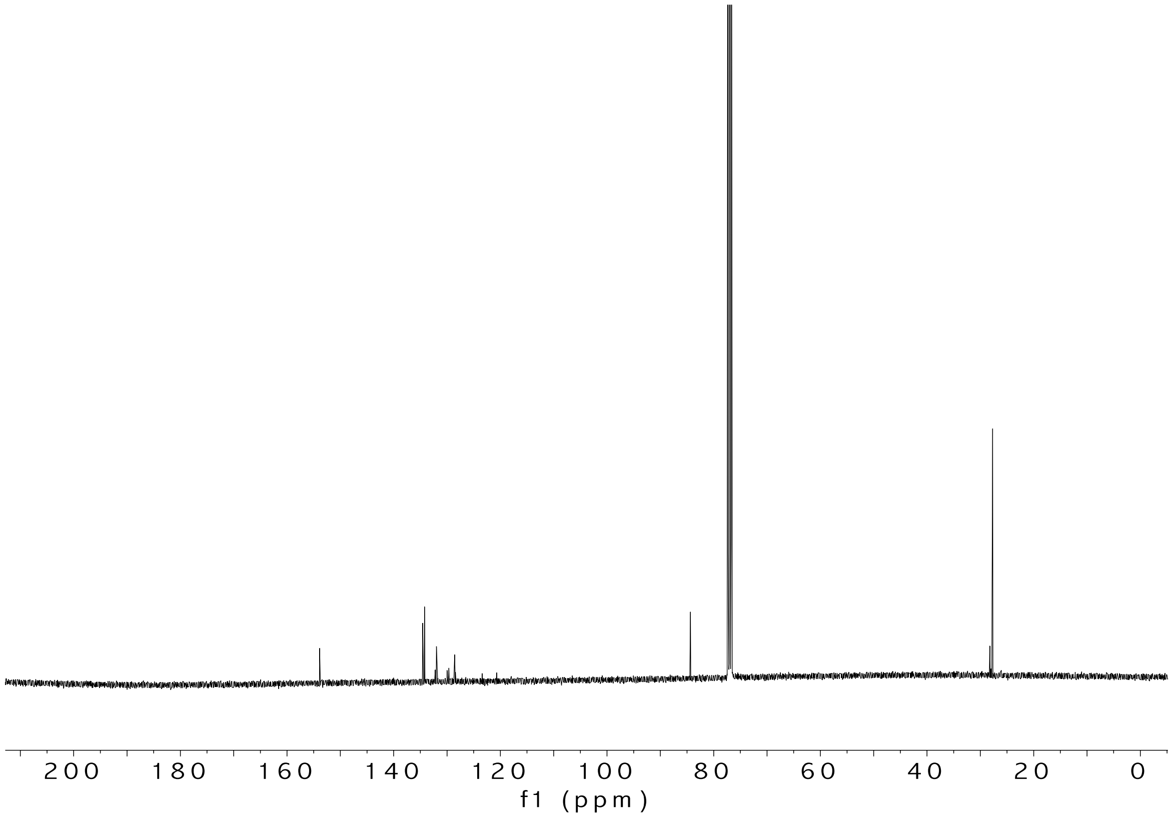

376 MHz, $CDCl_3$

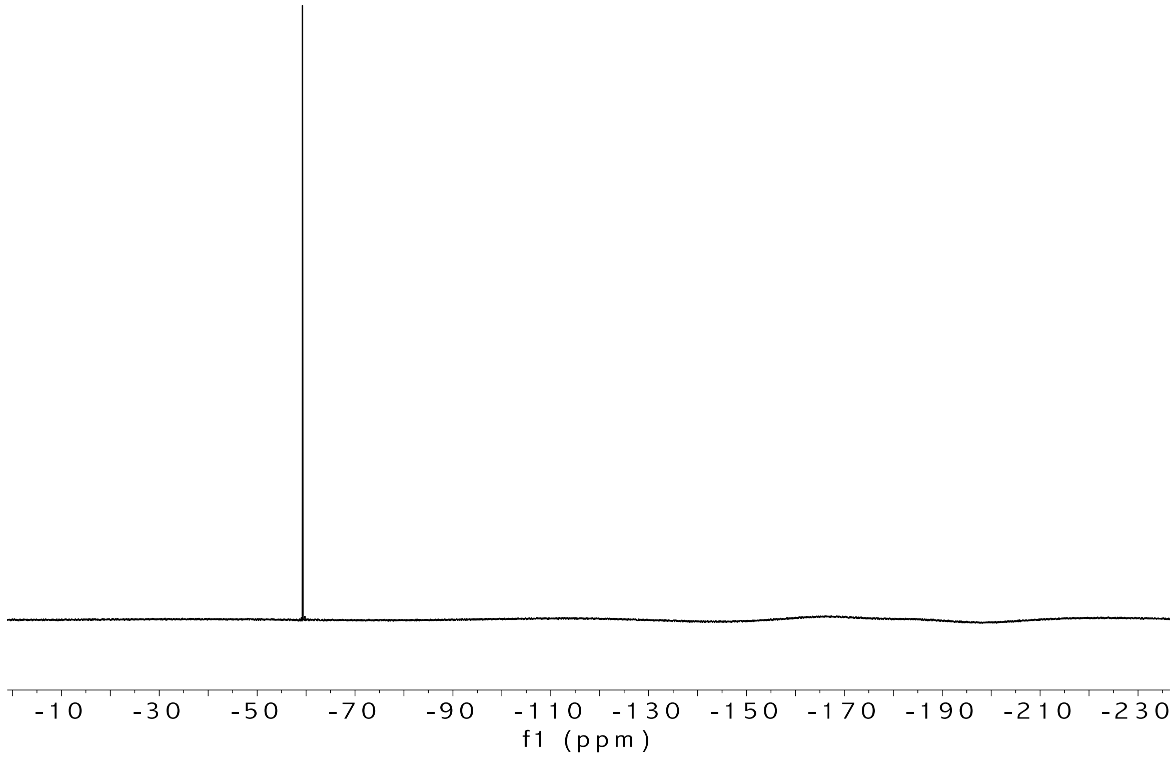

tert-Butyl (((3-(methylsulfonyl)phenyl)sulfonyl)oxy)carbamate 1zb

400 MHz, $CDCl_3$

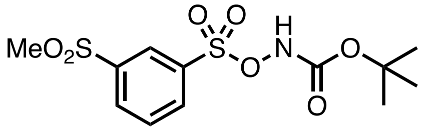

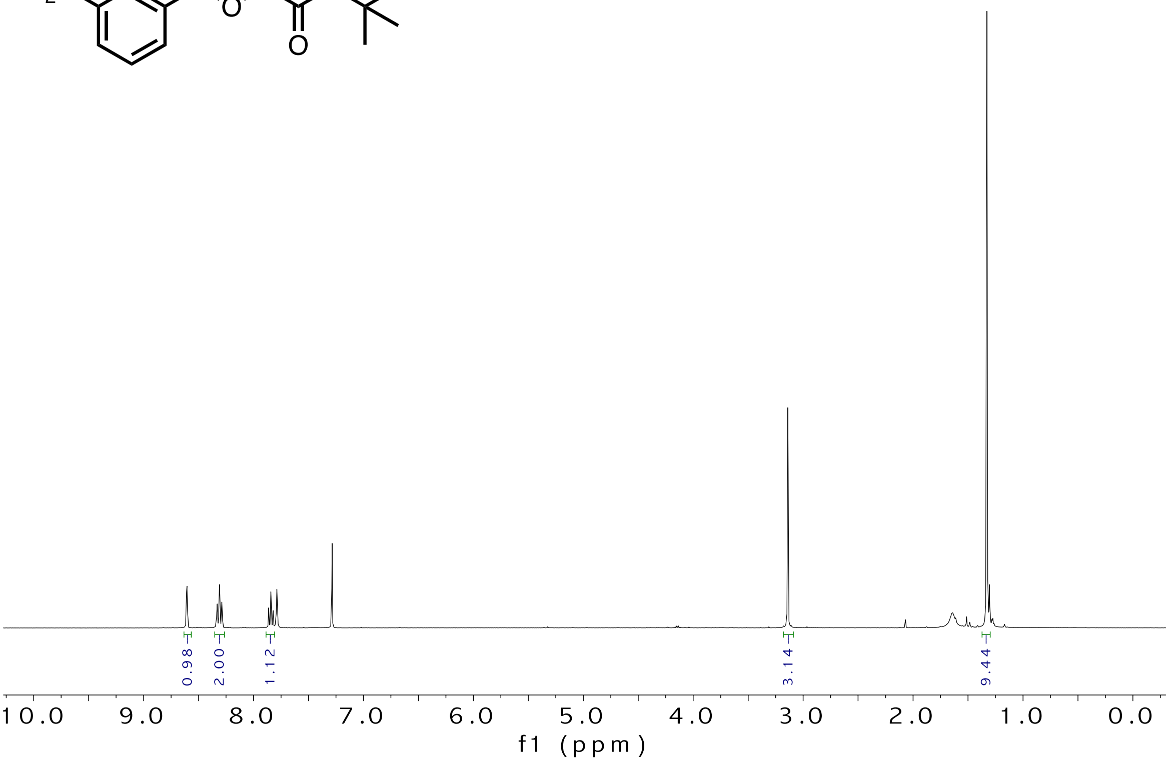

101 MHz, $CDCl_3$

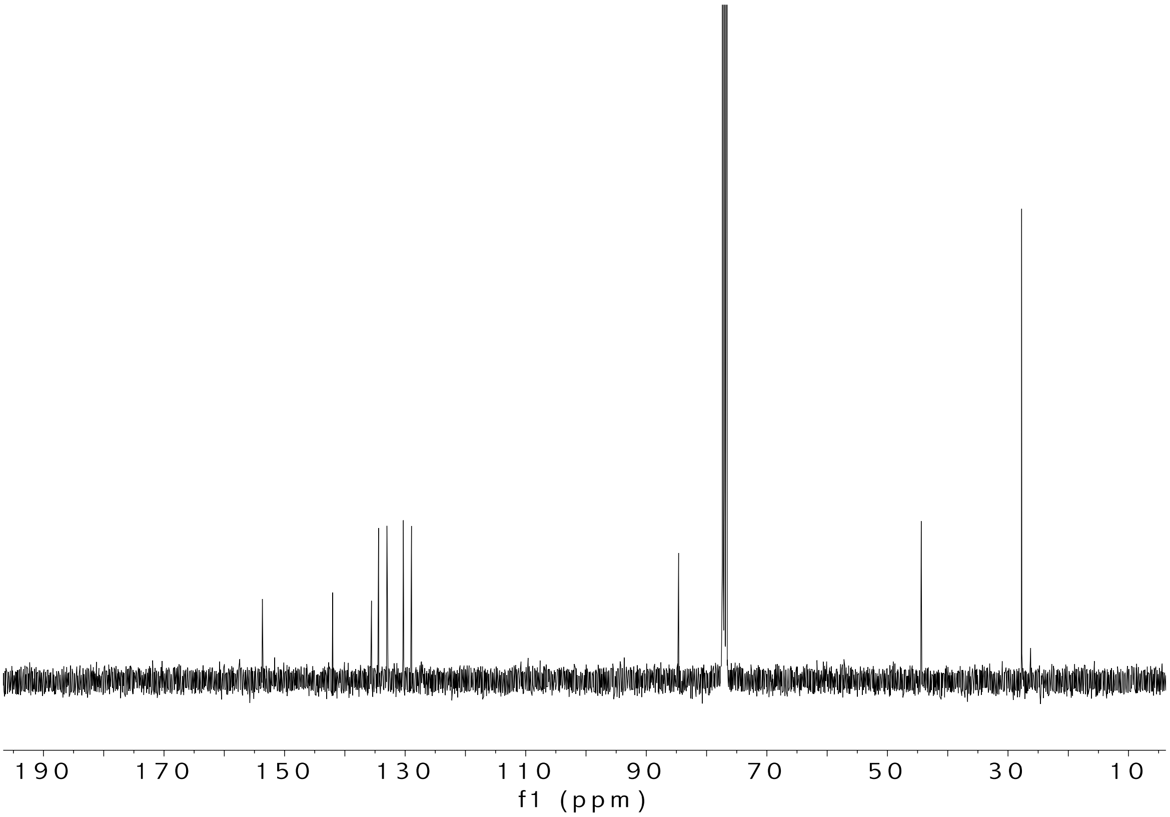

tert-Butyl (((4-nitrophenyl)sulfonyl)oxy)carbamate

400 MHz, $CDCl_3$

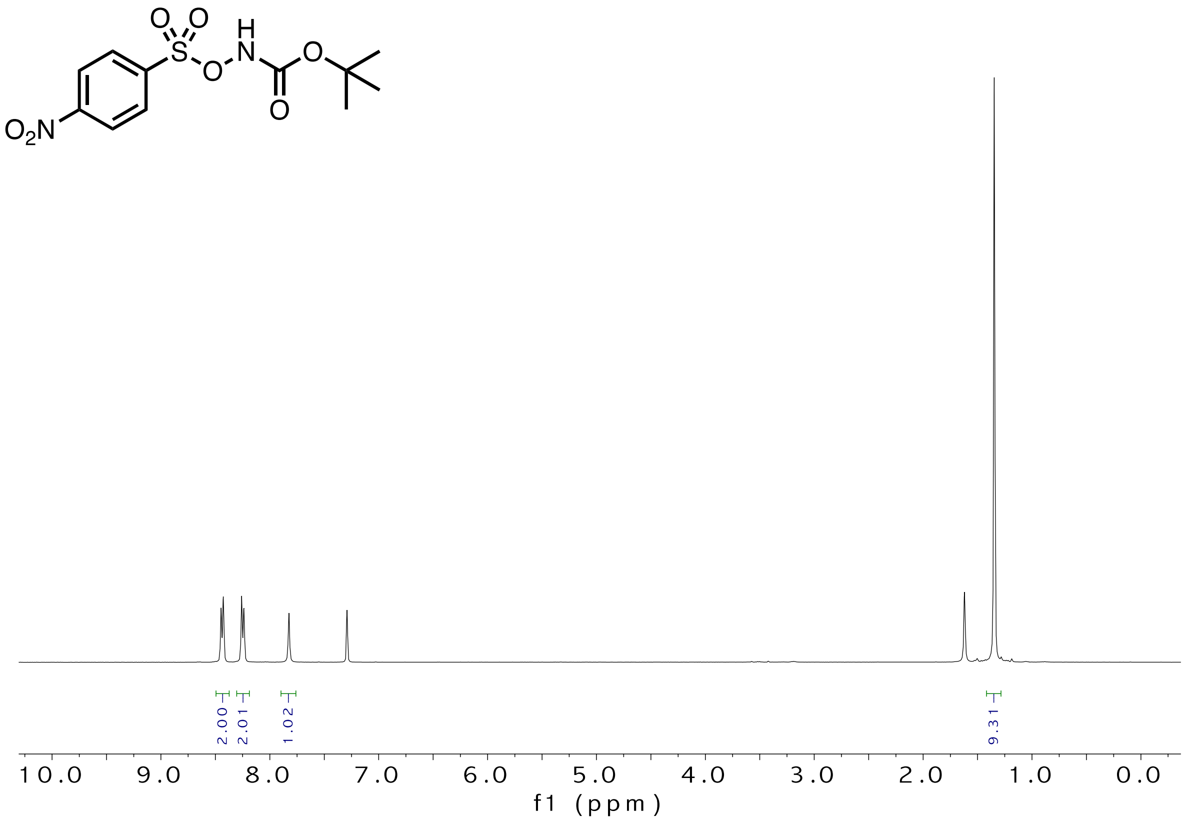

101 MHz, $CDCl_3$

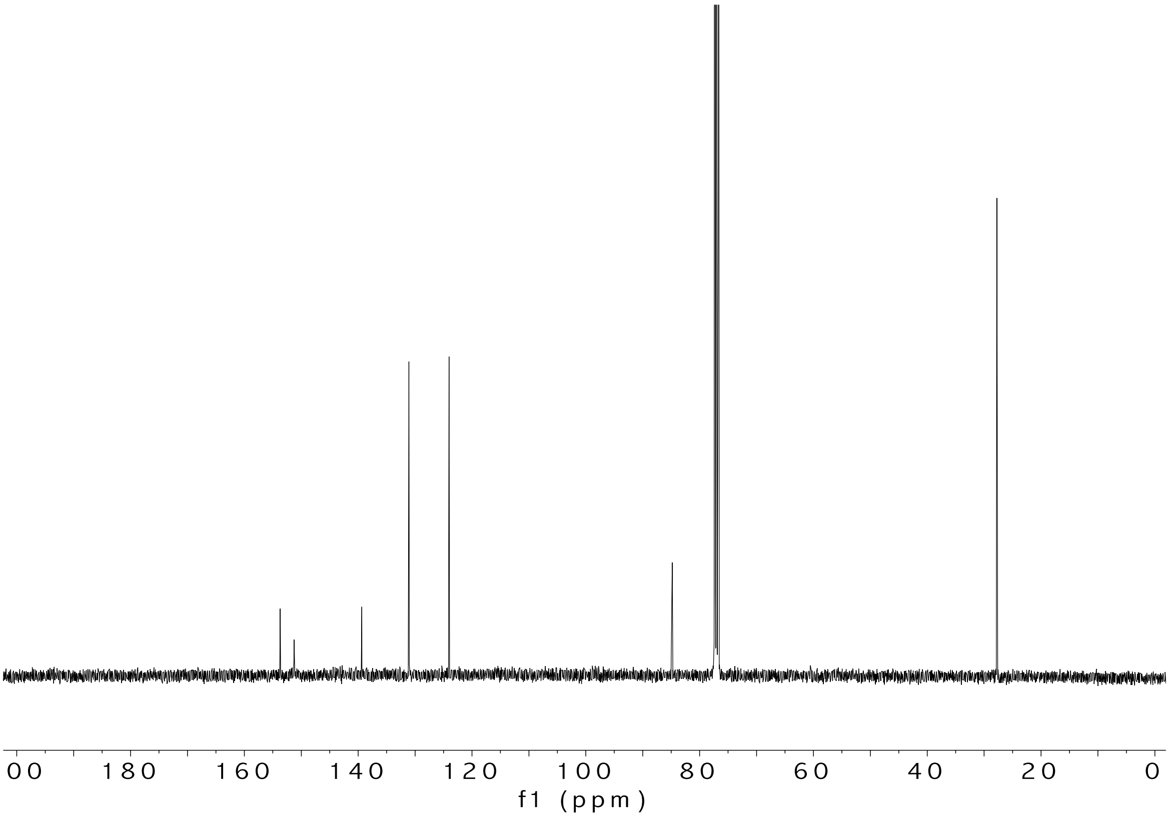

_O_-((4-Nitrophenyl)sulfonyl)hydroxylammonium trifluoromethanesulfonate 1zc

400 MHz, DMSO-d_6

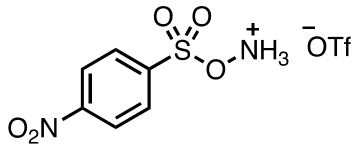

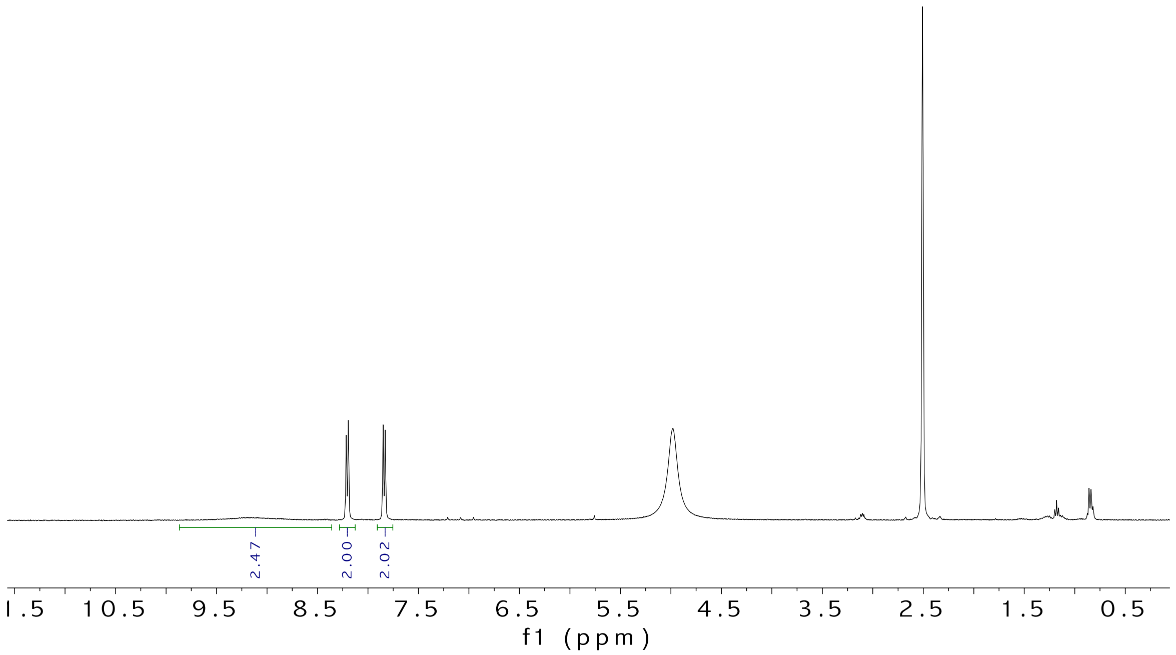

101 MHz, DMSO-d_6

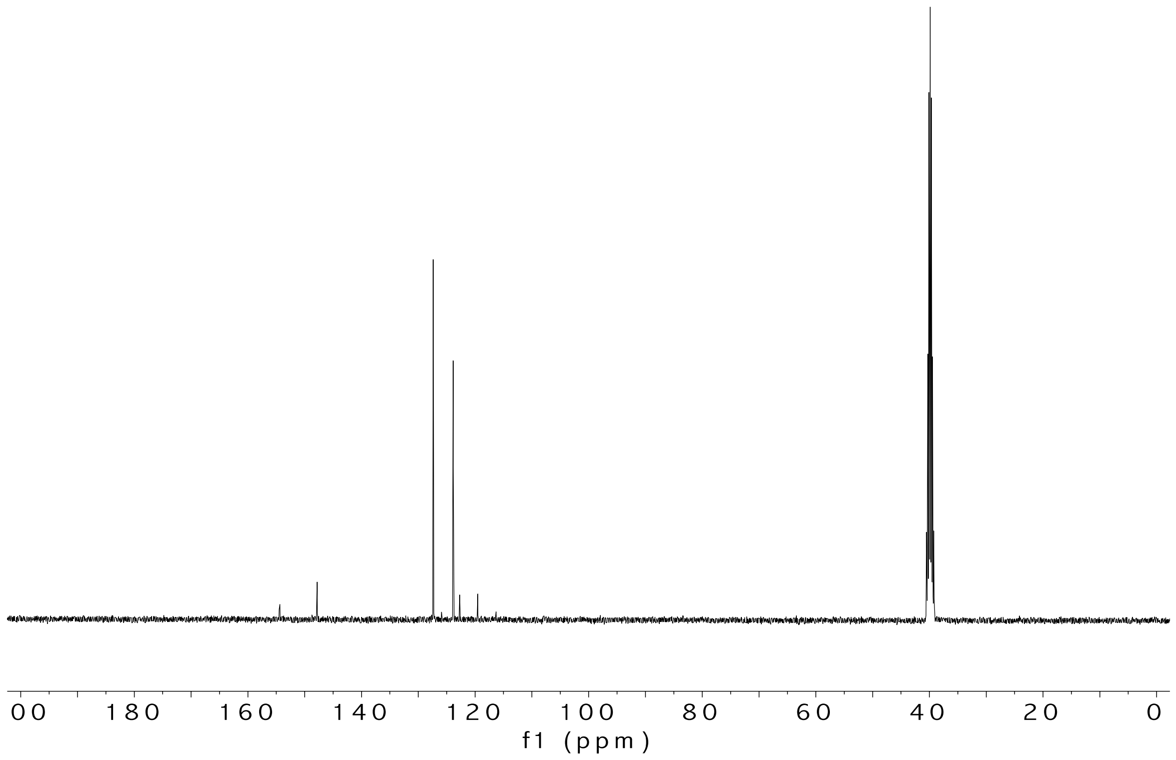

376 MHz, DMSO-d_6

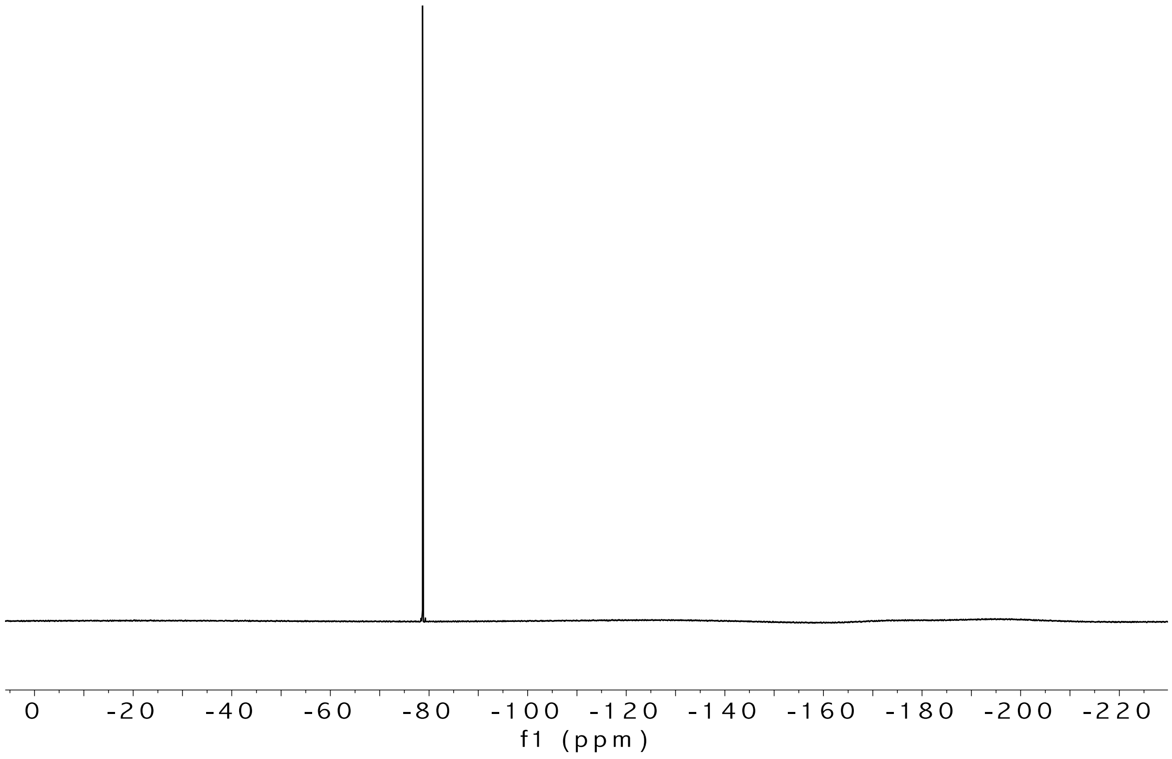

tert-Butyl ((benzylsulfonyl)oxy)carbamate 3a

400 MHz, $CDCl_3$

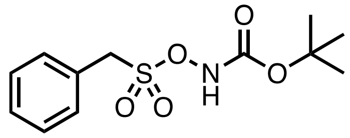

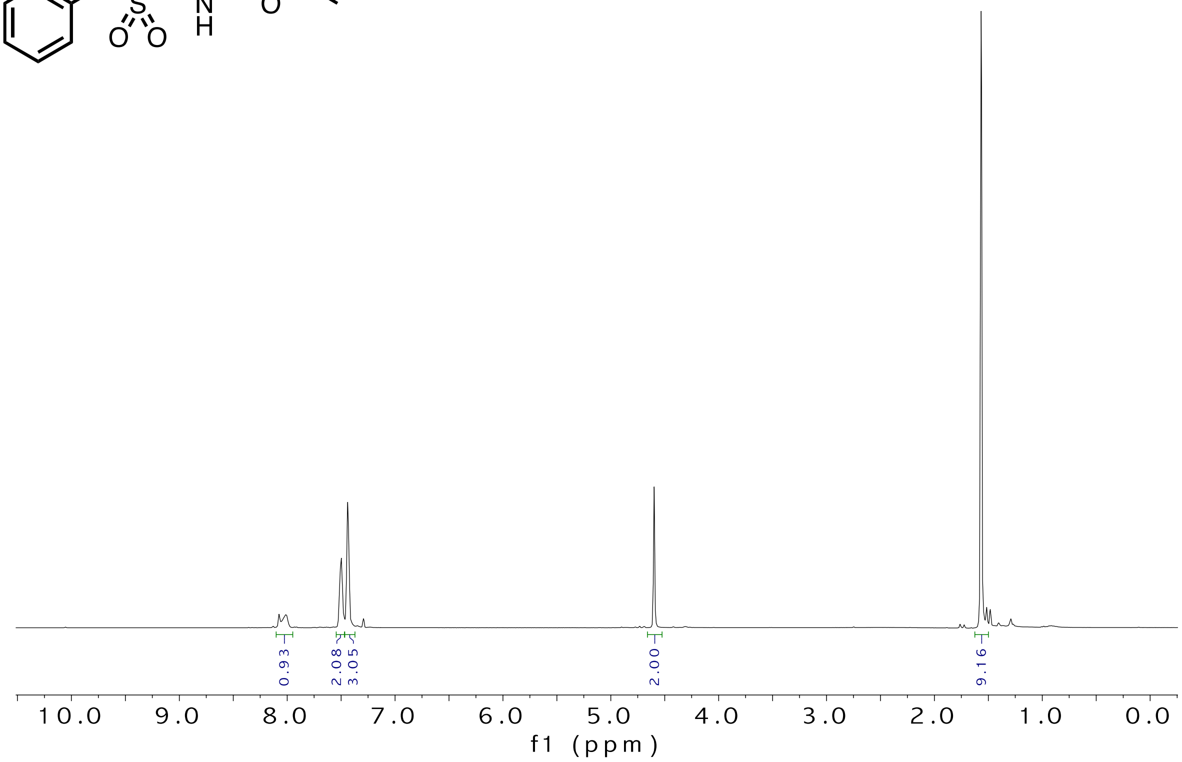

101 MHz, $CDCl_3$

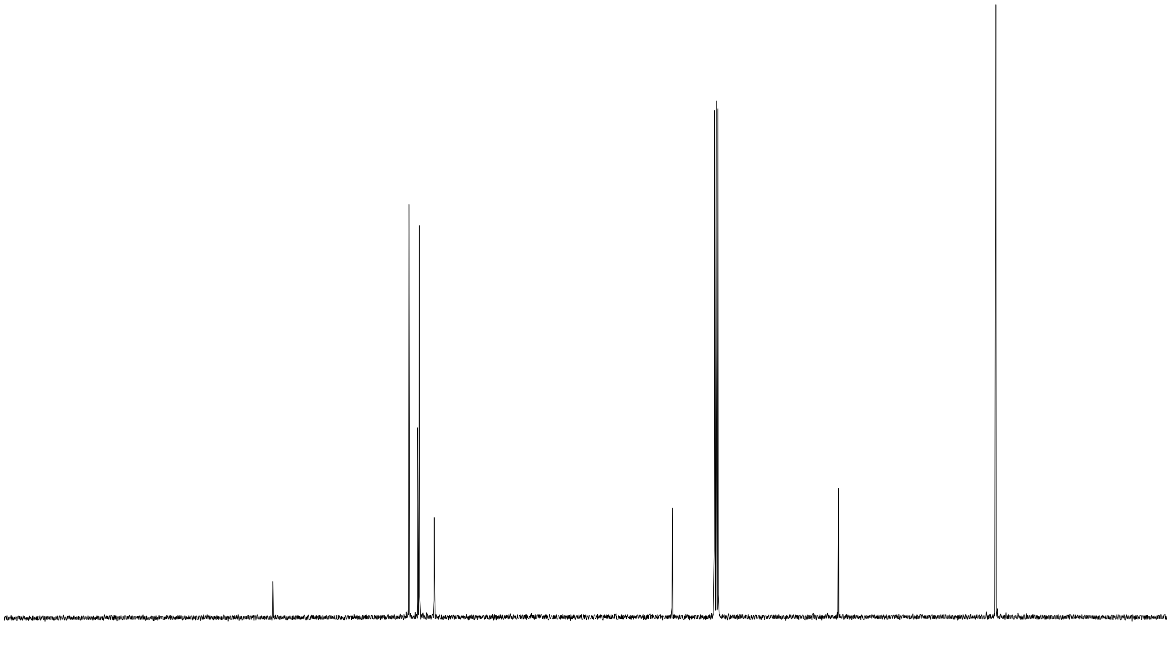

***tert*-Butyl (((4-chlorobenzyl)sulfonyl)oxy)carbamate 3b**

400 MHz, $CDCl_3$

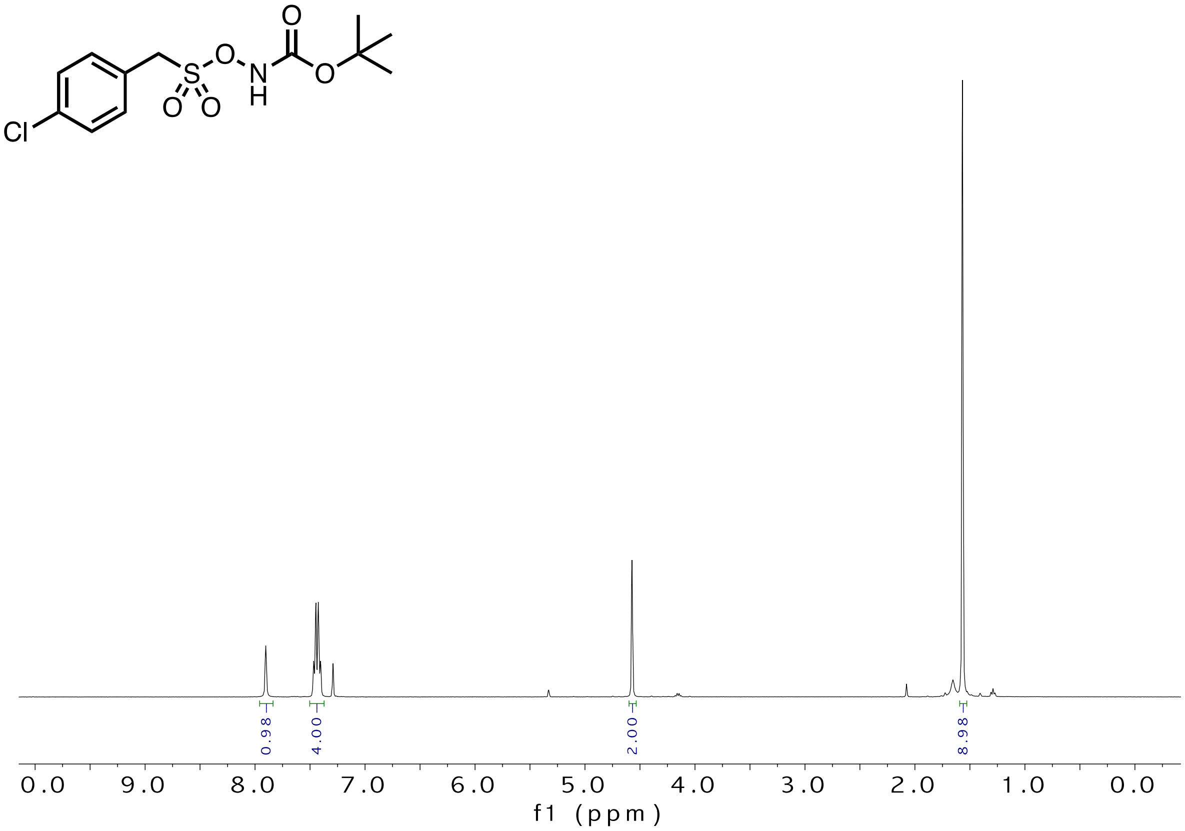

101 MHz, $CDCl_3$

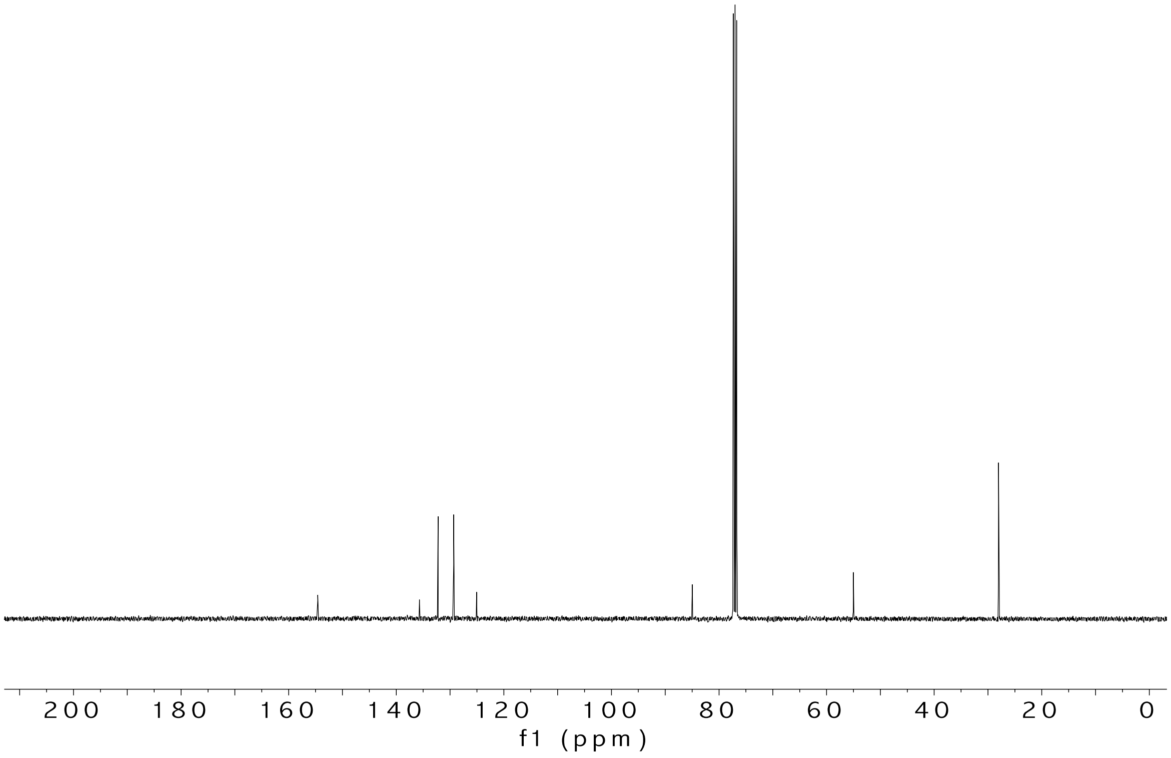

tert-Butyl (((2-chlorobenzyl)sulfonyl)oxy)carbamate 3c

400 MHz, $CDCl_3$

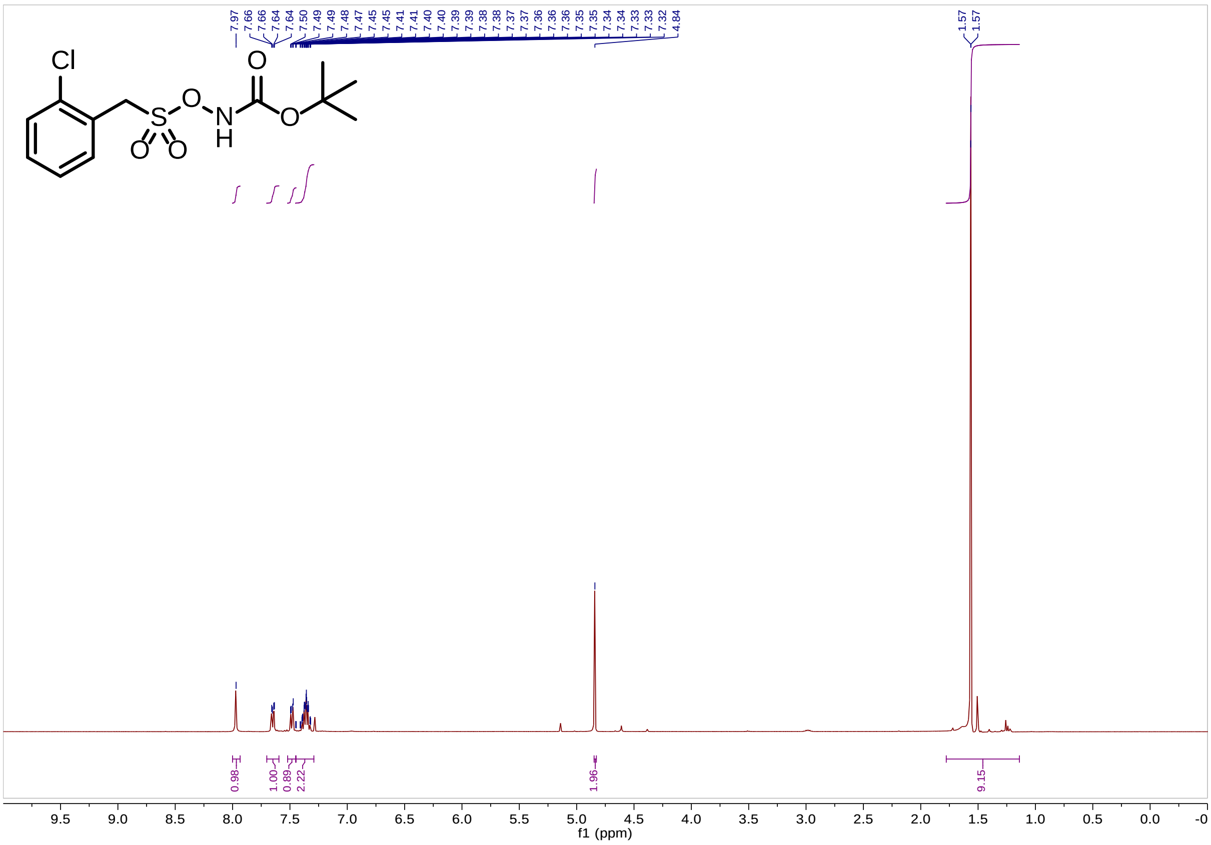

101 MHz, $CDCl_3$

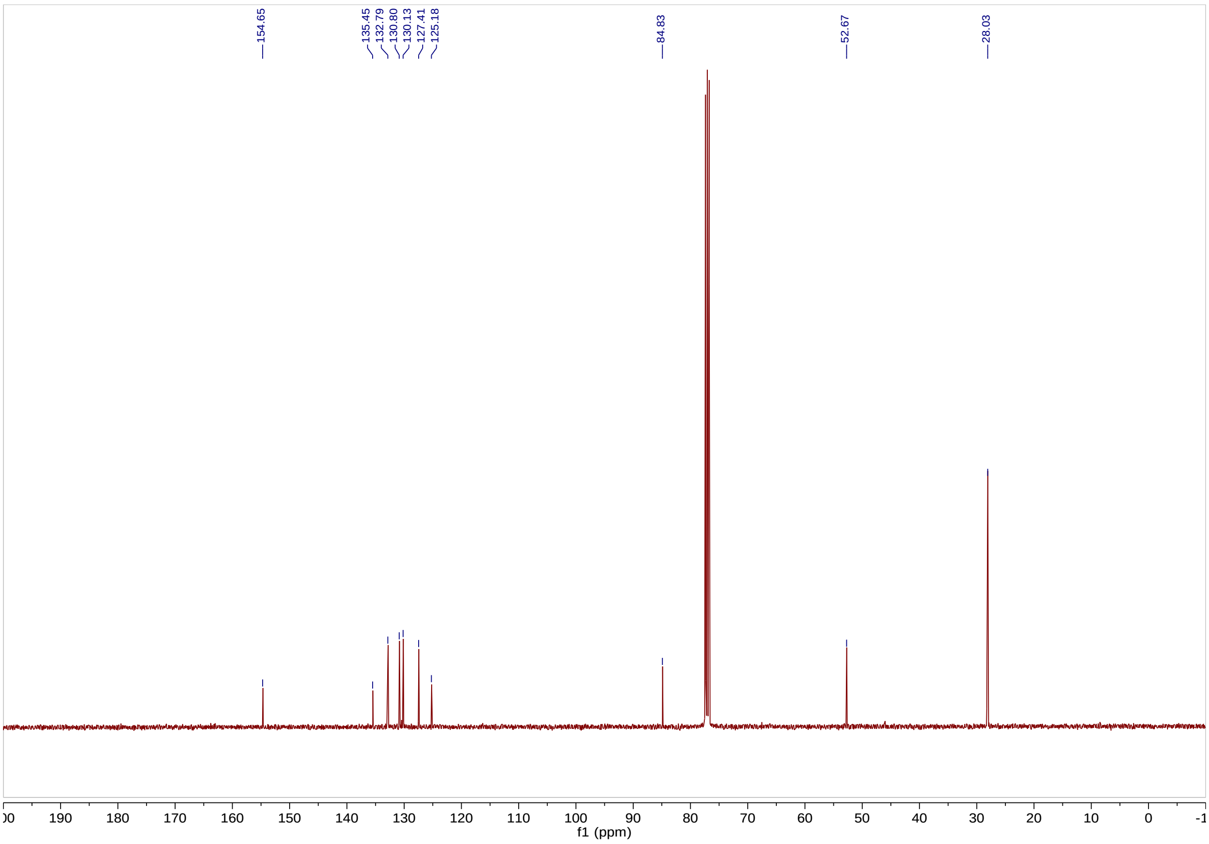

tert-Butyl (((4-methylbenzyl)sulfonyl)oxy)carbamate 3d

500 MHz, $CDCl_3$

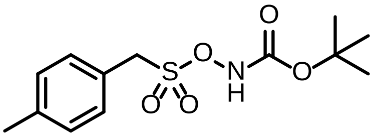

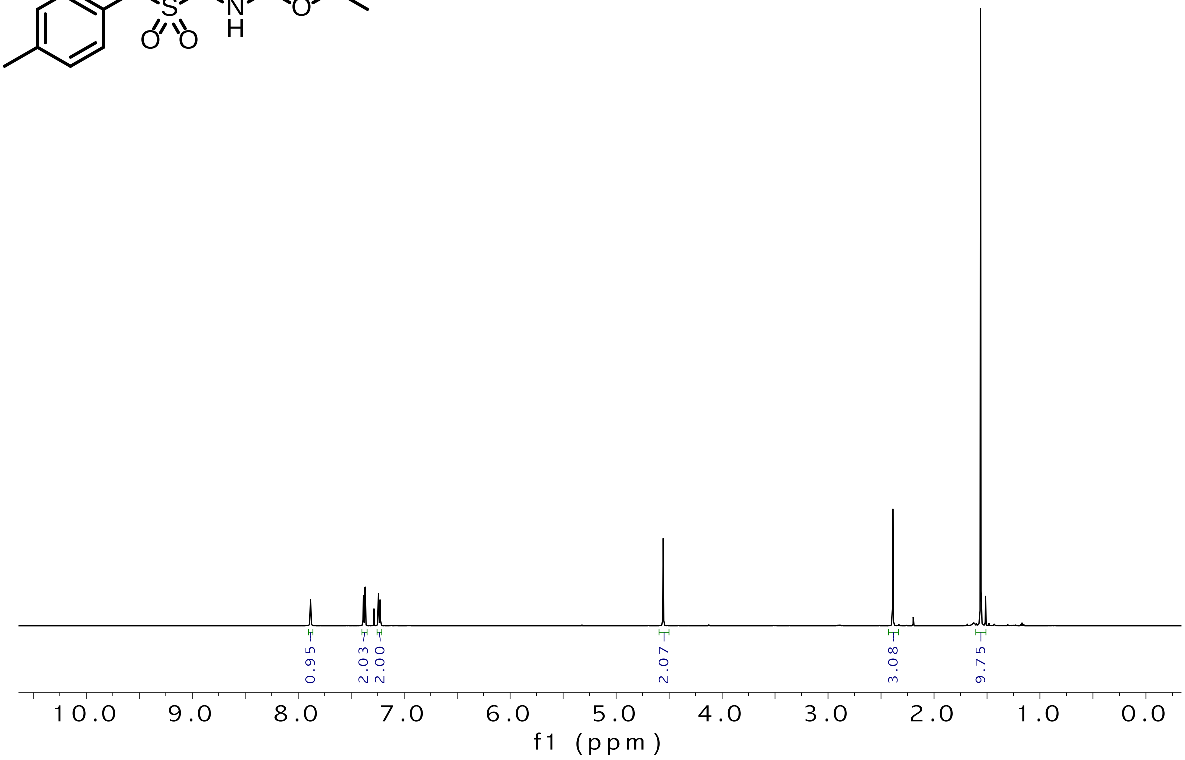

126 MHz, $CDCl_3$

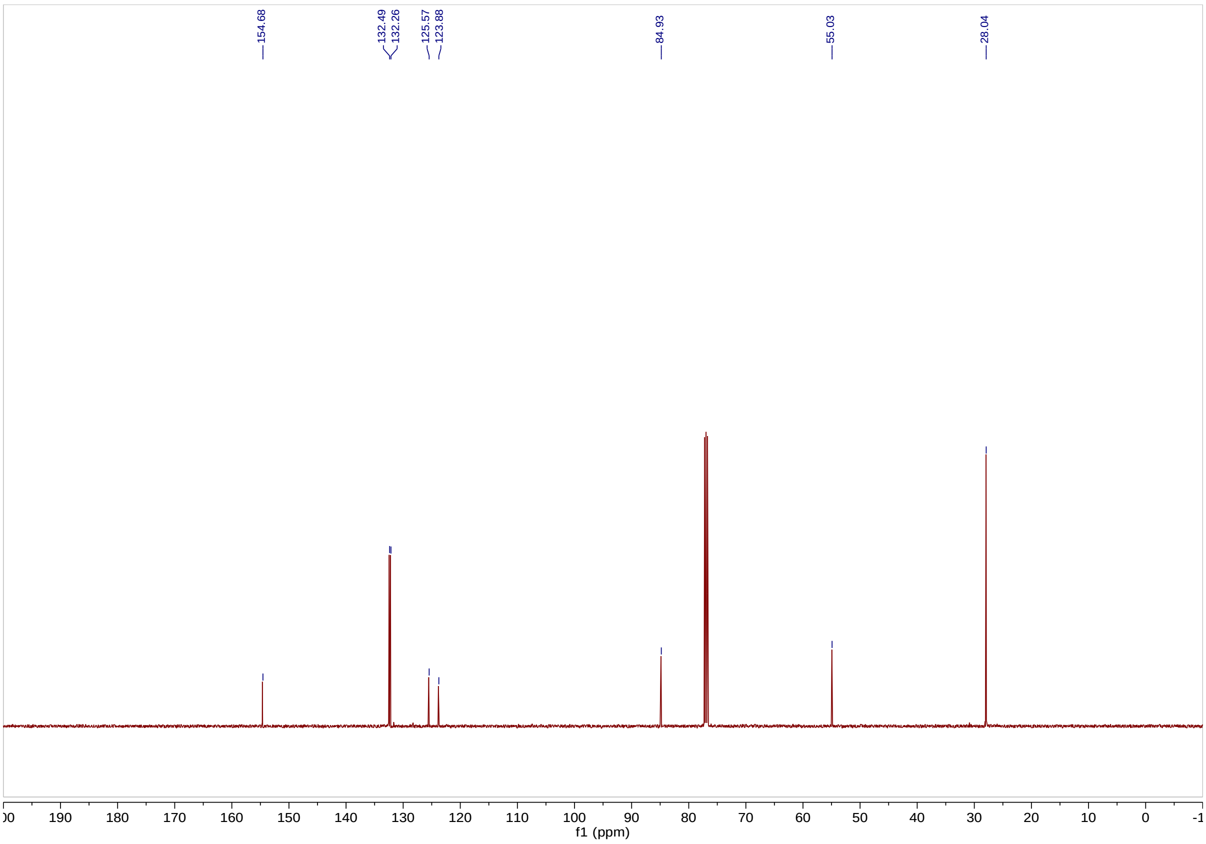

tert-Butyl (((3,5-dimethoxybenzyl)sulfonyl)oxy)carbamate 3e

400 MHz, $CDCl_3$

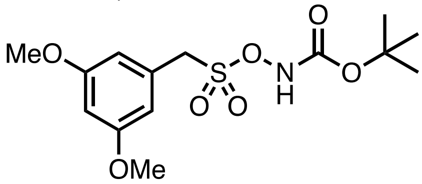

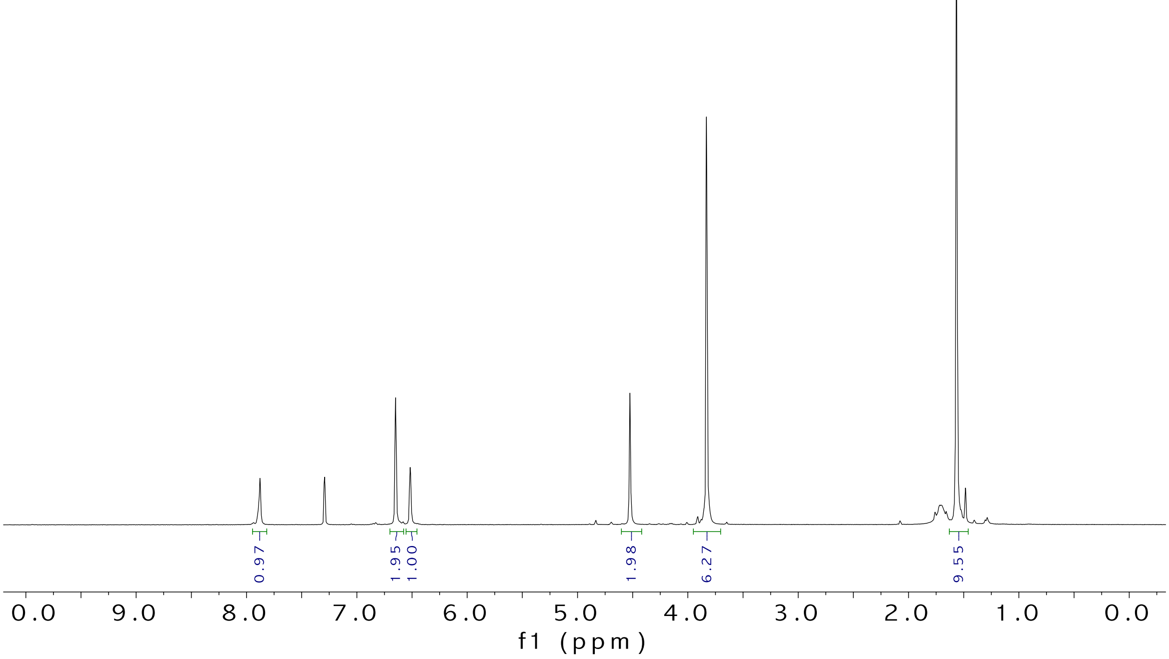

101 MHz, $CDCl_3$

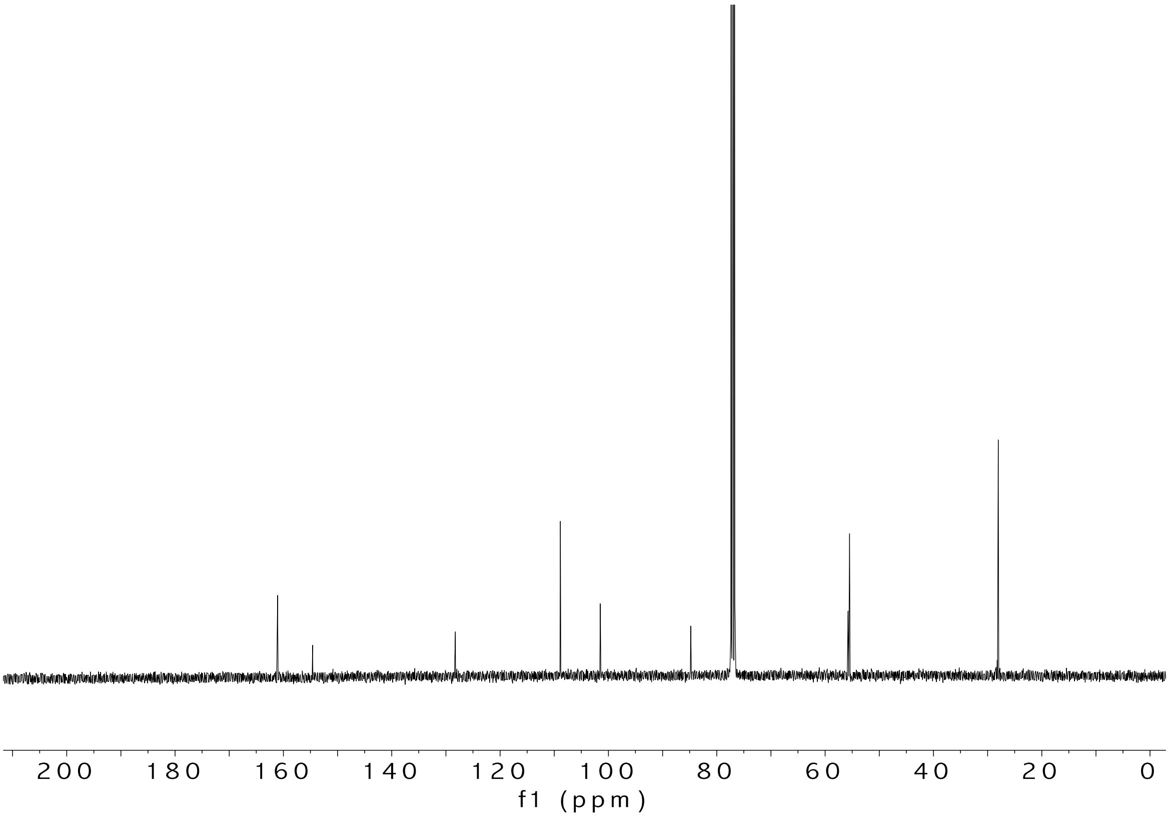

tert-Butyl (((4-bromobenzyl)sulfonyl)oxy)carbamate 3f

500 MHz, $CDCl_3$

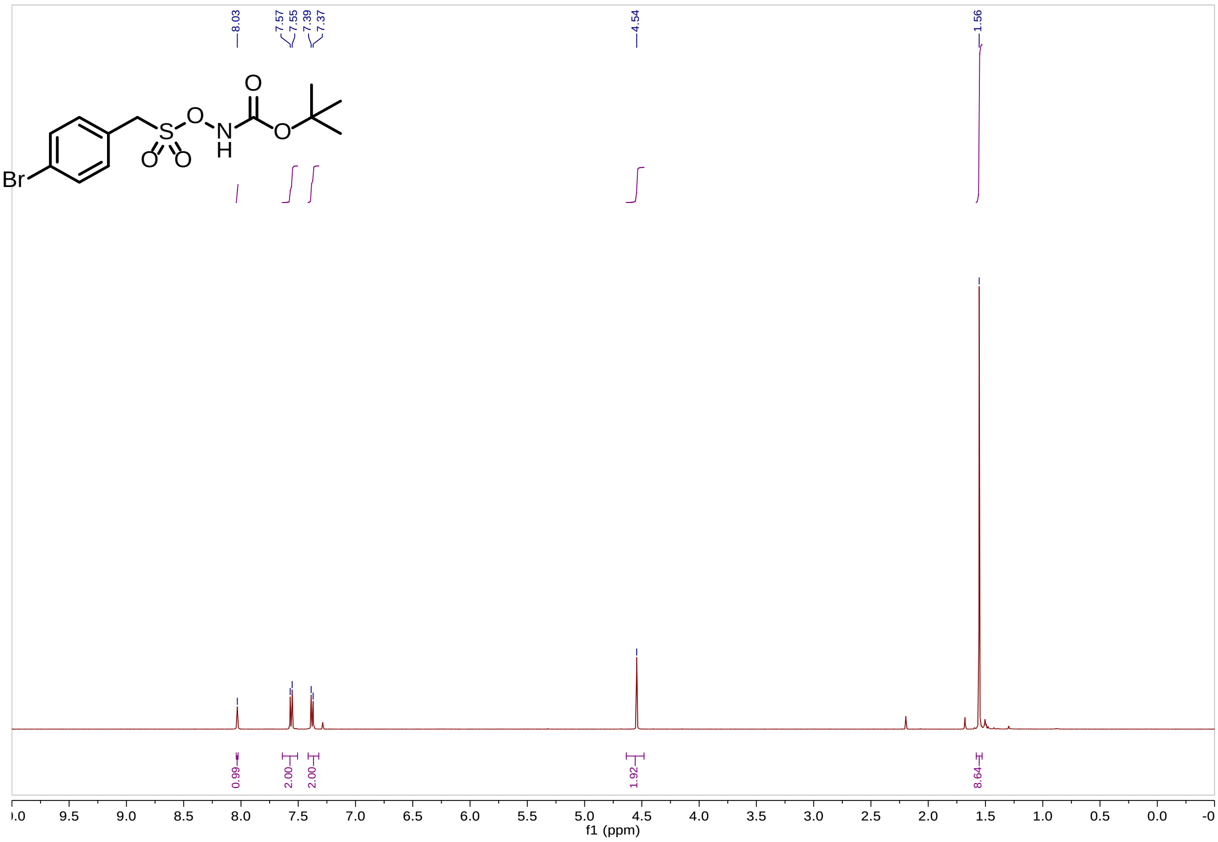

126 MHz, $CDCl_3$

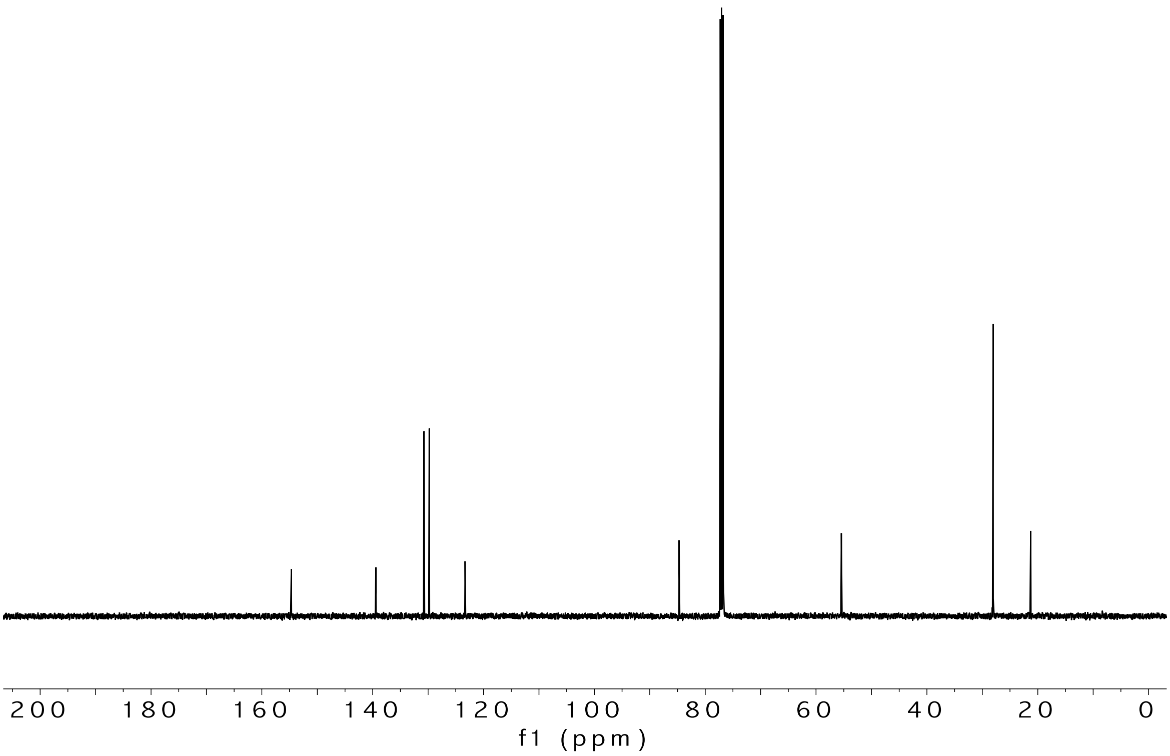

tert-Butyl (((3-bromobenzyl)sulfonyl)oxy)carbamate 3g

400 MHz, $CDCl_3$

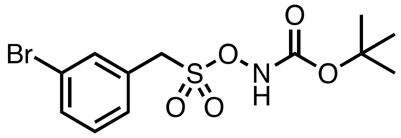

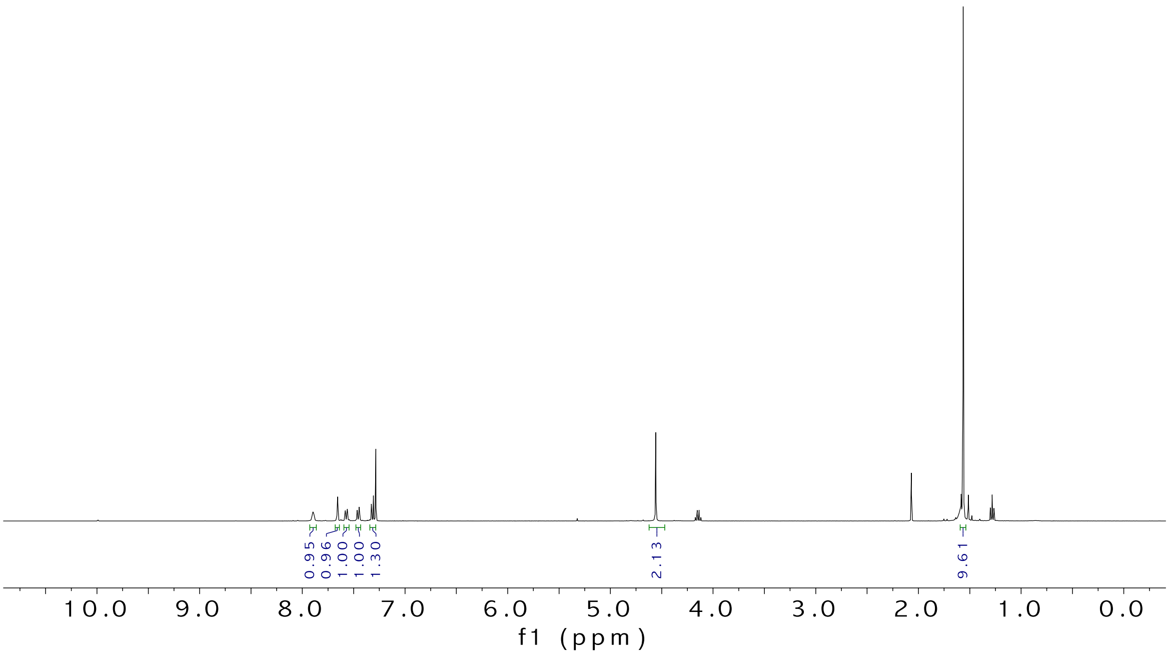

101 MHz, $CDCl_3$

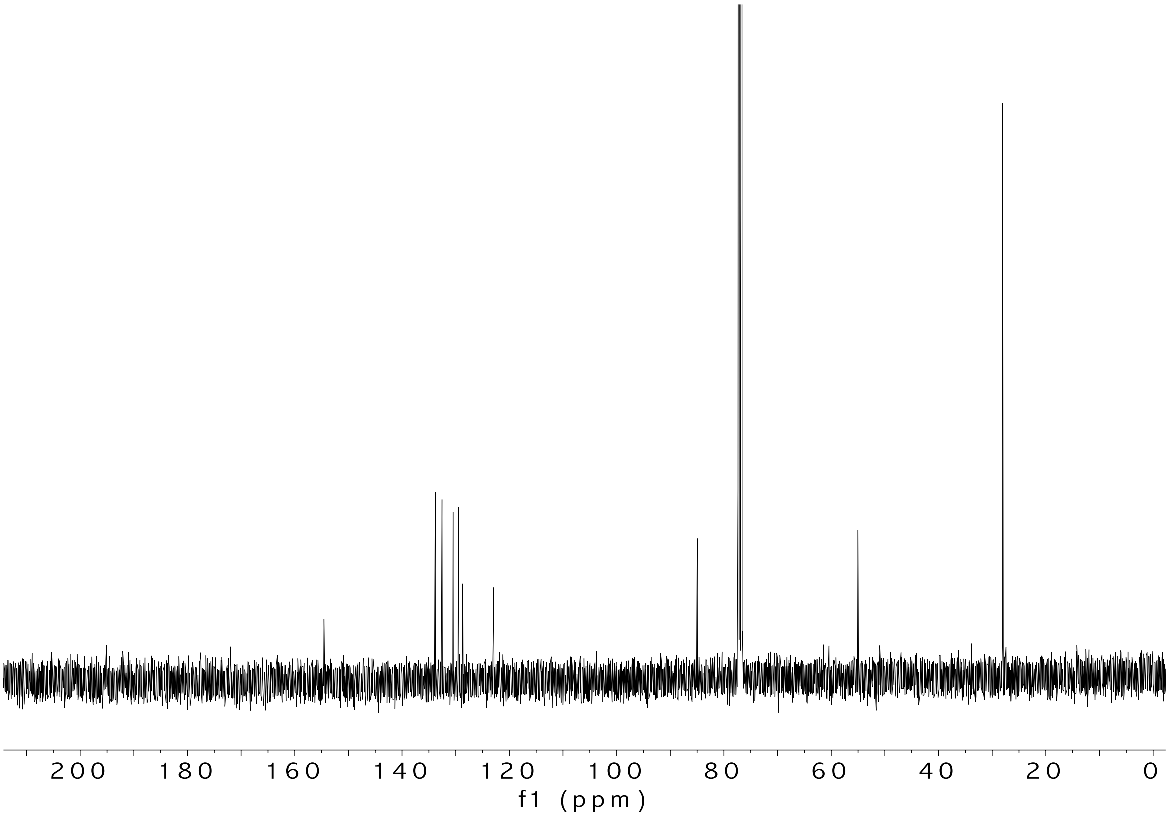

tert-Butyl (((4-fluorobenzyl)sulfonyl)oxy)carbamate 3h

400 MHz, $CDCl_3$

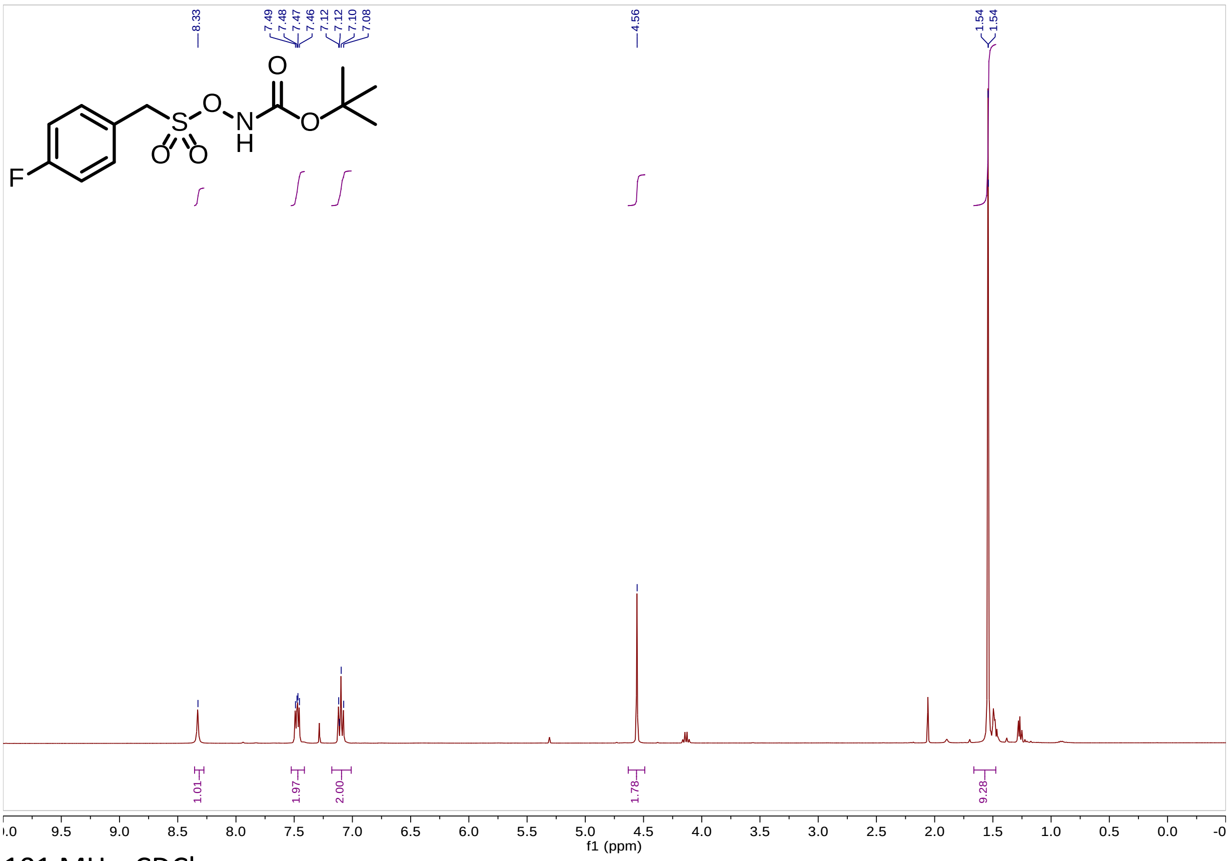

101 MHz, $CDCl_3$

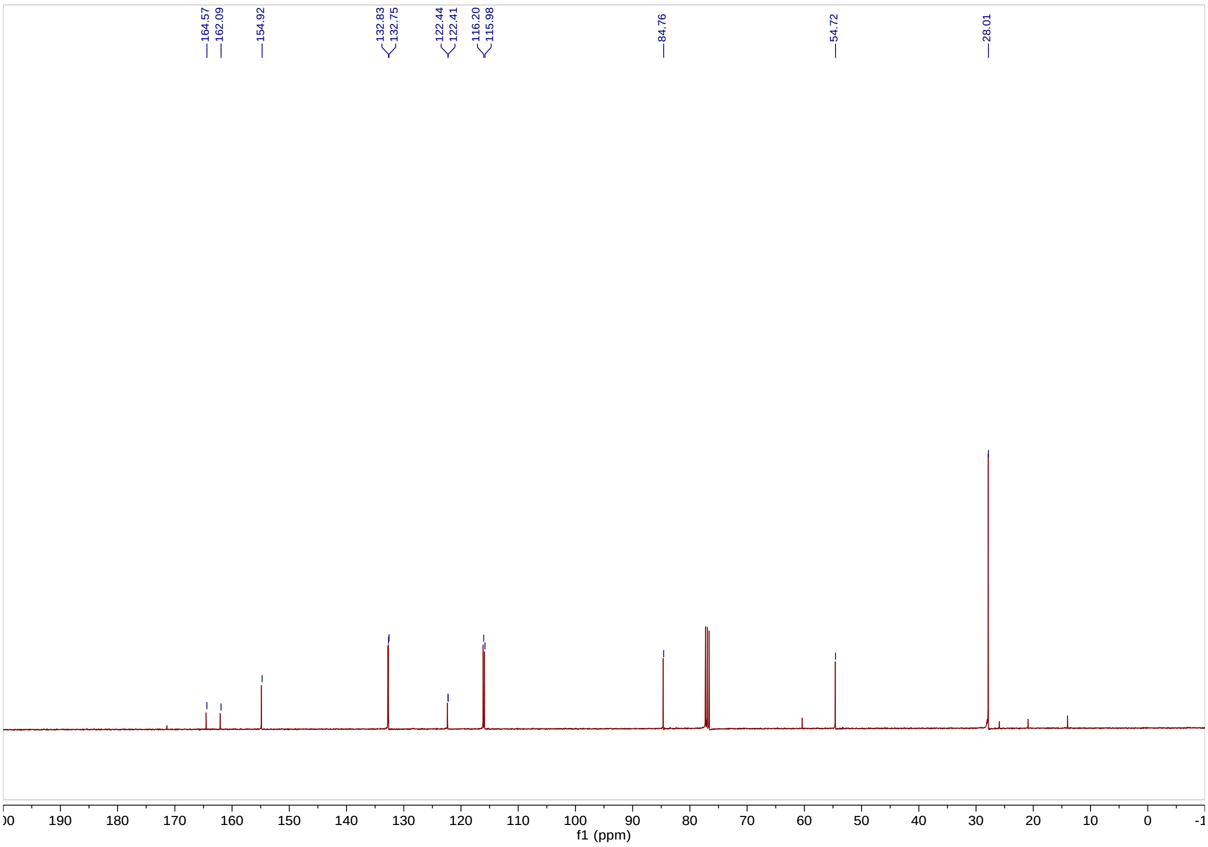

376 MHz, $CDCl_3$

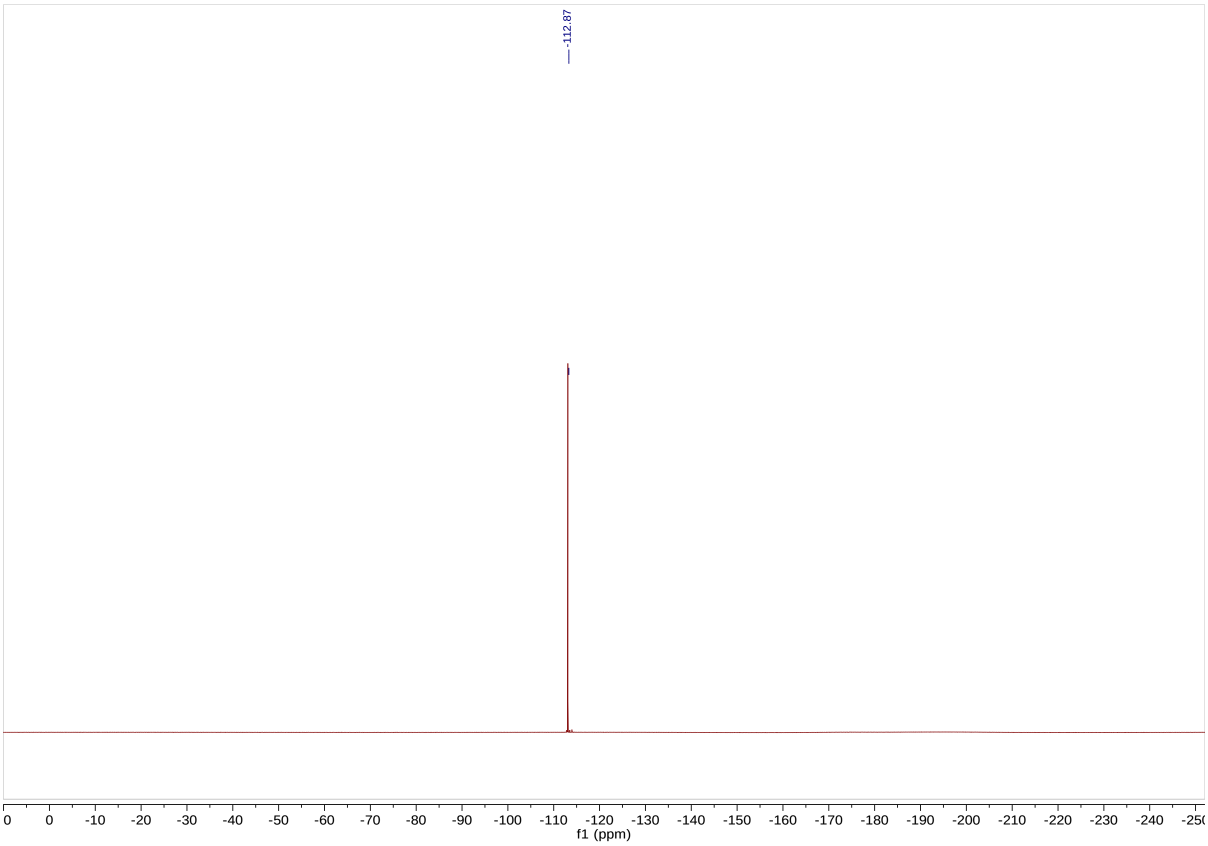

tert-Butyl (((4-(trifluoromethyl)benzyl)sulfonyl)oxy)carbamate 3i

400 MHz, $CDCl_3$

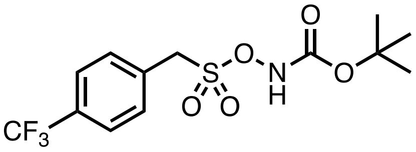

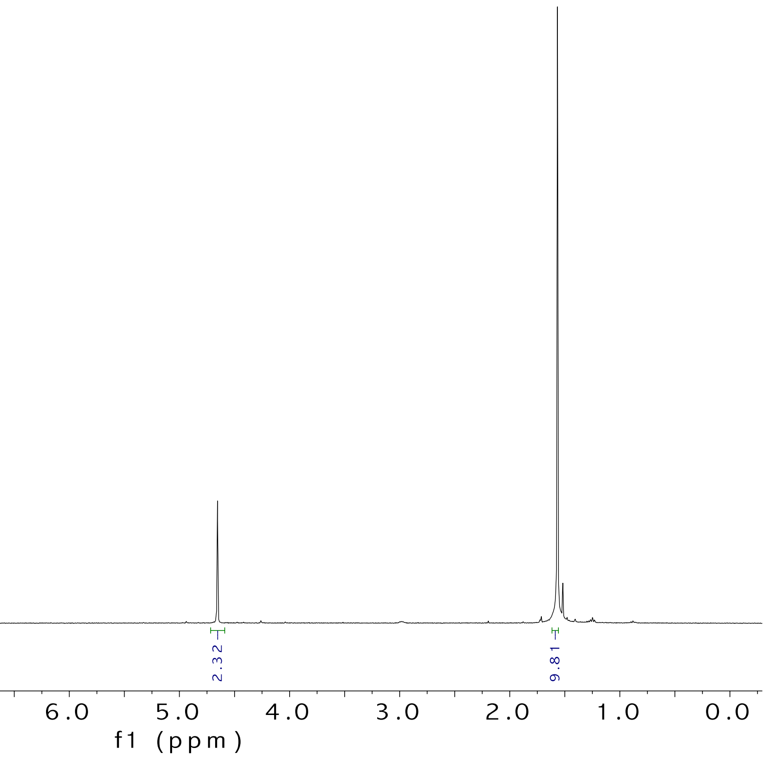

126 MHz, $CDCl_3$

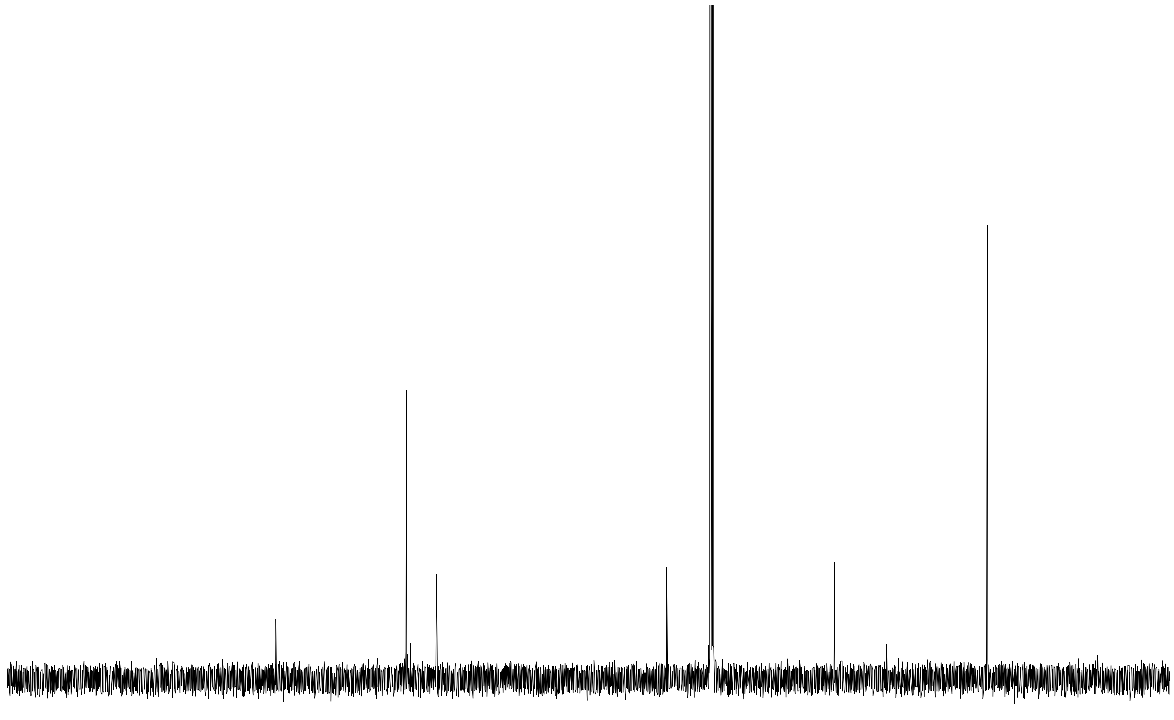

376 MHz, $CDCl_3$

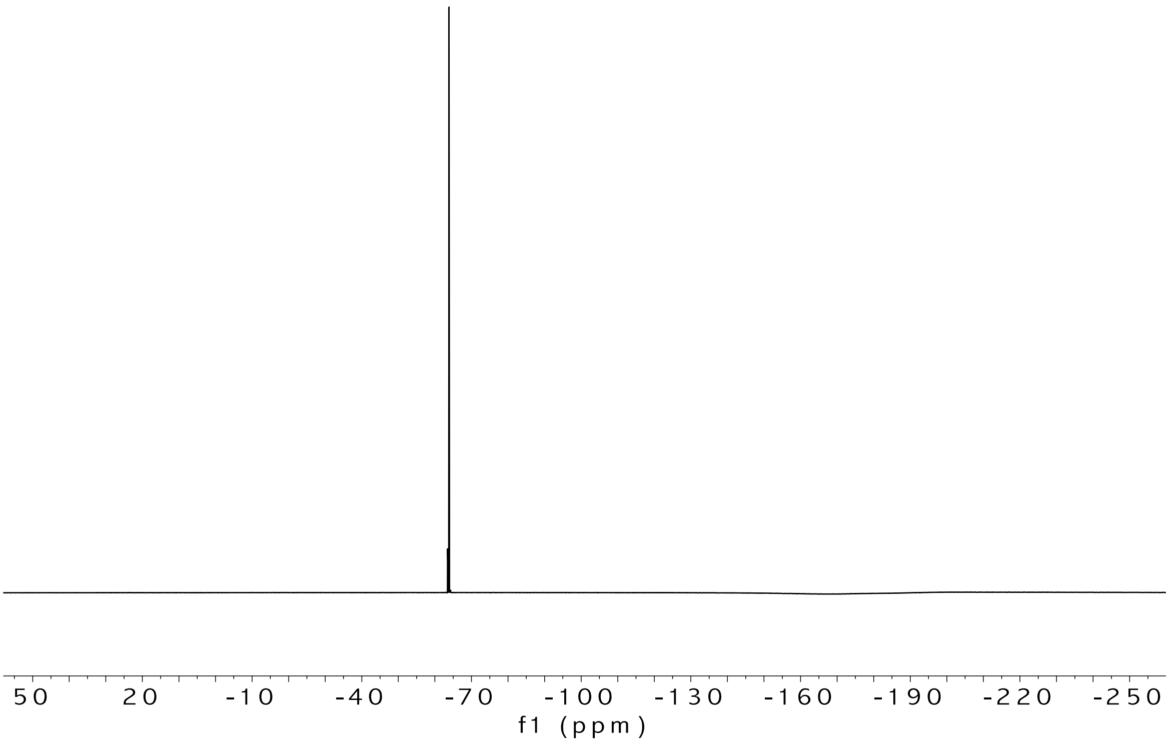

tert-Butyl methyl((phenylsulfonyl)oxy)carbamate 5a

400 MHz, $CDCl_3$

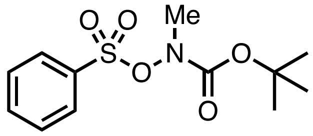

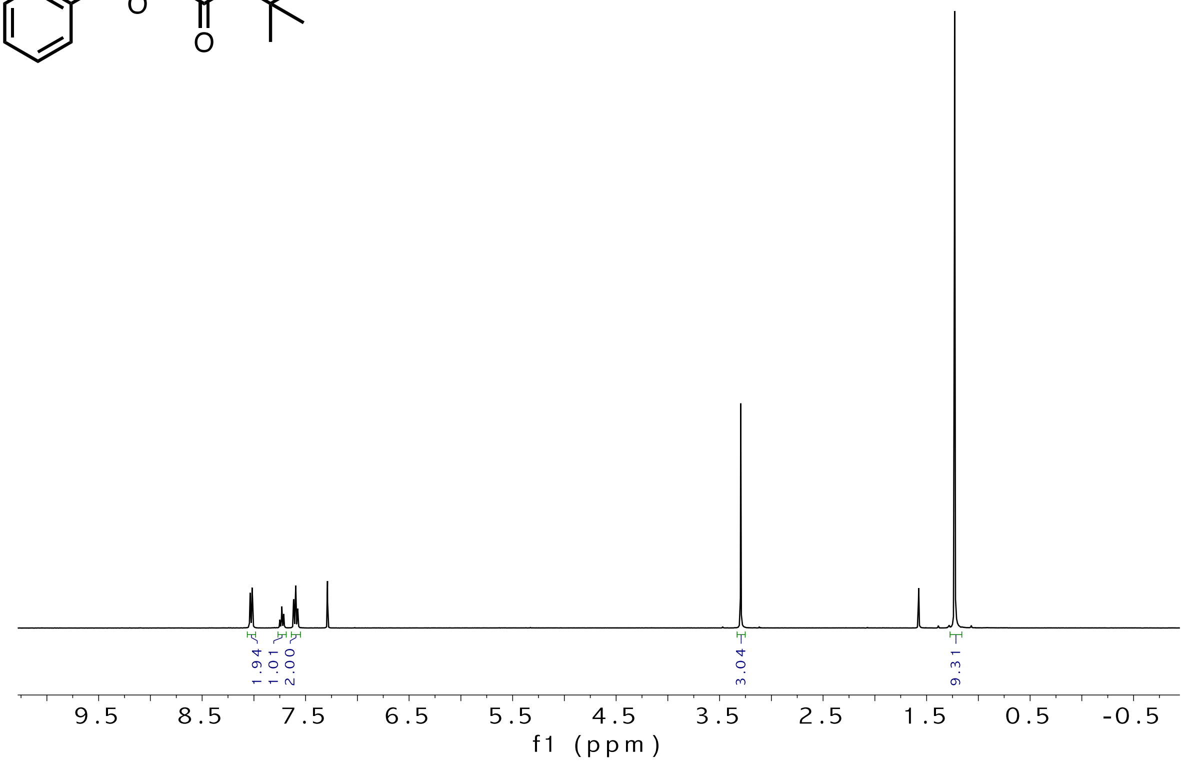

101 MHz, $CDCl_3$

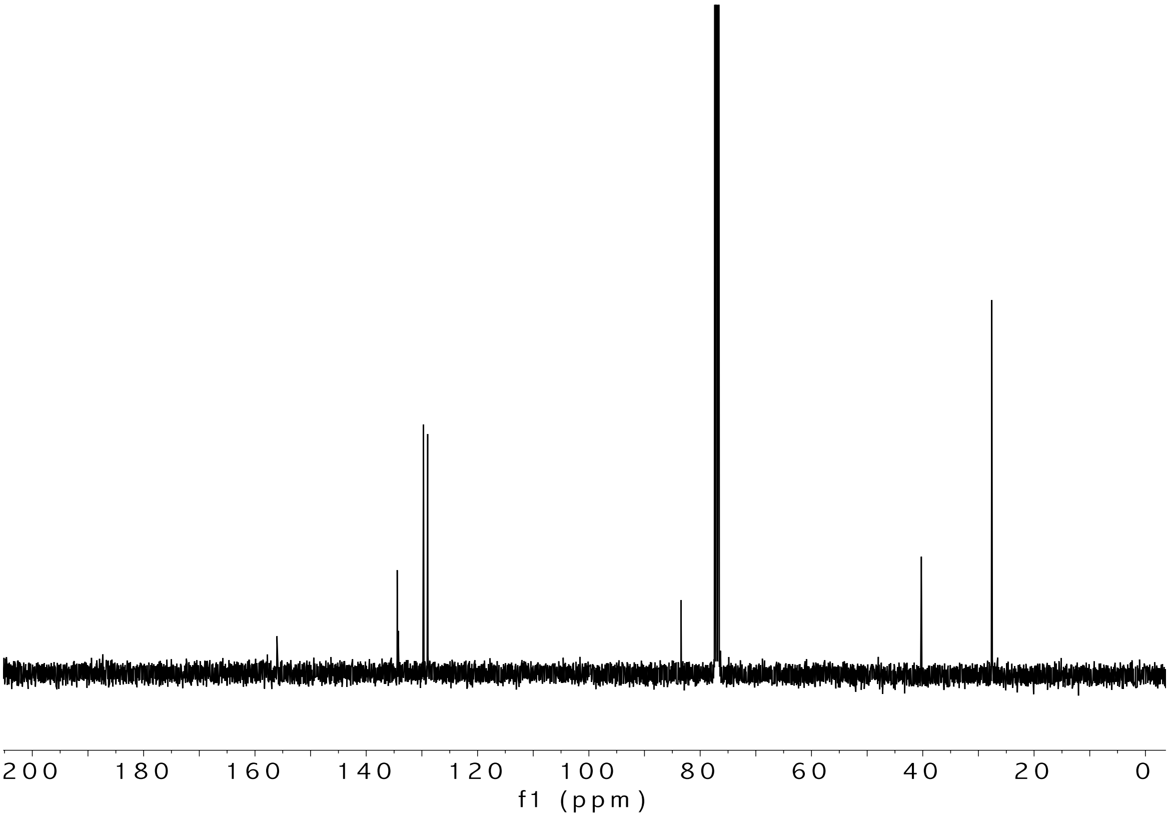

tert-Butyl methyl(tosyloxy)carbamate

400 MHz, $CDCl_3$

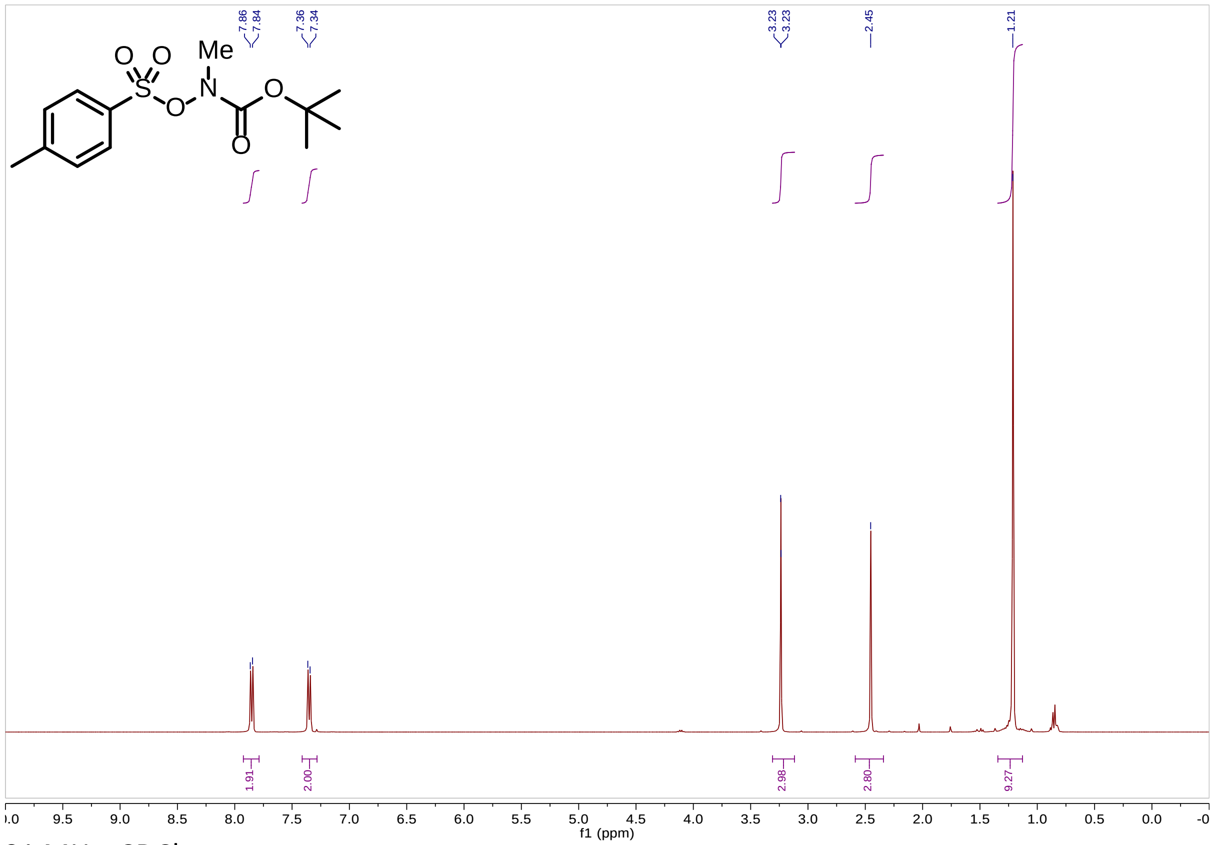

101 MHz, $CDCl_3$

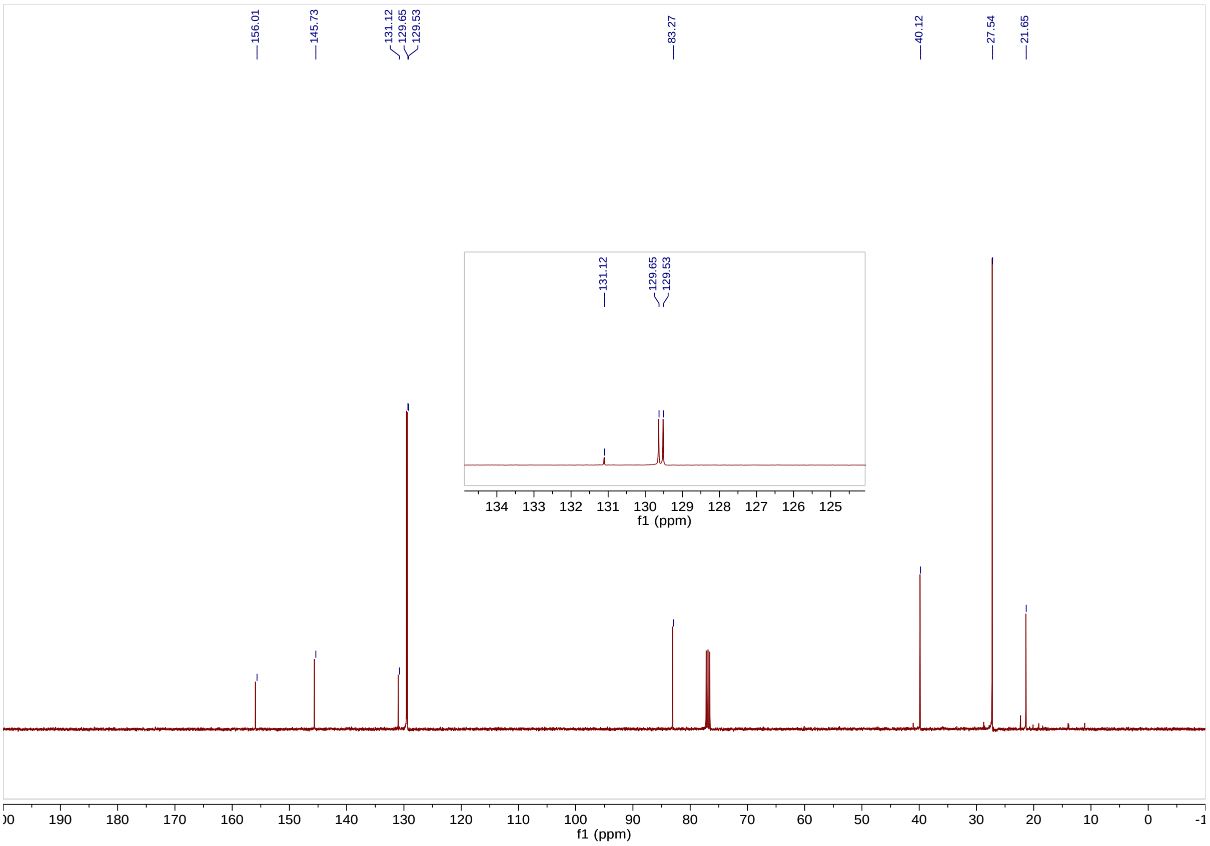

2-Aminobenzenesulfonic acid 2a

400 MHz, DMSO-d_6

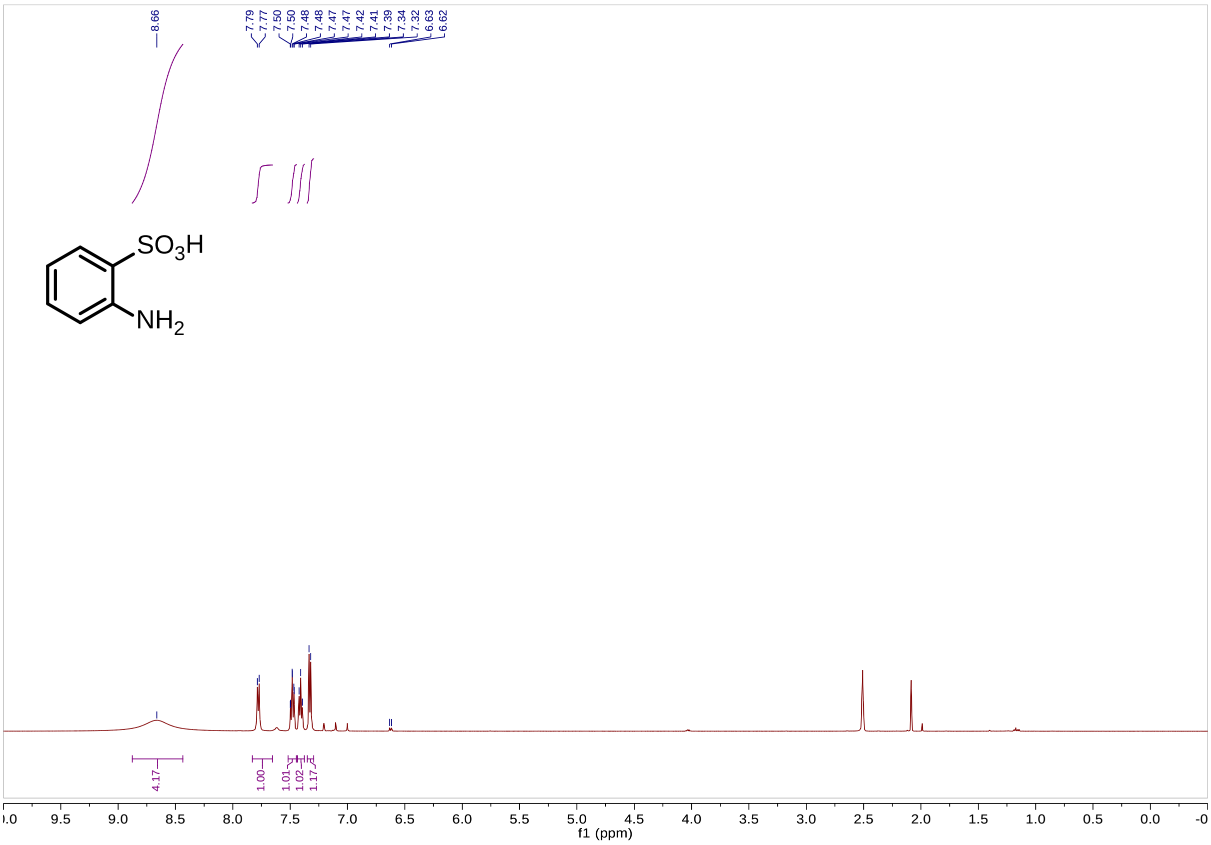

101 MHz, DMSO-d_6

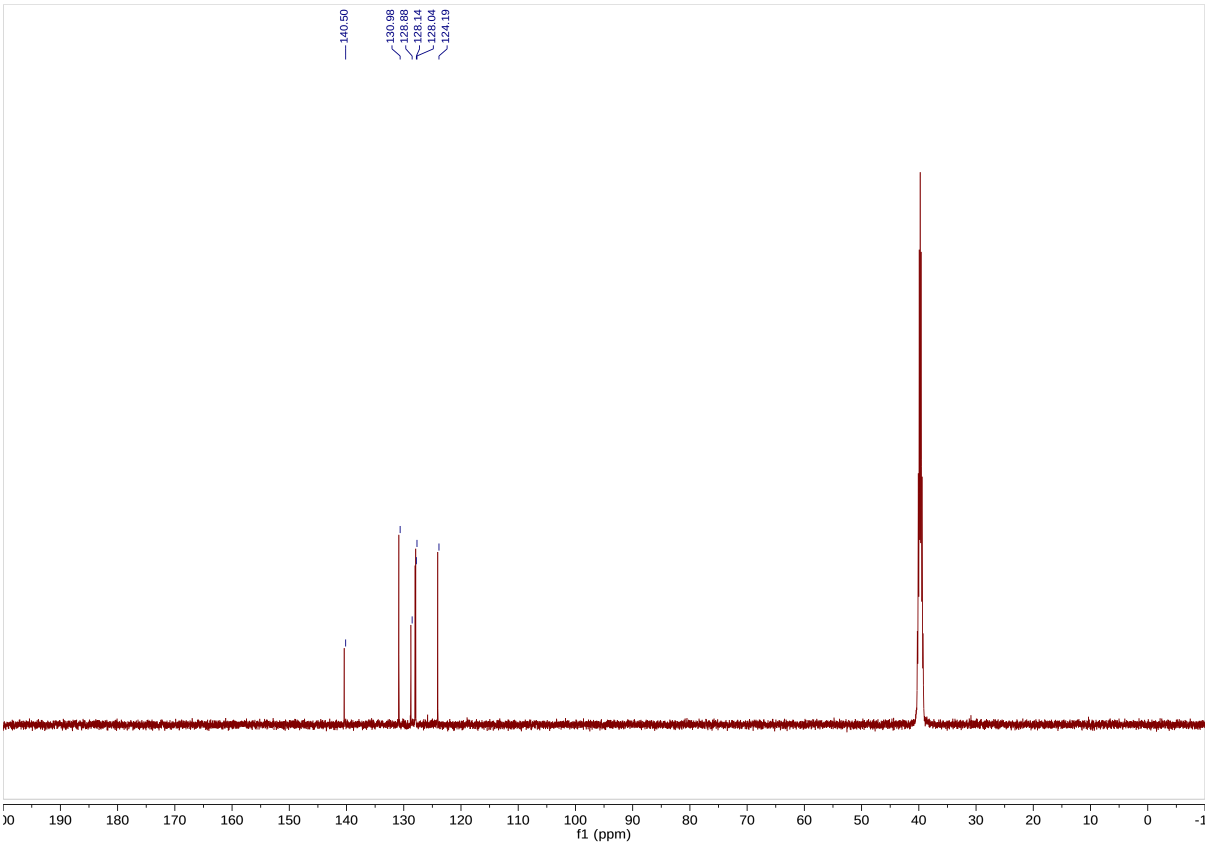

2-Amino-4-methylbenzenesulfonic acid 2b

400 MHz, DMSO-d_6

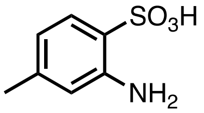

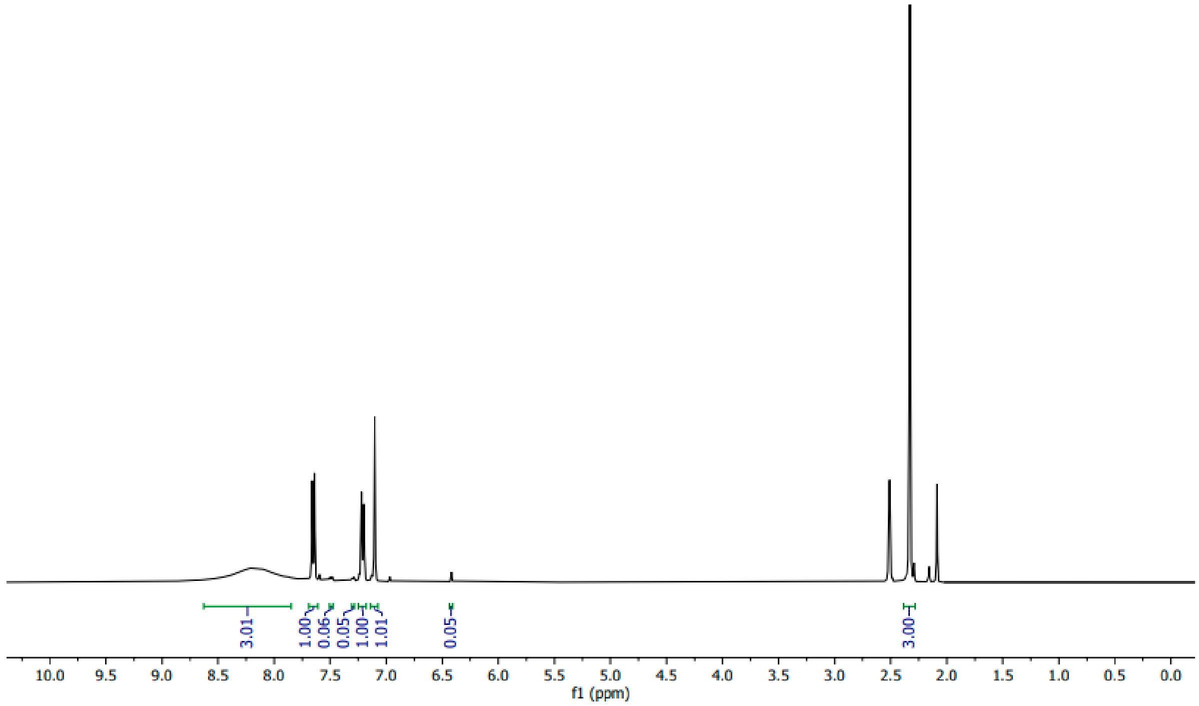

101 MHz, DMSO-d_6

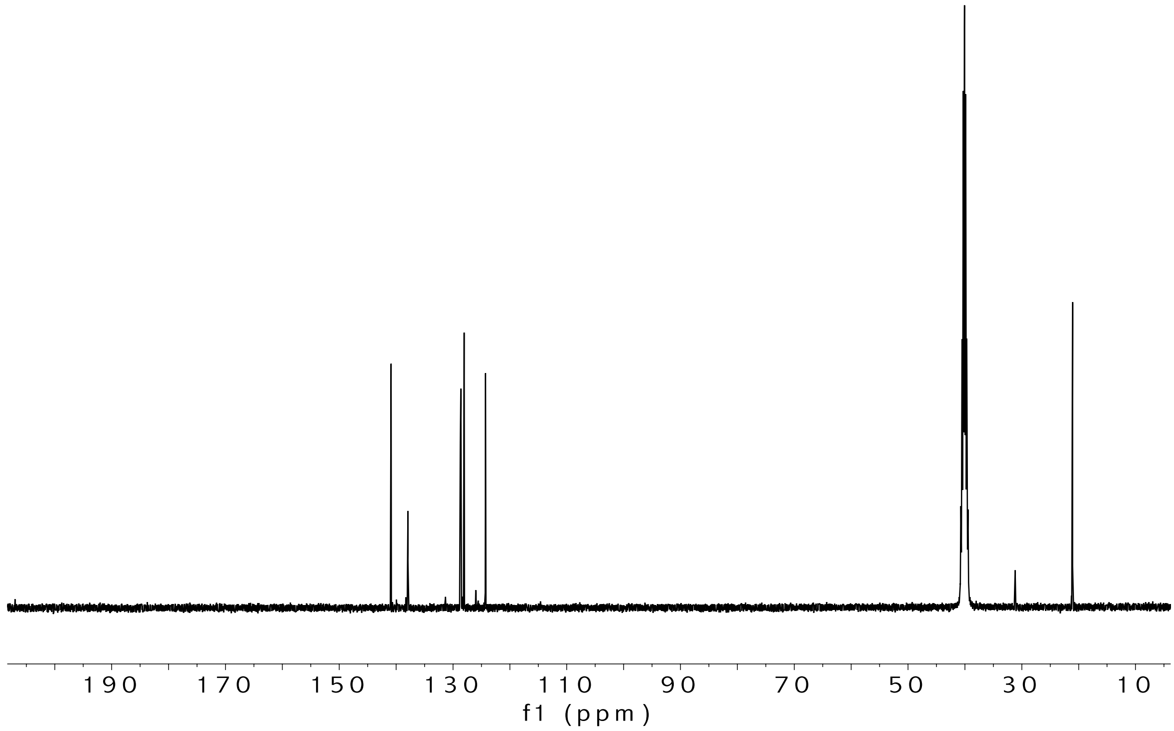

2-Amino-6-methylbenzenesulfonic acid 2c

500 MHz, DMSO-d_6

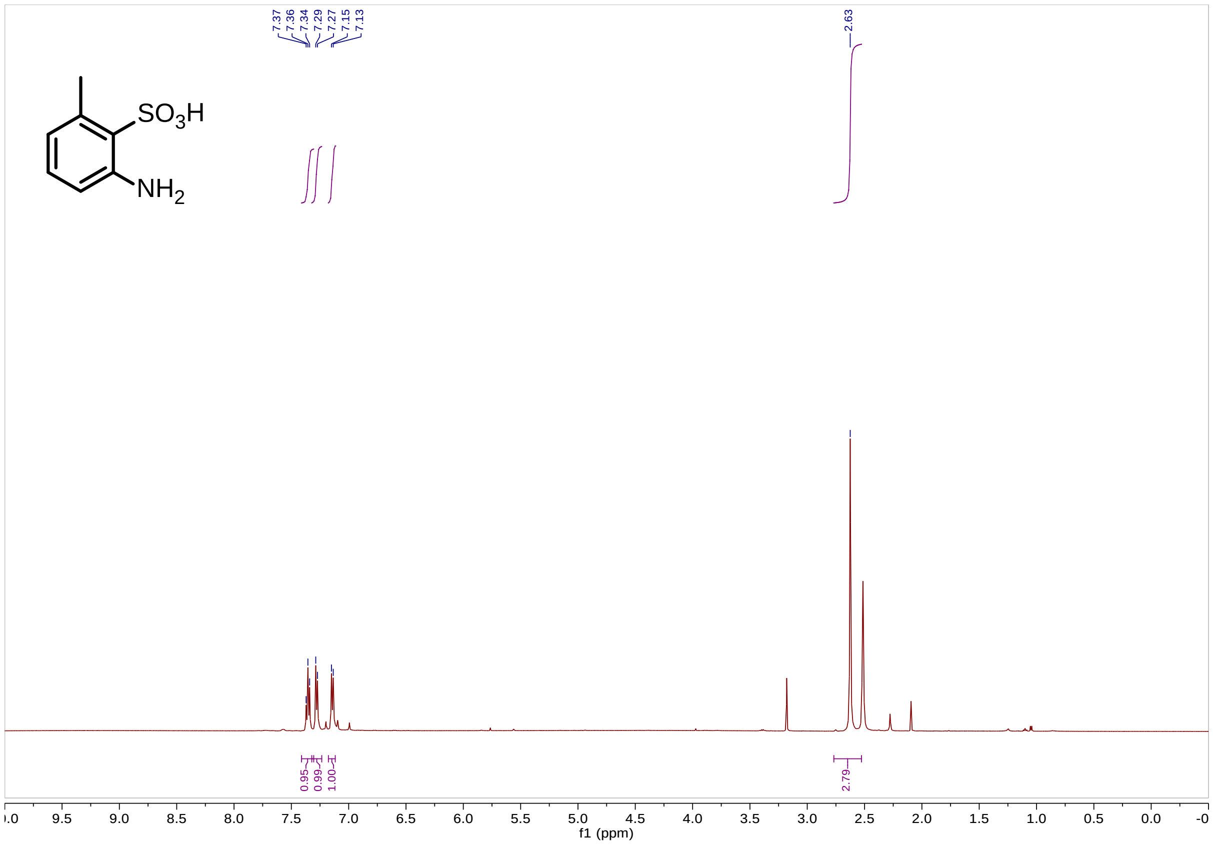

126 MHz, DMSO-d_6

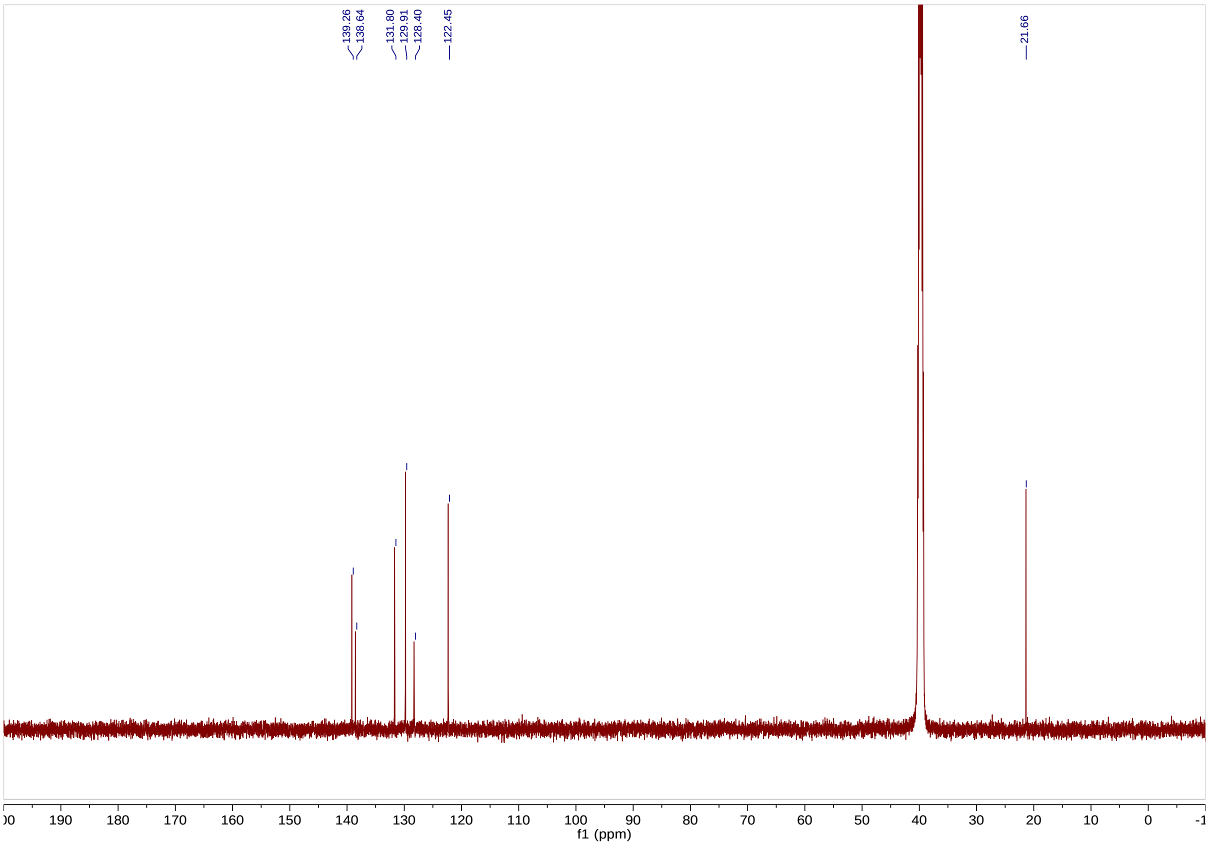

2-Amino-5-methylbenzenesulfonic acid and 2-amino-3-methylbenzenesulfonic acid 2d

400 MHz, DMSO-d_6

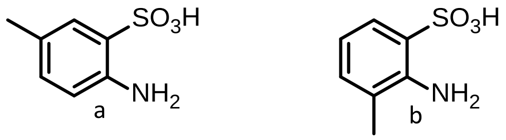

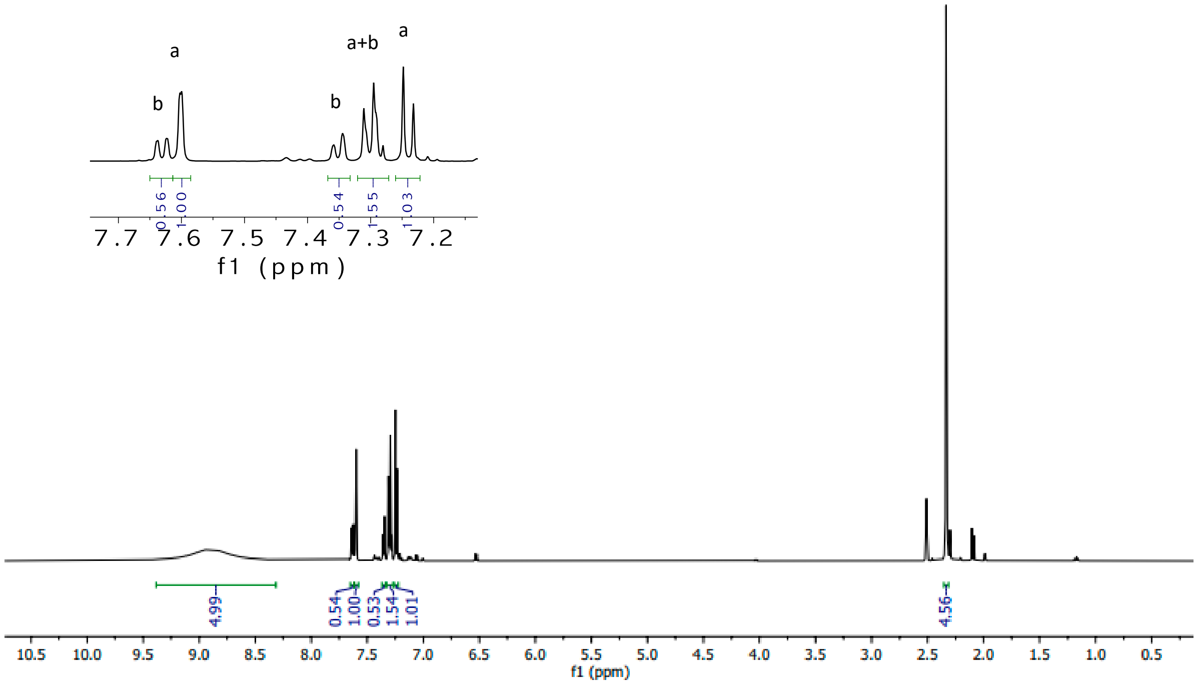

101 MHz, DMSO-d_6

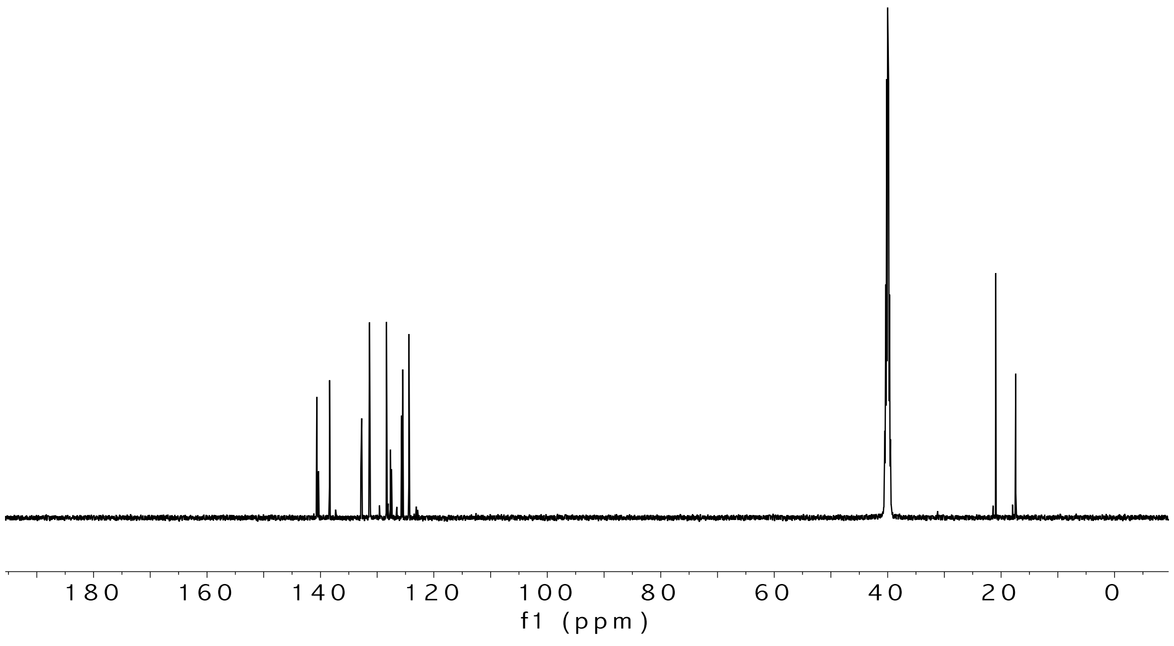

2-Amino-4,5-dimethylbenzenesulfonic acid and 2-amino-3,4-dimethylbenzenesulfonic acid 2e

500 MHz, DMSO-d_6

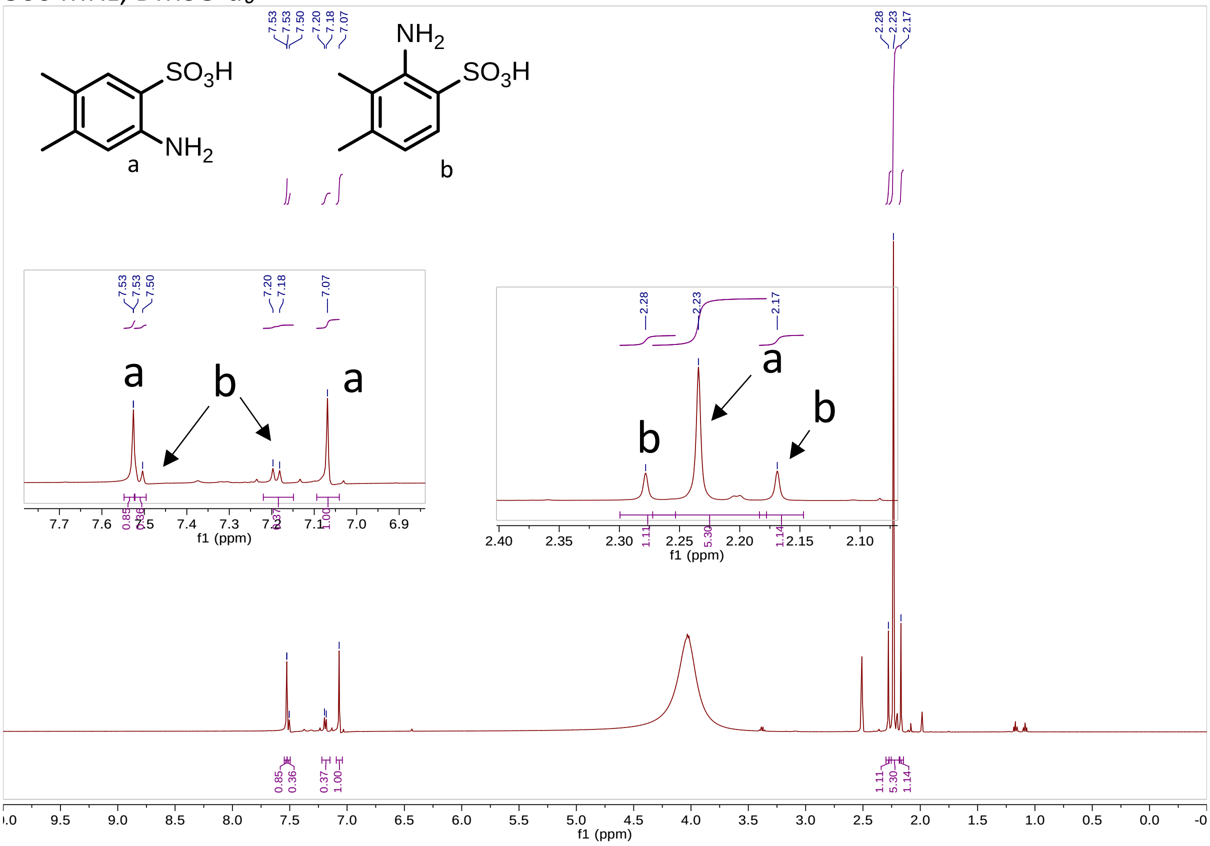

126 MHz, DMSO-d_6

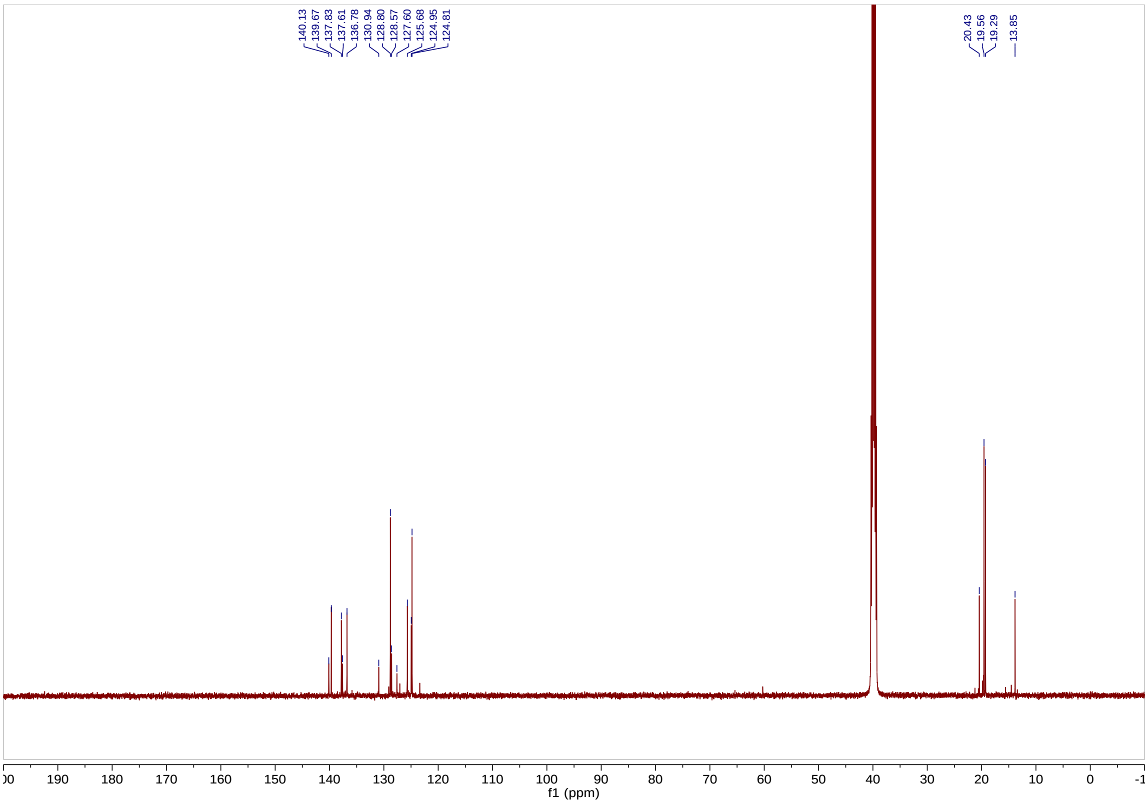

2-Amino-3,5-dimethylbenzenesulfonic acid 2f

400 MHz, DMSO-d_6

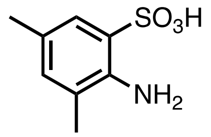

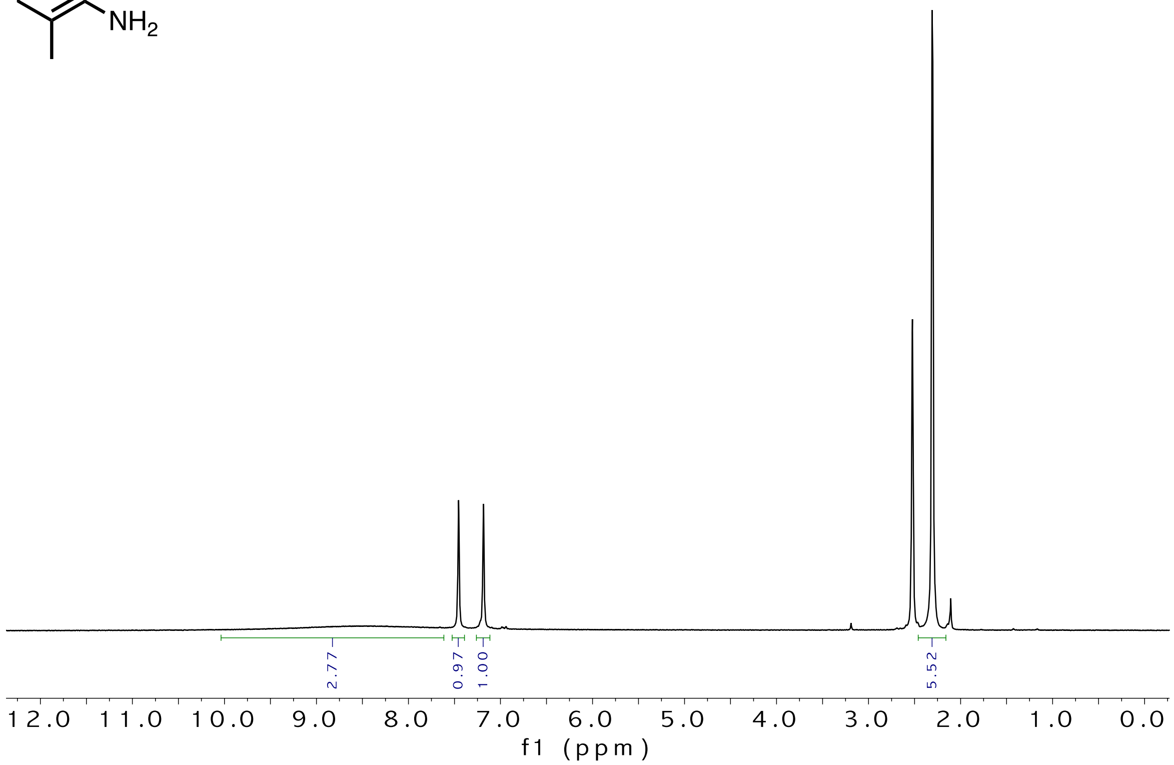

101 MHz, DMSO-d_6

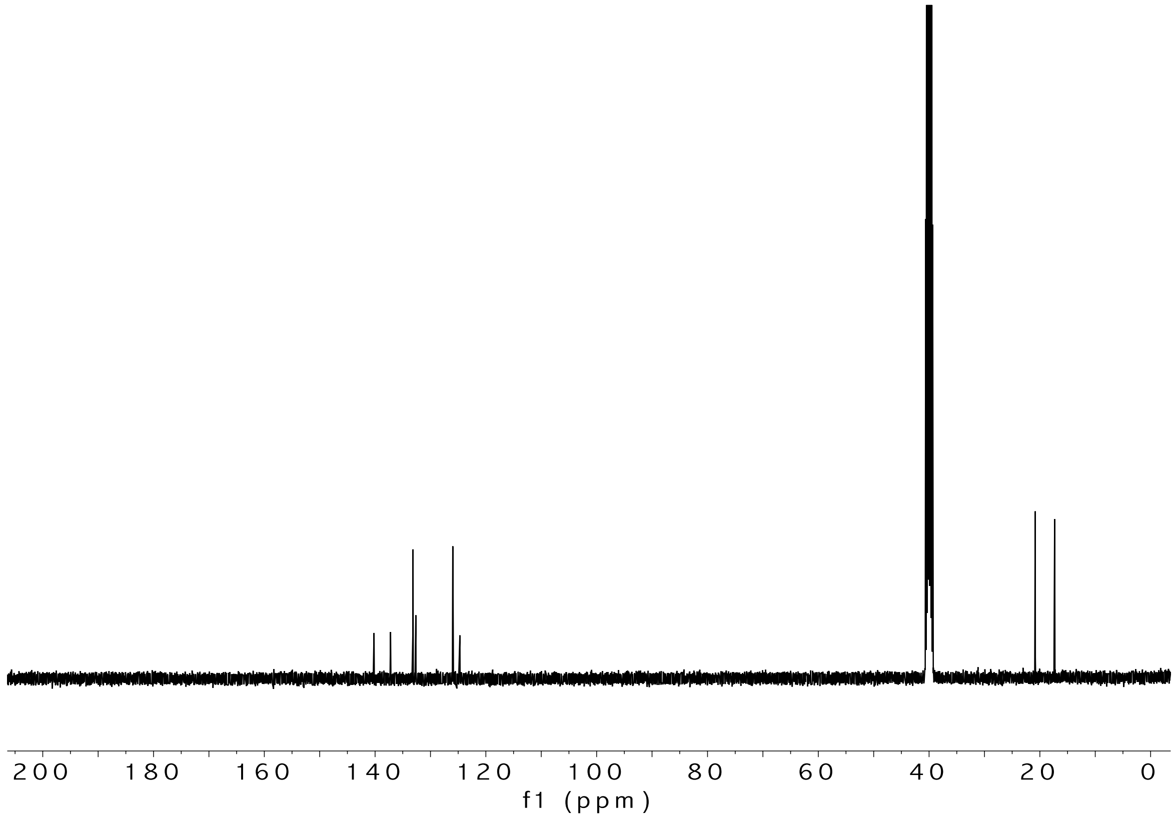

2-Amino-4-butylbenzenesulfonic acid 2g

400 MHz, DMSO-d_6

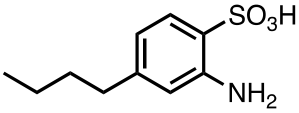

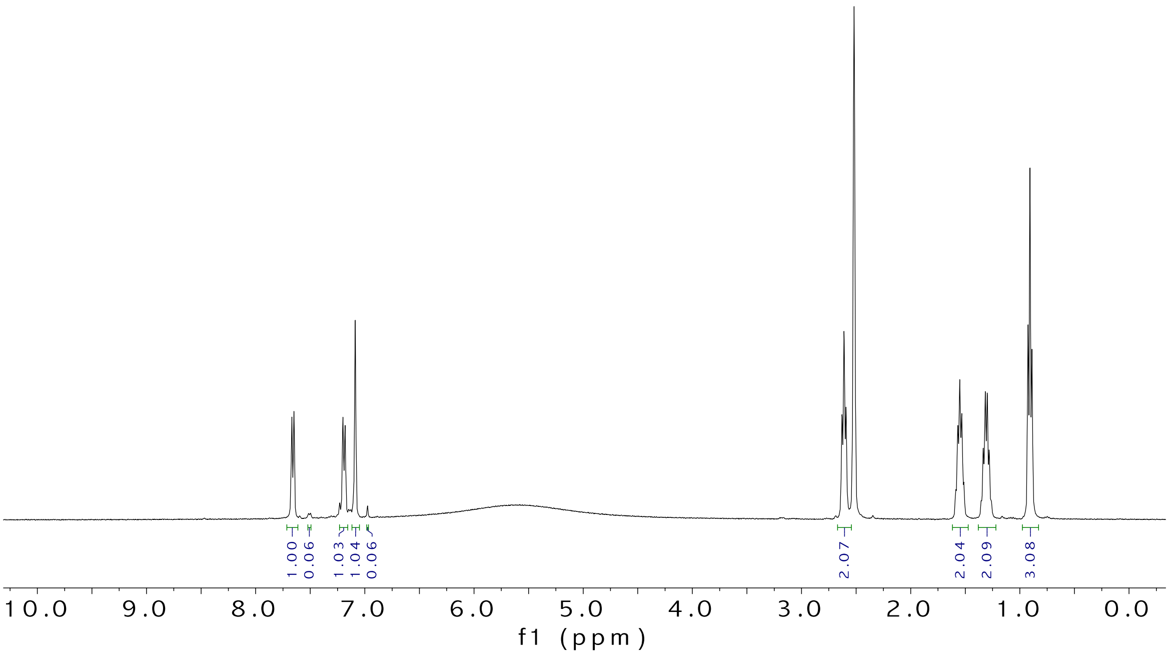

101 MHz, DMSO-d_6

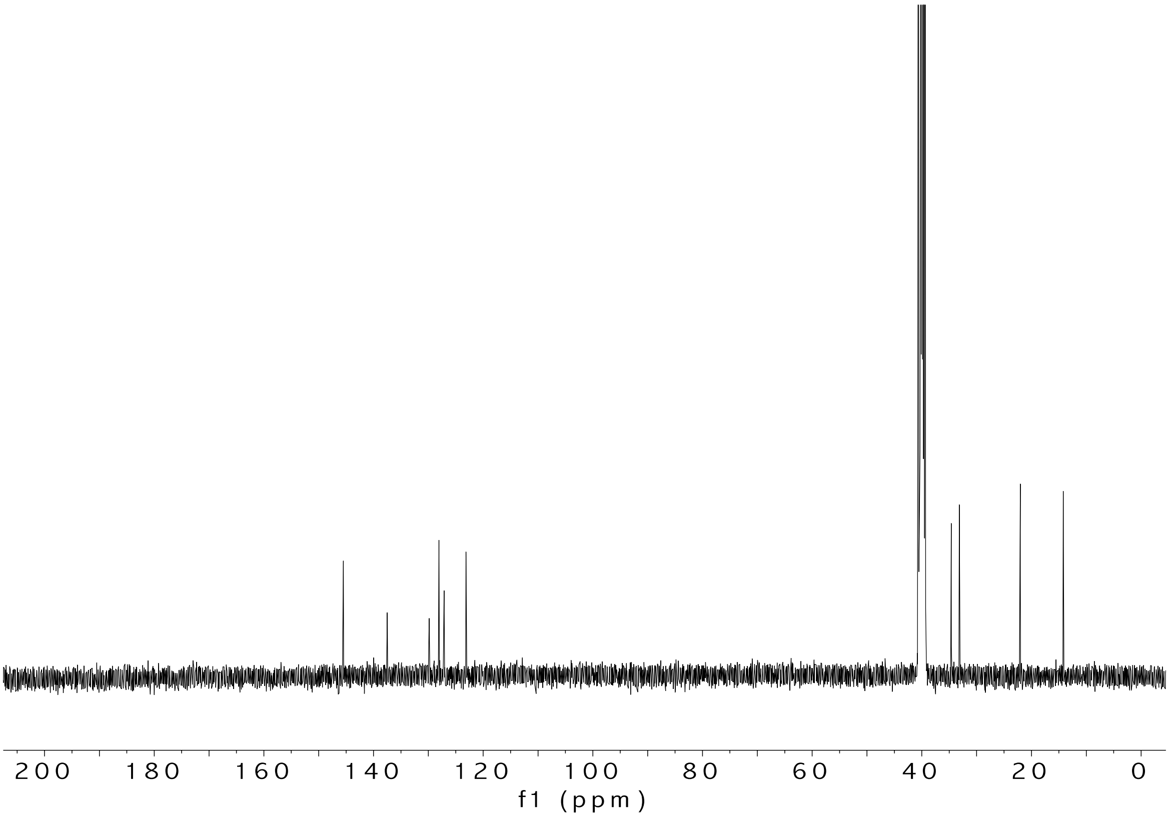

2-Amino-4-(*tert*-butyl)benzenesulfonic acid 2h

400 MHz, DMSO-d_6

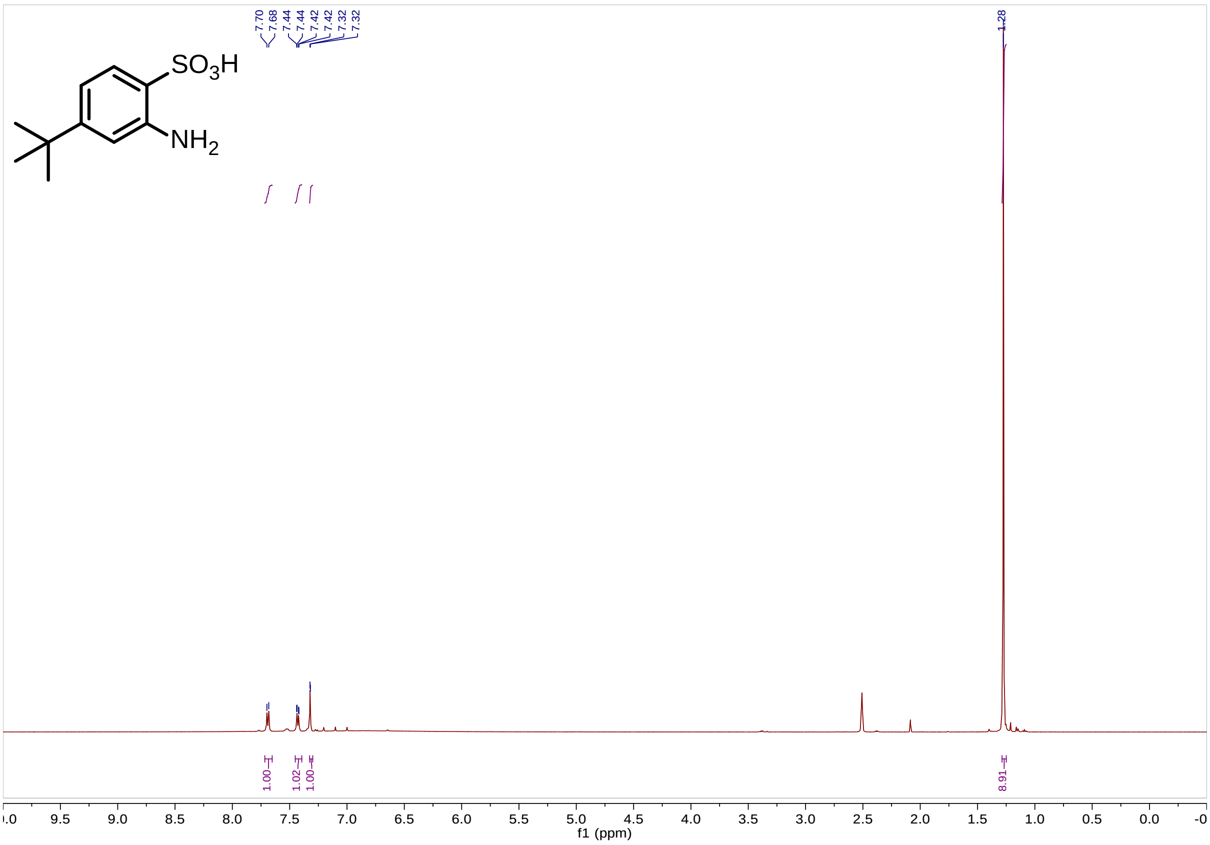

101 MHz, DMSO-d_6

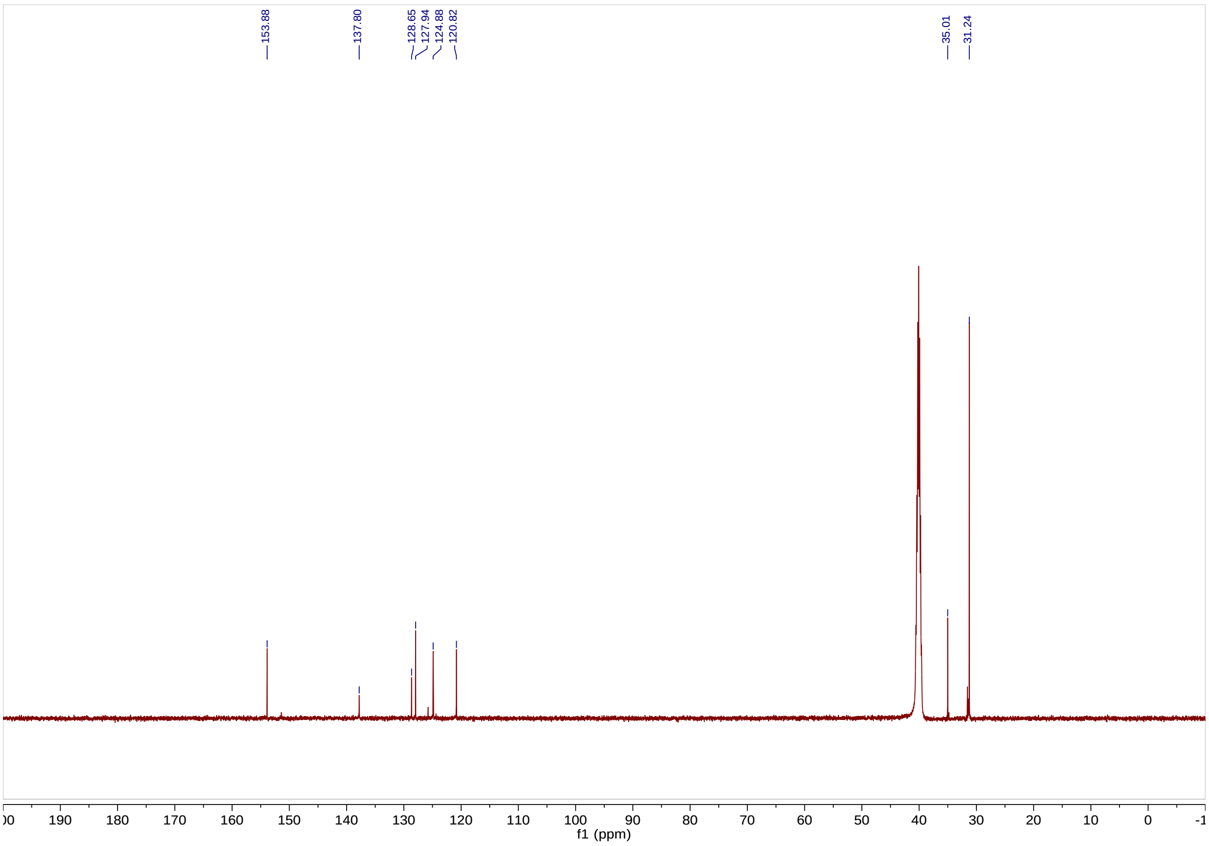

2-Amino-5-fluoro-4-methylbenzenesulfonic acid and 2-amino-3-fluoro-4-methylbenzenesulfonic acid 2i

400 MHz, DMSO-d_6

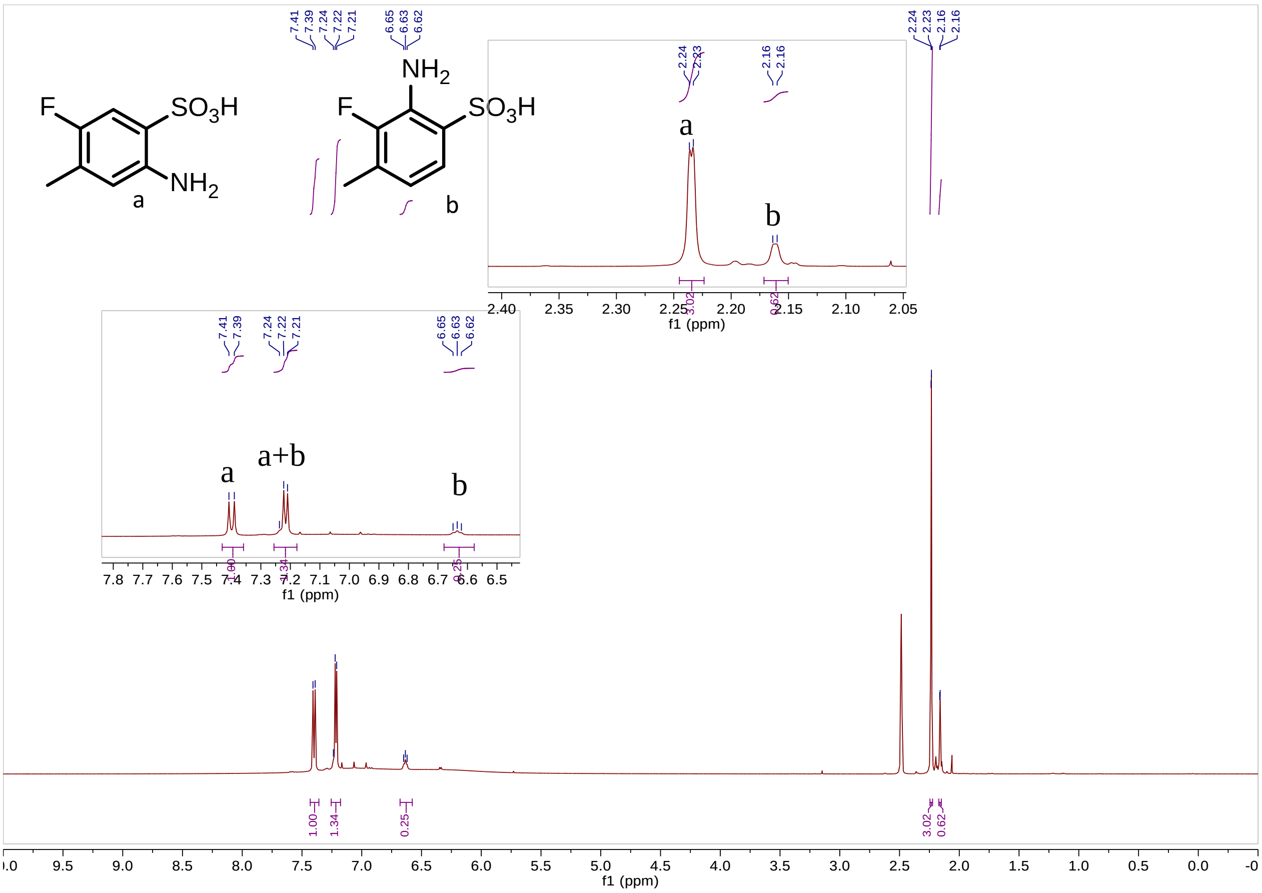

101 MHz, DMSO-d_6

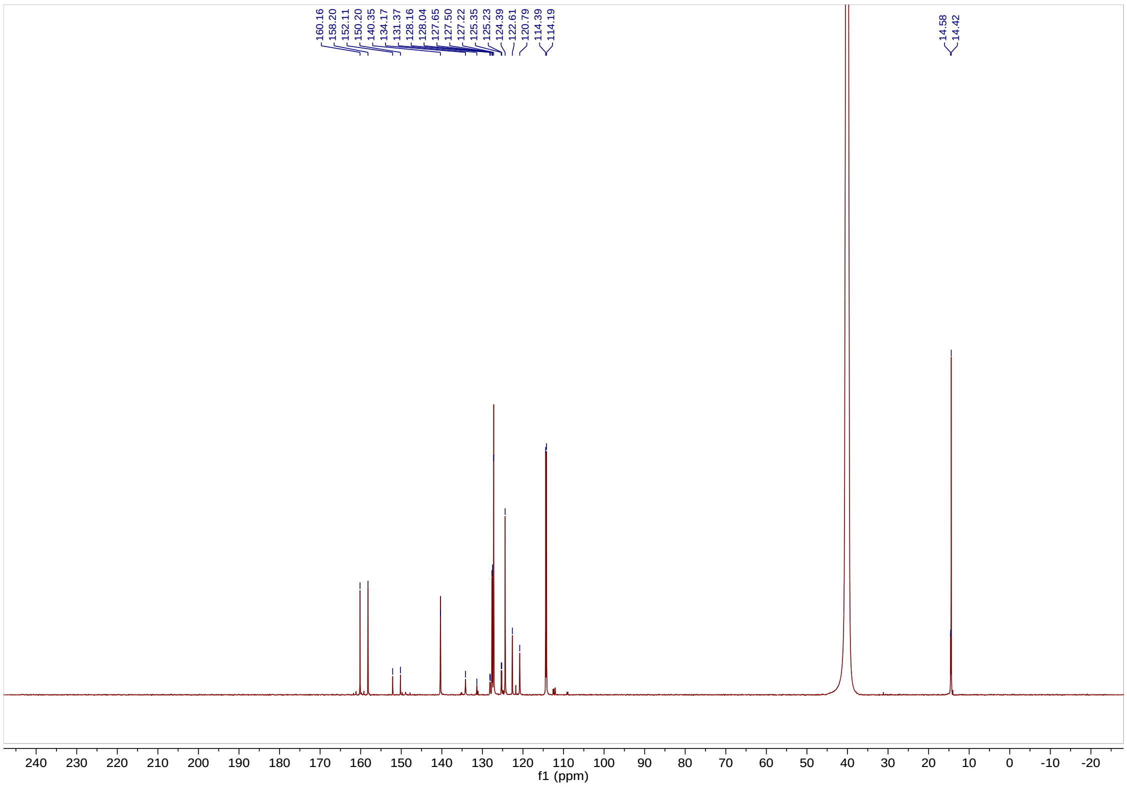

471 MHz, DMSO-d_6

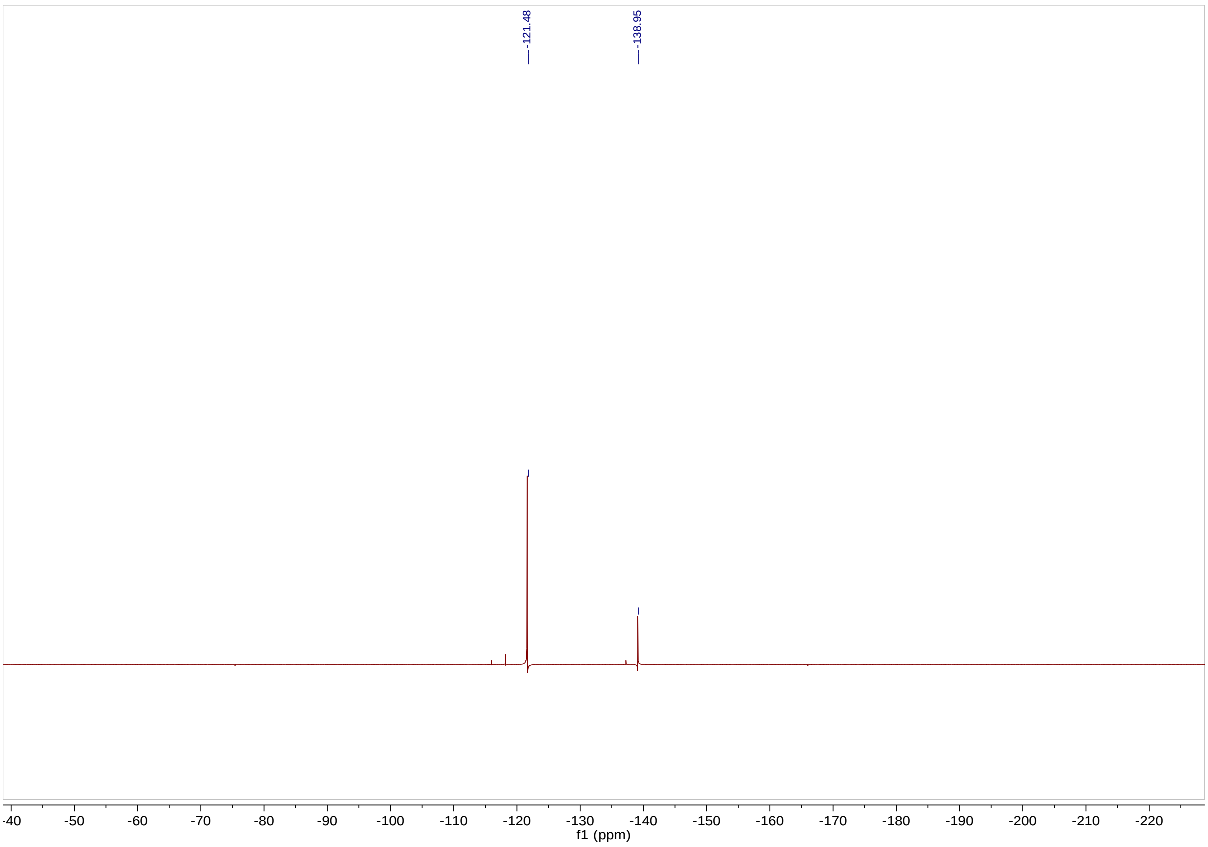

2-Amino-5-fluorobenzenesulfonic acid and 2-amino-3-fluorobenzenesulfonic acid 2j

400 MHz, DMSO-d_6

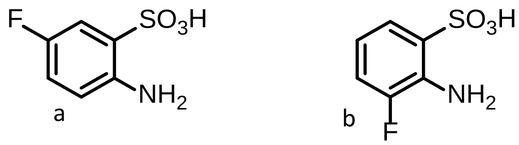

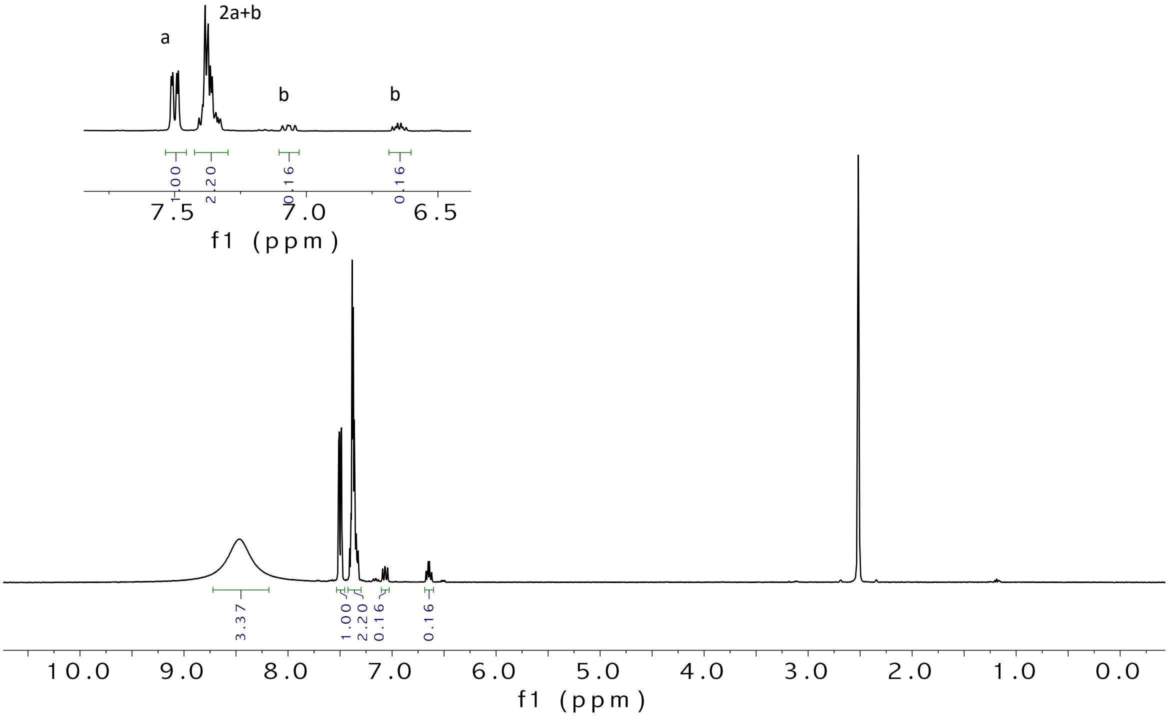

101 MHz, DMSO-d_6

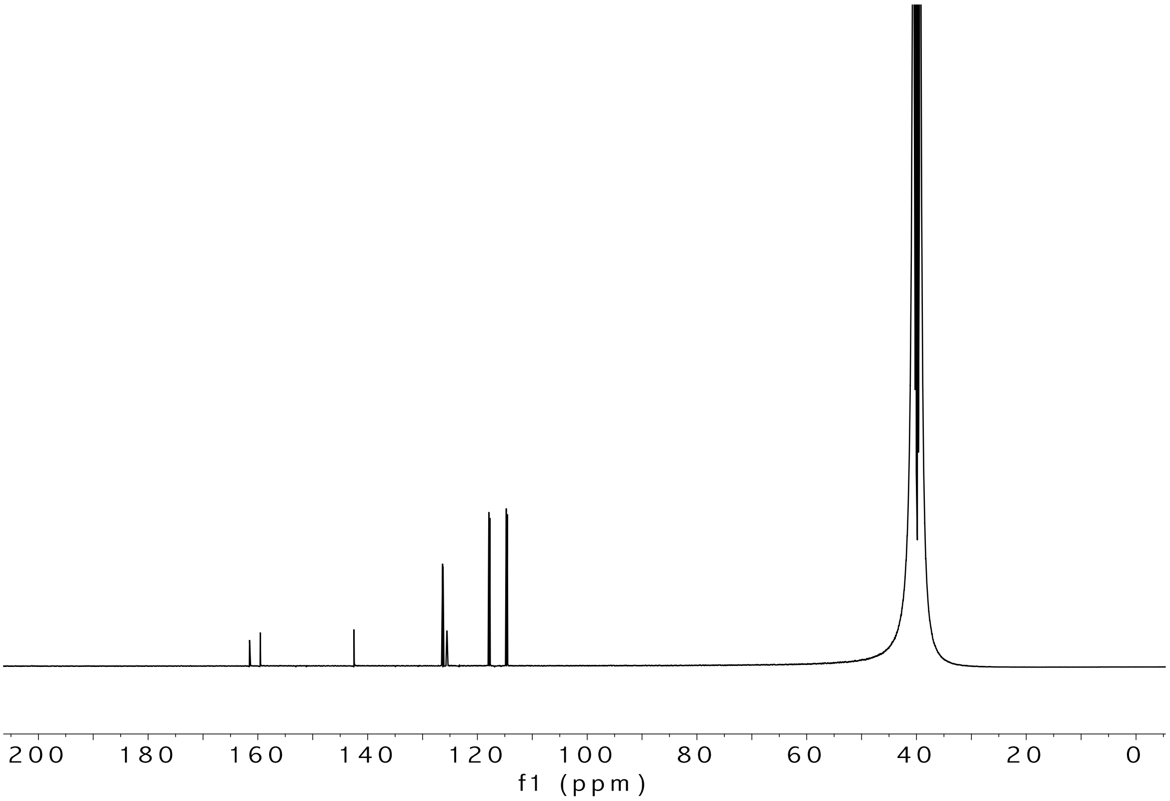

471 MHz, DMSO-d_6

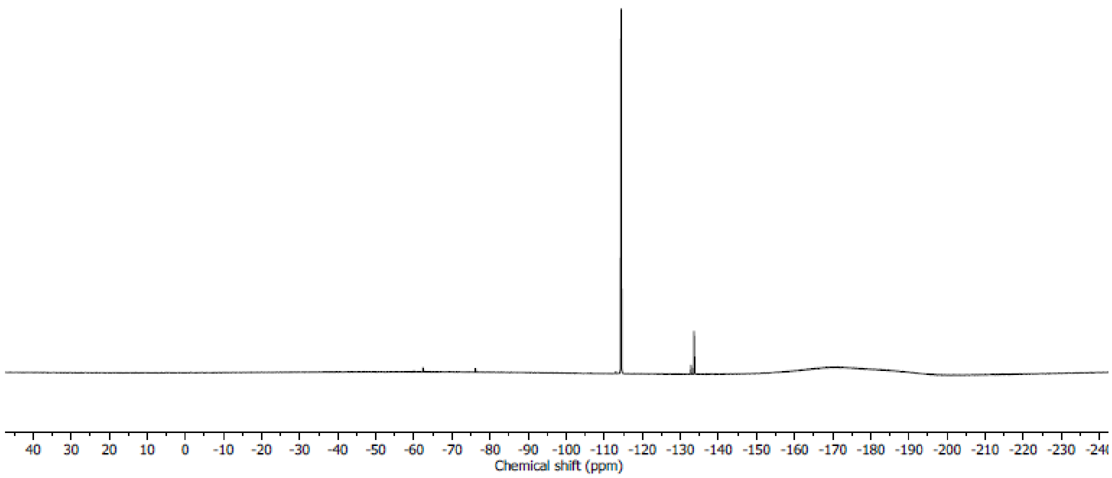

2-Amino-3,4,5-trifluorobenzenesulfonic acid 2k

400 MHz, DMSO-d_6

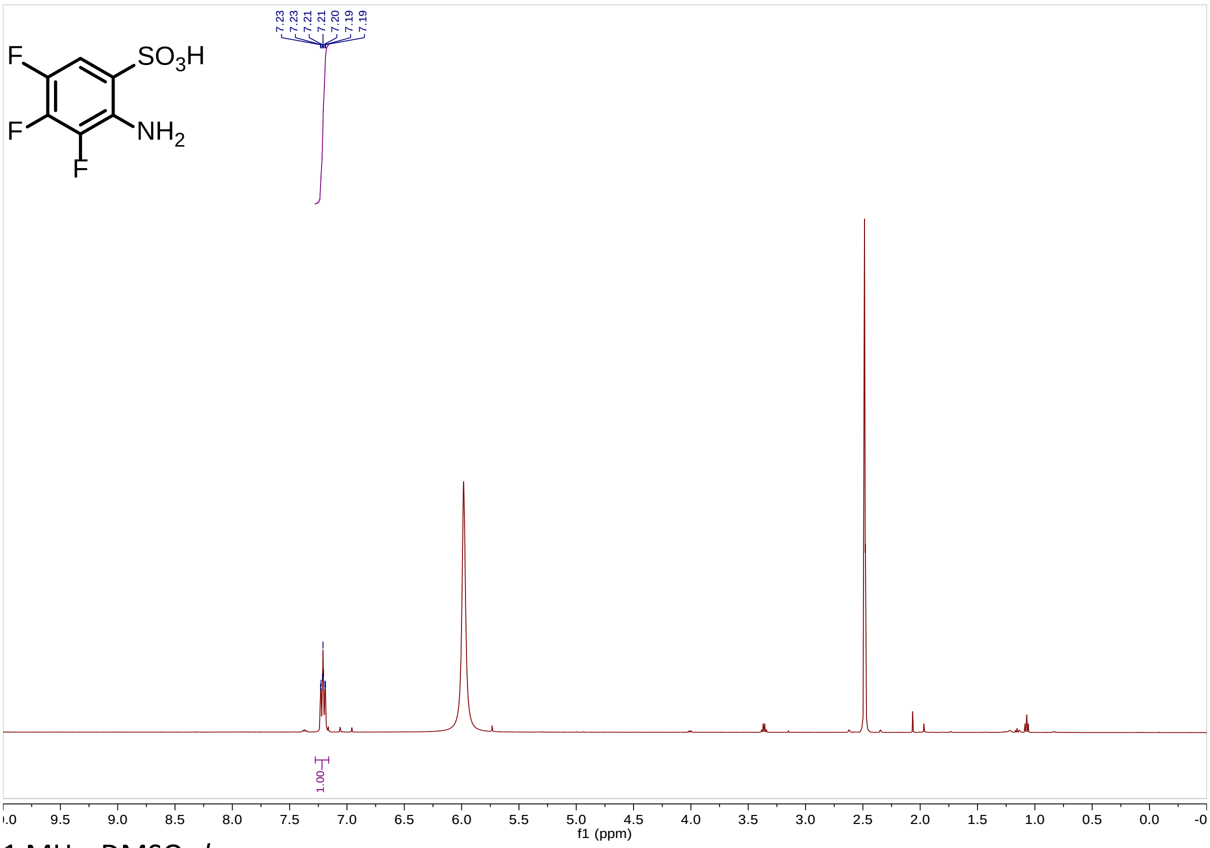

101 MHz, DMSO-d_6

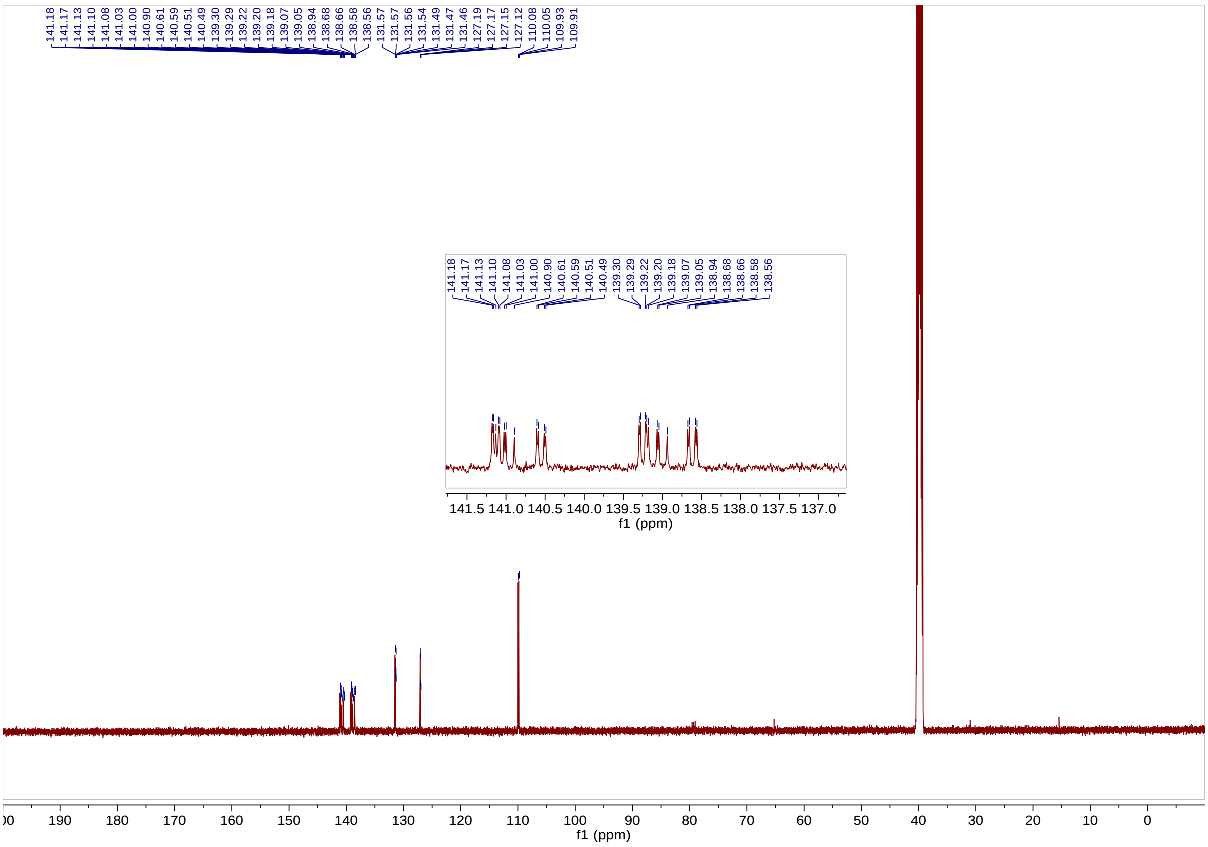

471 MHz, DMSO-d_6

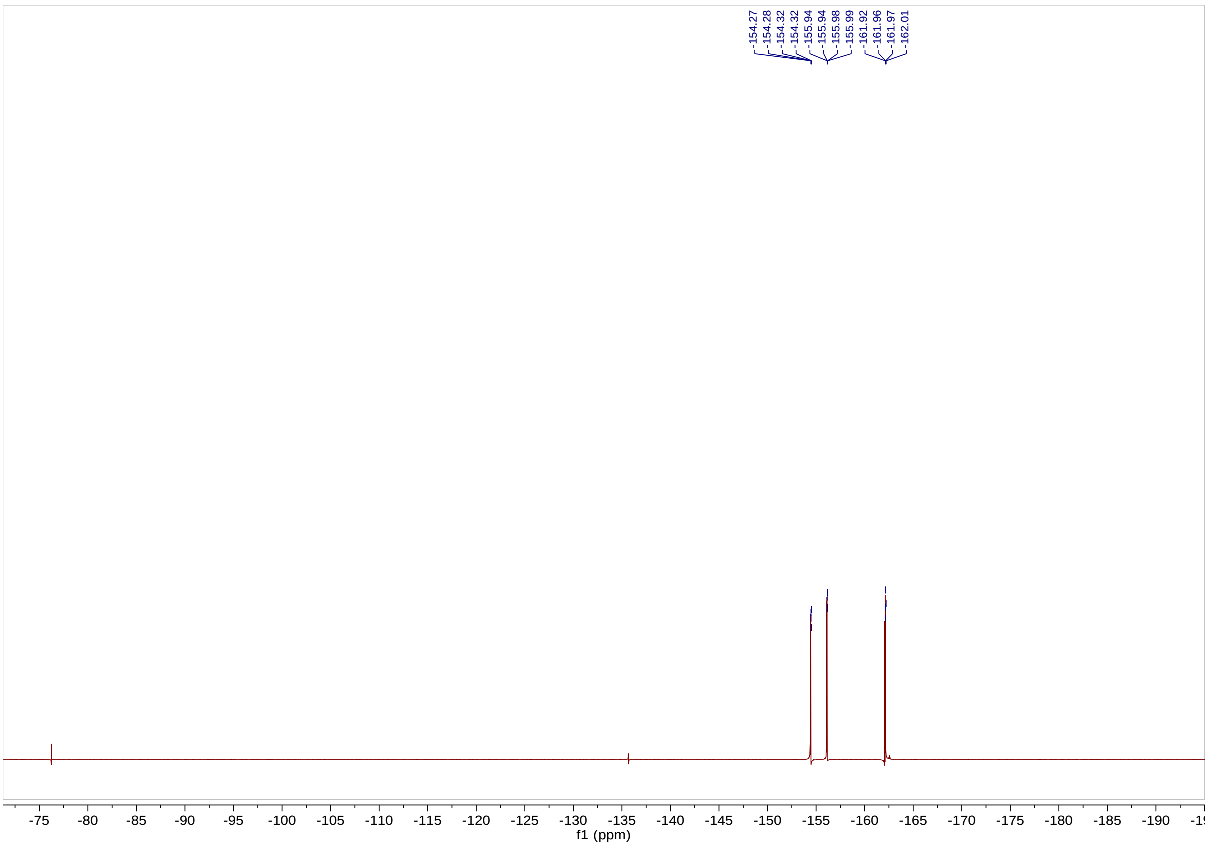

2-Amino-5-chloro-4-fluorobenzenesulfonic acid 2l

500 MHz, DMSO-d_6

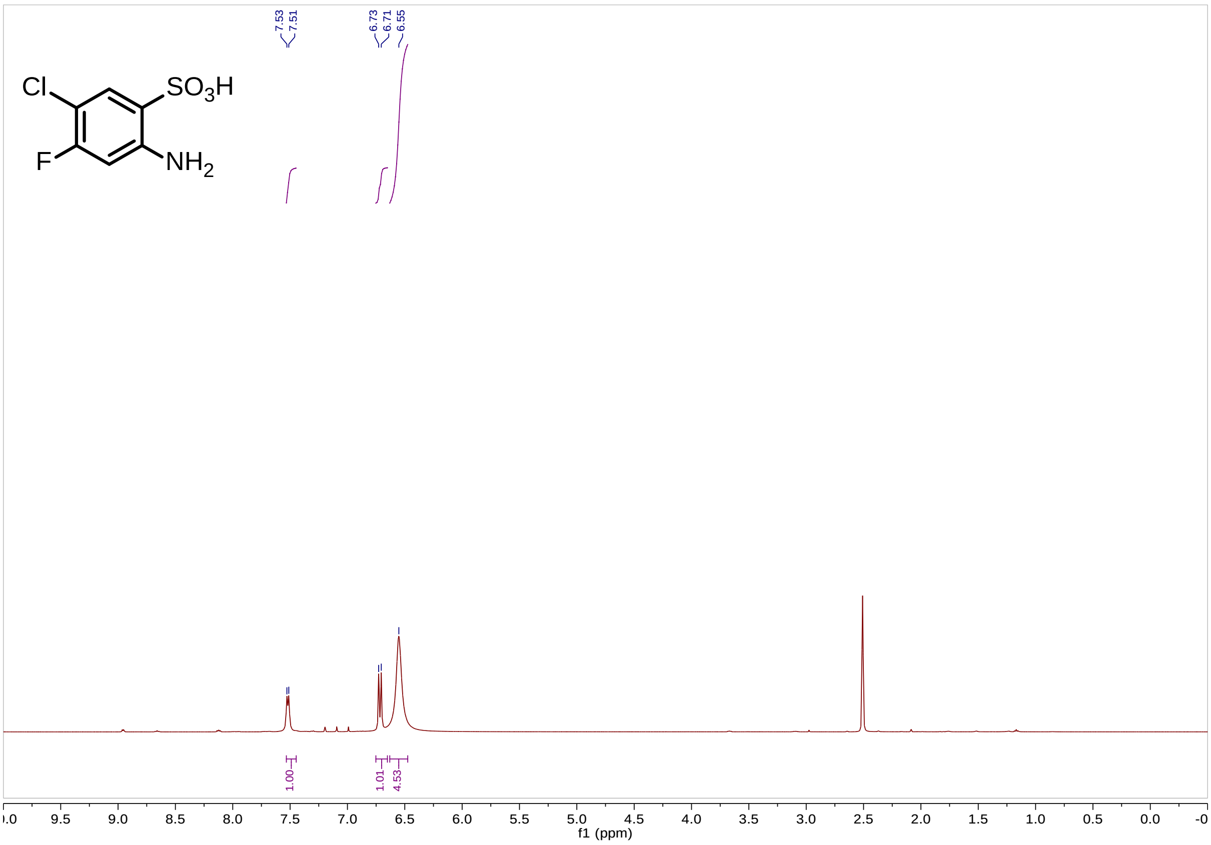

126 MHz, DMSO-d_6

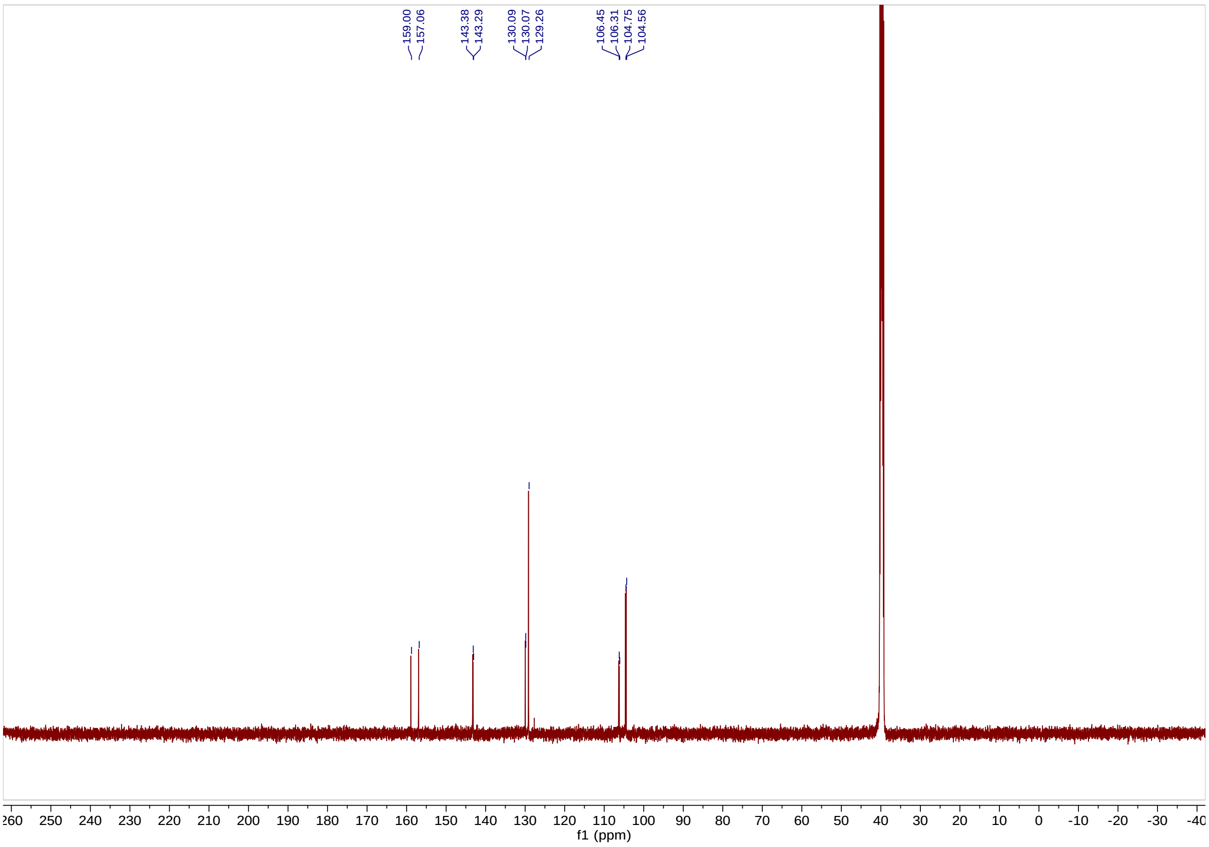

471 MHz, DMSO-d_6

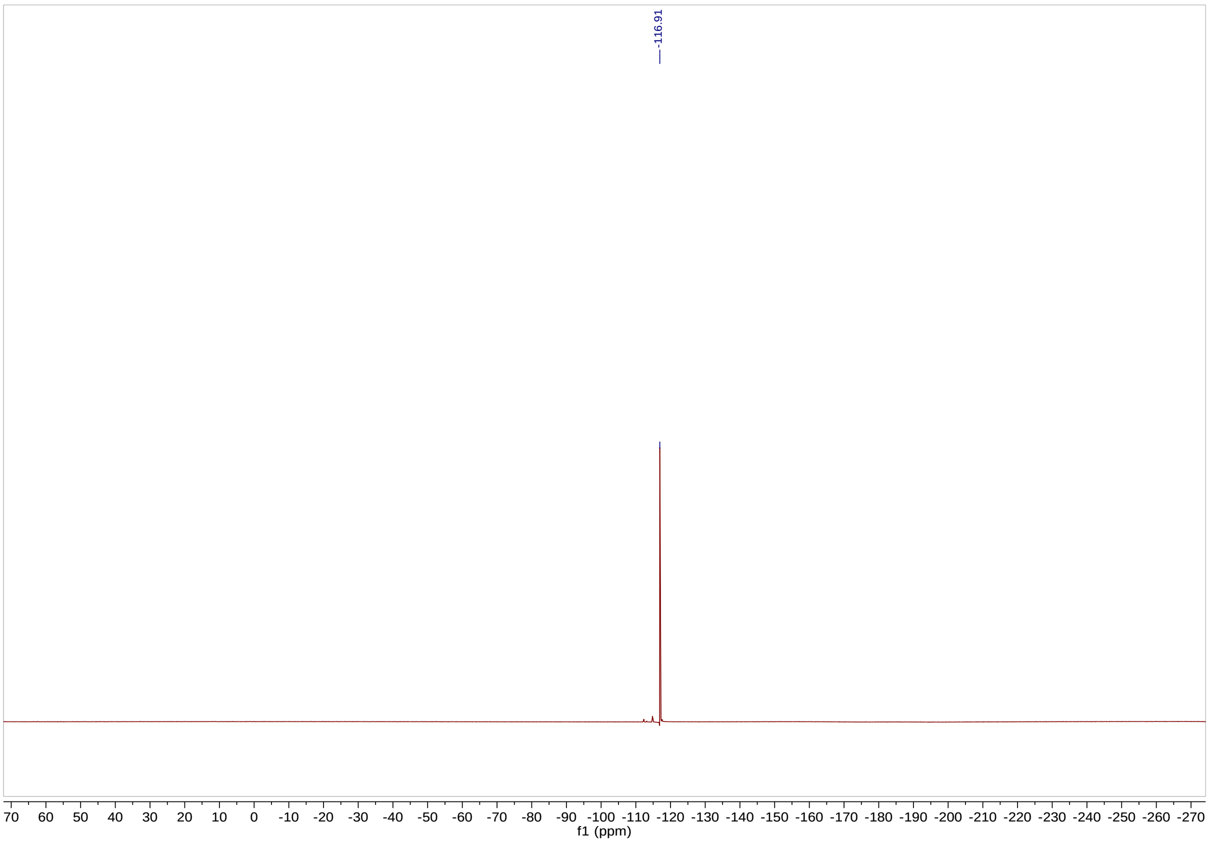

2-Amino-5-chlorobenzenesulfonic acid 2m

400 MHz, DMSO-d_6

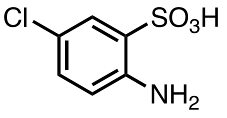

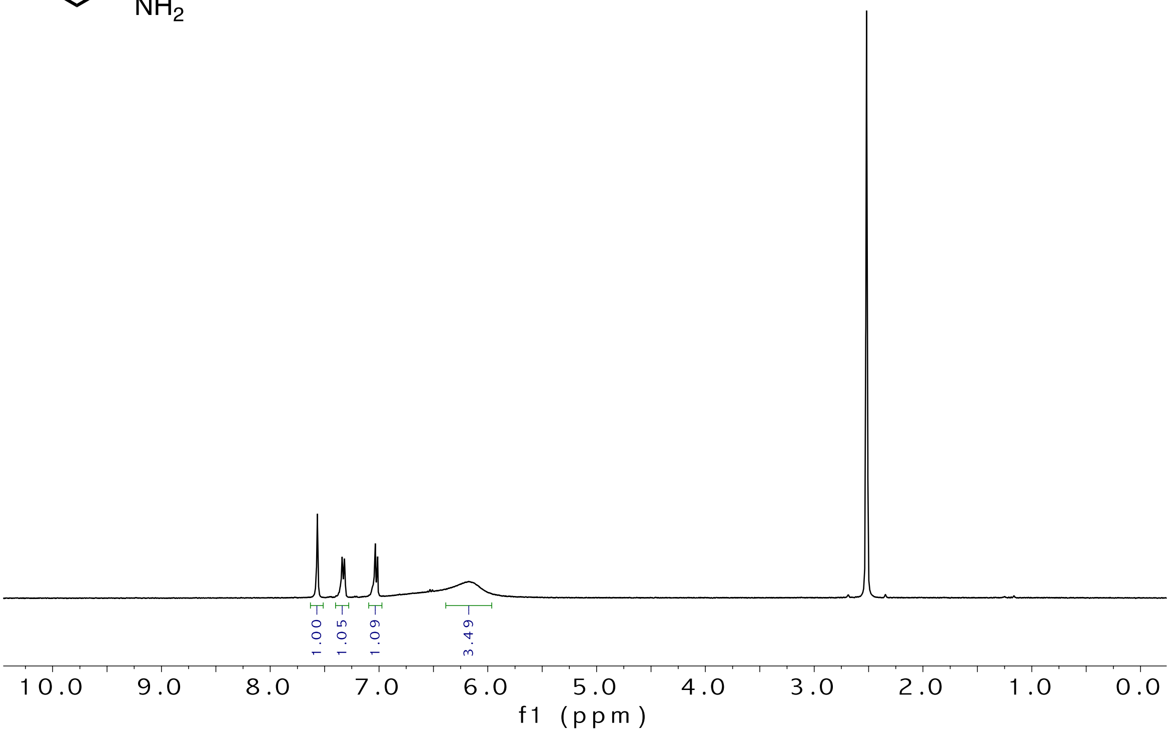

101 MHz, DMSO-d_6

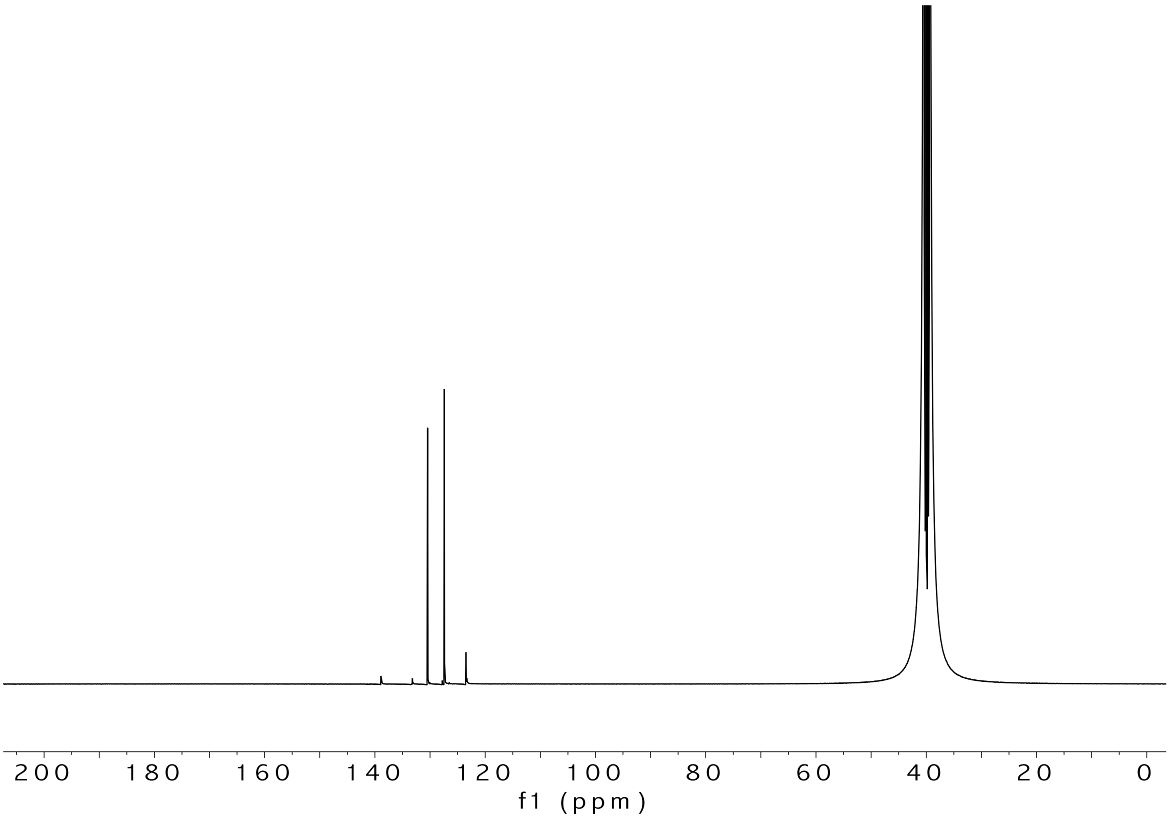

2-Amino-6-chlorobenzenesulfonic acid 2n

500 MHz, DMSO-d_6

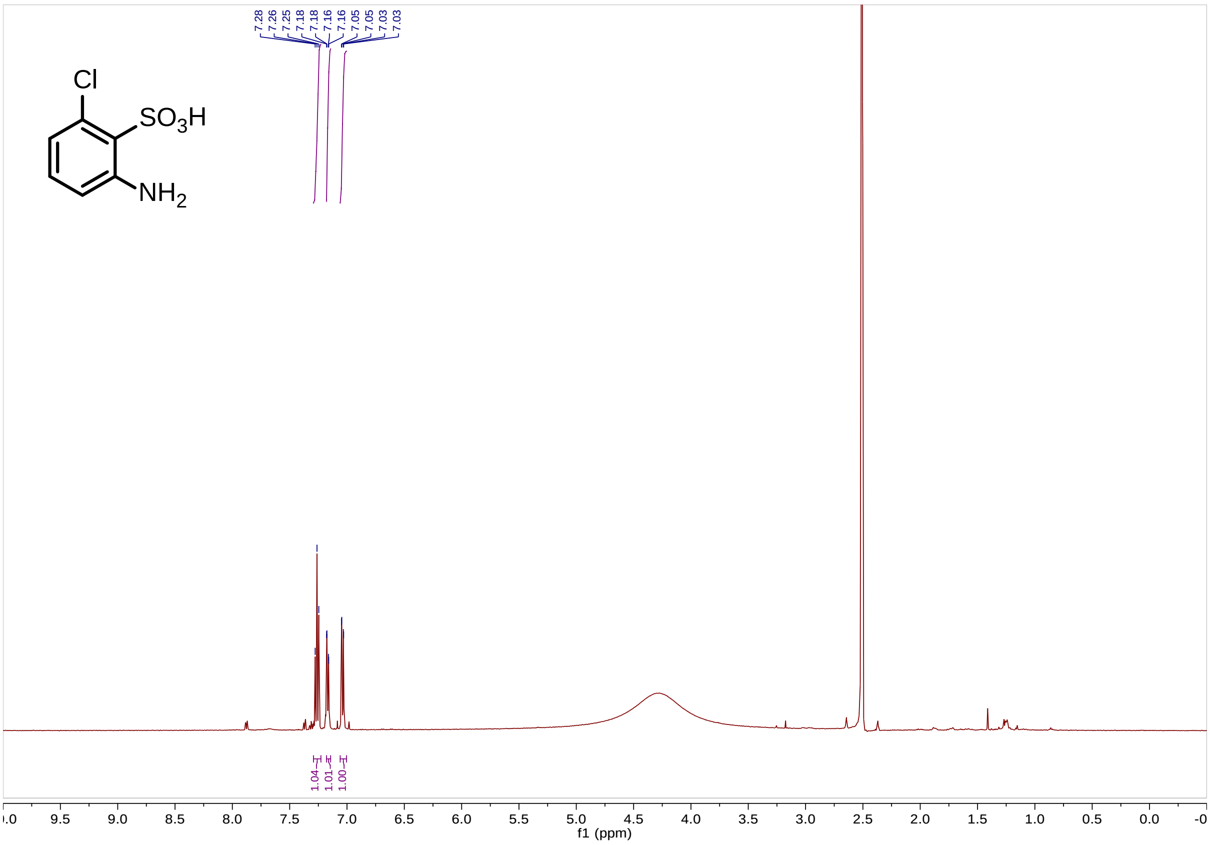

126 MHz, DMSO-d_6

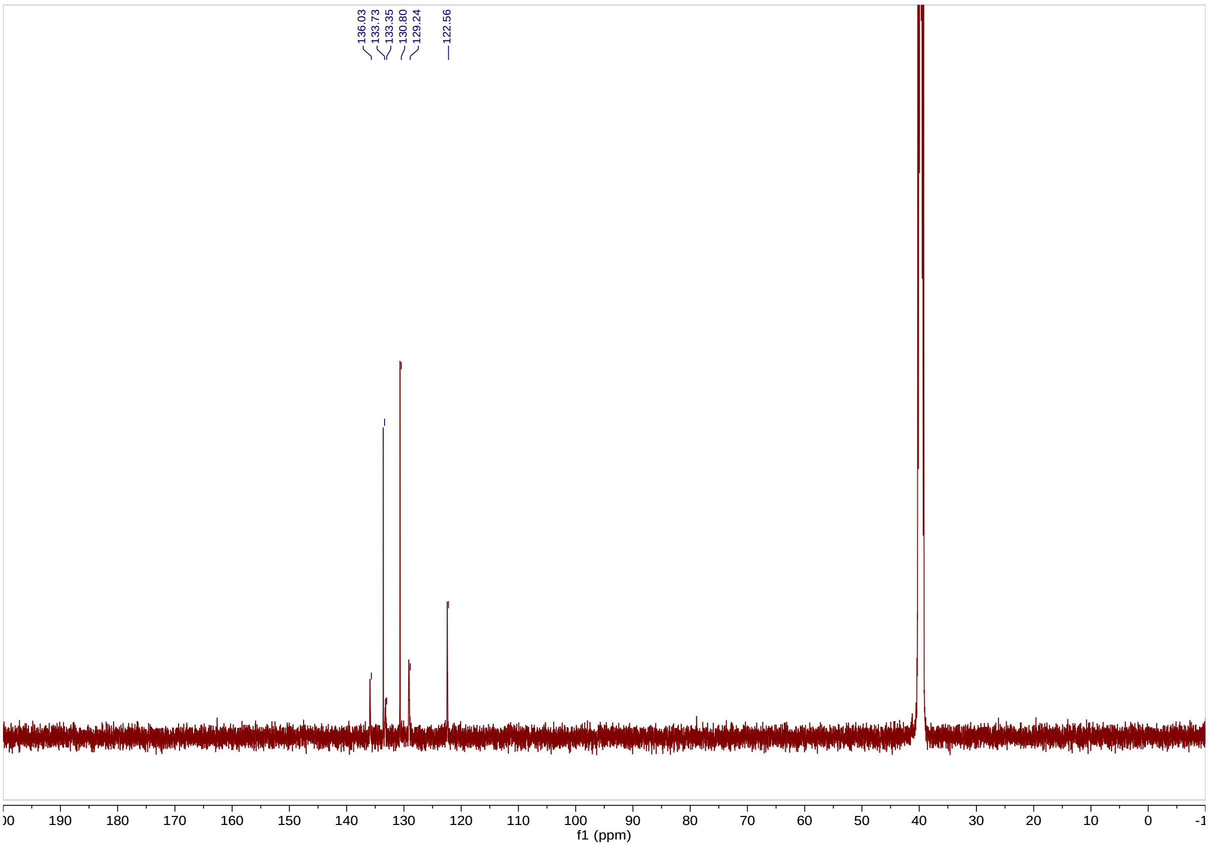

2-Amino-4-chlorobenzenesulfonic acid 2o

500 MHz, DMSO-d_6

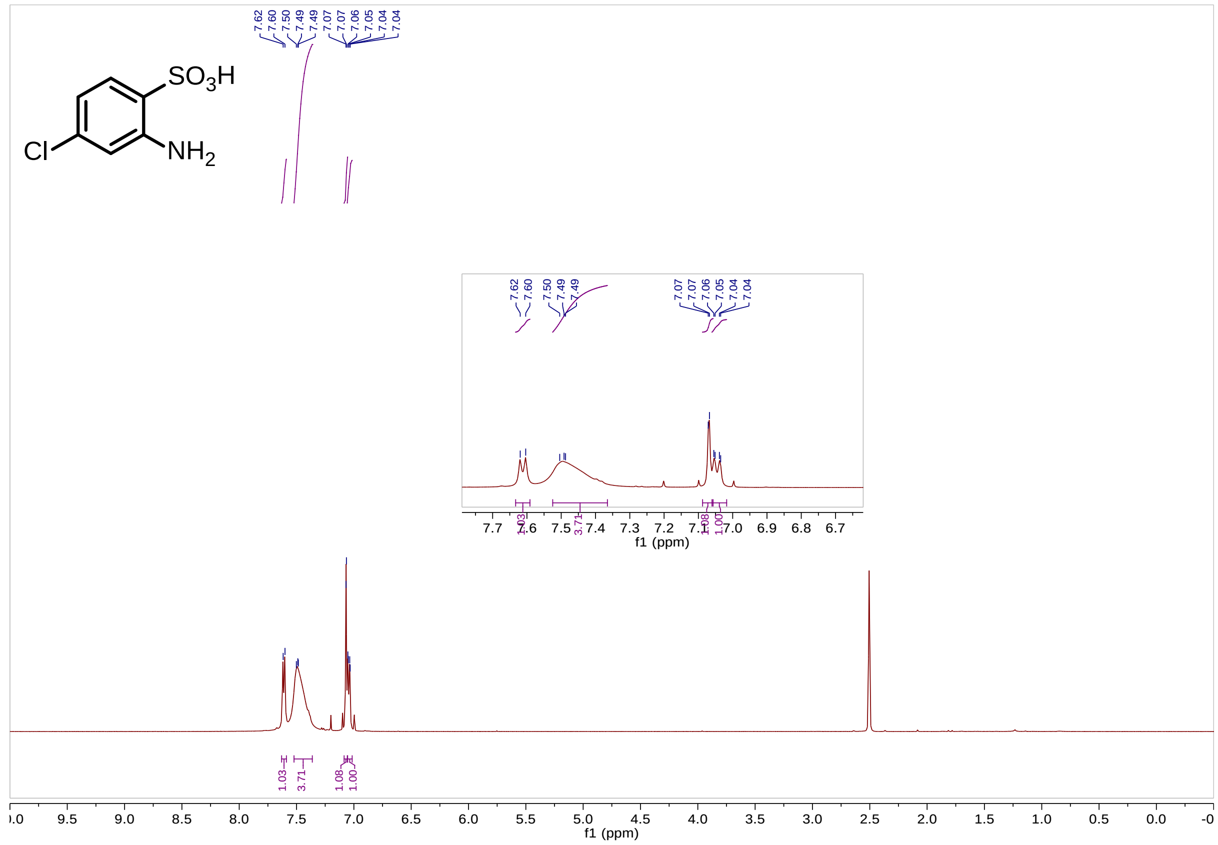

126 MHz, DMSO-d_6

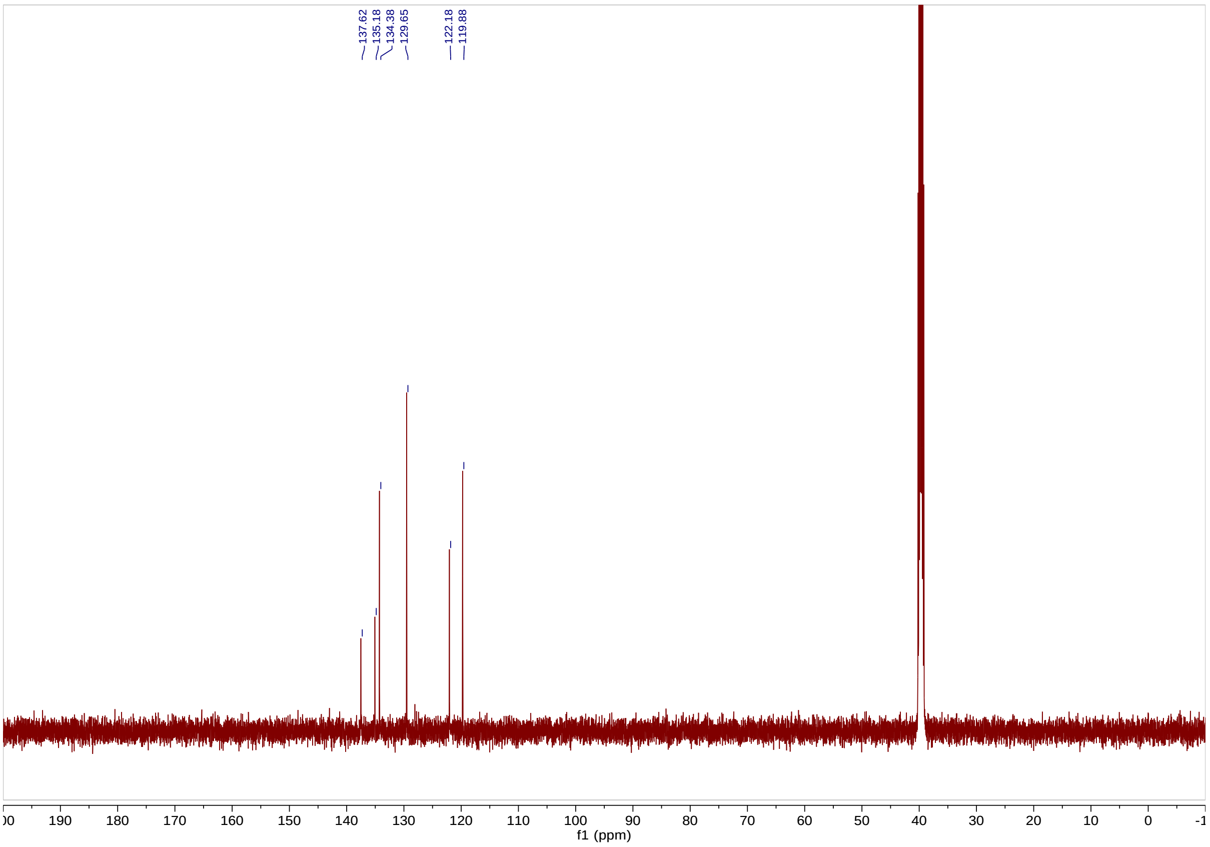

2-Amino-3,5-dichlorobenzenesulfonic acid 2p

400 MHz, DMSO-d_6

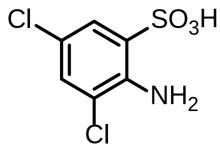

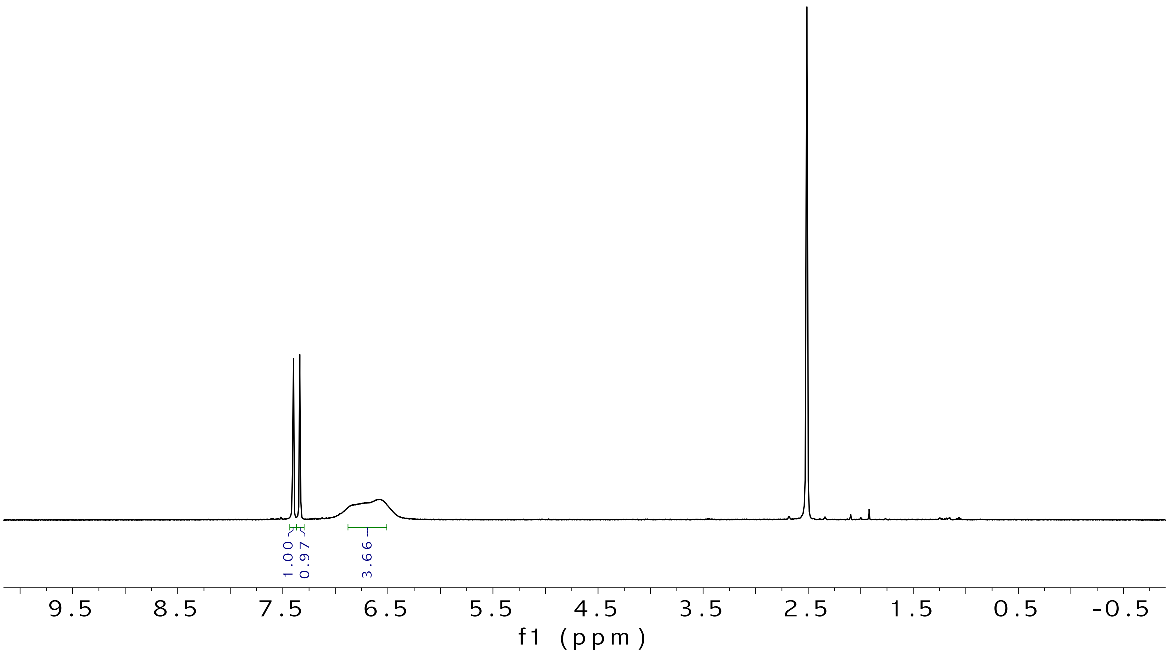

101 MHz, DMSO-d_6

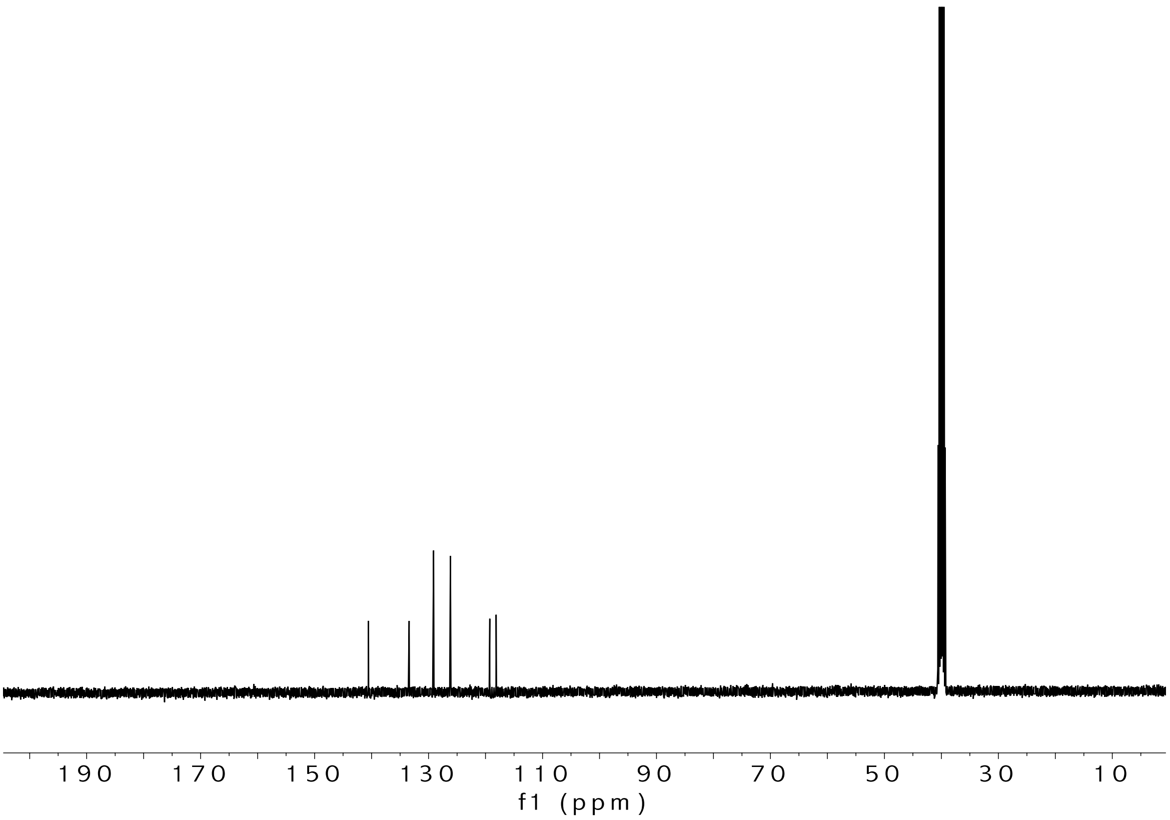

2-Amino-5-chloro-4-methylbenzenesulfonic acid and 2-amino-3-chloro-4-methylbenzenesulfonic acid 2q

500 MHz, DMSO-d_6

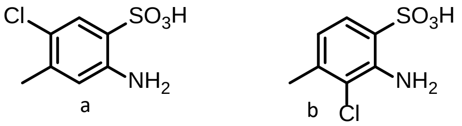

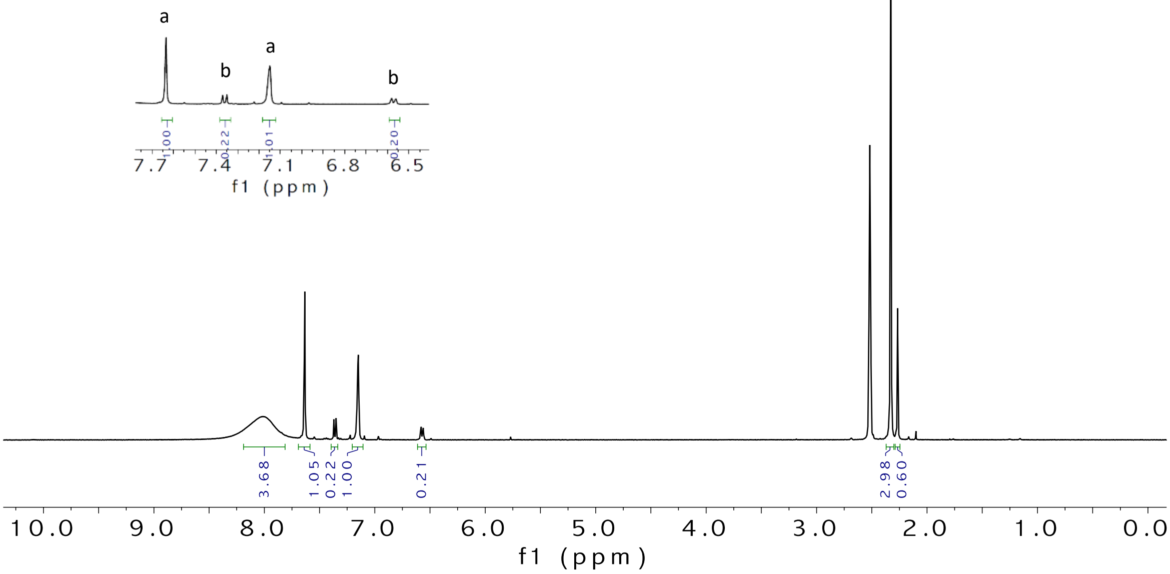

126 MHz, DMSO-d_6

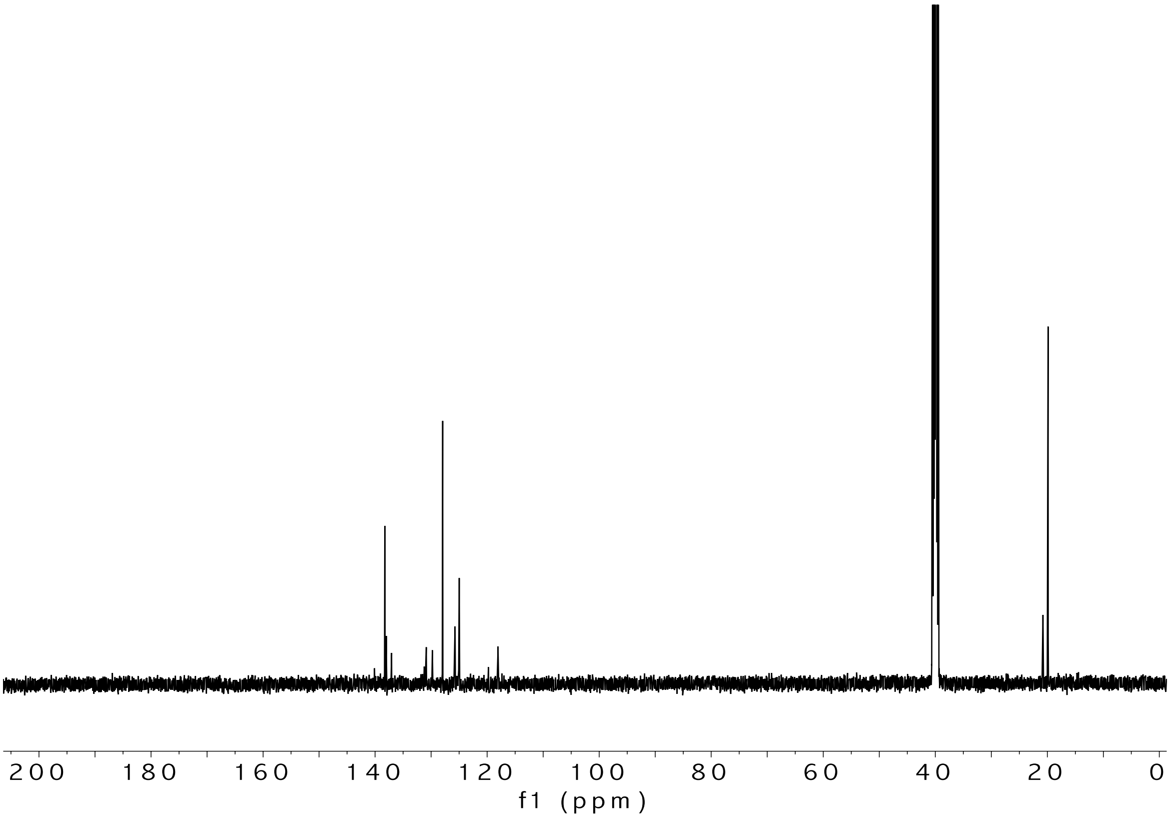

2-Amino-4-bromobenzenesulfonic acid 2r

400 MHz, DMSO-d_6

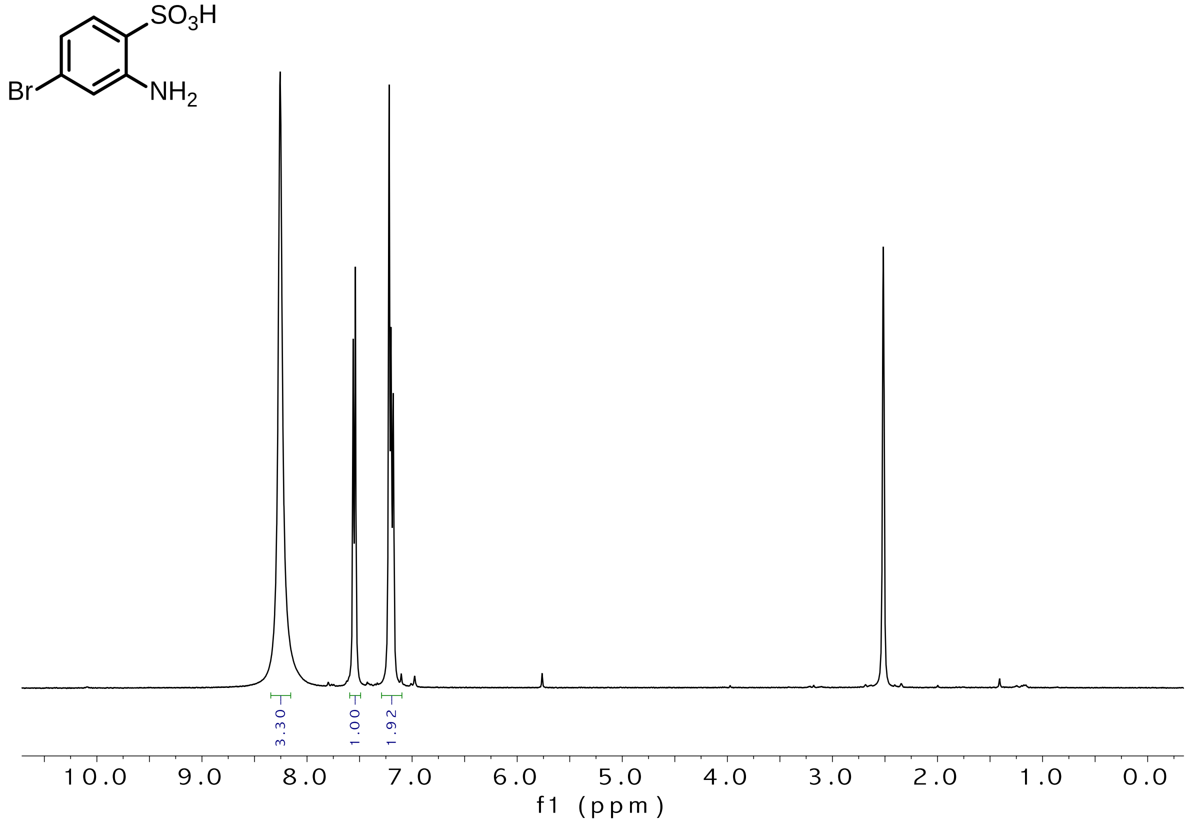

101 MHz, DMSO-d_6

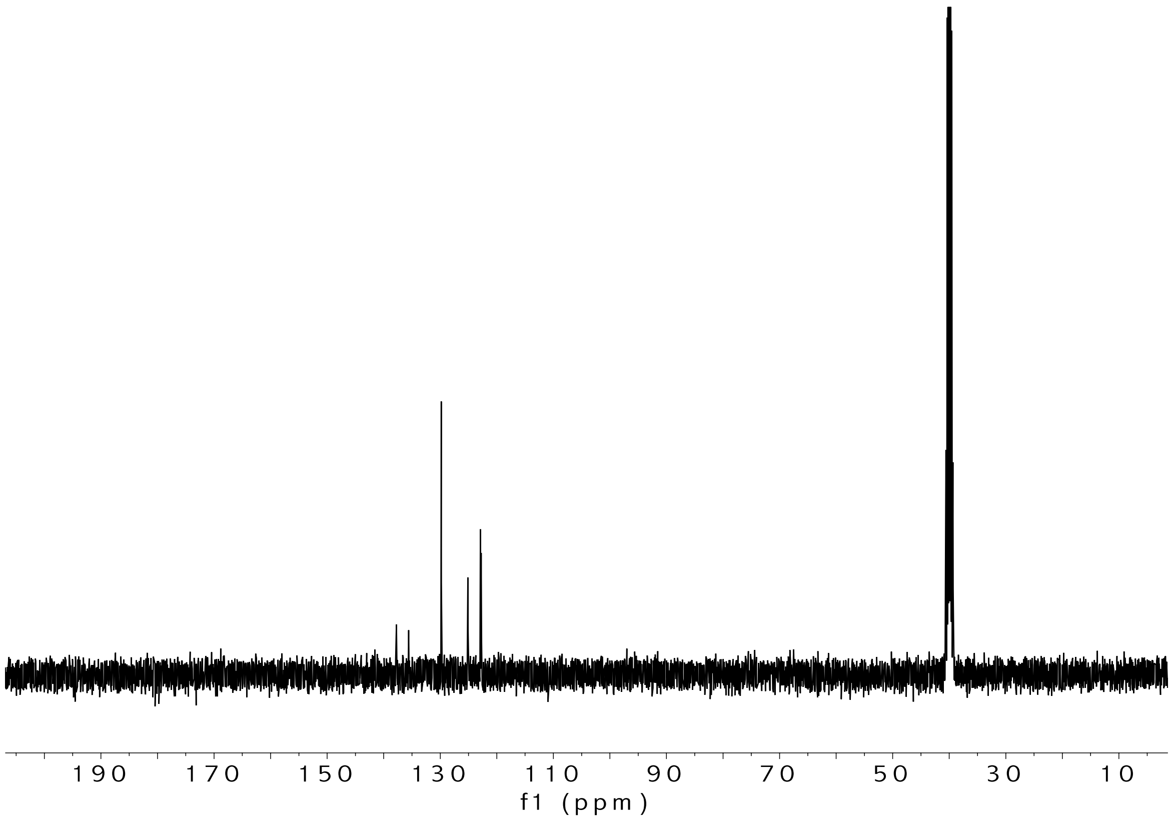

2-Amino-5-bromobenzenesulfonic acid 2s

400 MHz, DMSO-d_6

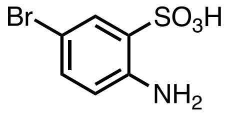

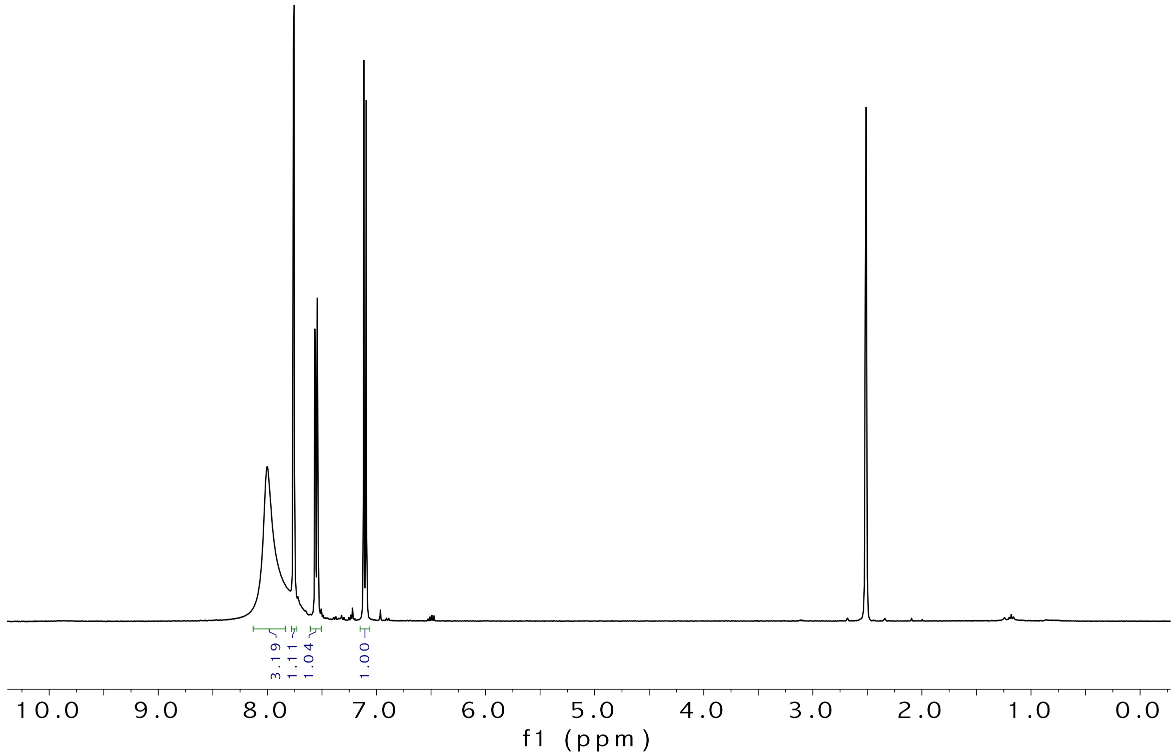

101 MHz, DMSO-d_6

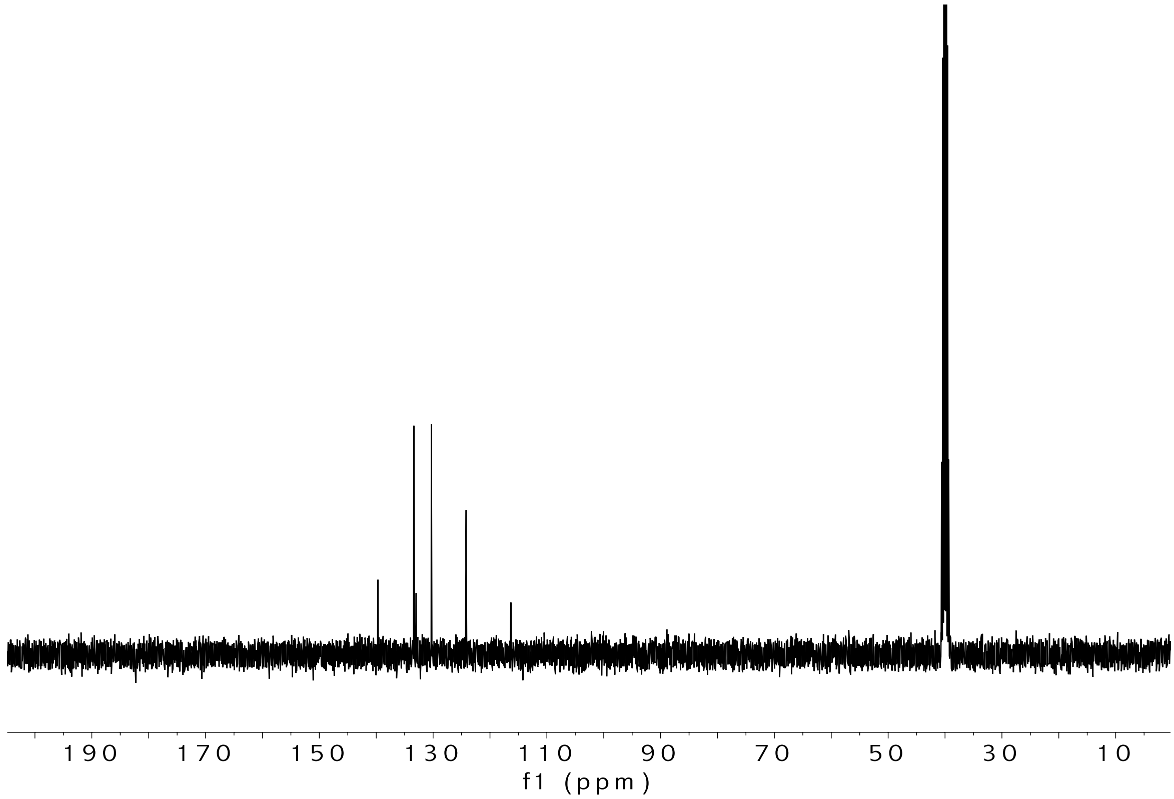

2-Amino-4-methoxybenzenesulfonic acid and 3-amino-4-methoxybenzenesulfonic acid 2t

500 MHz, DMSO-d_6

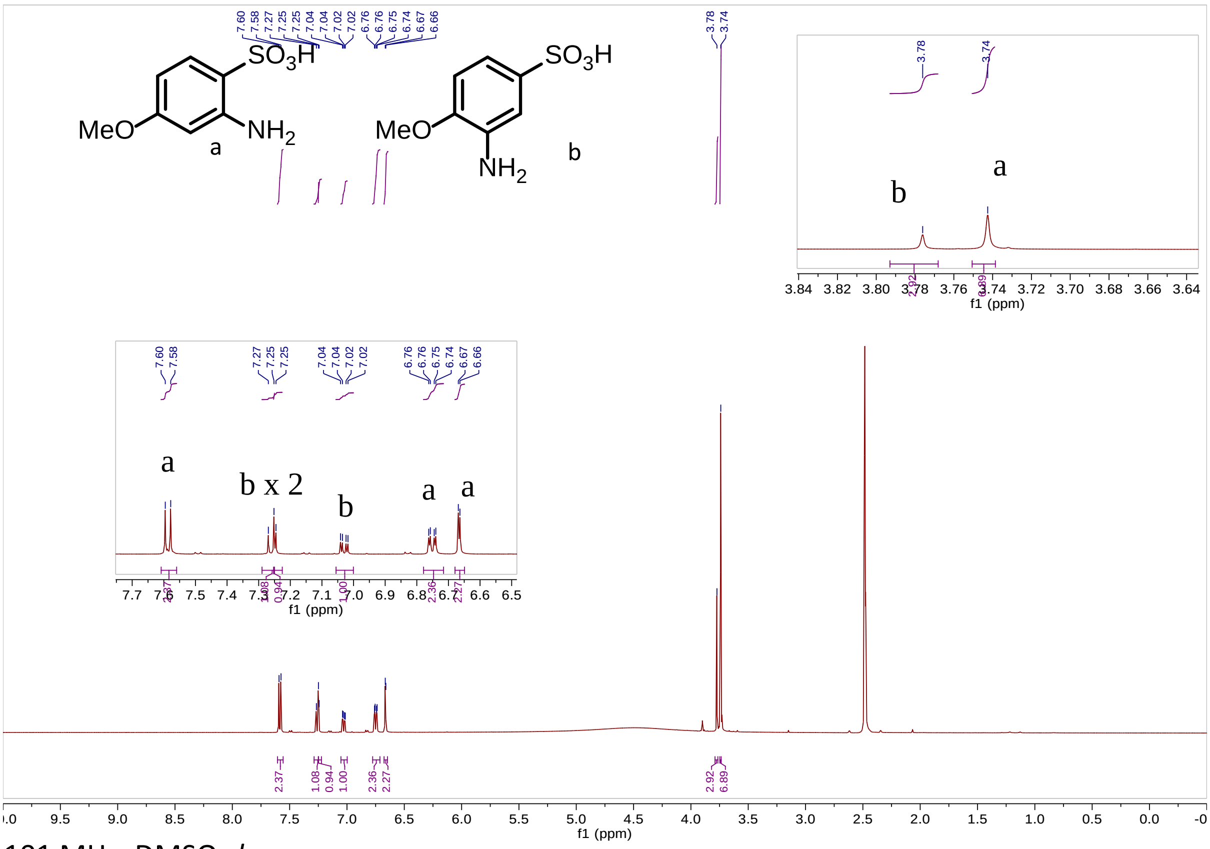

101 MHz, DMSO-d_6

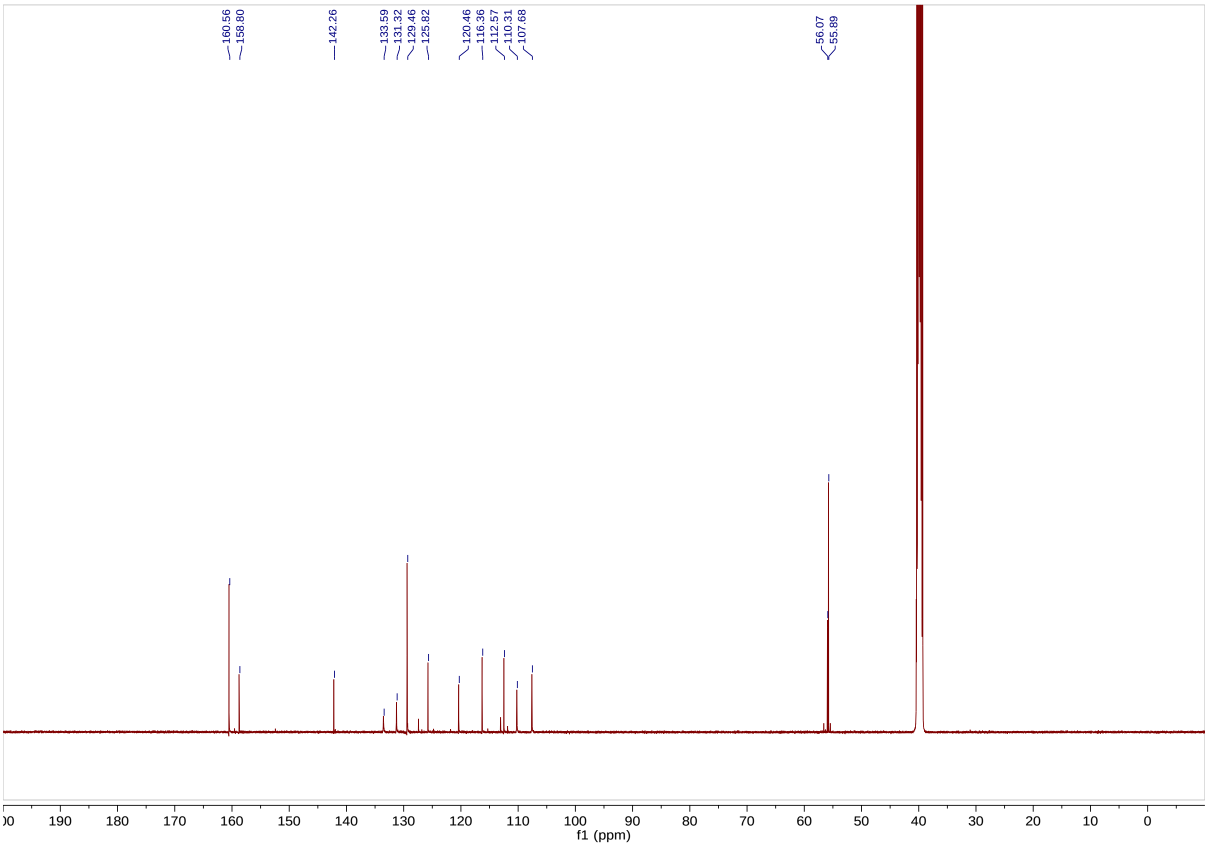

2-Amino-5-fluoro-4-methoxybenzenesulfonic acid and 2-amino-3-fluoro-4-methoxybenzenesulfonic acid 2u

500 MHz, DMSO-d_6

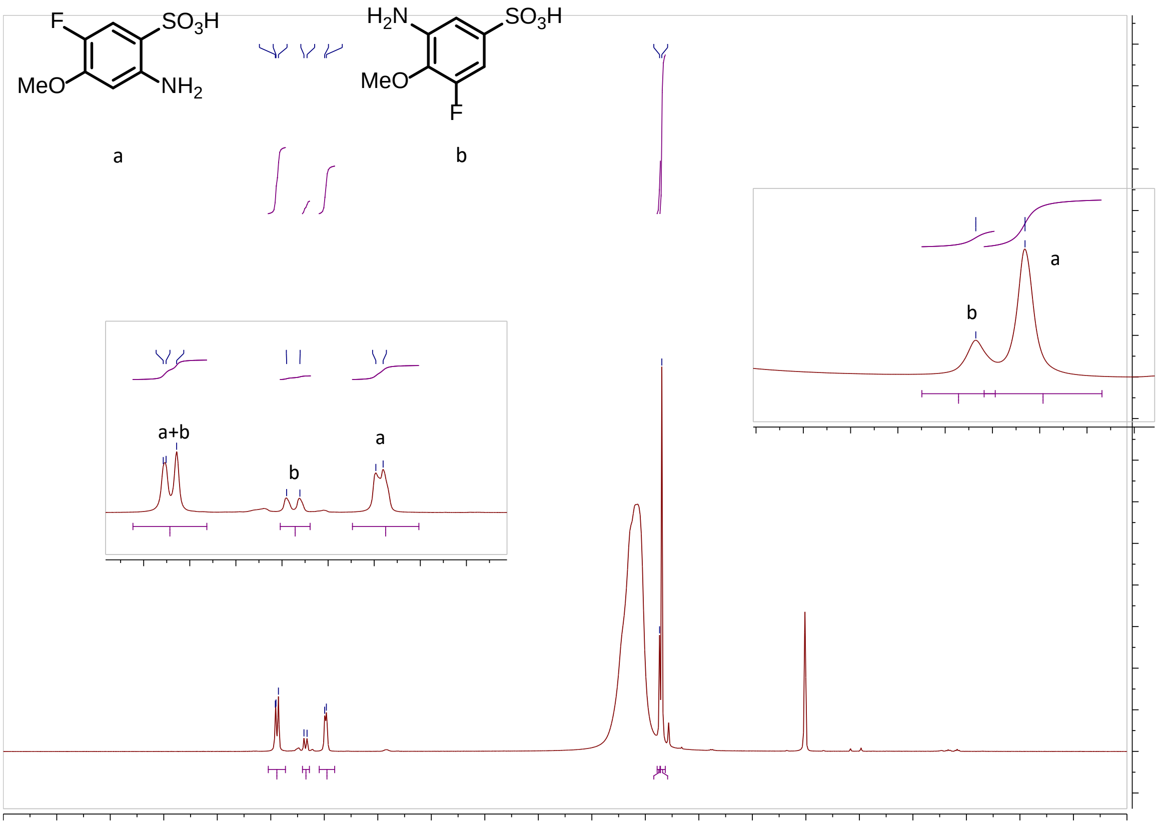

126 MHz, DMSO-d_6

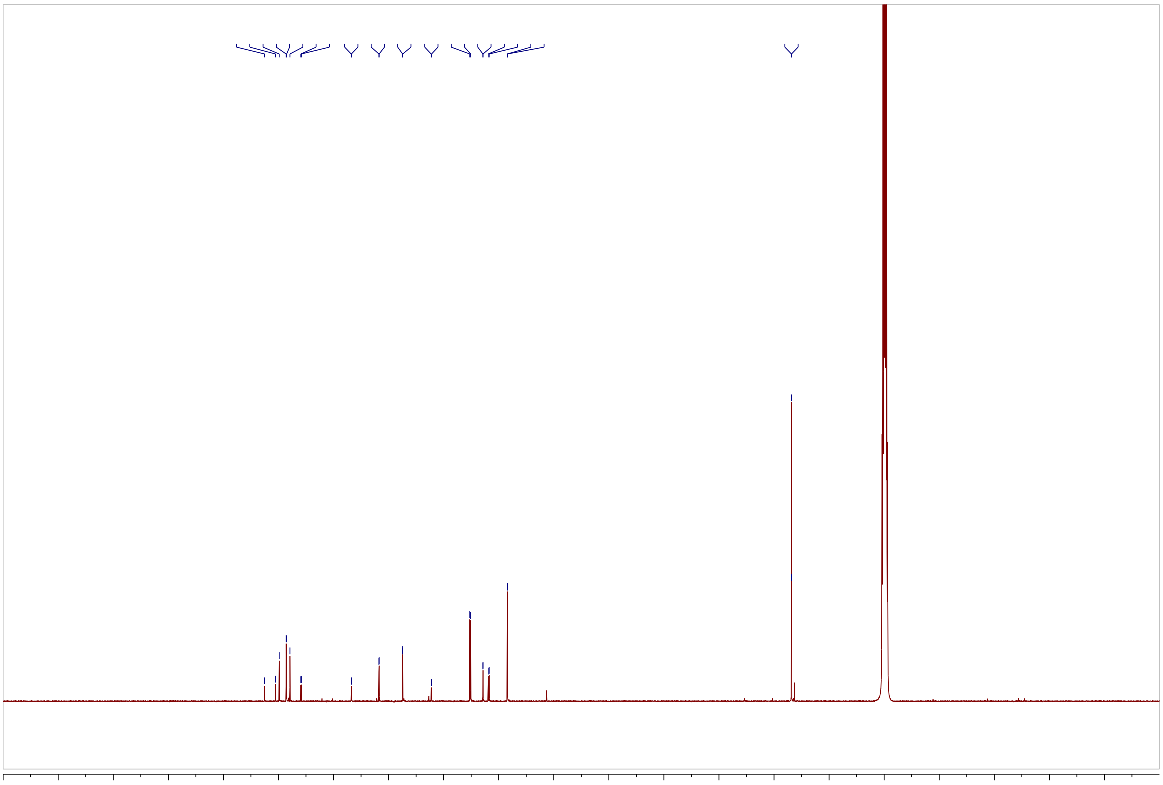

471 MHz, DMSO-d_6

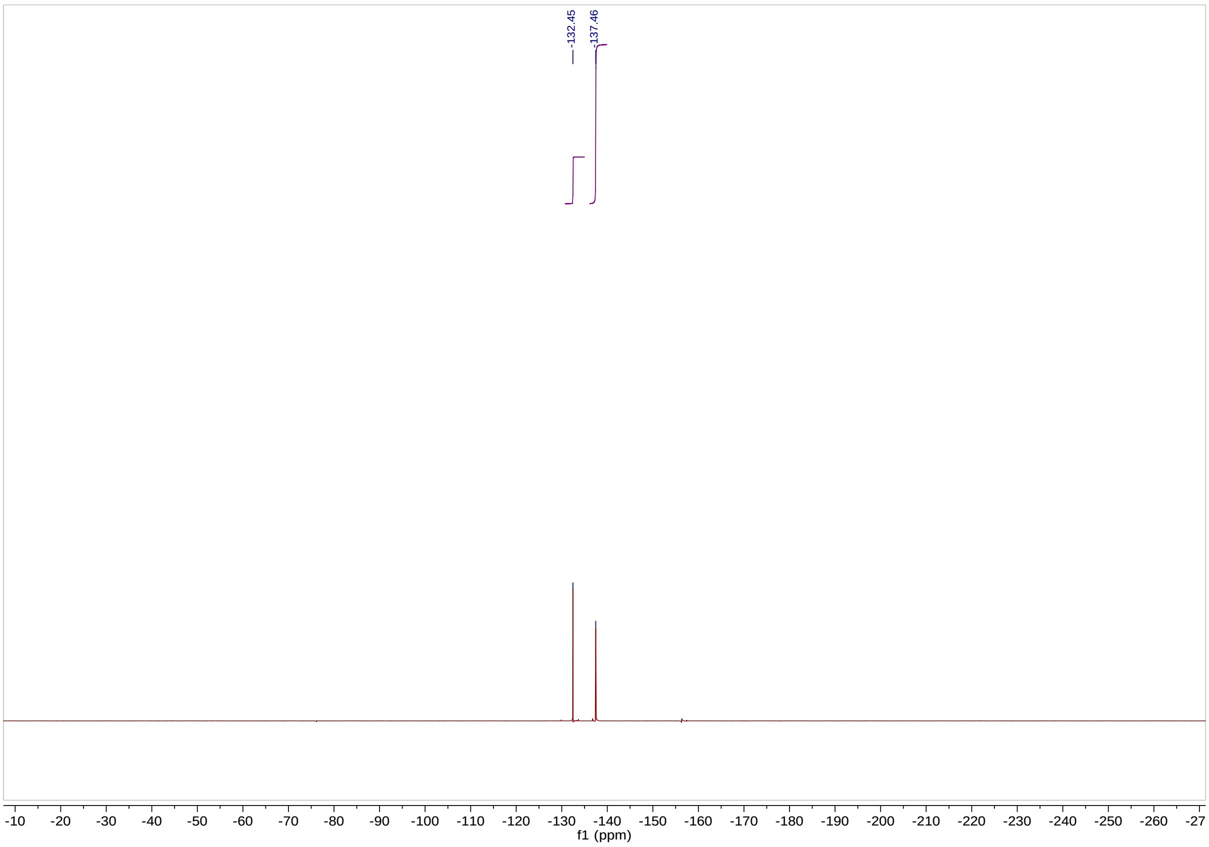

2-Amino-4,5-dimethoxybenzenesulfonic acid 2v

400 MHz, DMSO-d_6

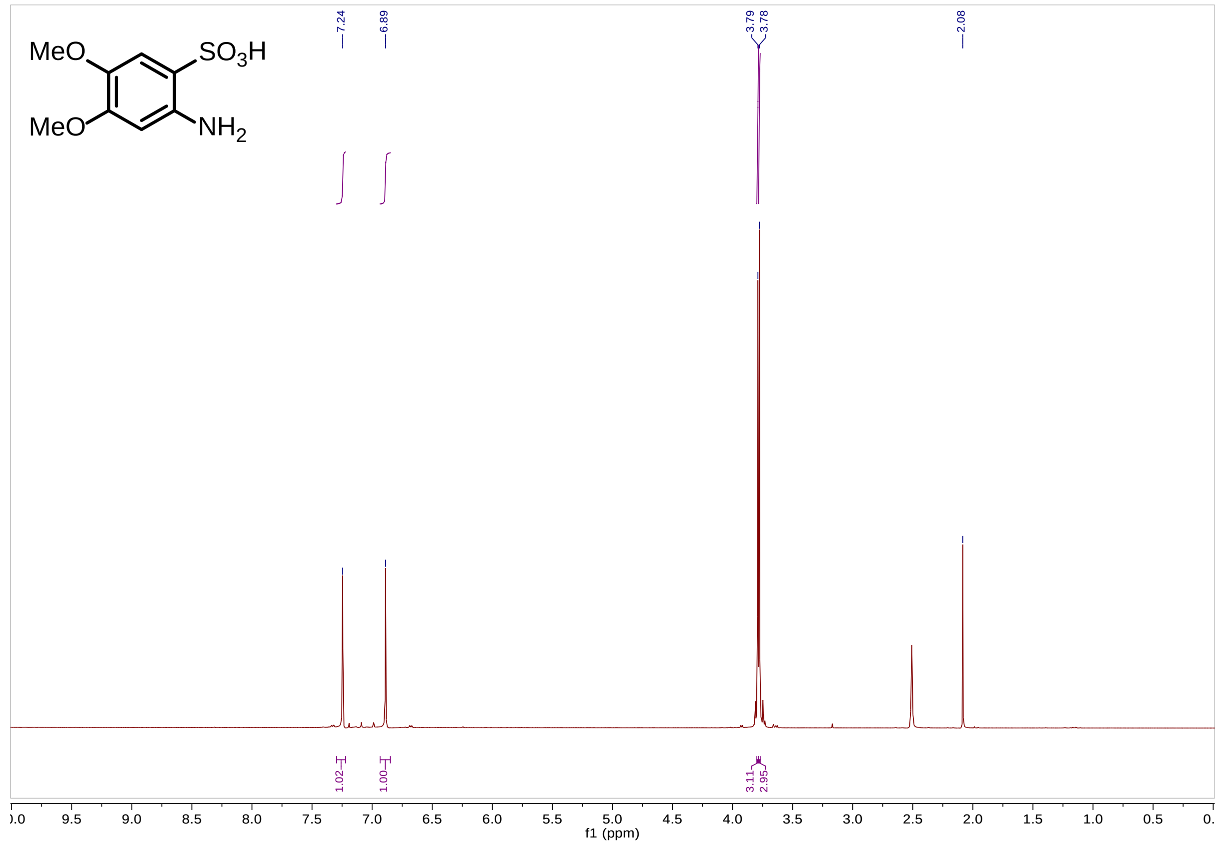

101 MHz, DMSO-d_6

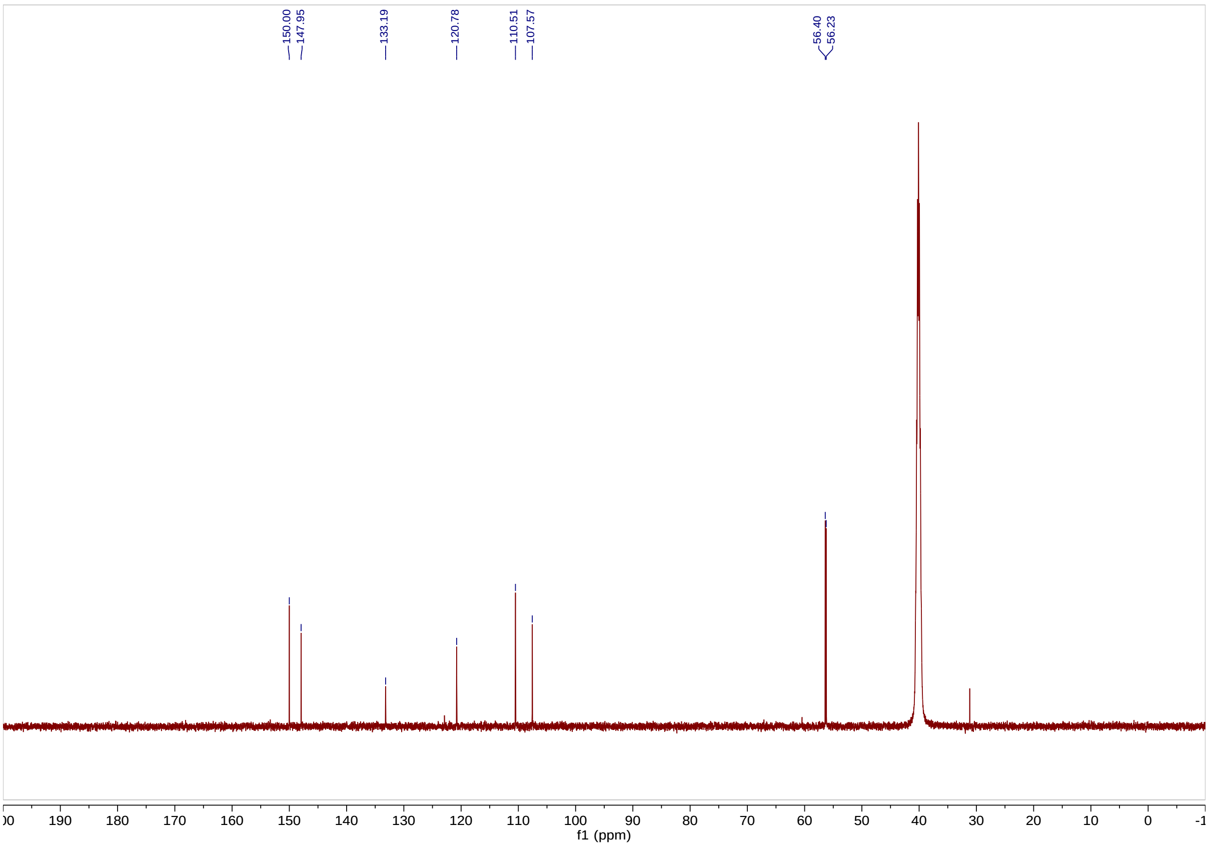

3-Aminonaphthalene-2-sulfonic acid 2w

500 MHz, DMSO-d_6

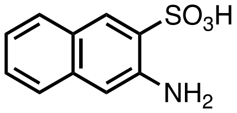

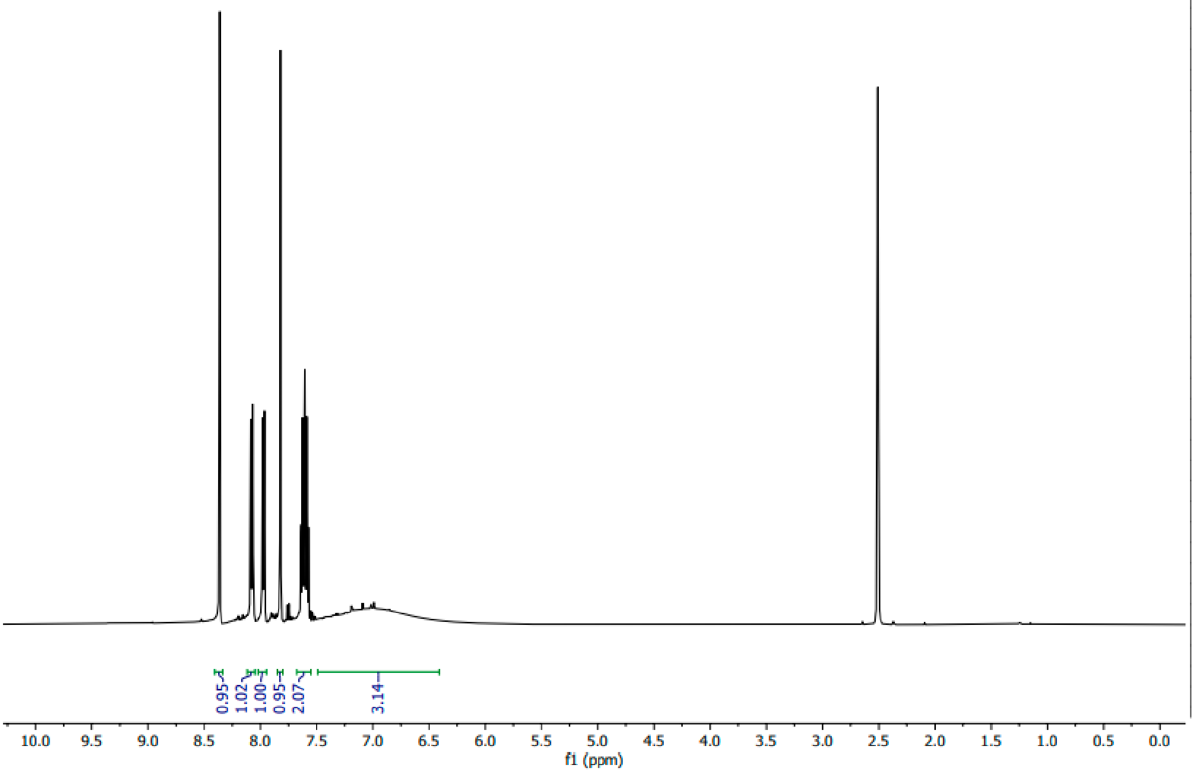

126 MHz, DMSO-d_6

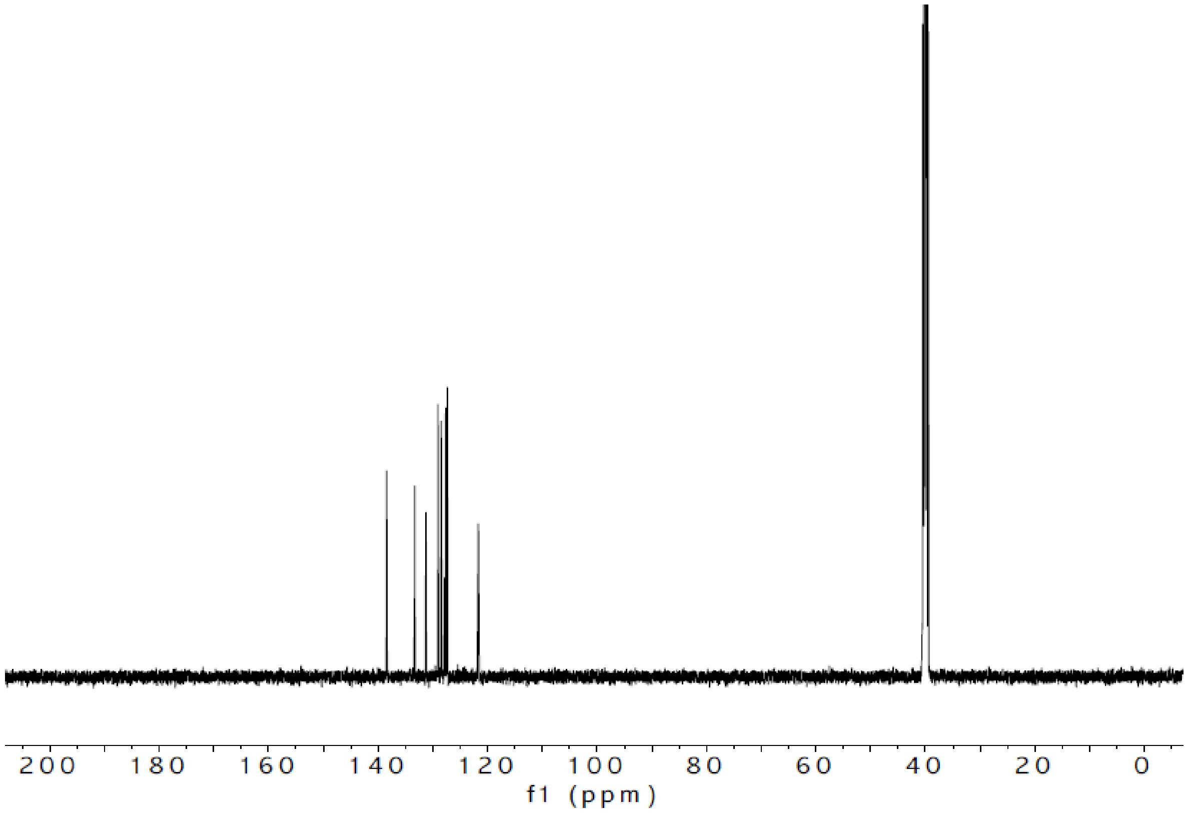

2-Amino-5-(trifluoromethoxy)benzenesulfonic acid 2x

500 MHz, DMSO-d_6

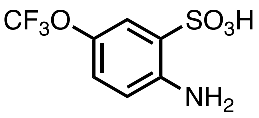

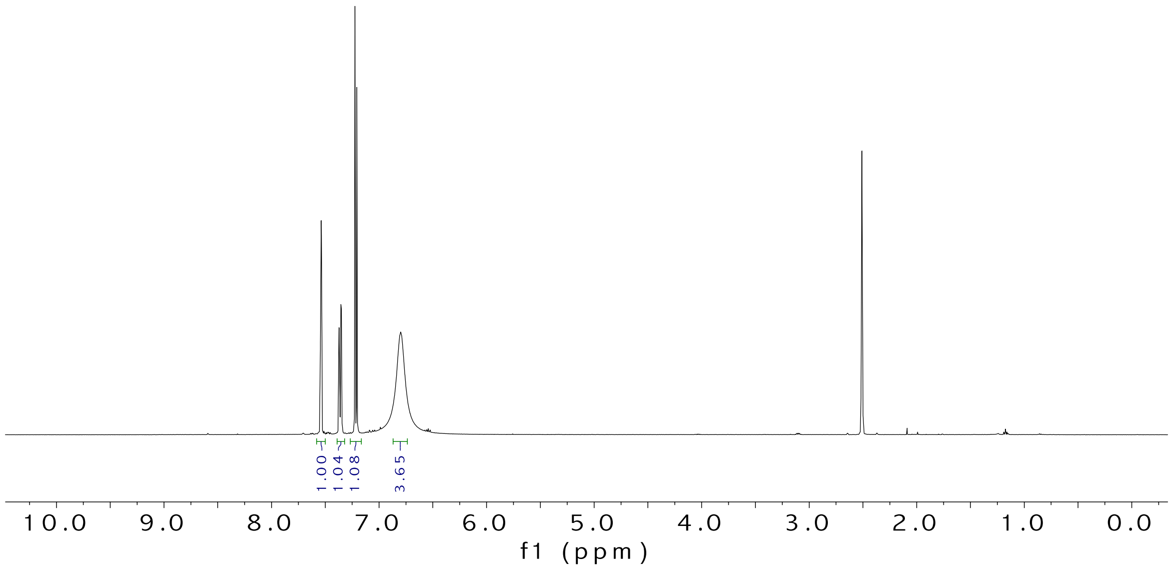

126 MHz, DMSO-d_6

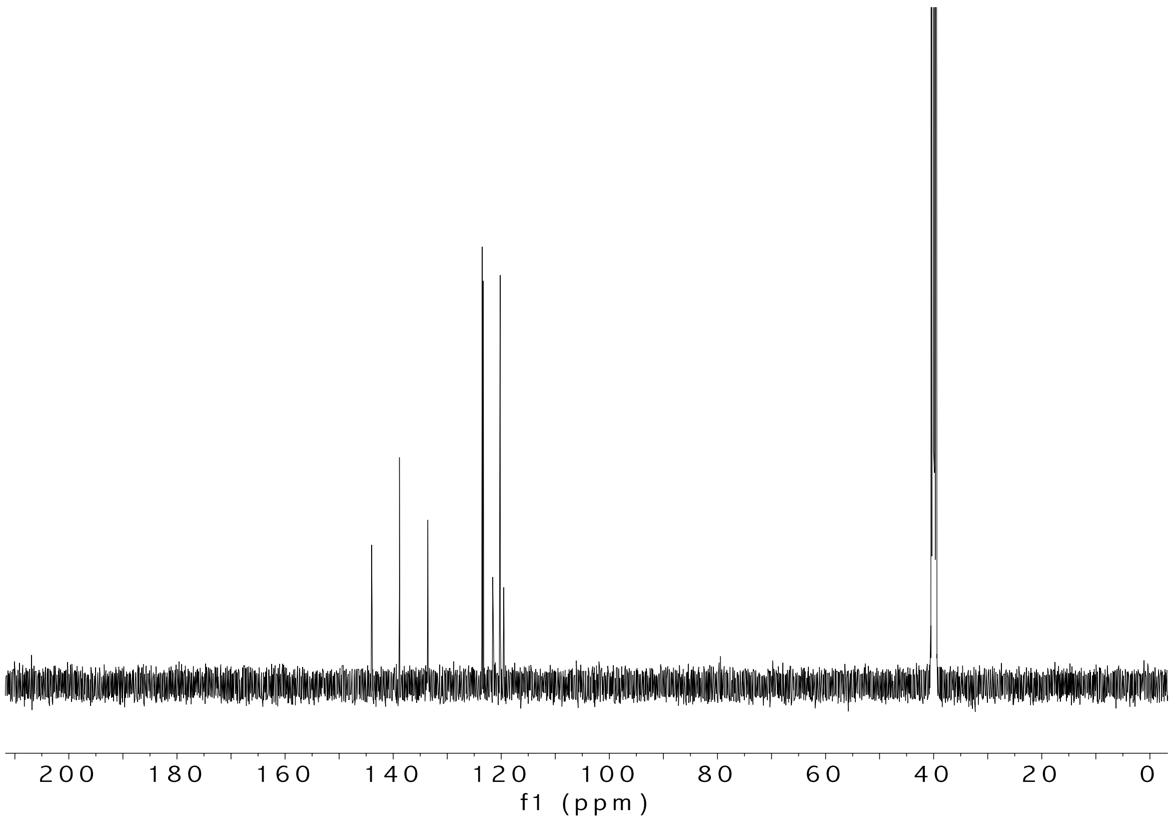

376 MHz, DMSO-d_6

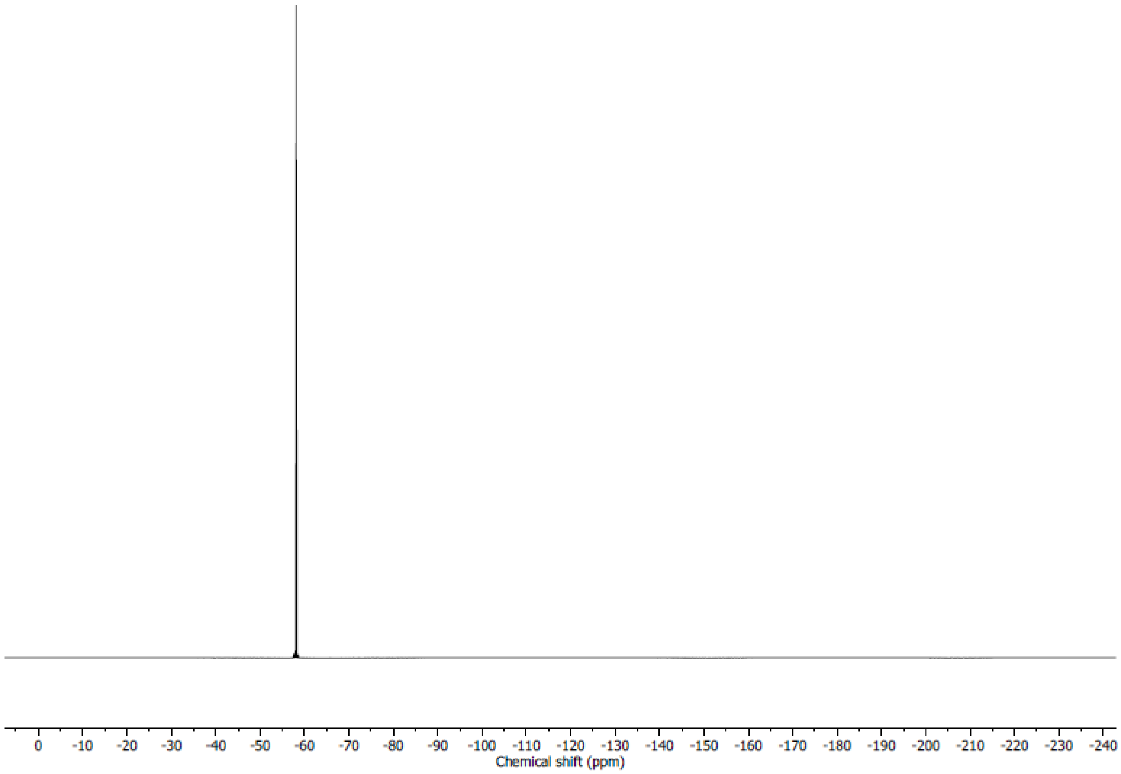

2-Amino-4-(trifluoromethyl)benzenesulfonic acid 2y

500 MHz, DMSO-d_6

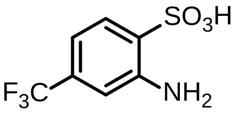

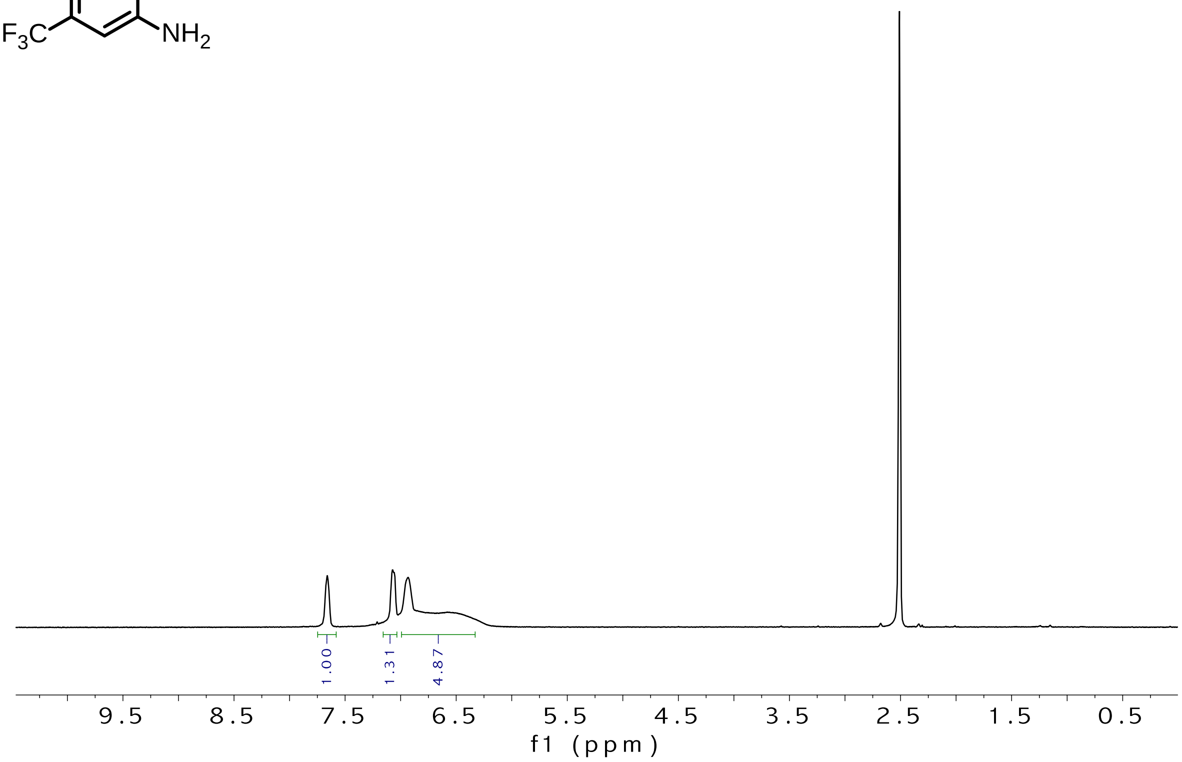

126 MHz, DMSO-d_6

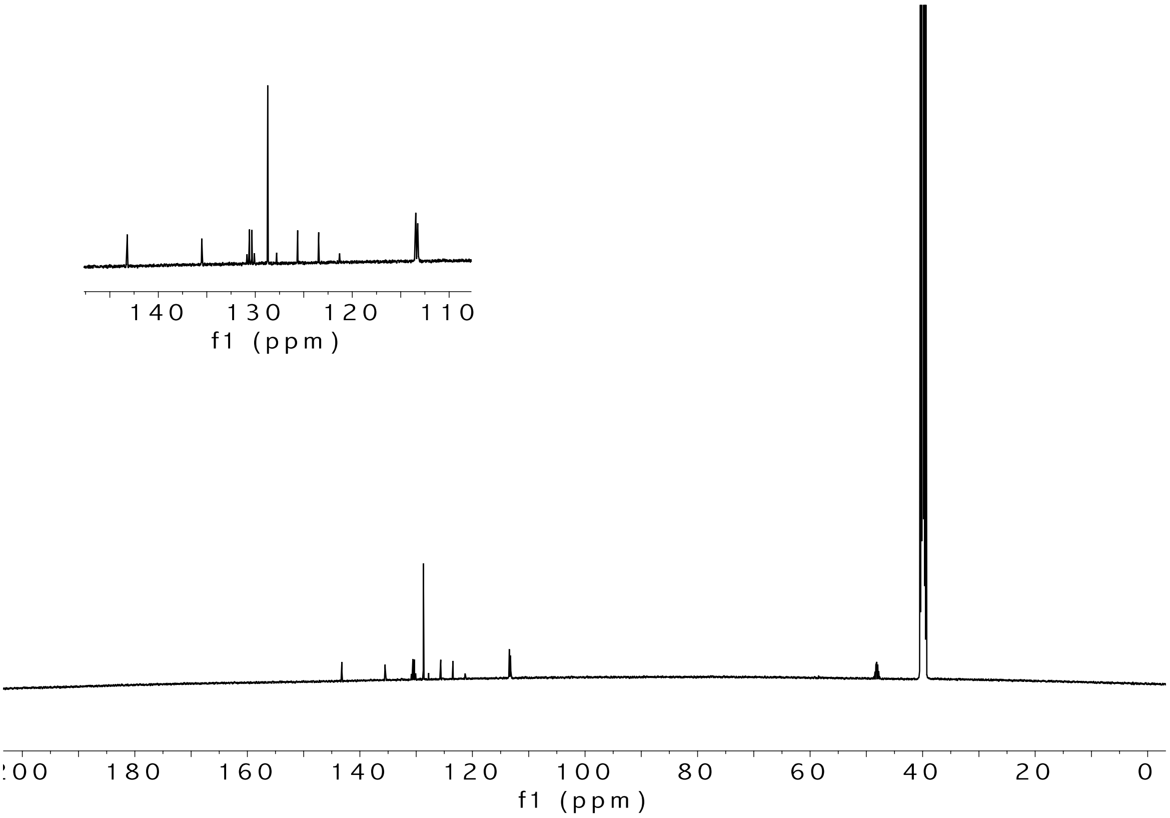

471 MHz, DMSO-*d*$_6$

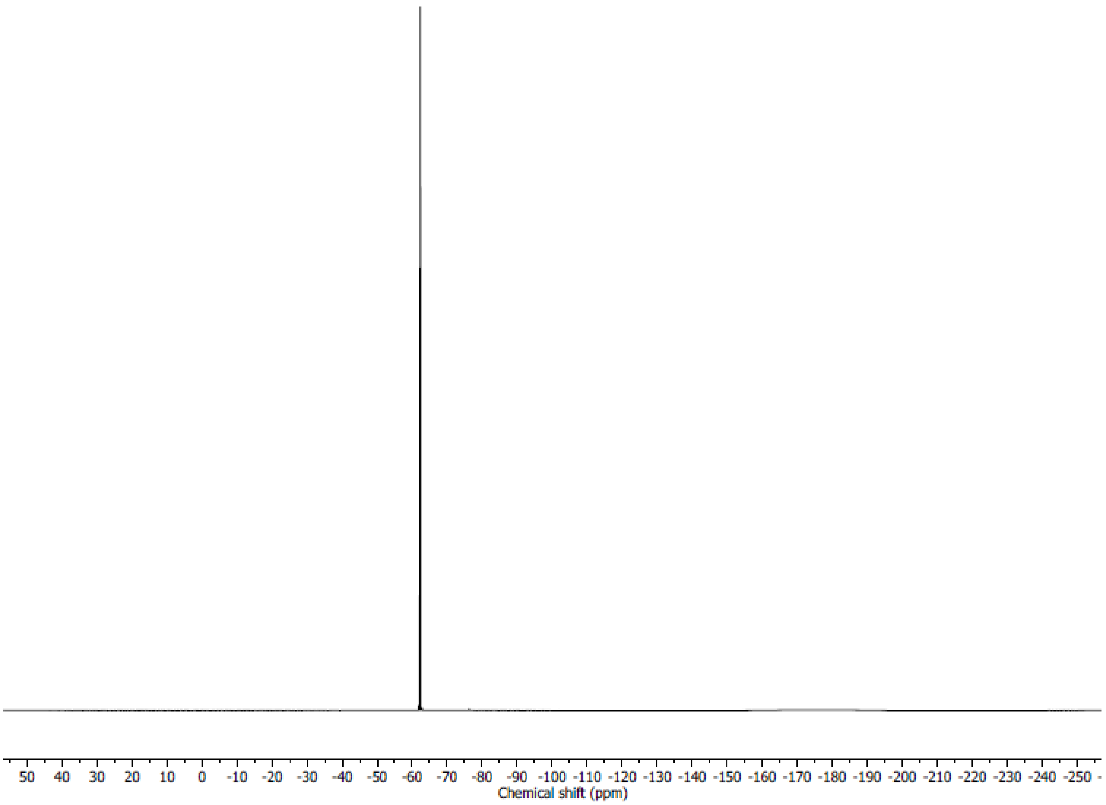

2-Amino-4-(methoxycarbonyl)benzenesulfonic acid 2z

500 MHz DMSO-d_6

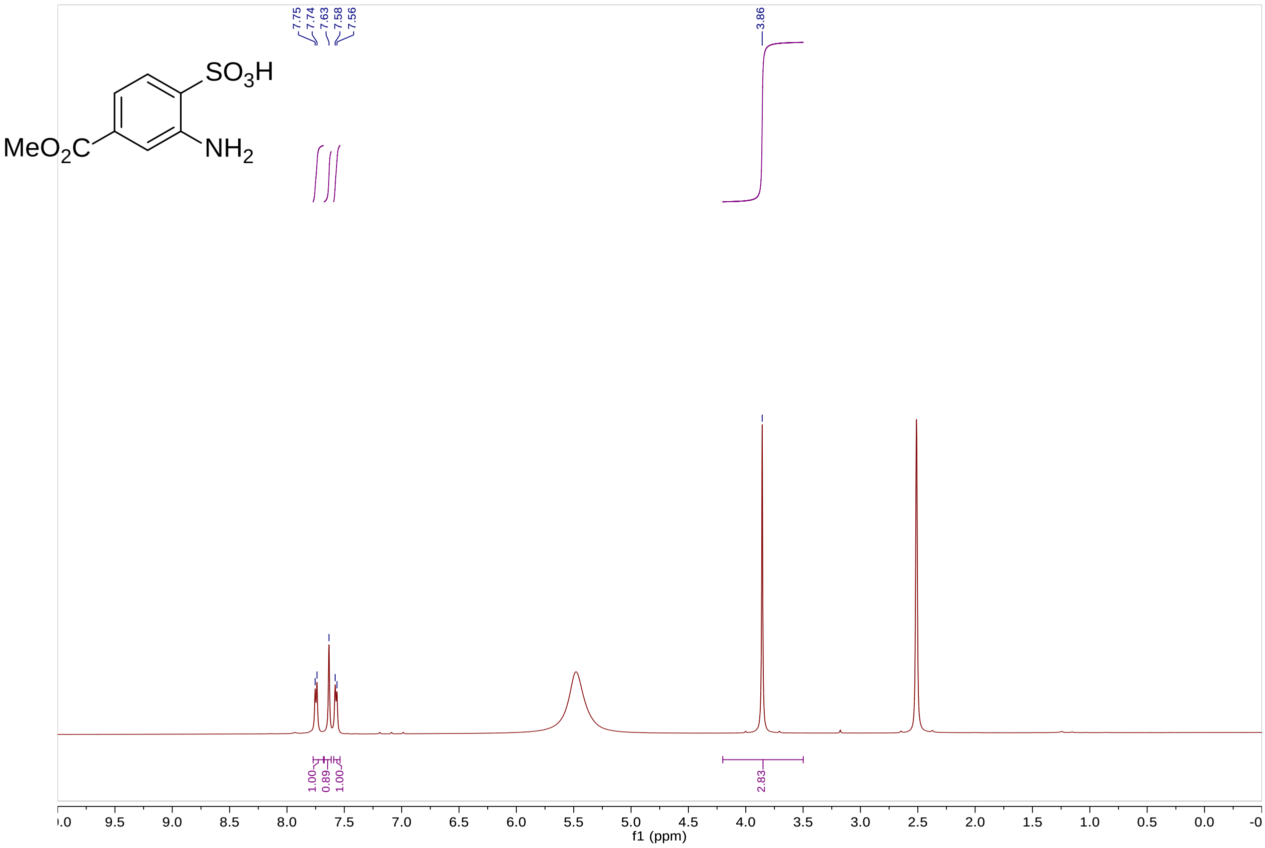

126 MHz DMSO-d_6

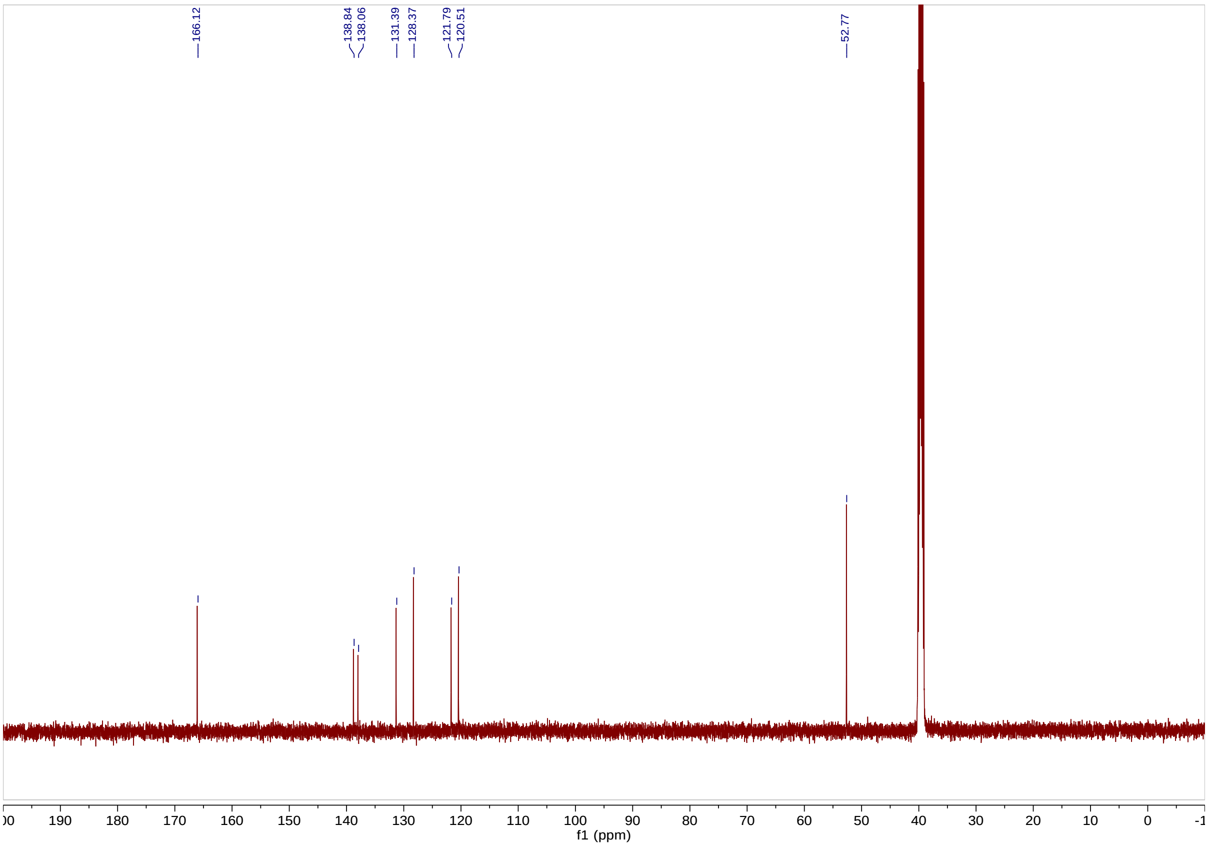

2-Amino-6-(trifluoromethyl)benzenesulfonic acid 2za

500 MHz, DMSO-d_6

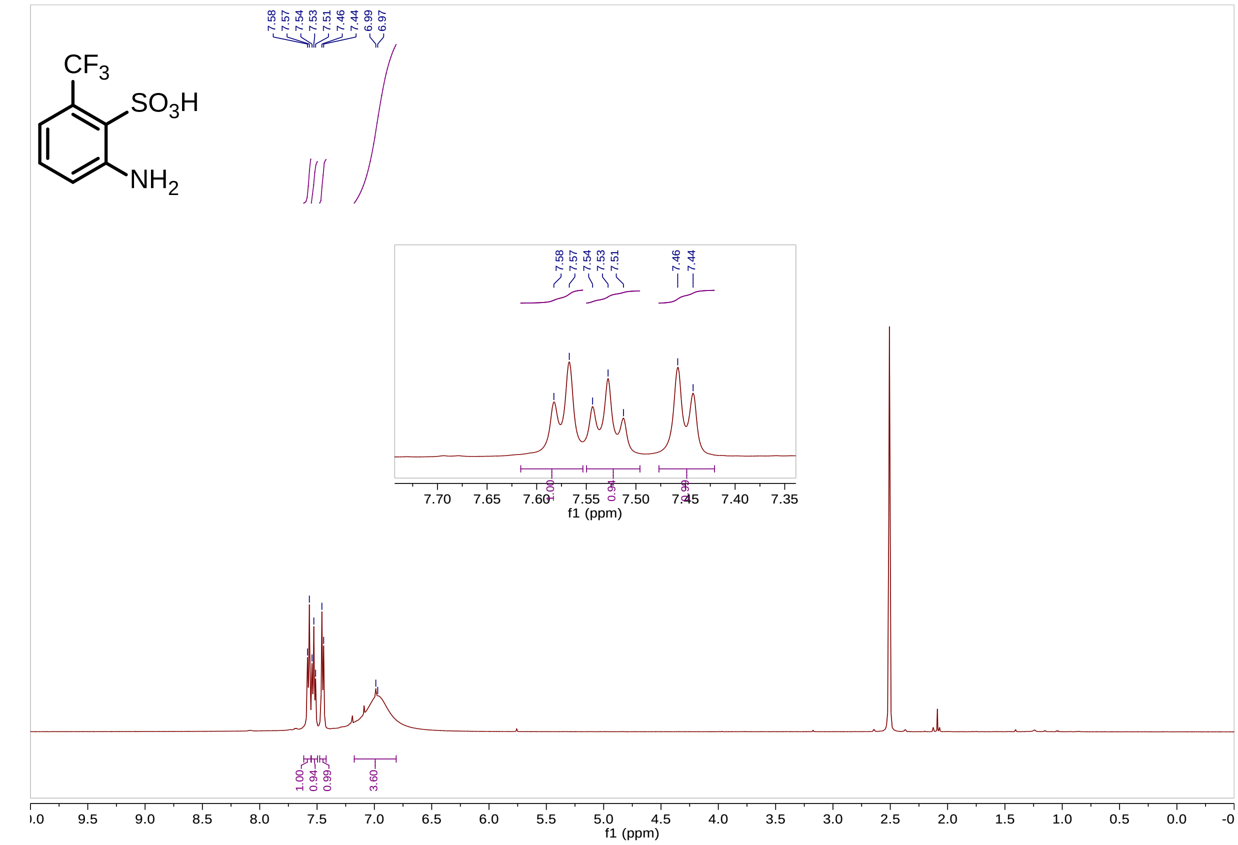

126 MHz, DMSO-d_6

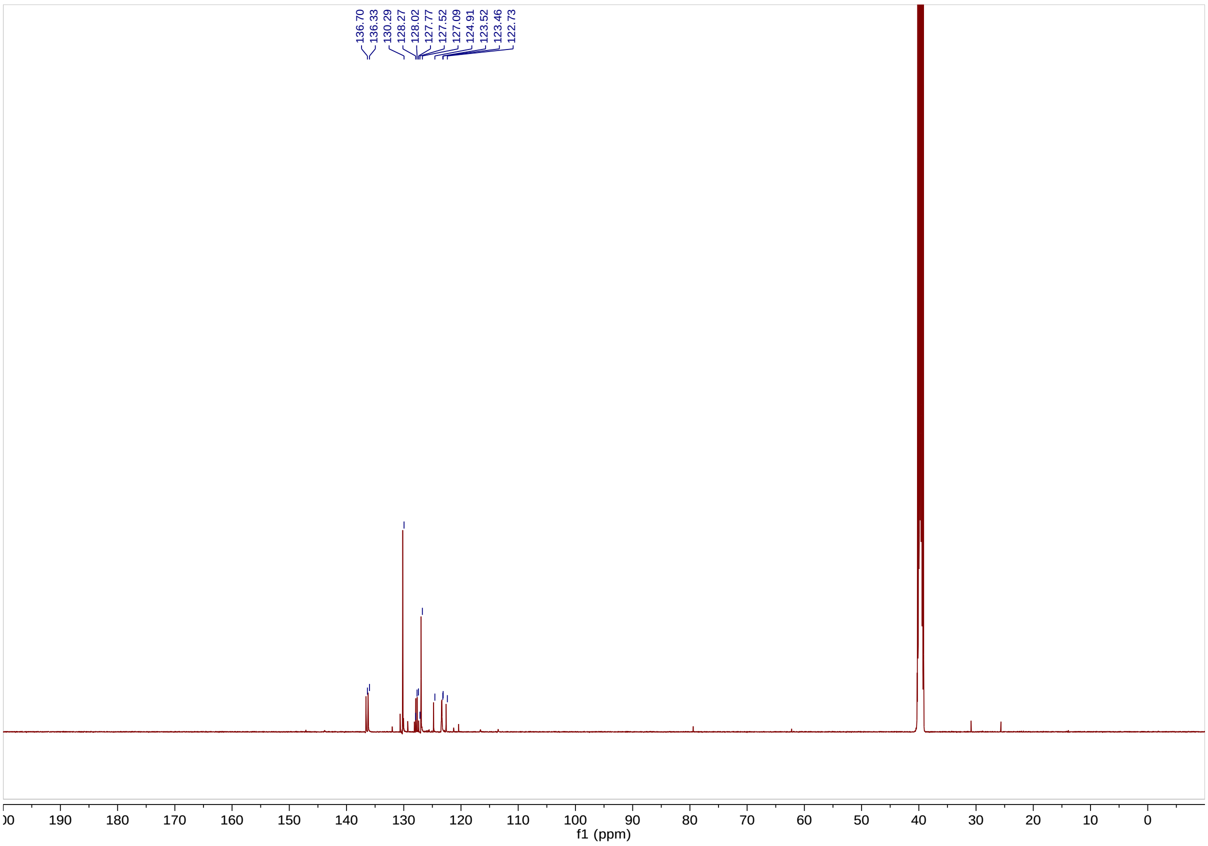

471 MHz, DMSO-d_6

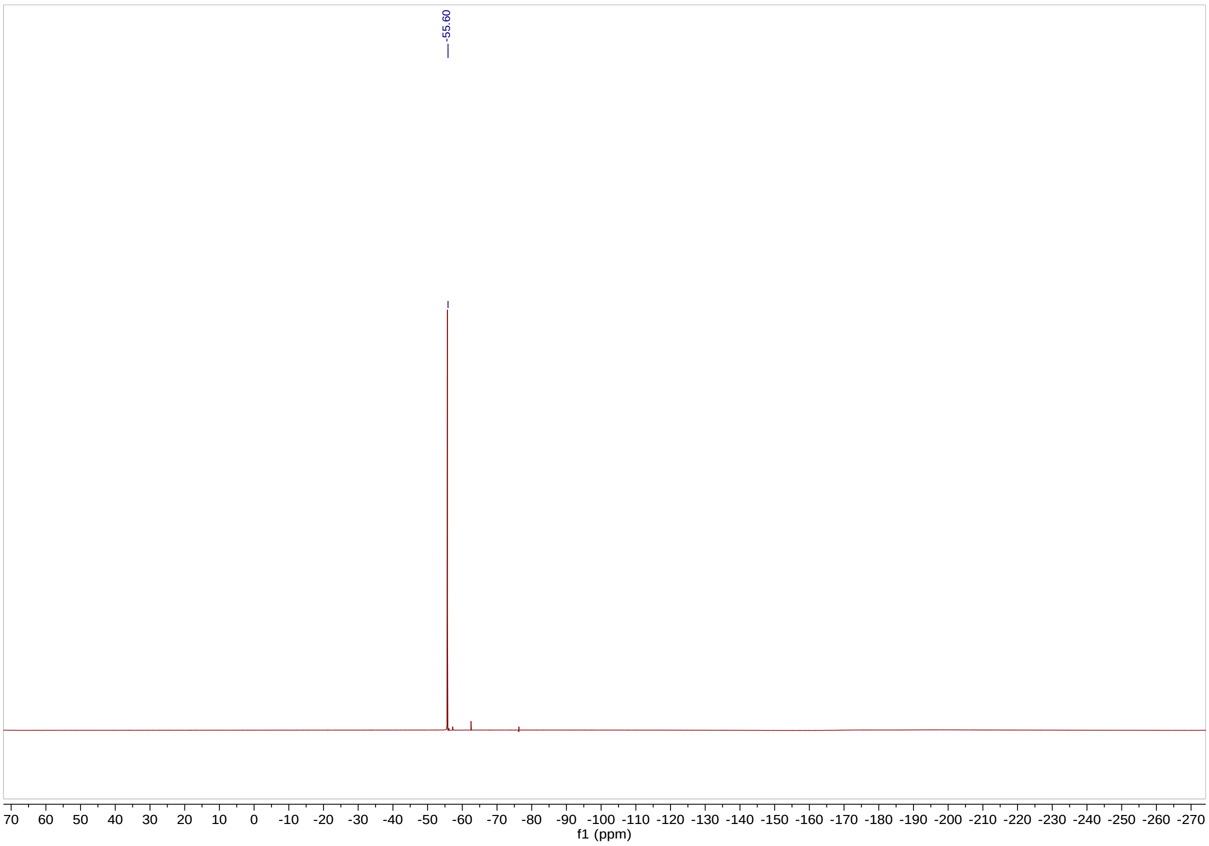

2-Amino-5-(methylsulfonyl)benzenesulfonic acid 2zb

400 MHz, DMSO-d_6

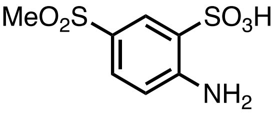

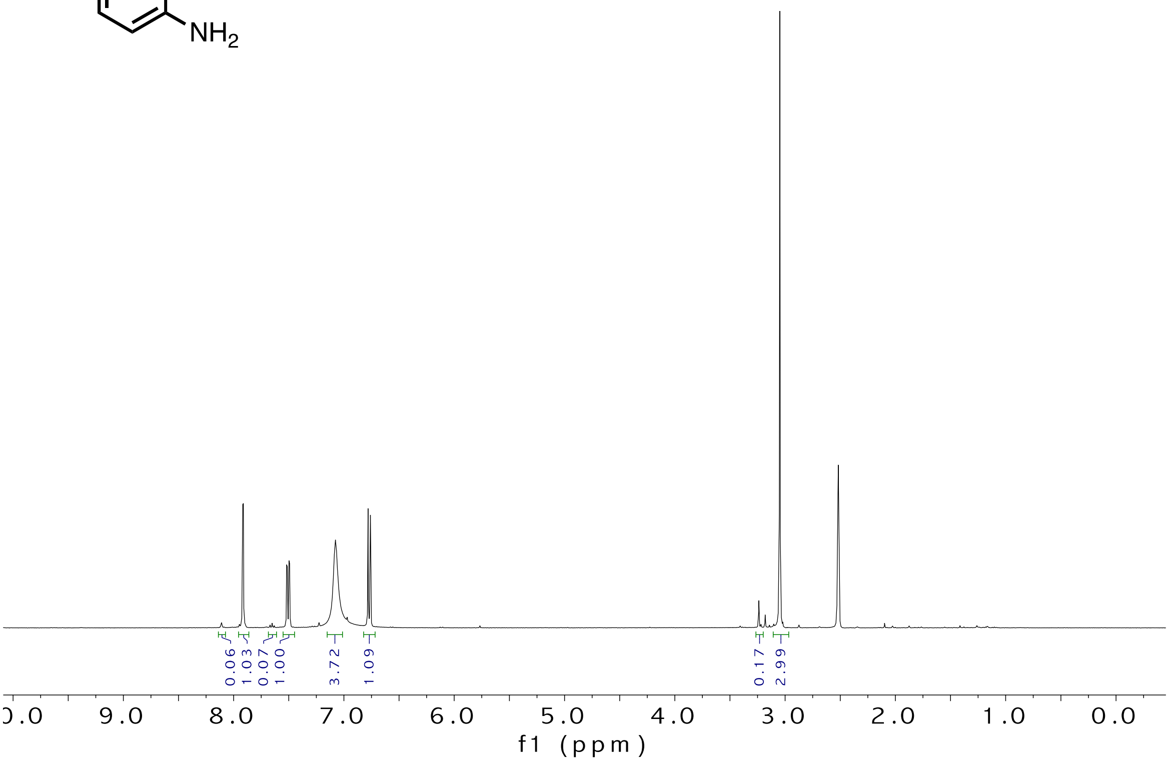

101 MHz, DMSO-d_6

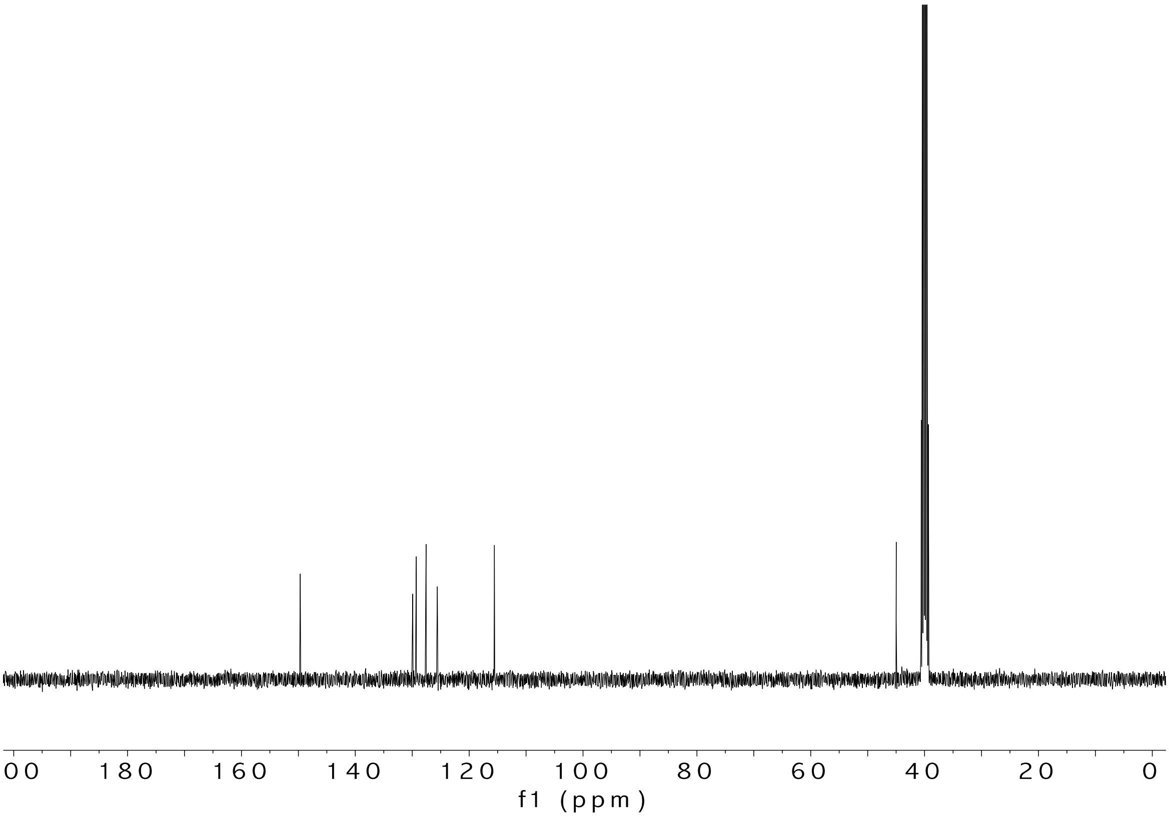

2-Amino-4-nitrobenzenesulfonic acid 2zc

400 MHz, DMSO-*d*$_6$

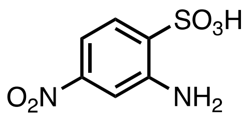

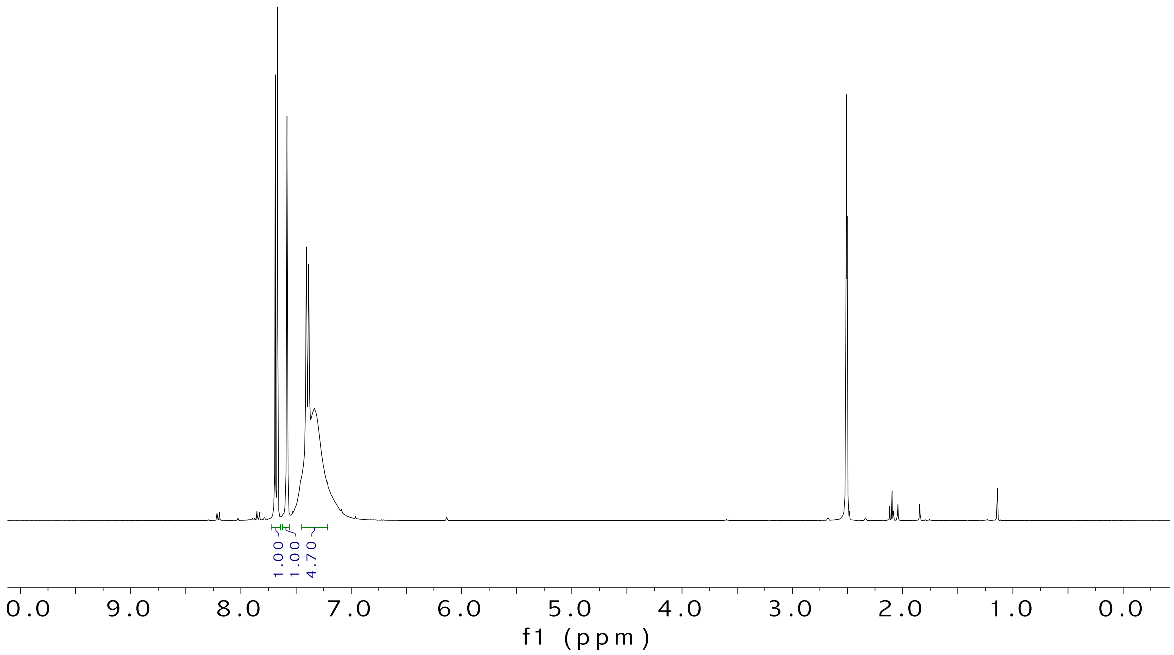

101 MHz, DMSO-*d*$_6$

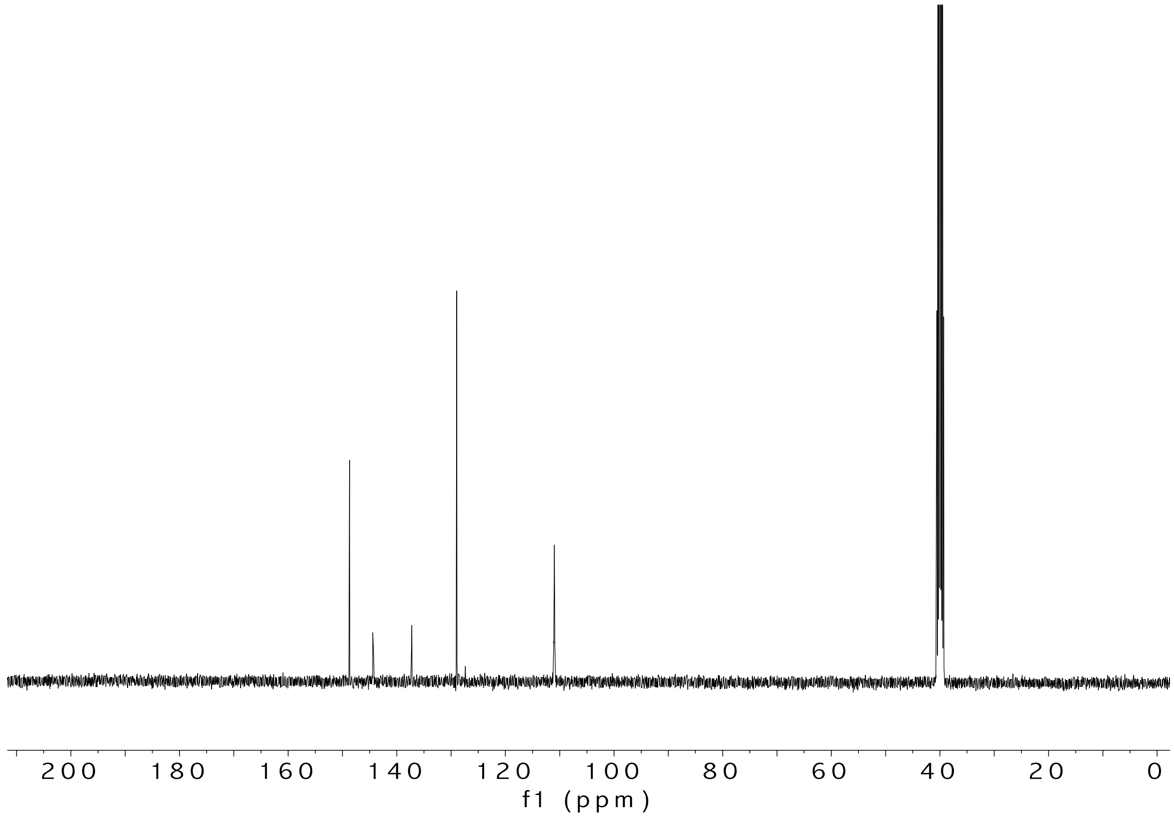

(2-Aminophenyl)methanesulfonic acid 4a

400 MHz, DMSO-d_6

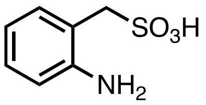

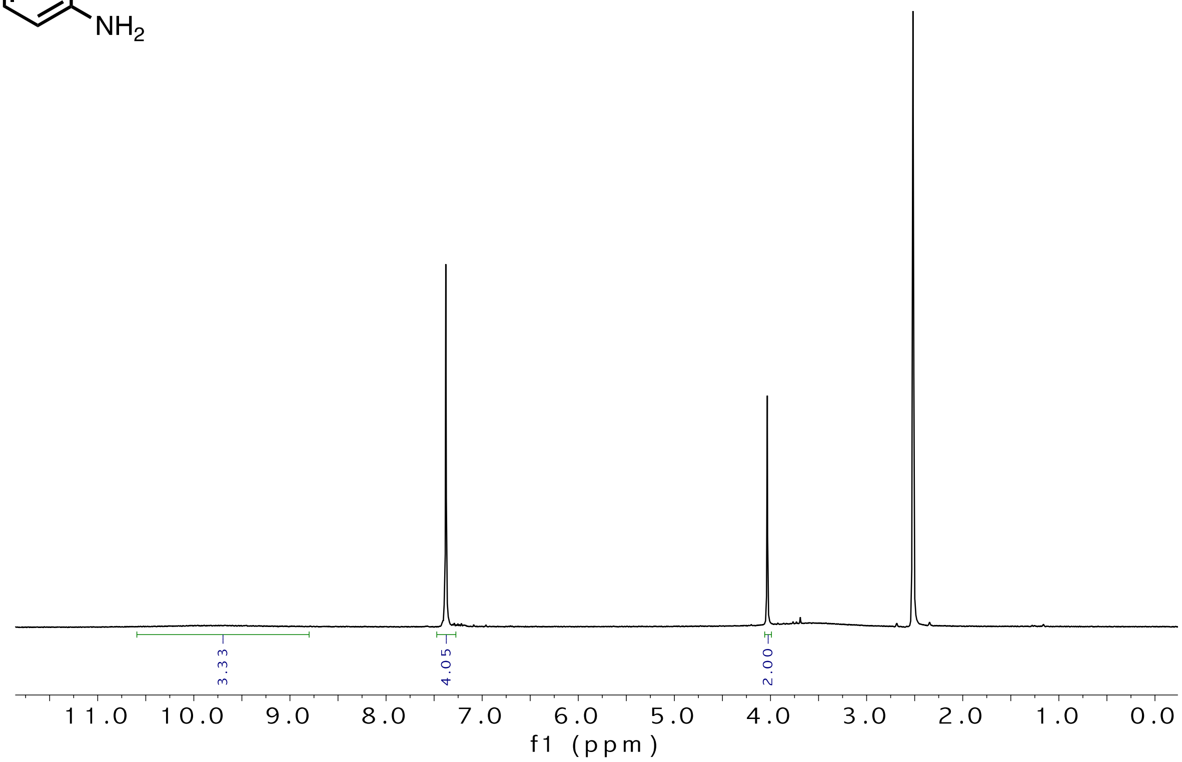

101 MHz, DMSO-d_6

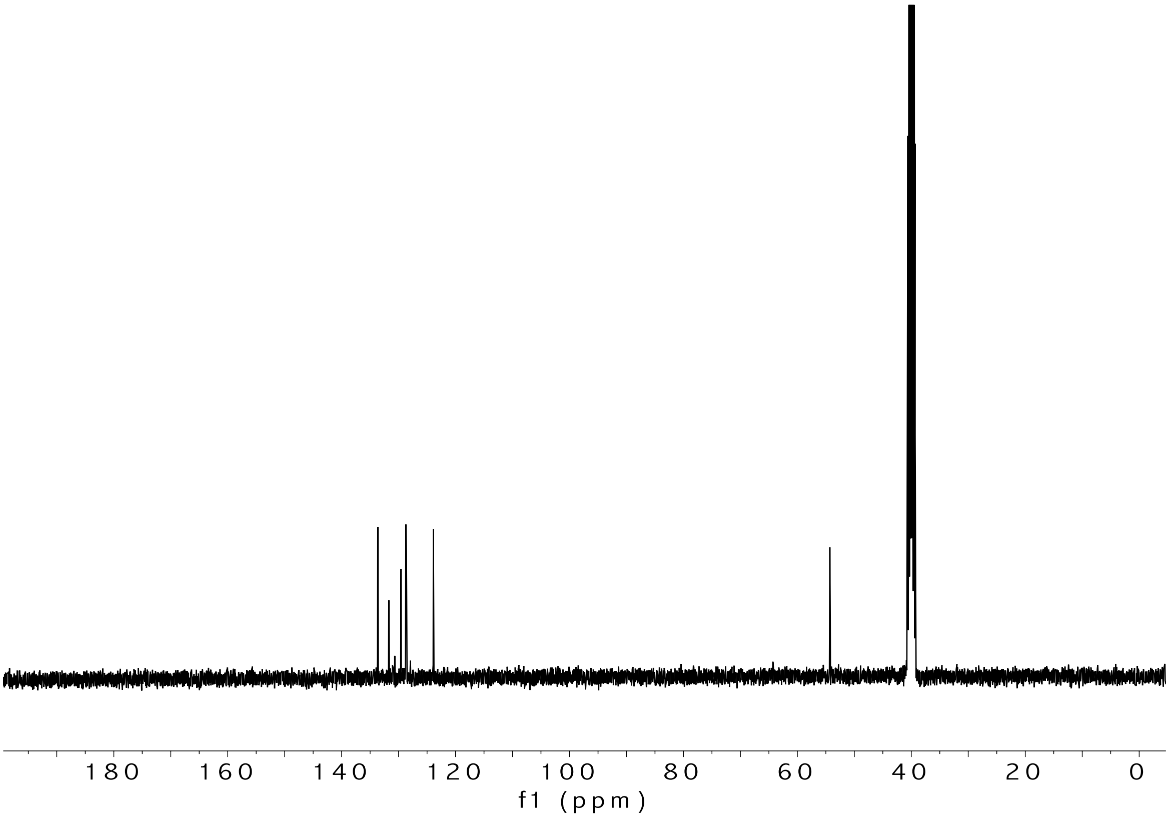

(2-Amino-4-chlorophenyl)methanesulfonic acid 4b

500 MHz, DMSO-d_6

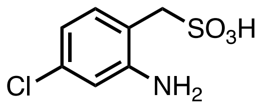

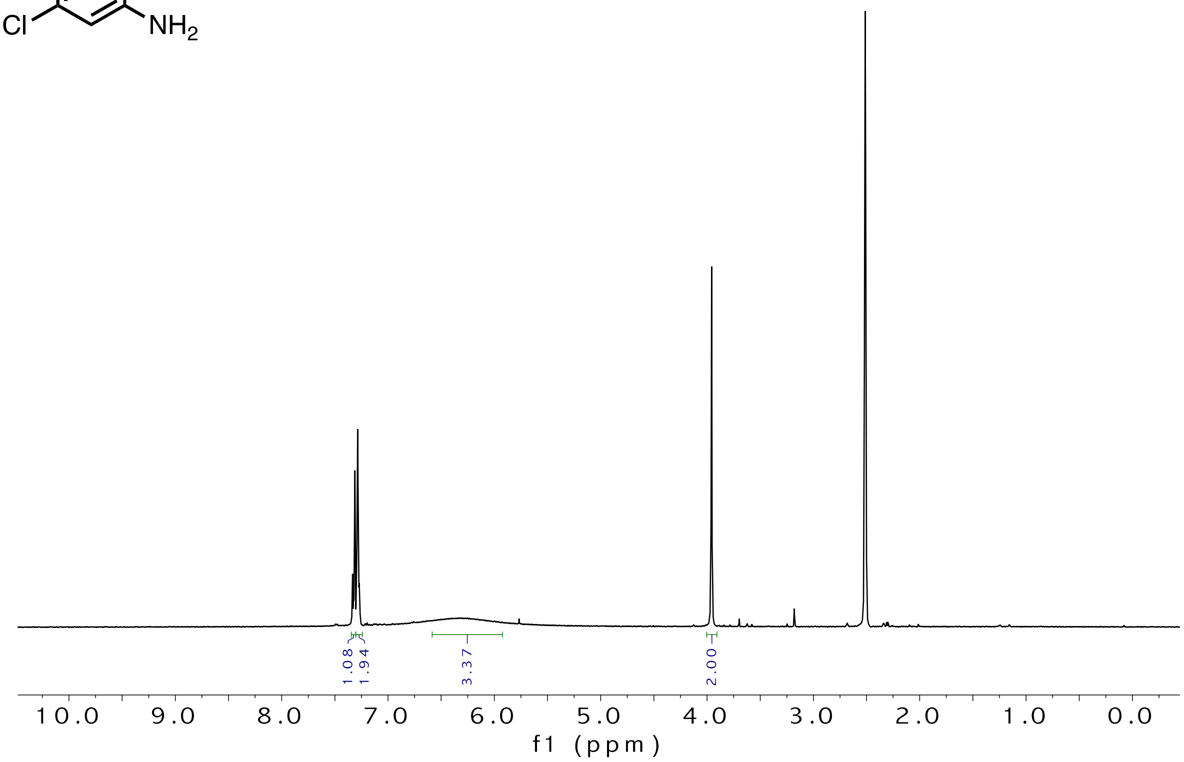

101 MHz, DMSO-d_6

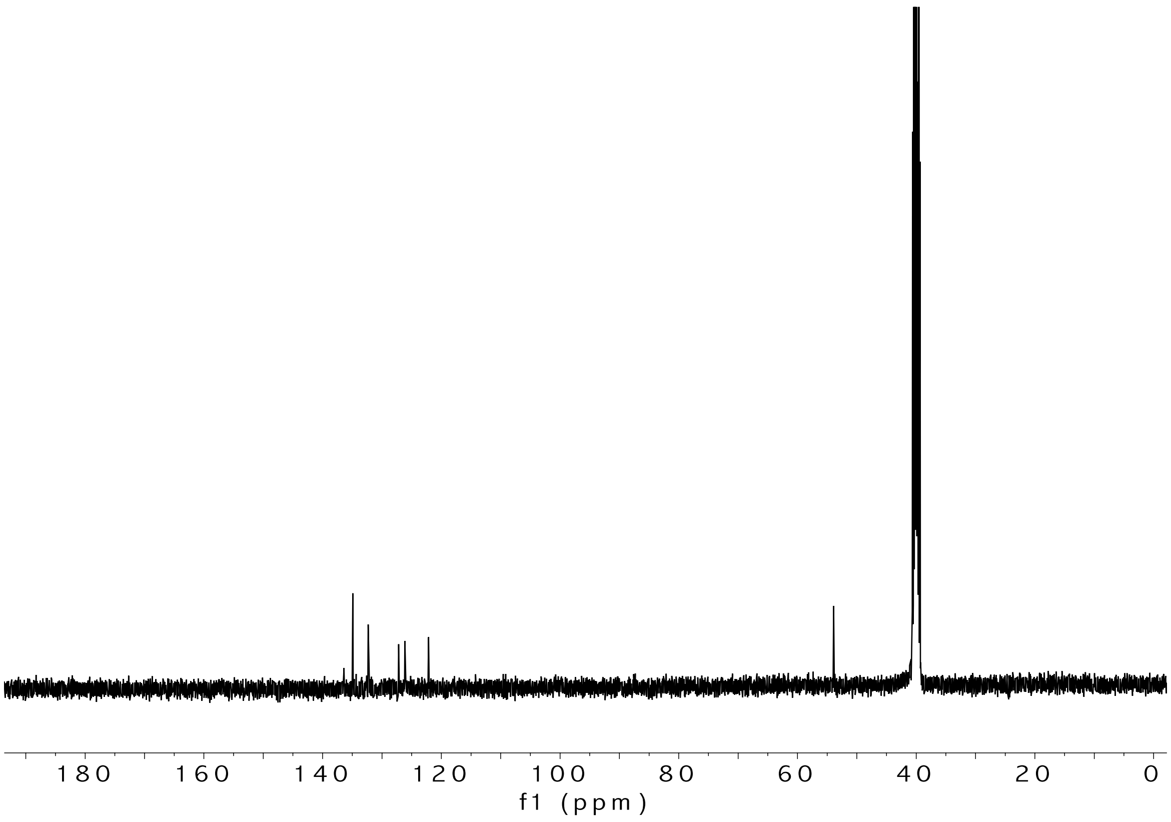

(2-Amino-6-chlorophenyl)methanesulfonic acid 4c

400 MHz, DMSO-d_6

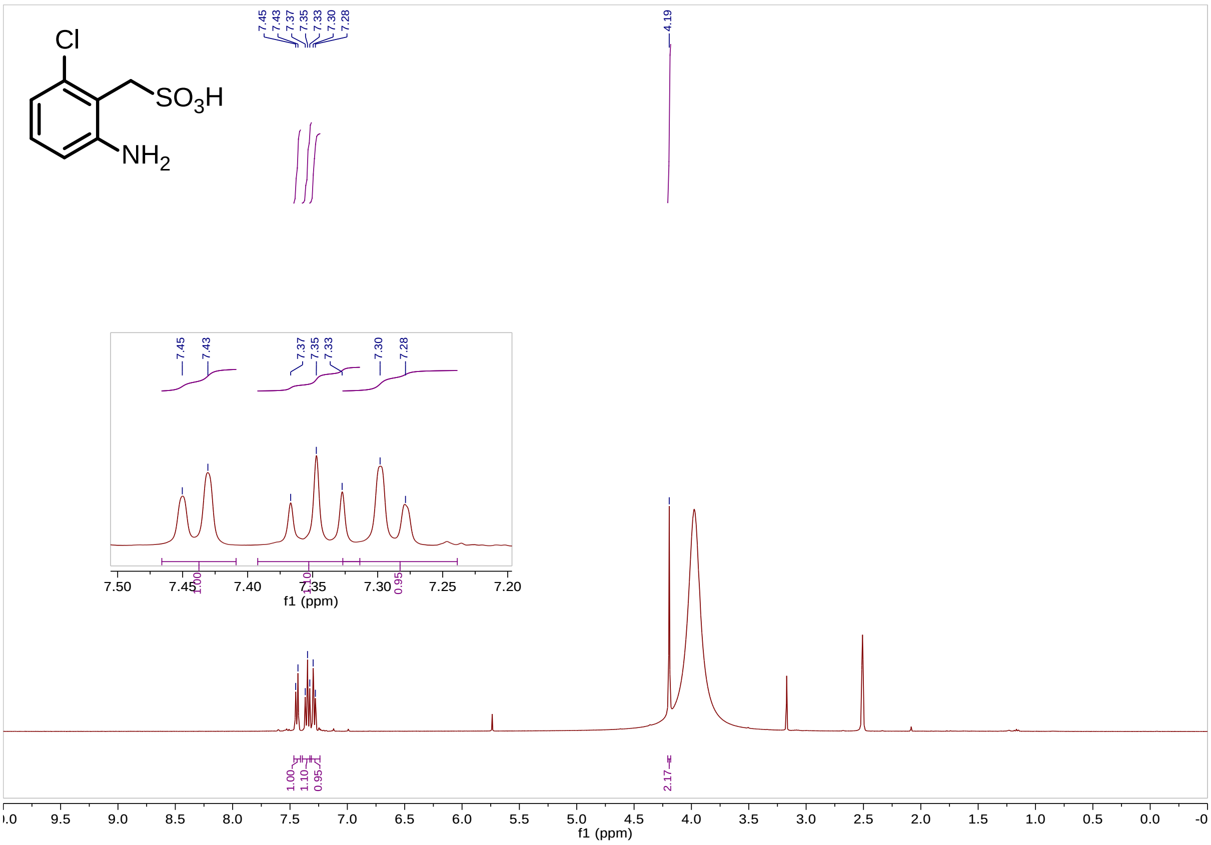

126 MHz, DMSO-d_6

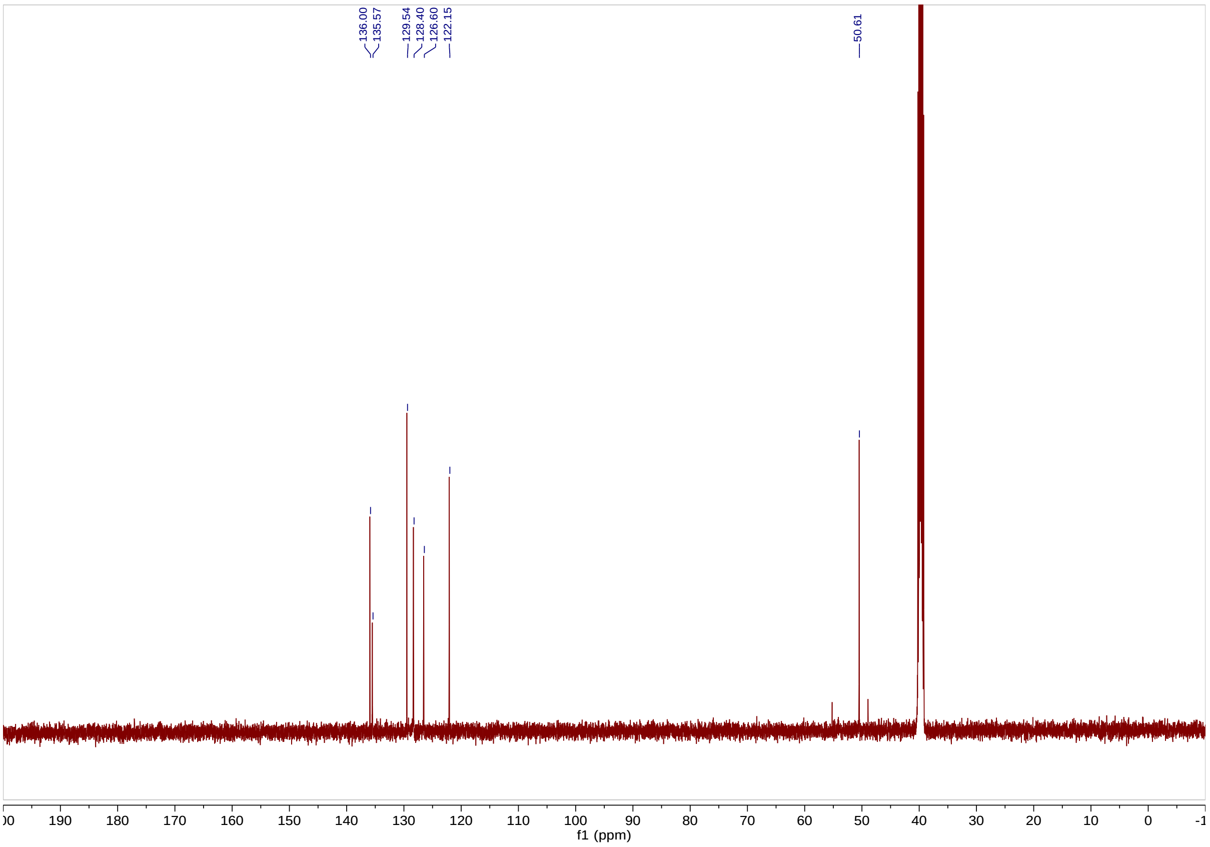

(2-Amino-4-methylphenyl)methanesulfonic acid 4d

400 MHz, DMSO-d_6

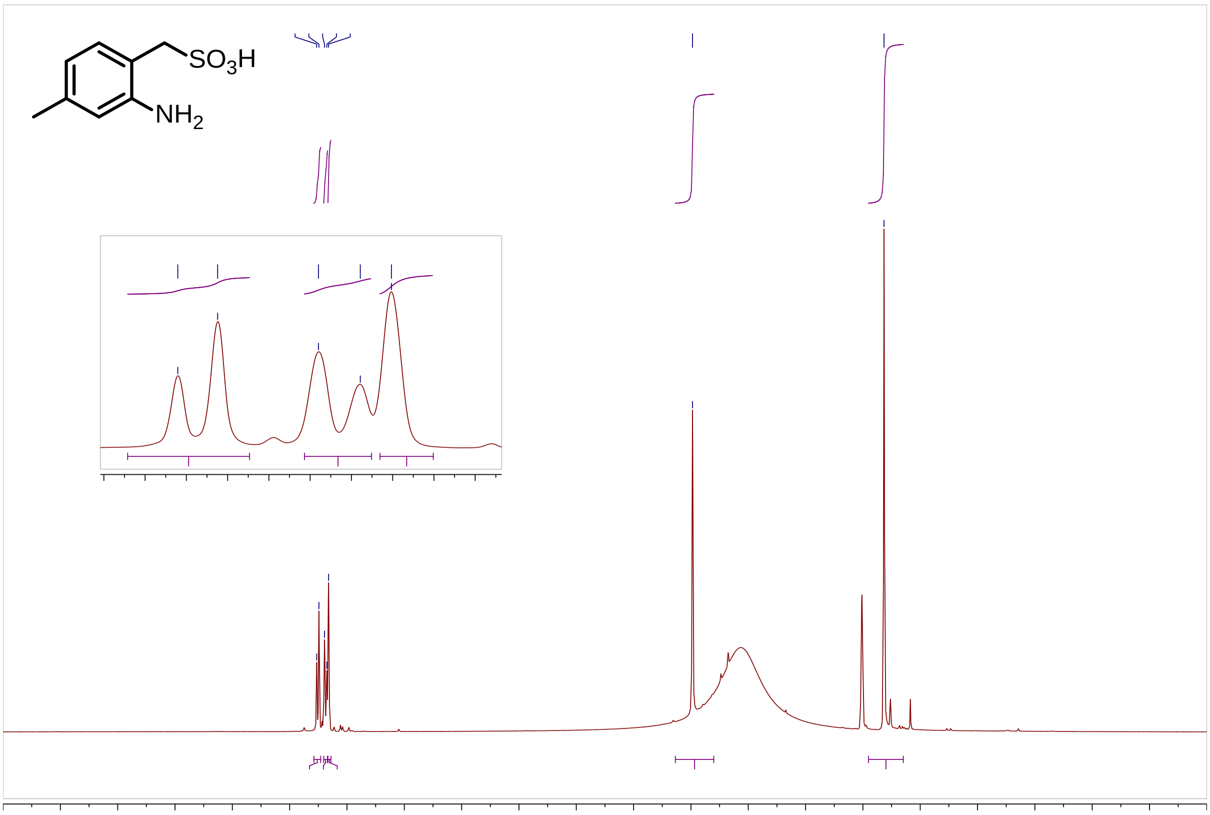

101 MHz, DMSO-d_6

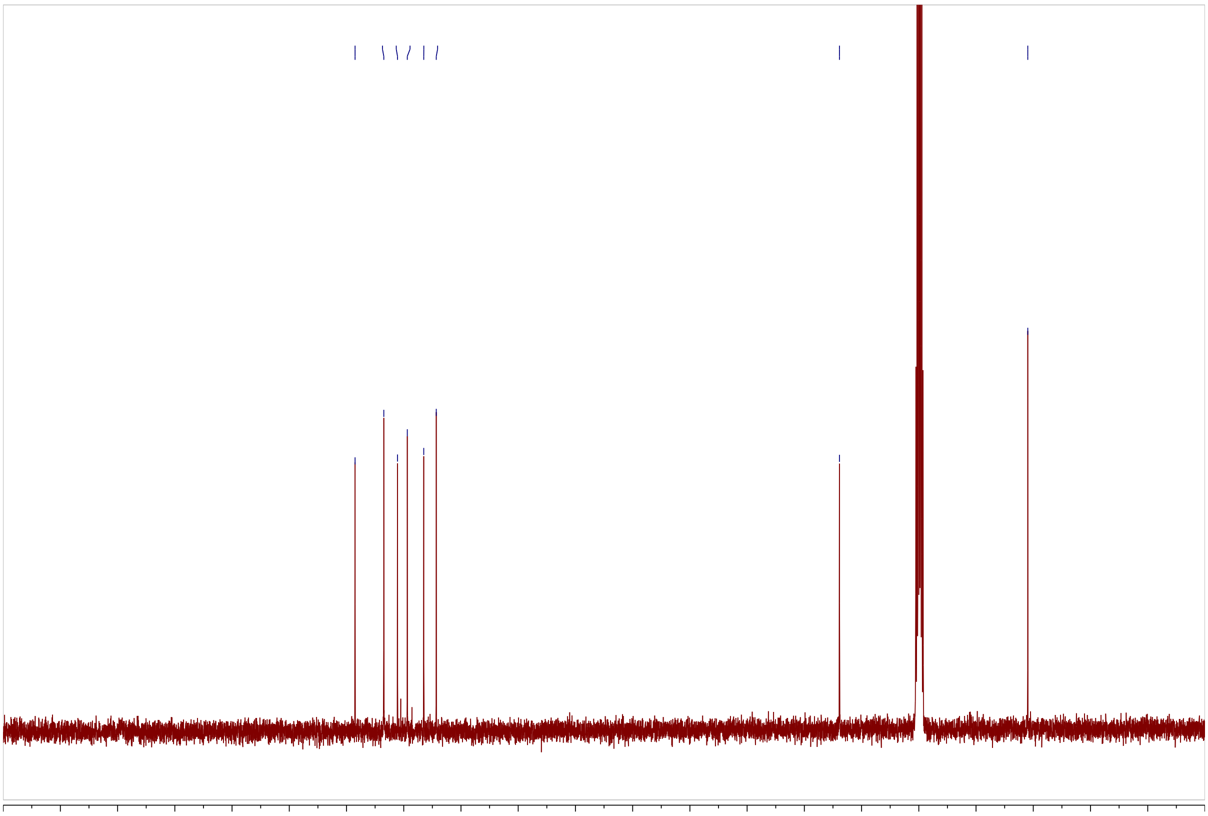

(2-Amino-3,5-dimethoxyphenyl)methanesulfonic acid 4e

500 MHz, DMSO-d_6

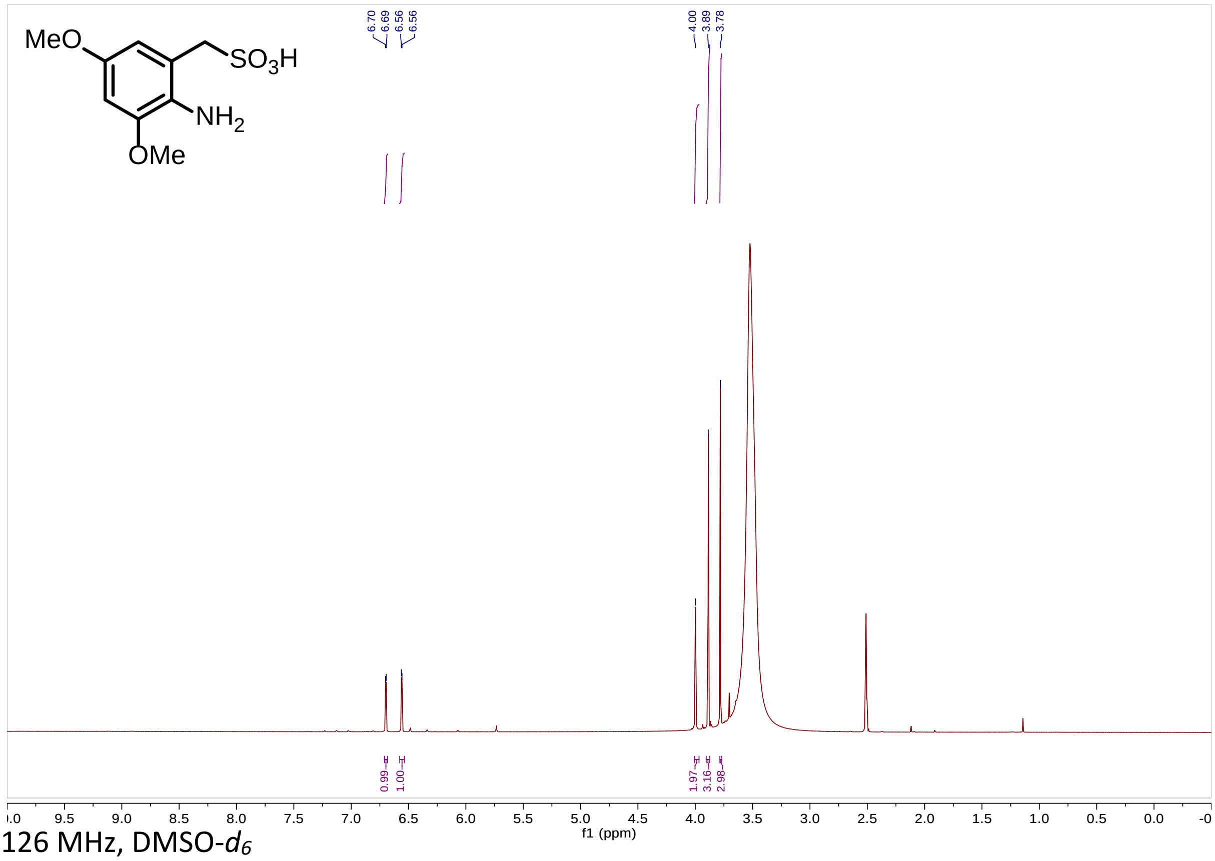

126 MHz, DMSO-d_6

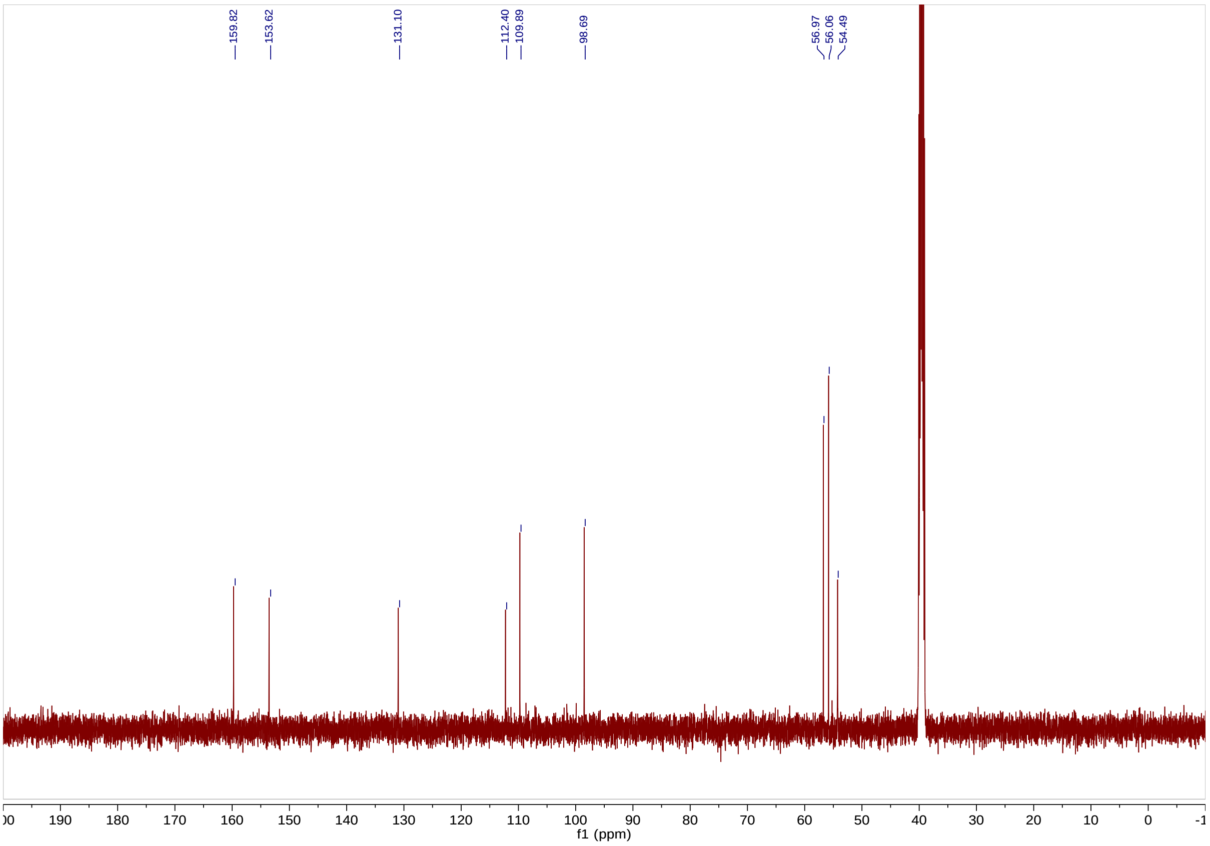

(2-Amino-4-bromophenyl)methanesulfonic acid 4f

400 MHz, DMSO-d_6

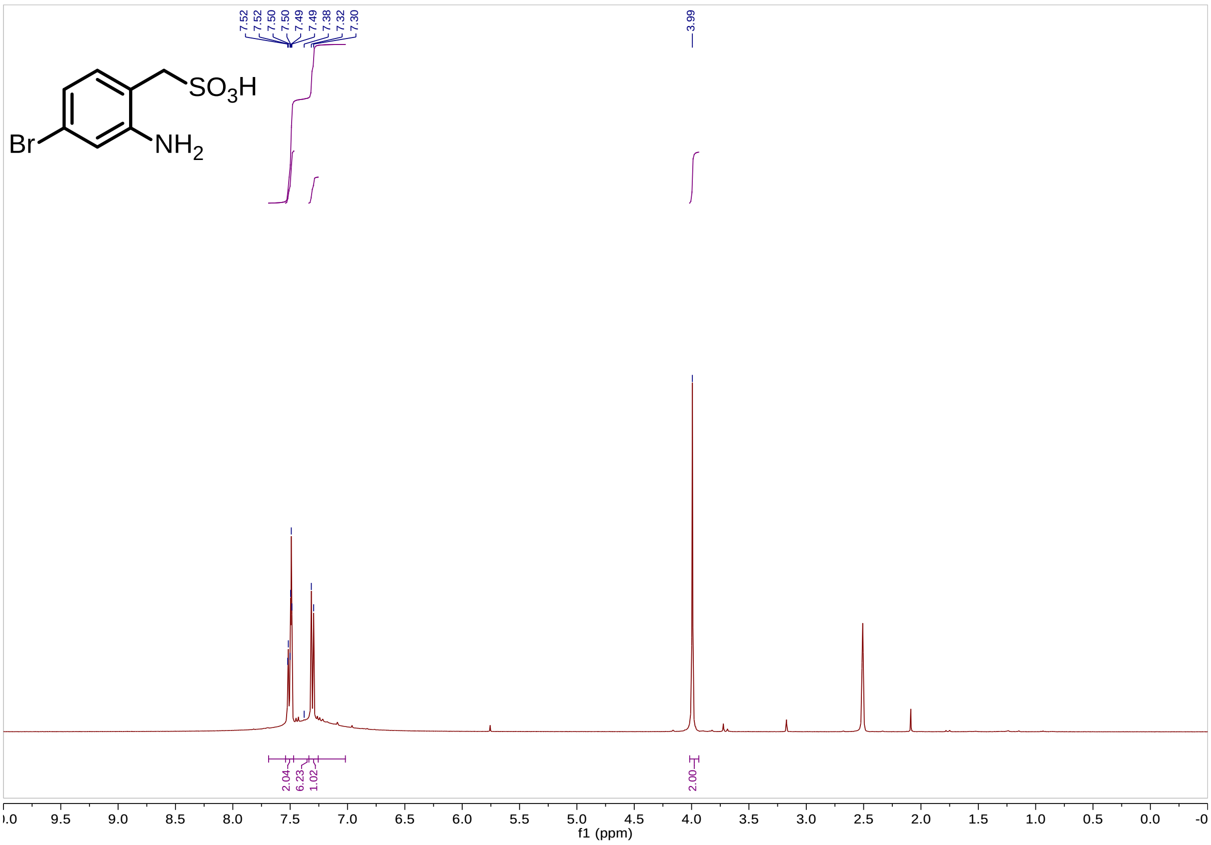

101 MHz, DMSO-d_6

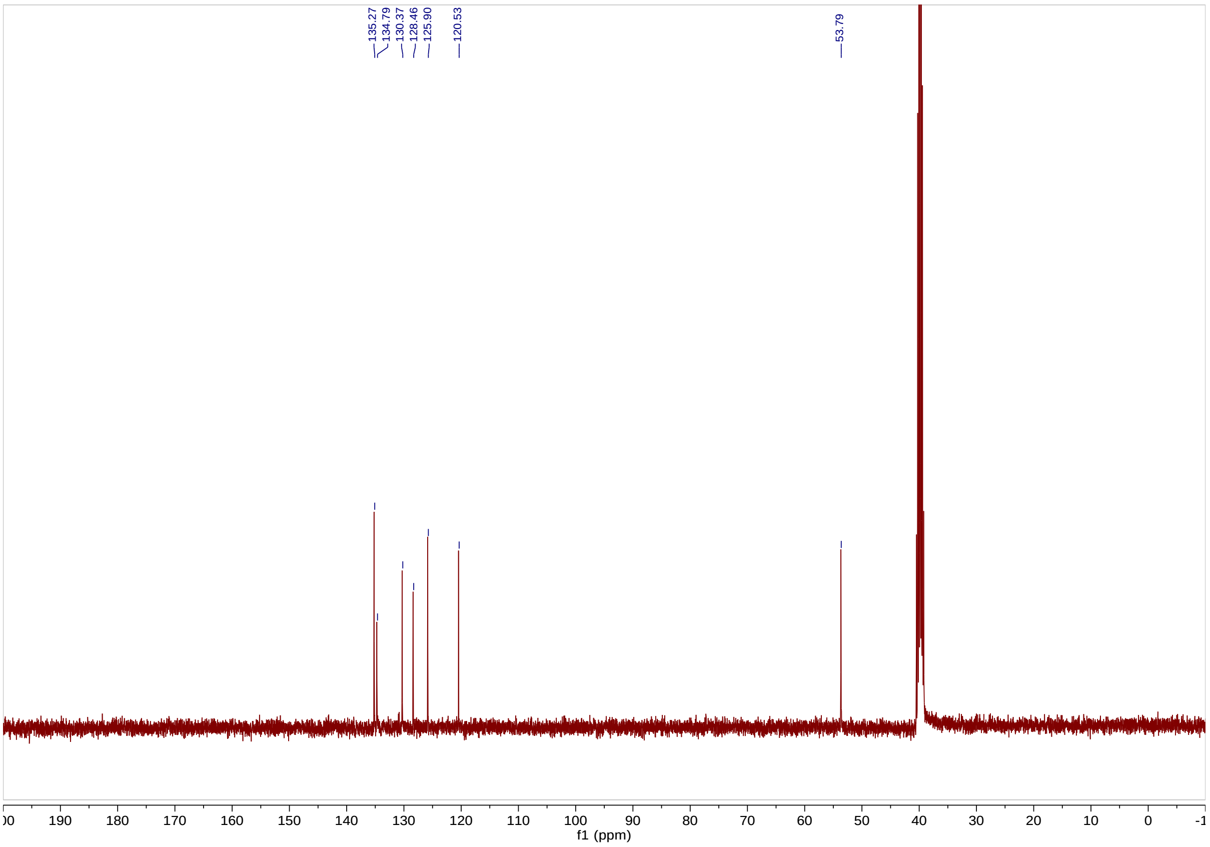

(2-Amino-5-bromophenyl)methanesulfonic acid and (2-amino-3-bromophenyl)methanesulfonic acid 4g

500 MHz, DMSO-d_6

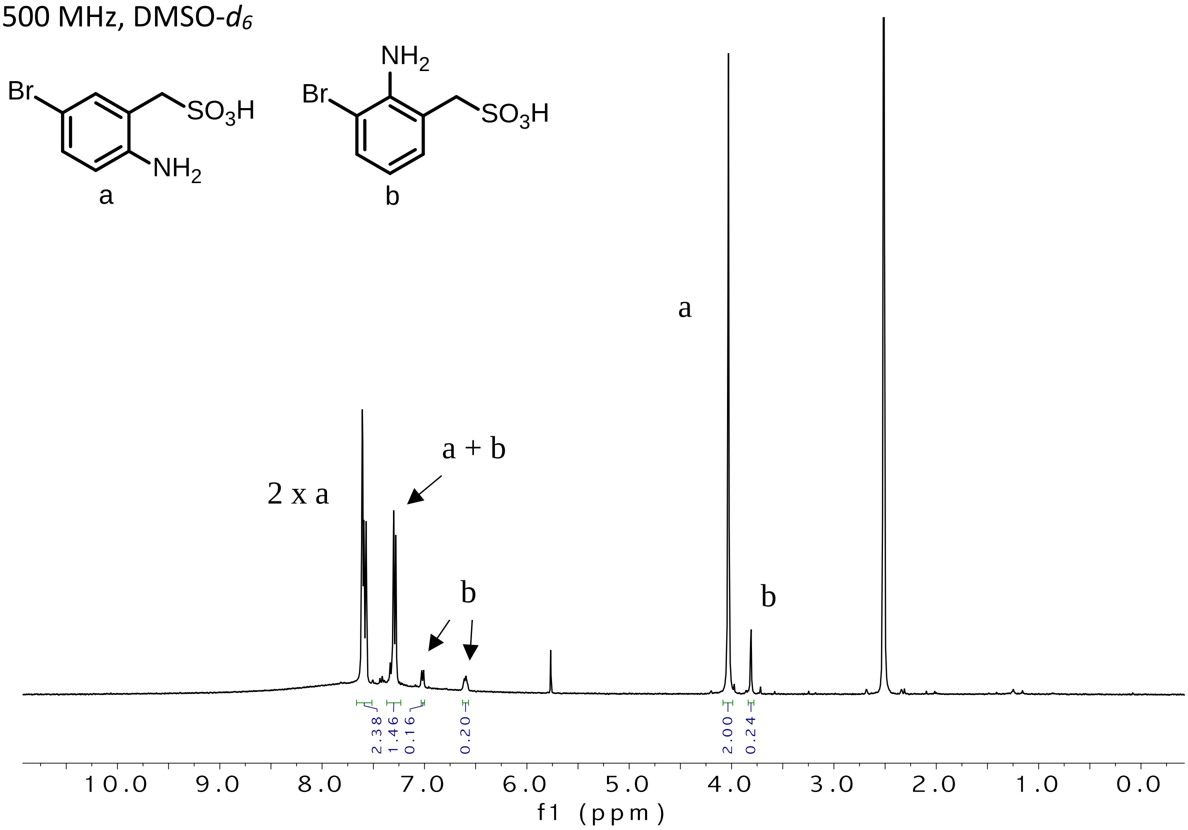

126 MHz, DMSO-d_6

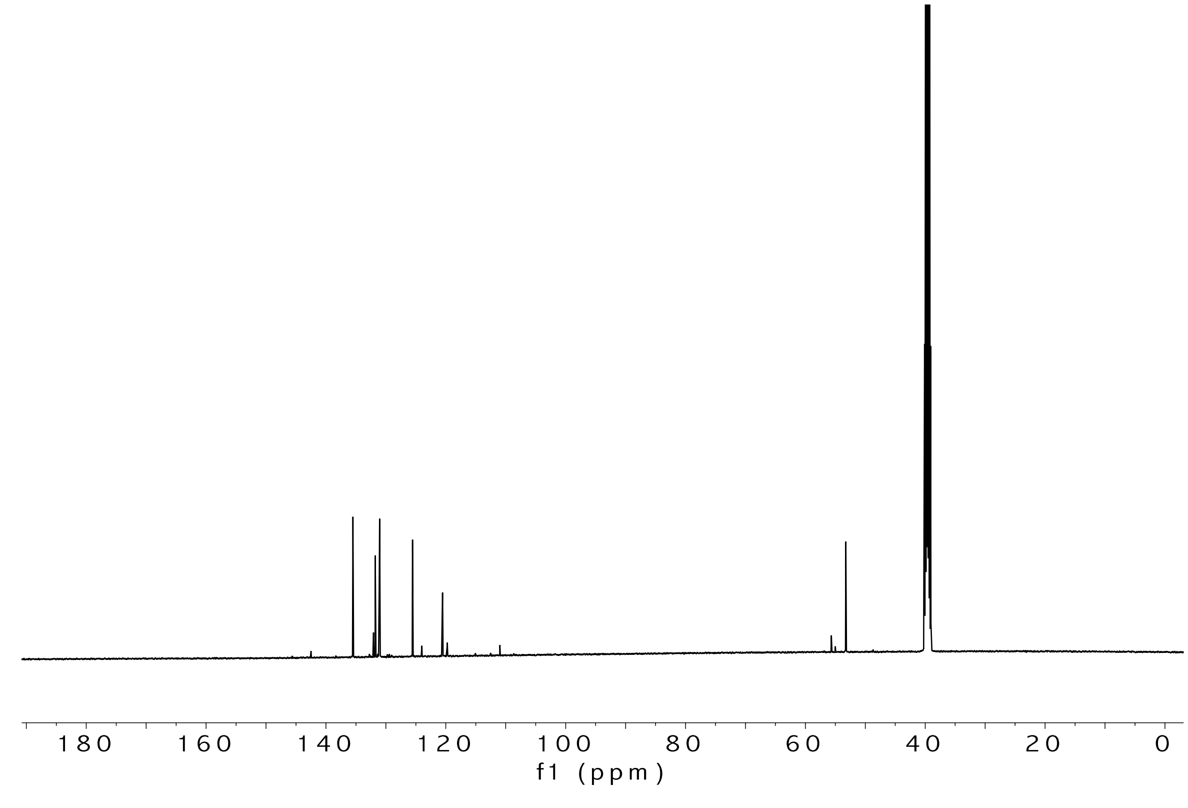

(2-Amino-4-(fluoro)phenyl)methanesulfonic acid 4h

500 MHz, DMSO-d_6

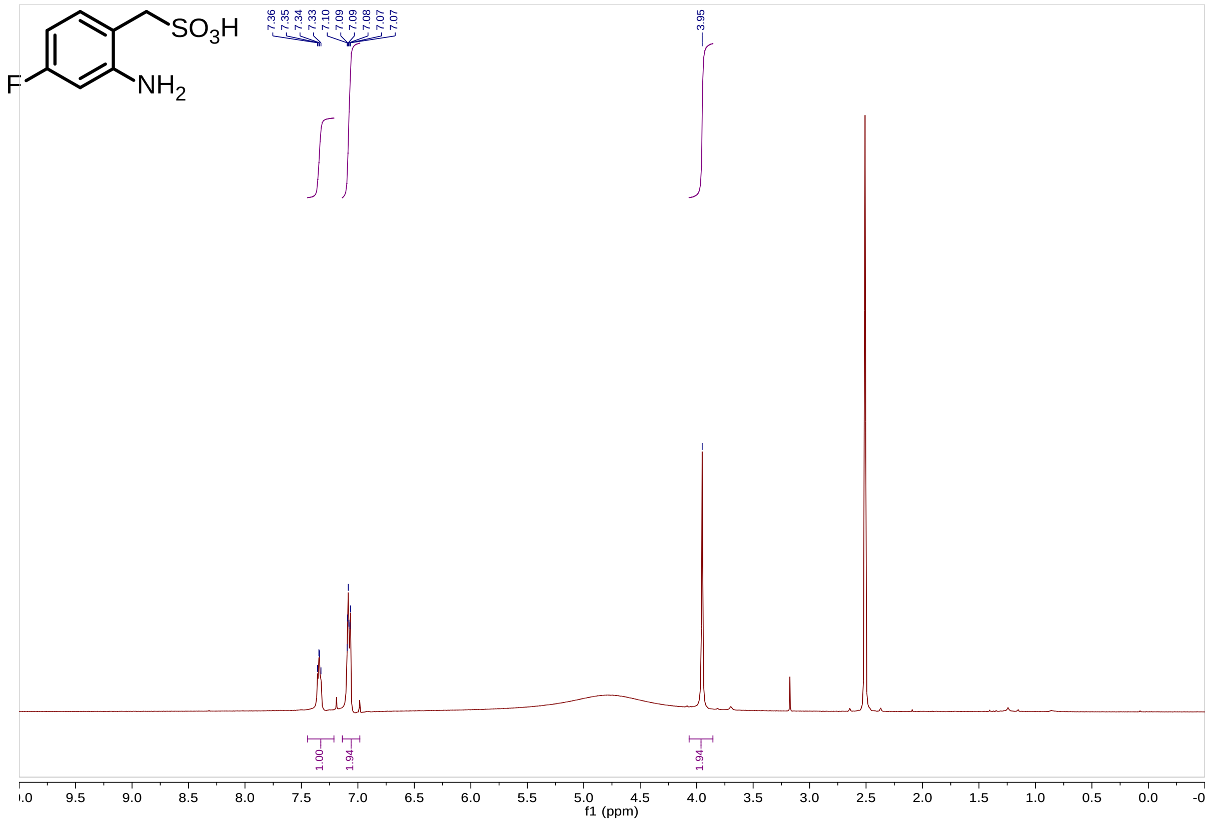

126 MHz, DMSO-d_6

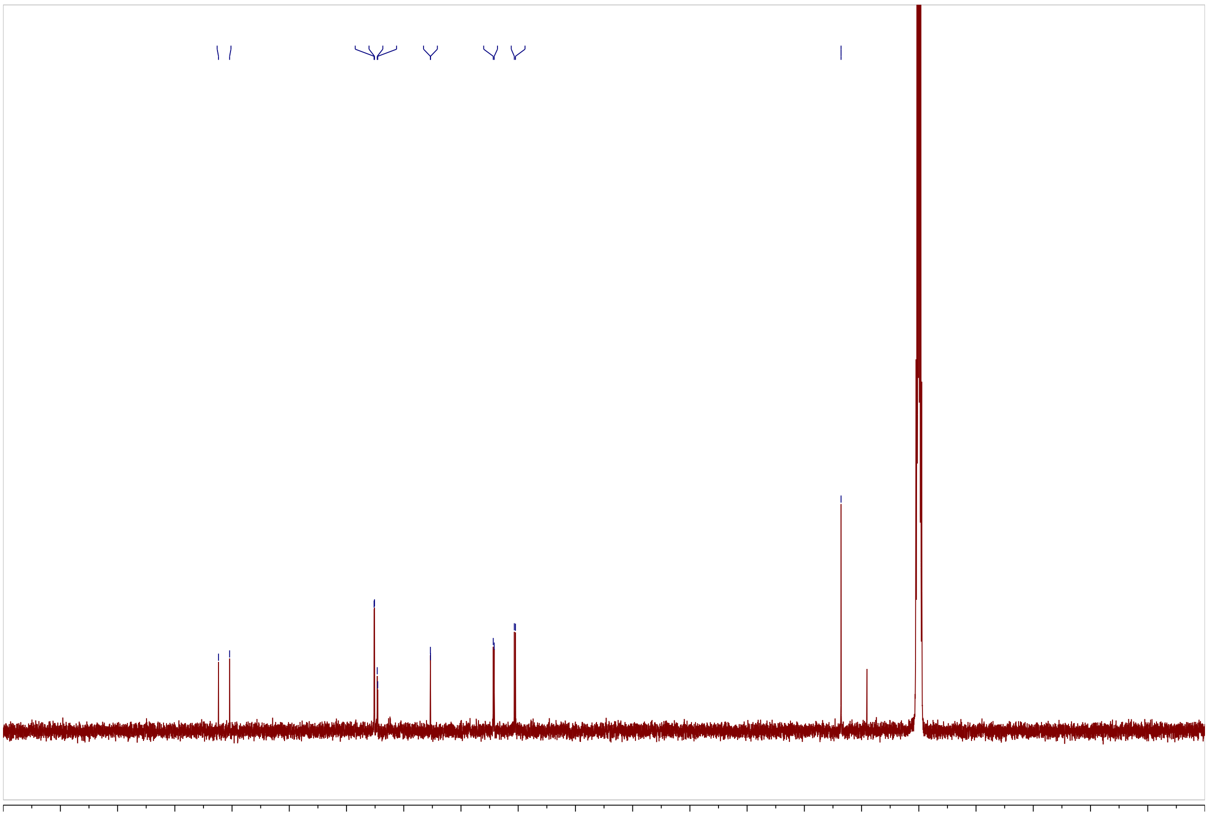

471 MHz, DMSO-d_6

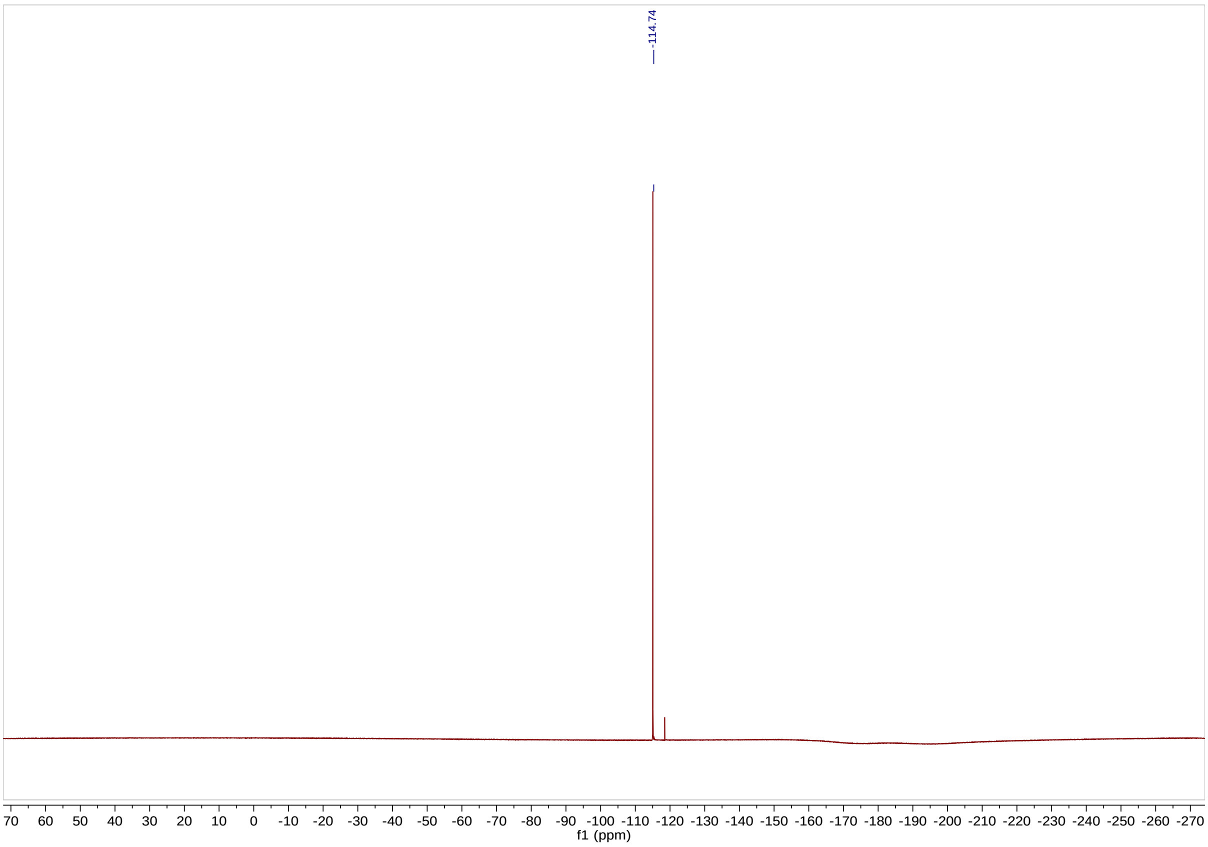

(2-Amino-4-(trifluoromethyl)phenyl)methanesulfonic acid 4i

500 MHz, DMSO-d_6

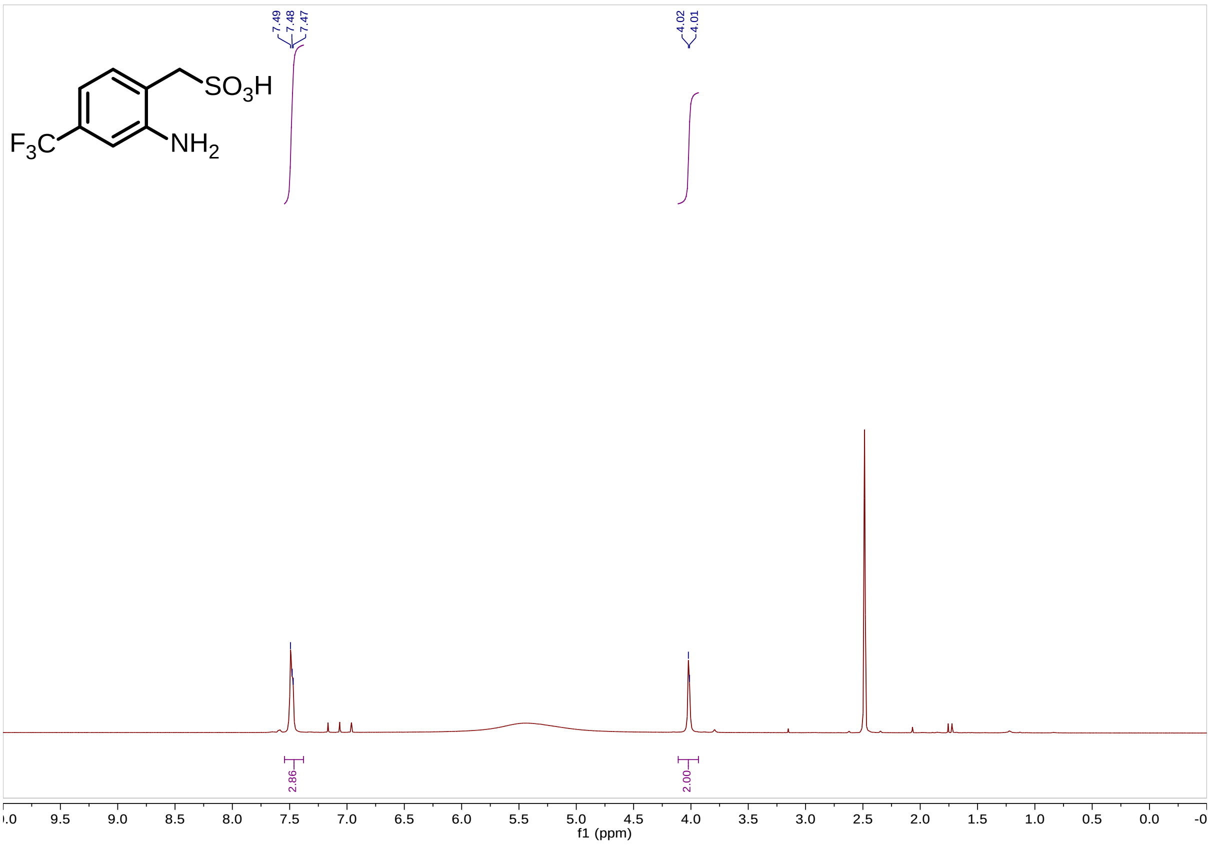

126 MHz, DMSO-d_6

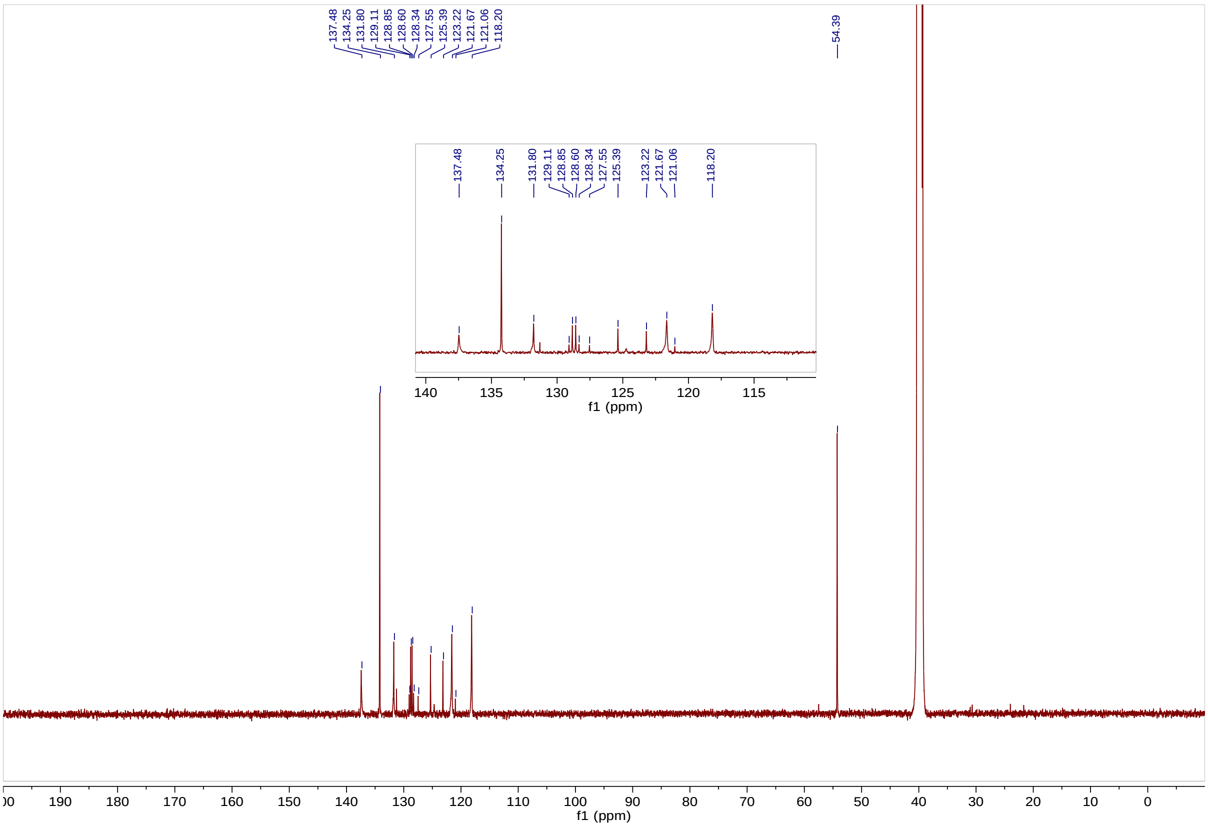

471 MHz, DMSO-d_6

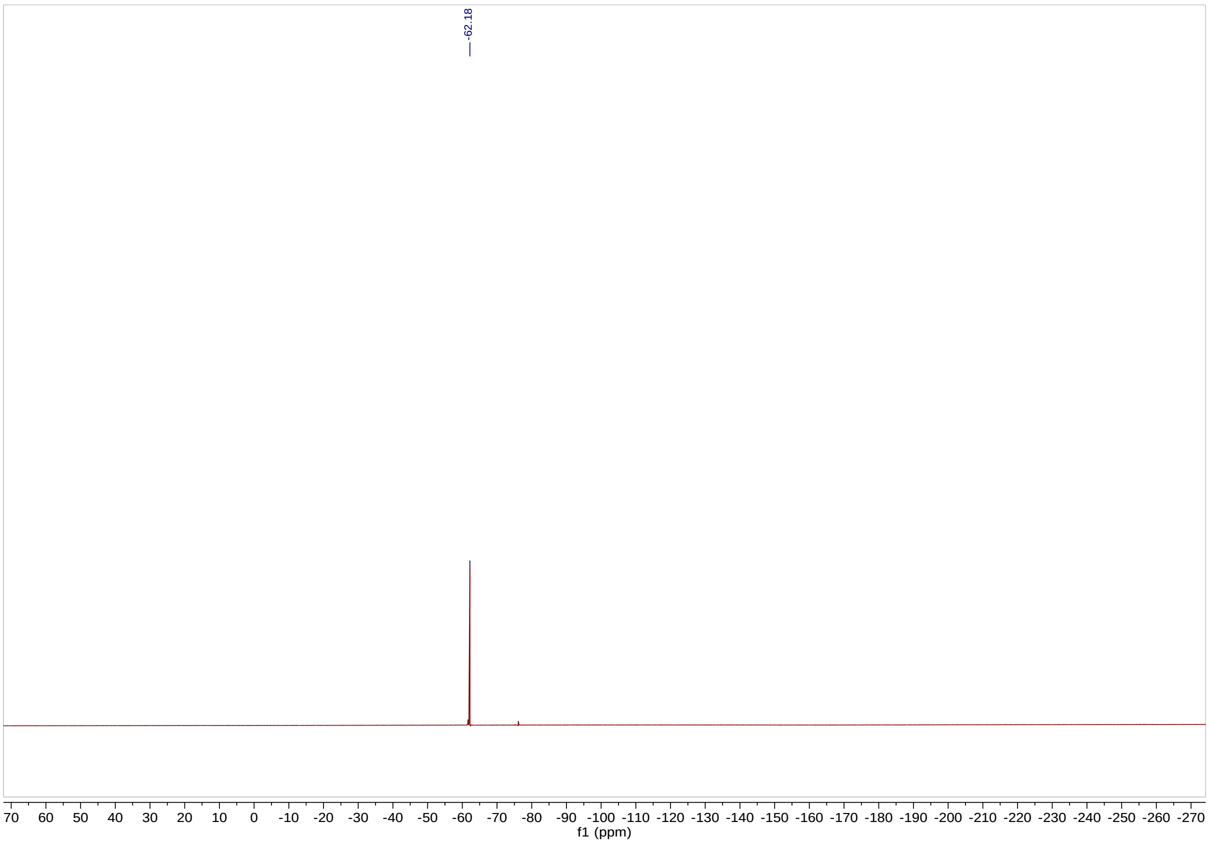

2-Aminobenzenesulfonyl chloride 8a

400 MHz, $CDCl_3$

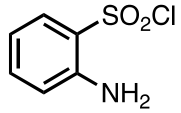

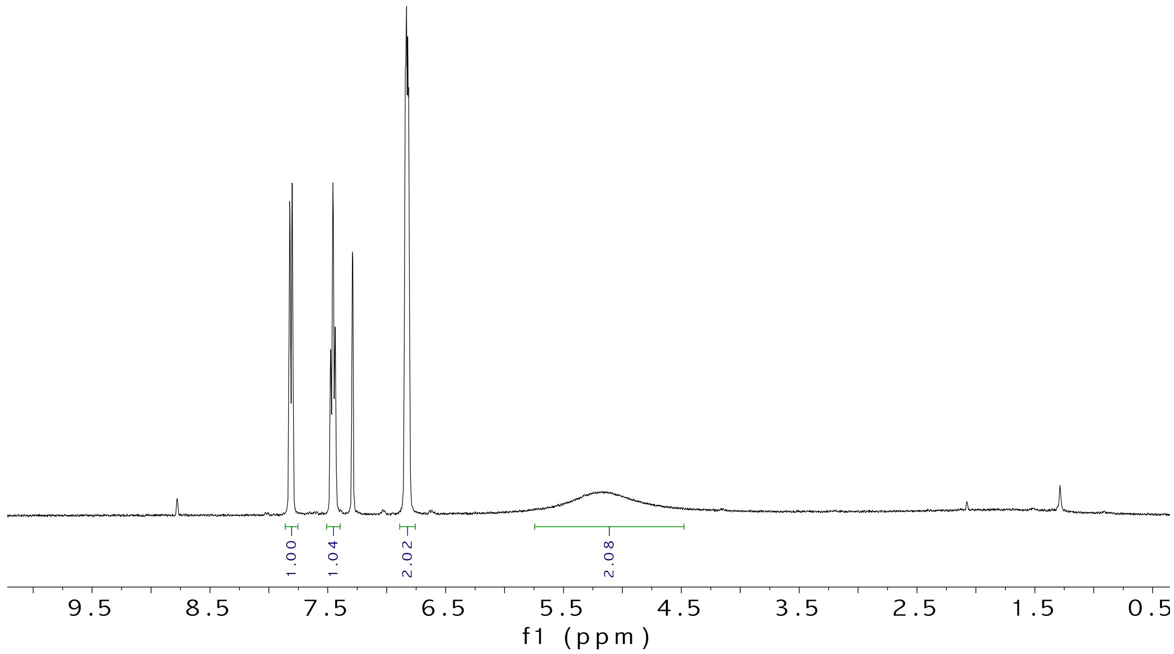

101 MHz, $CDCl_3$

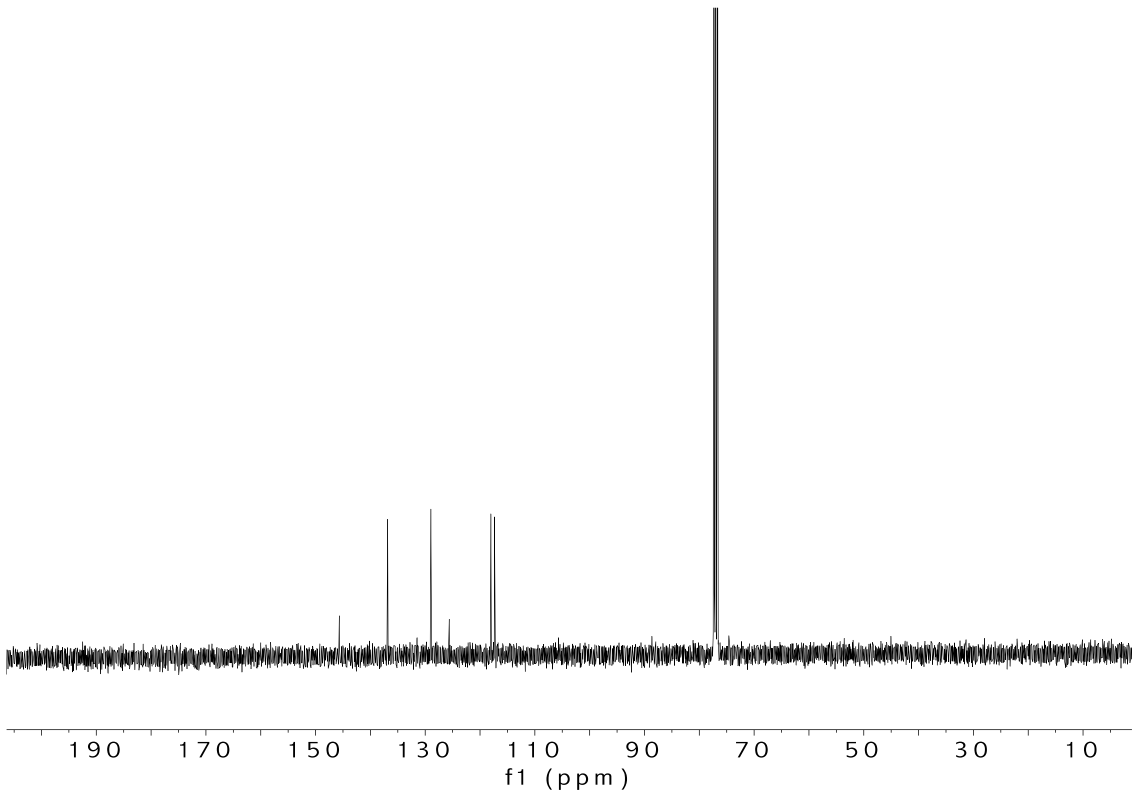

***N*-Allyl-2-aminobenzenesulfonamide 8b**

500 MHz, $CDCl_3$

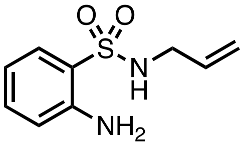

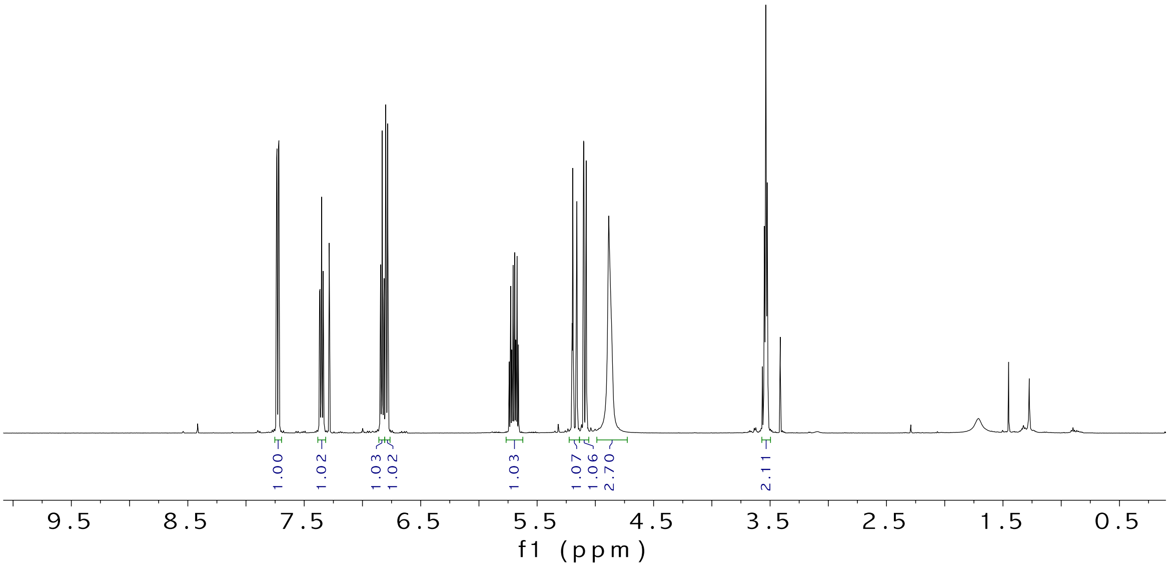

126 MHz, $CDCl_3$

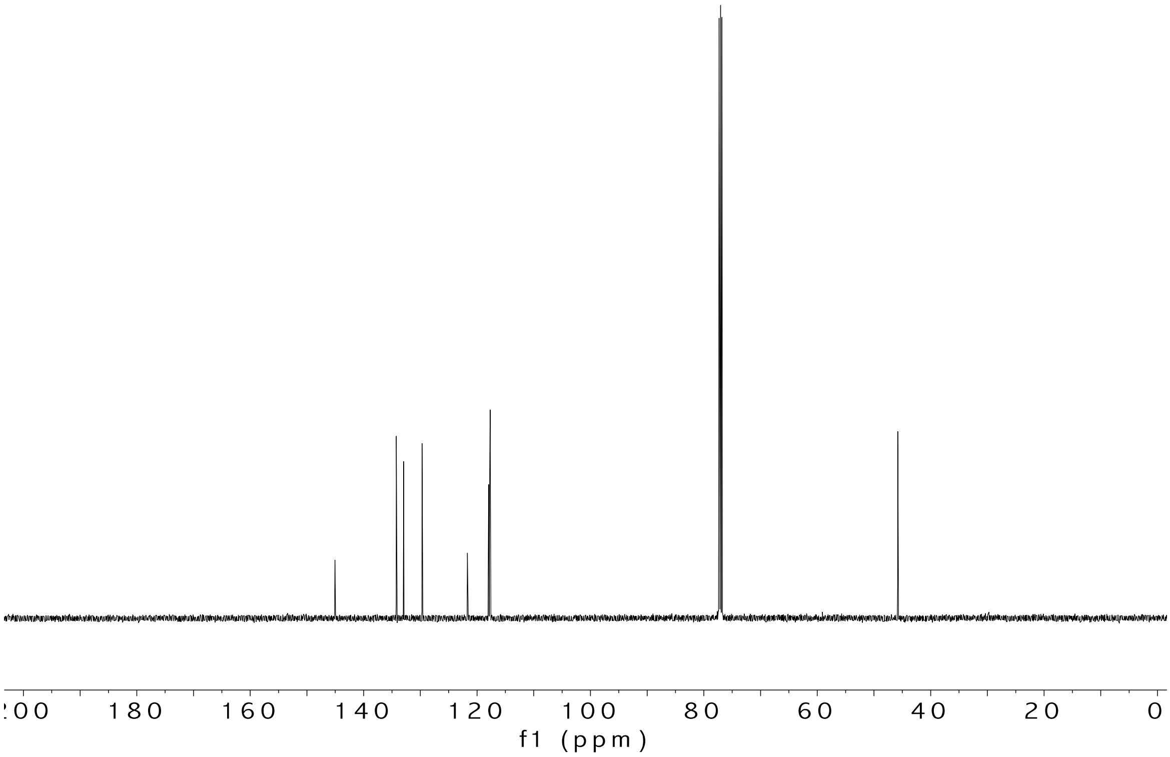

2-(Methylsulfonyl)aniline 8c

400 MHz, $CDCl_3$

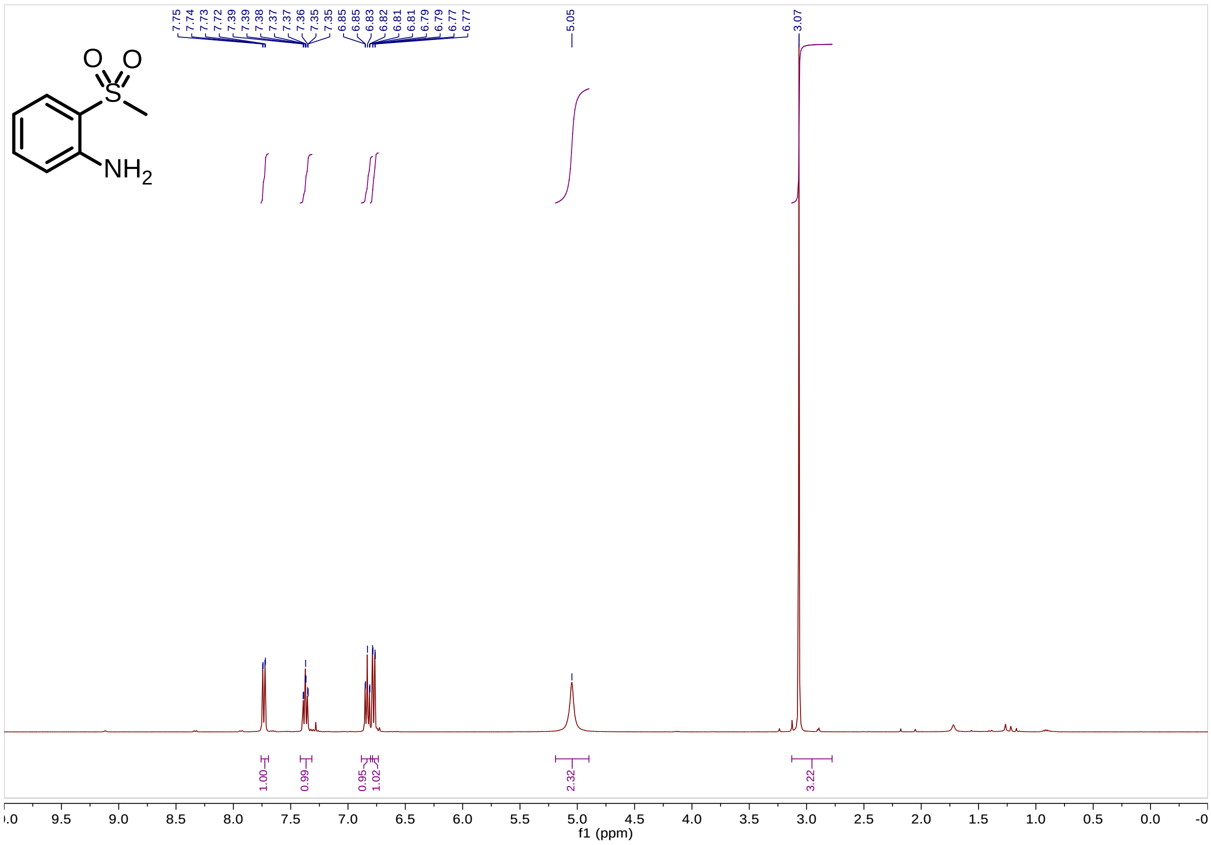

101 MHz, $CDCl_3$

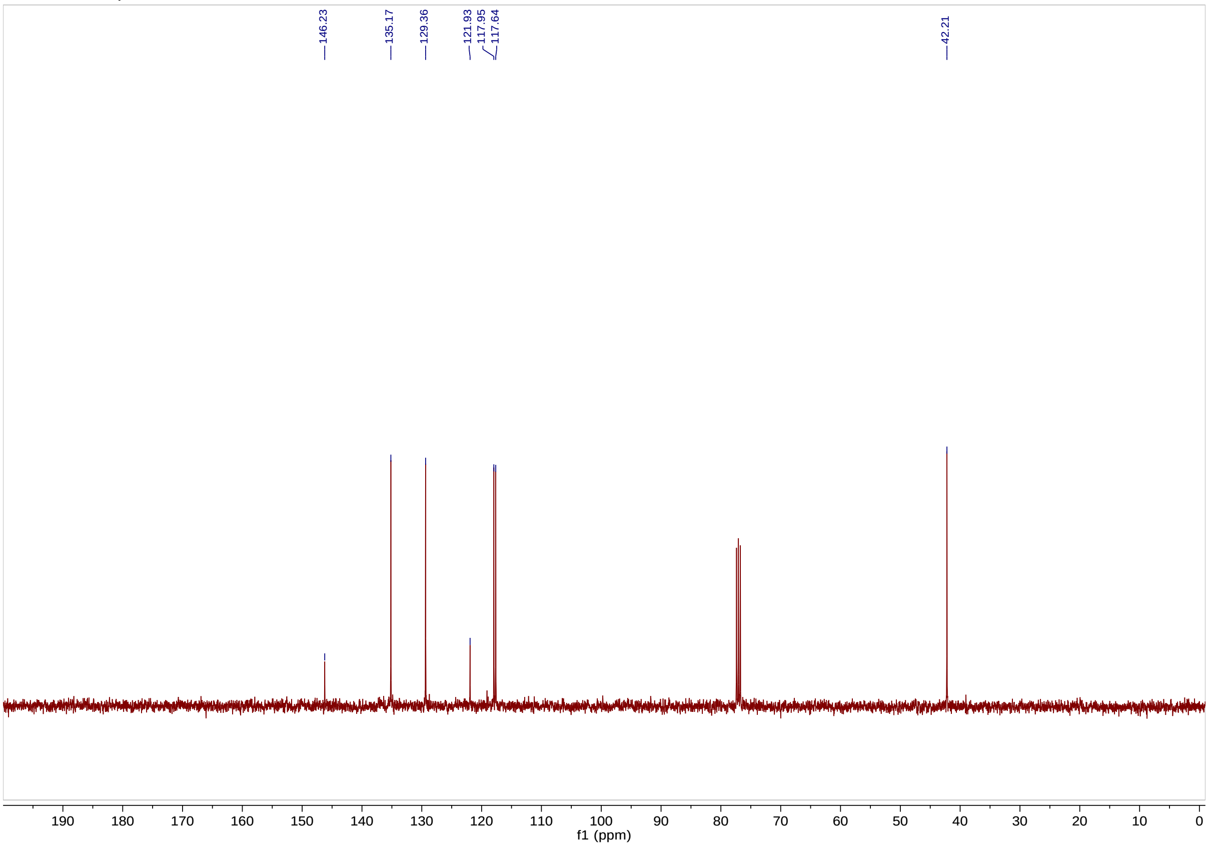

2-Iodobenzenesulfonic acid 8d

400 MHz, D_2O

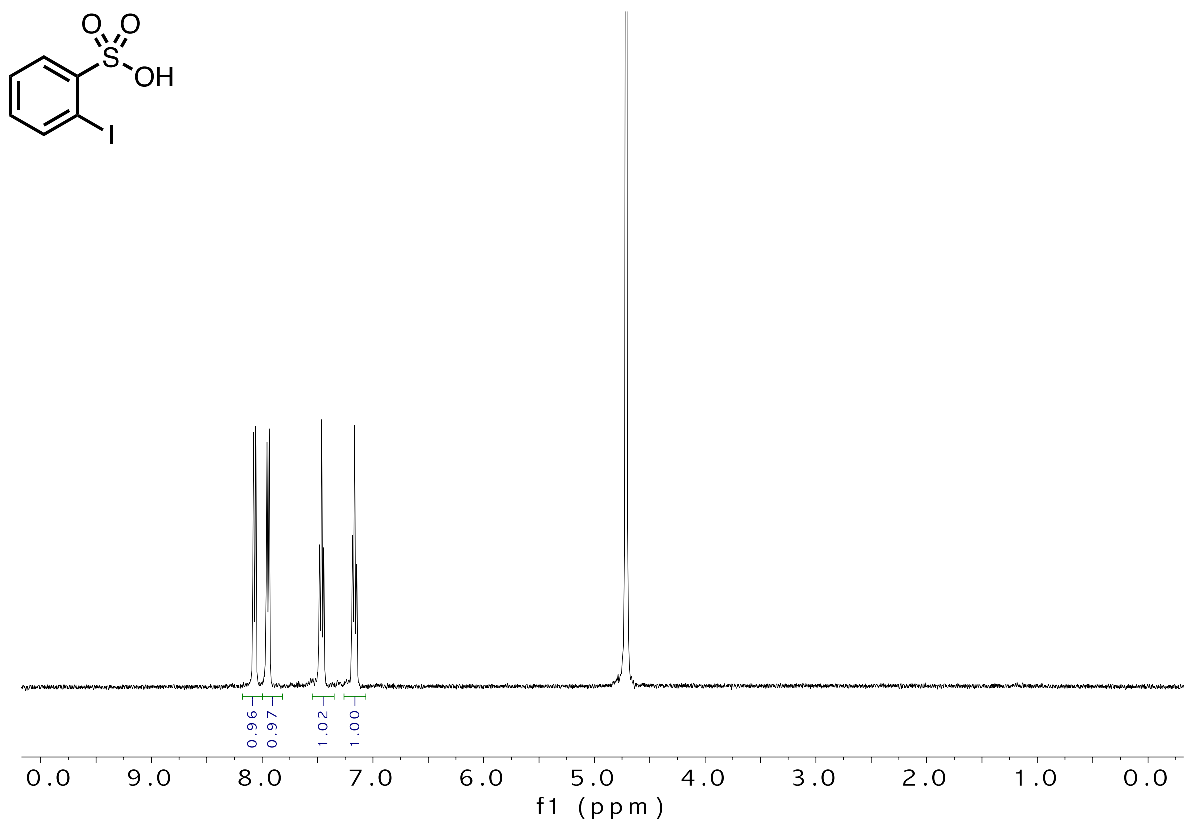

101 MHz, D_2O

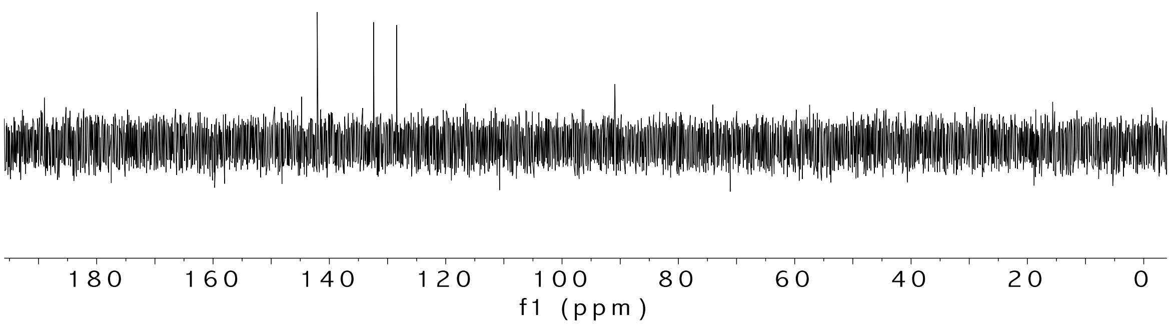

2-Acetamidobenzenesulfonic acid 8e

500 MHz, DMSO-d_6

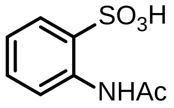

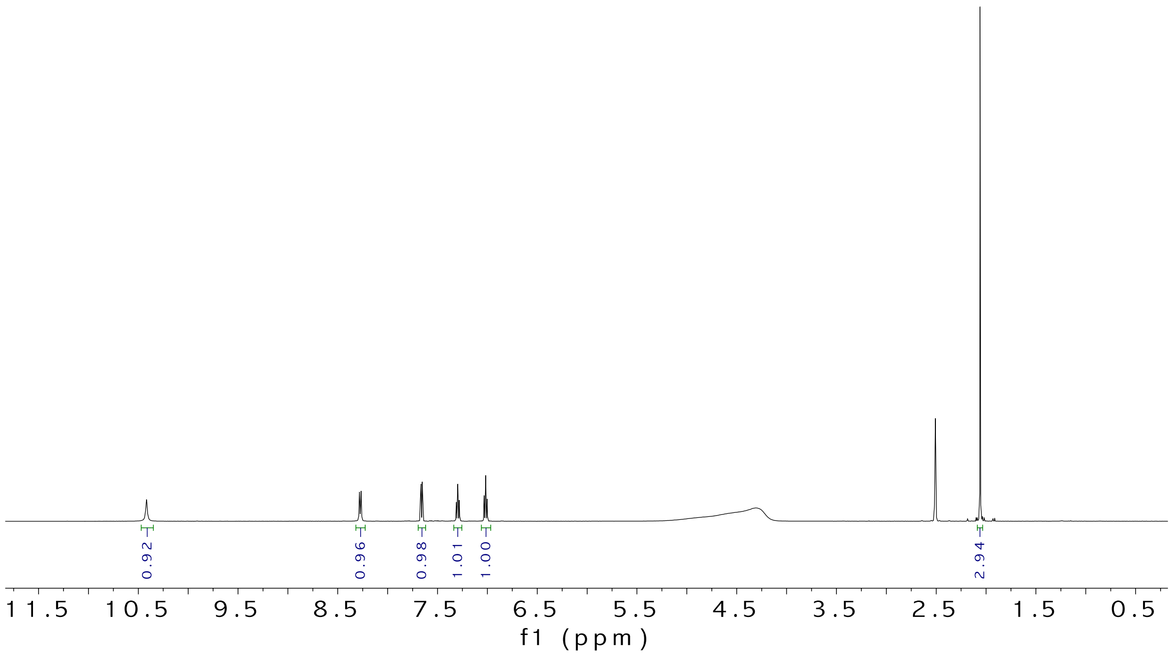

126 MHz, DMSO-d_6

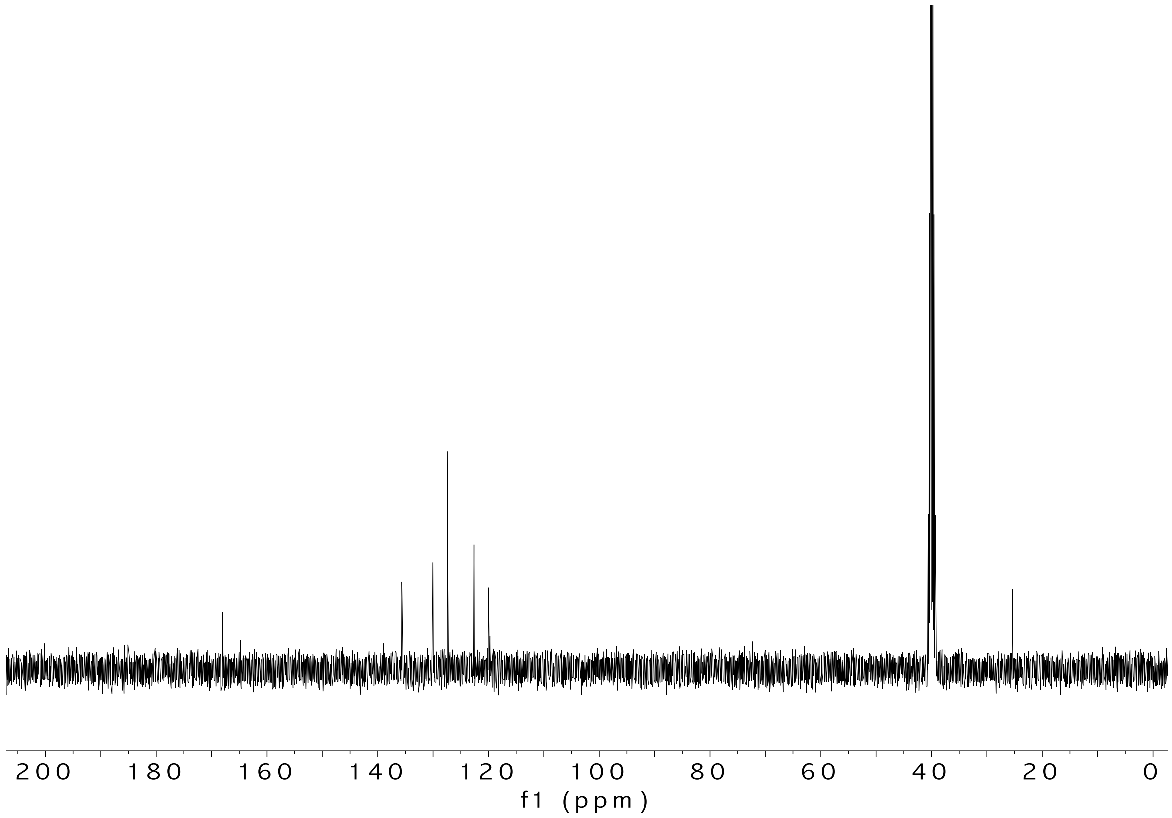

Sodium 2-aminobenzenesulfinate 8f

400 MHz, D_2O

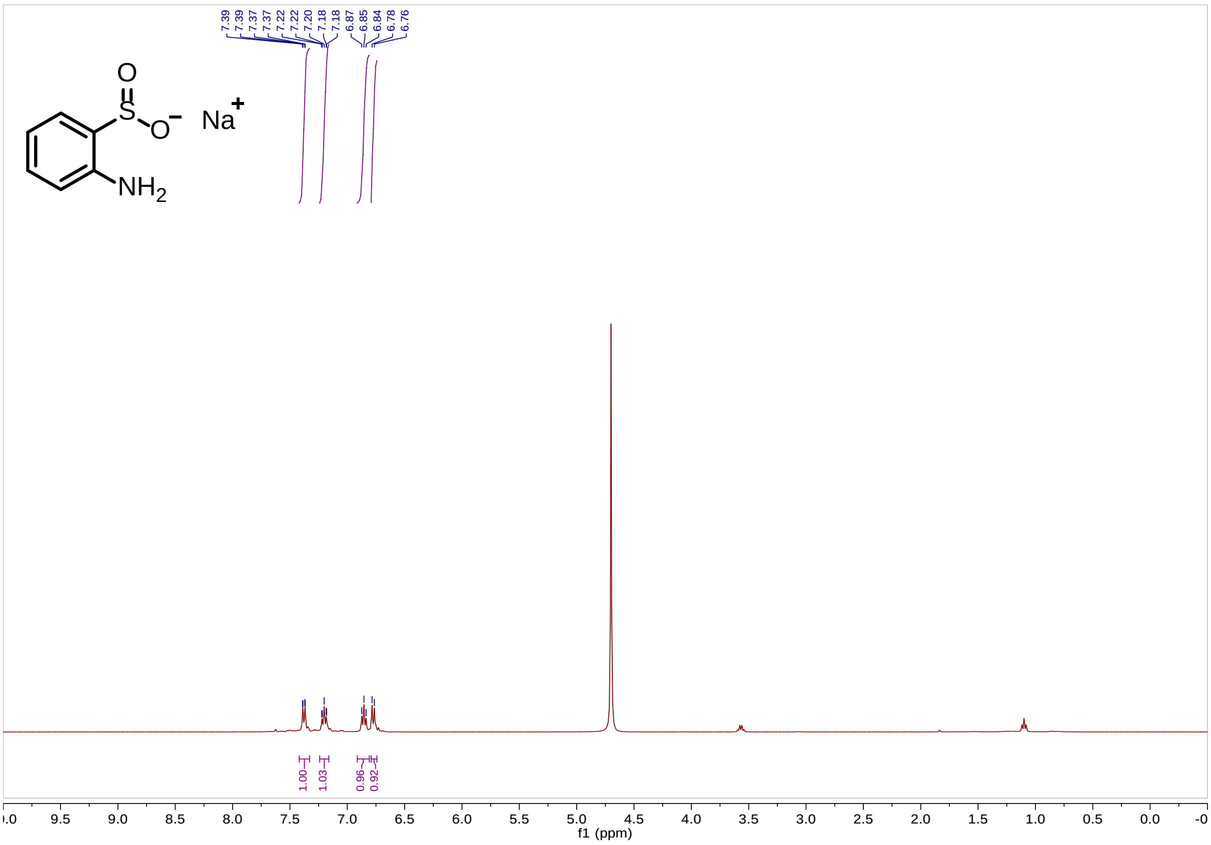

101 MHz, D_2O

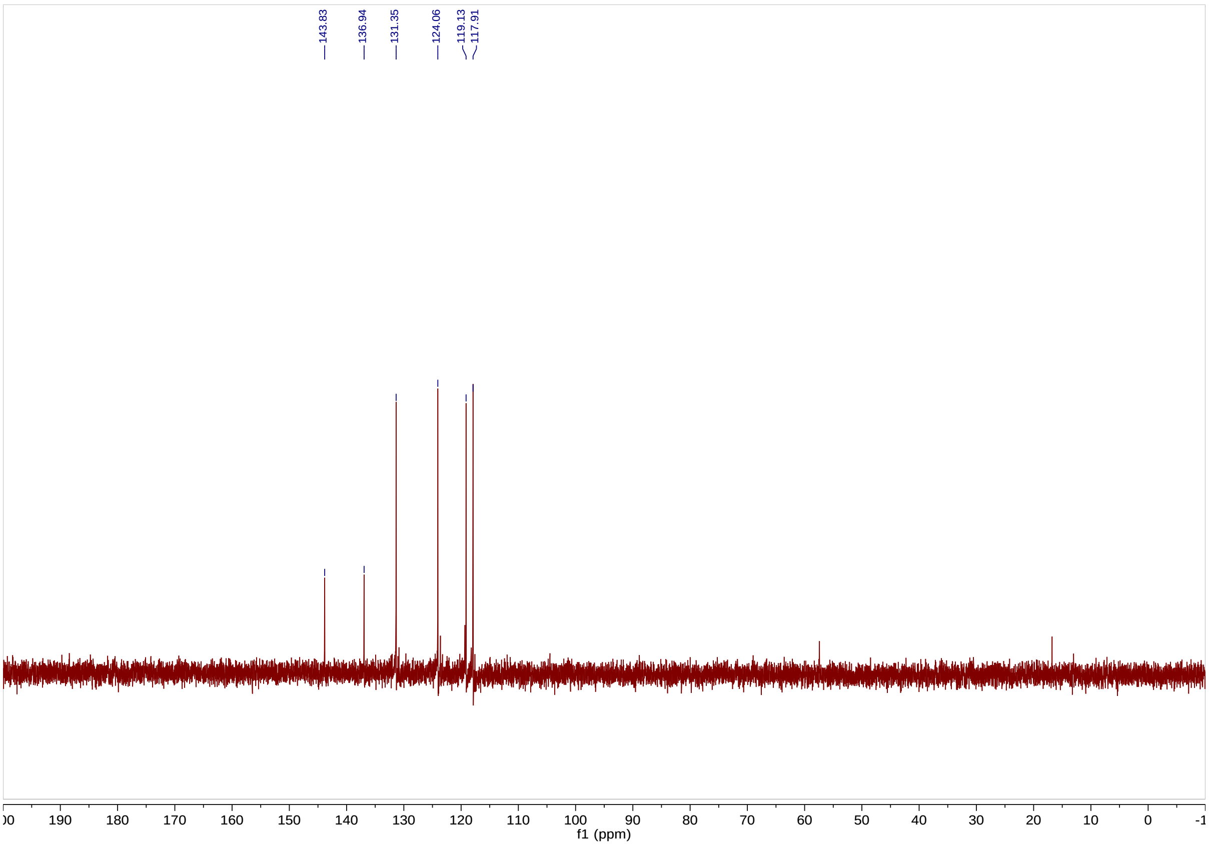

2-(Phenylsulfonyl)aniline 8g

400 MHz, $CDCl_3$

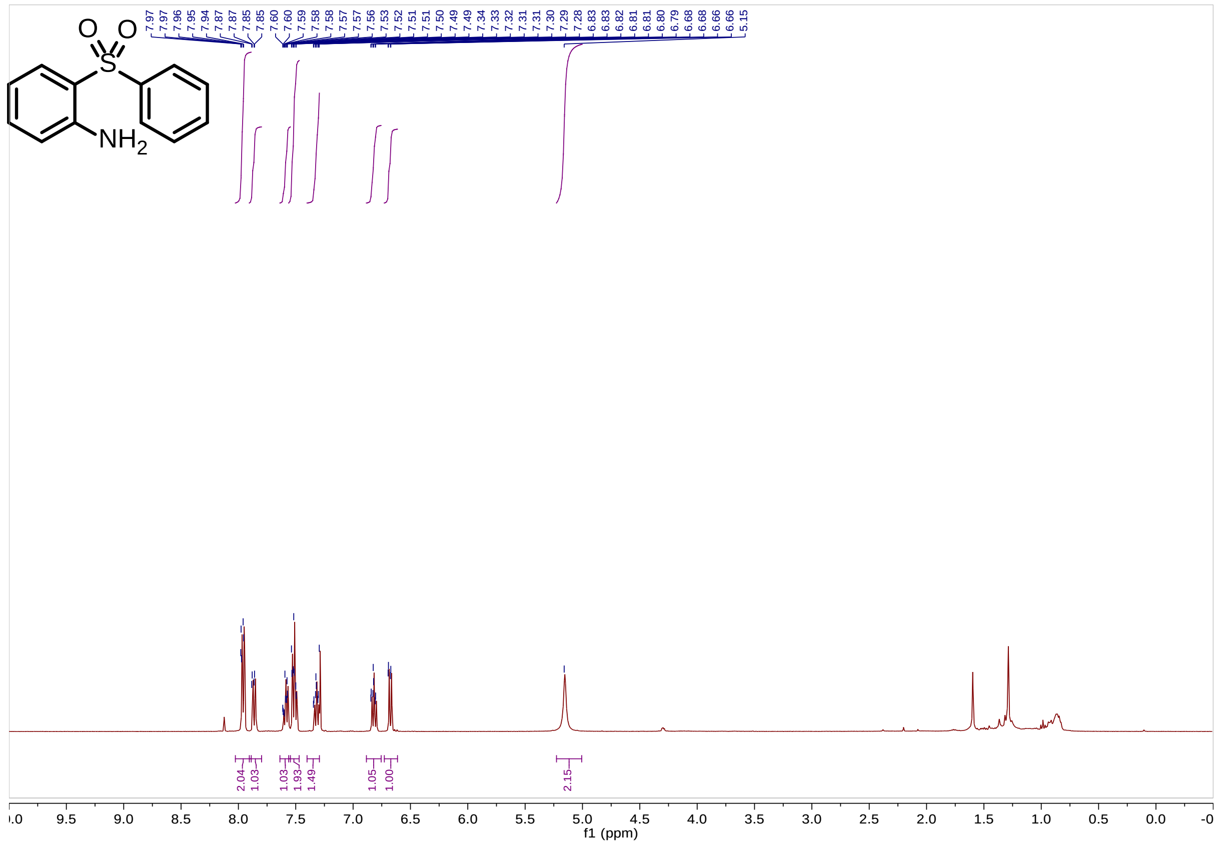

126 MHz, $CDCl_3$

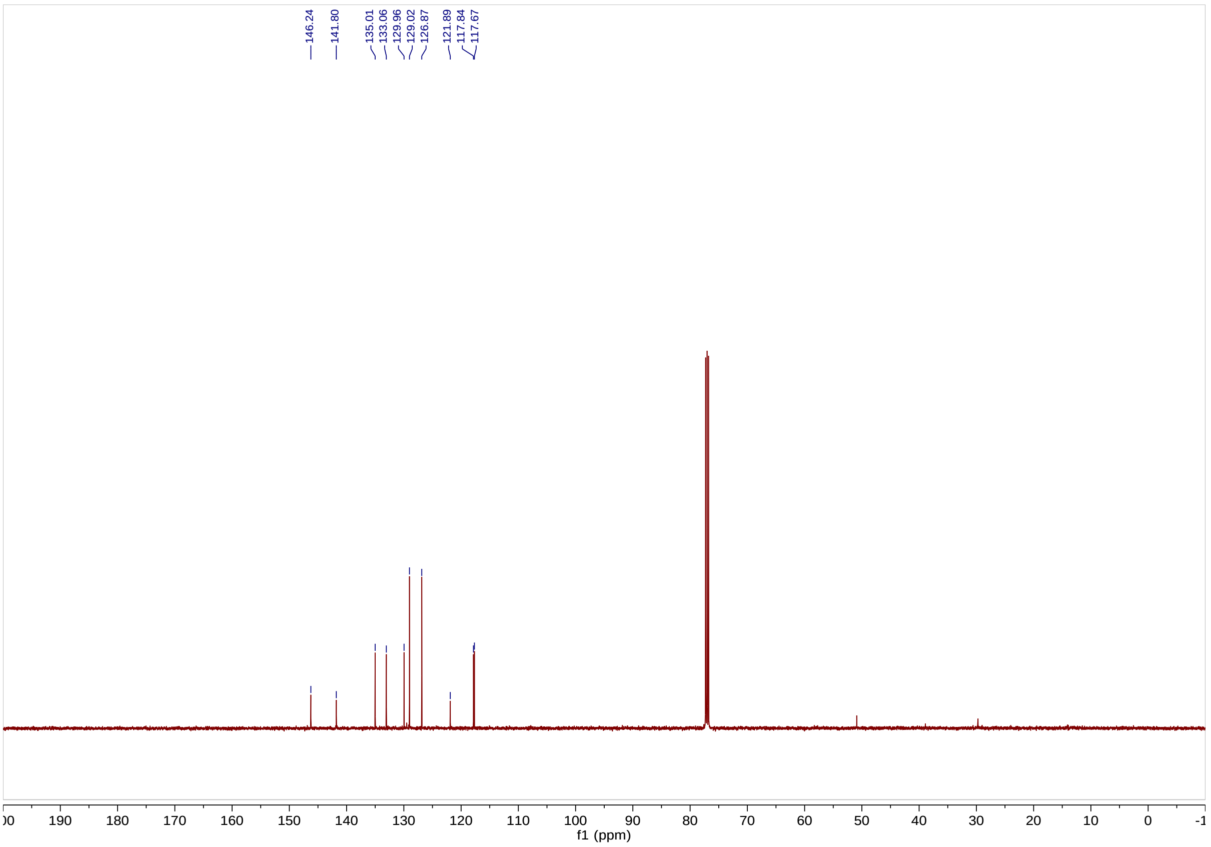

6-Bromo-1,3-dihydrobenzo[c]isothiazole-2,2-dioxide 9

400 MHz, $CDCl_3$

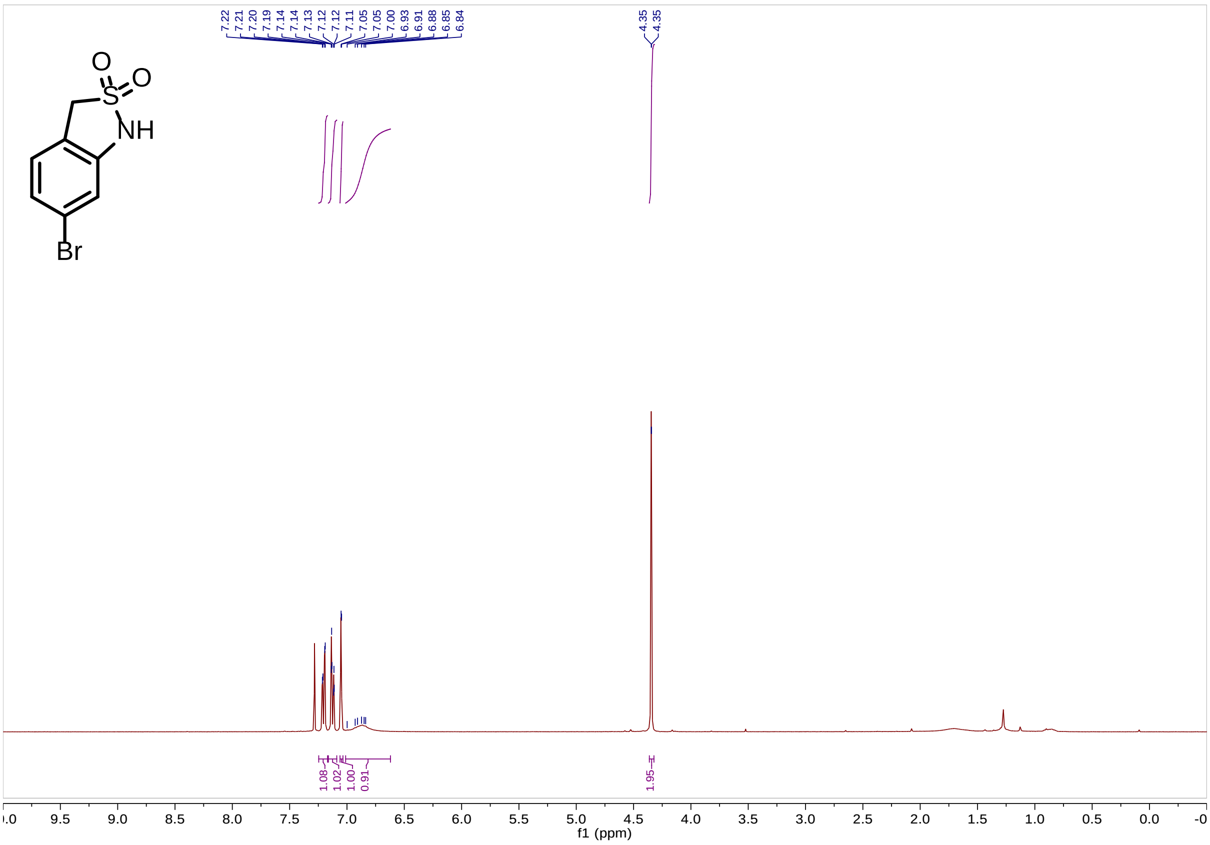

101 MHz, $CDCl_3$

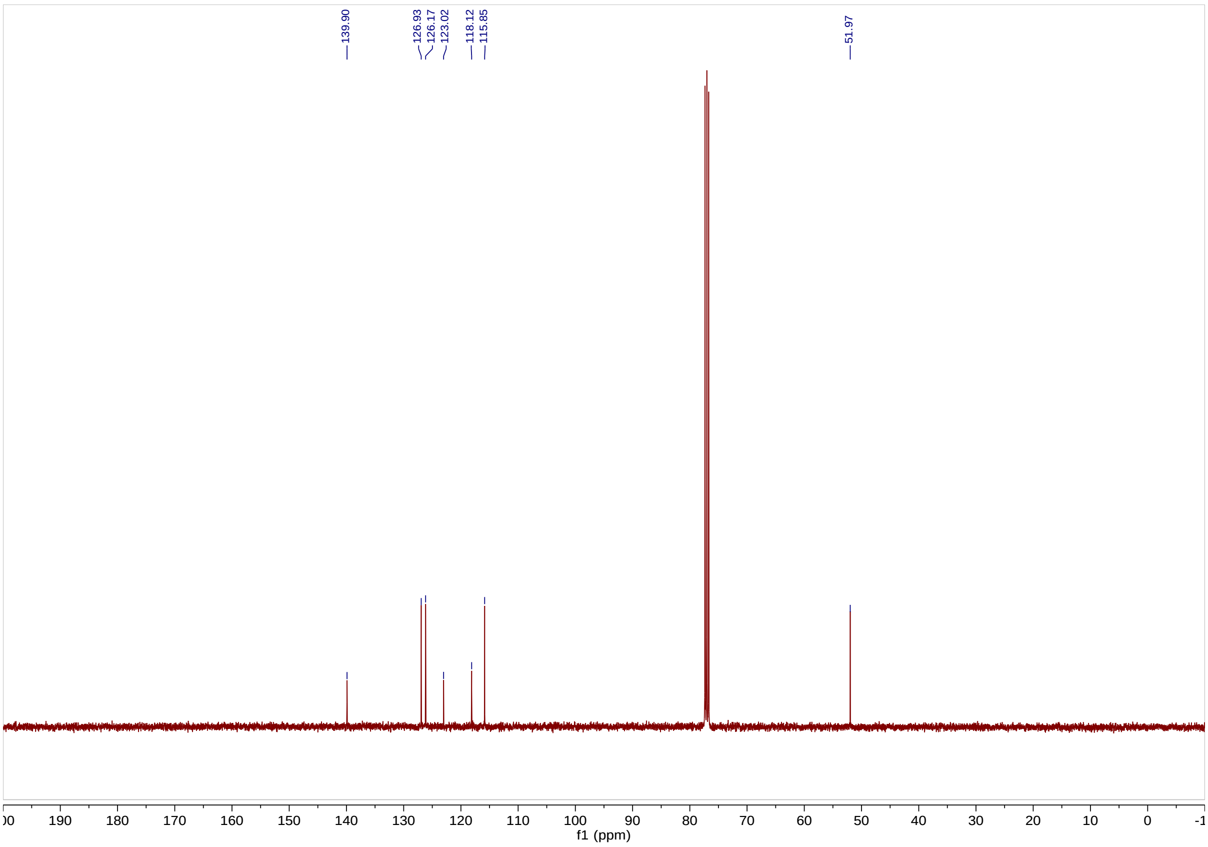

Triethylammonium 2-(methylamino)benzenesulfonate 5aa

500 MHz, $CDCl_3$

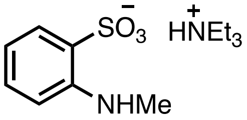

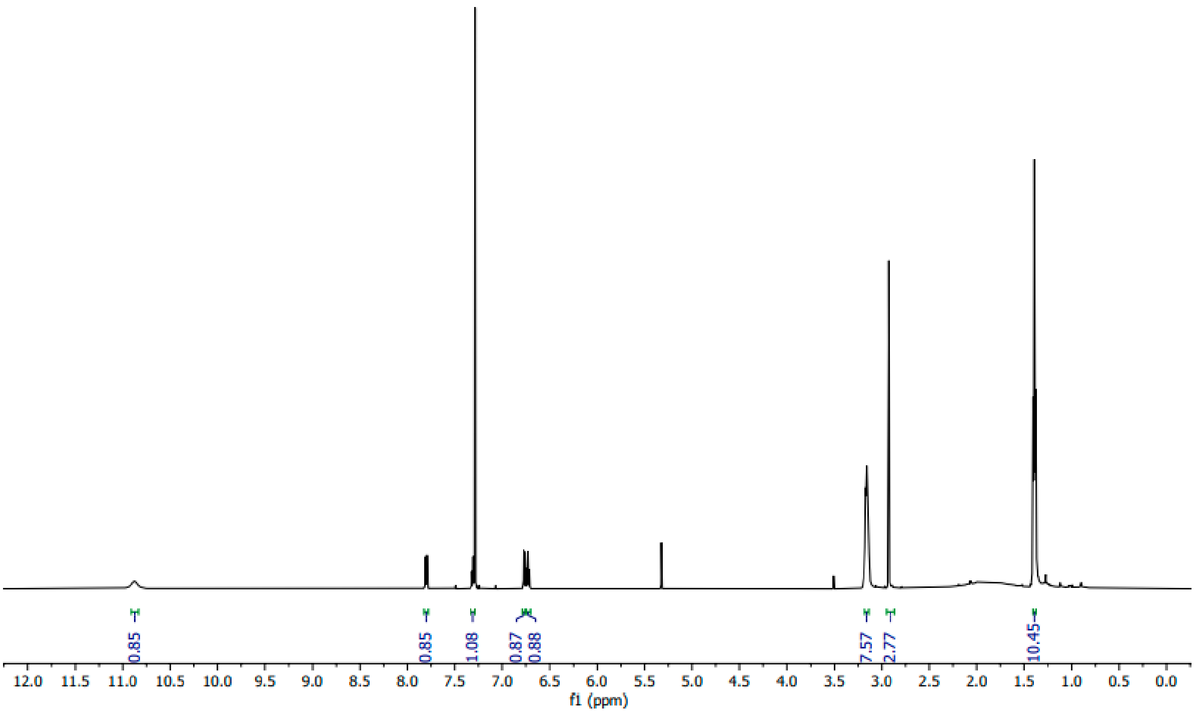

126 MHz, $CDCl_3$

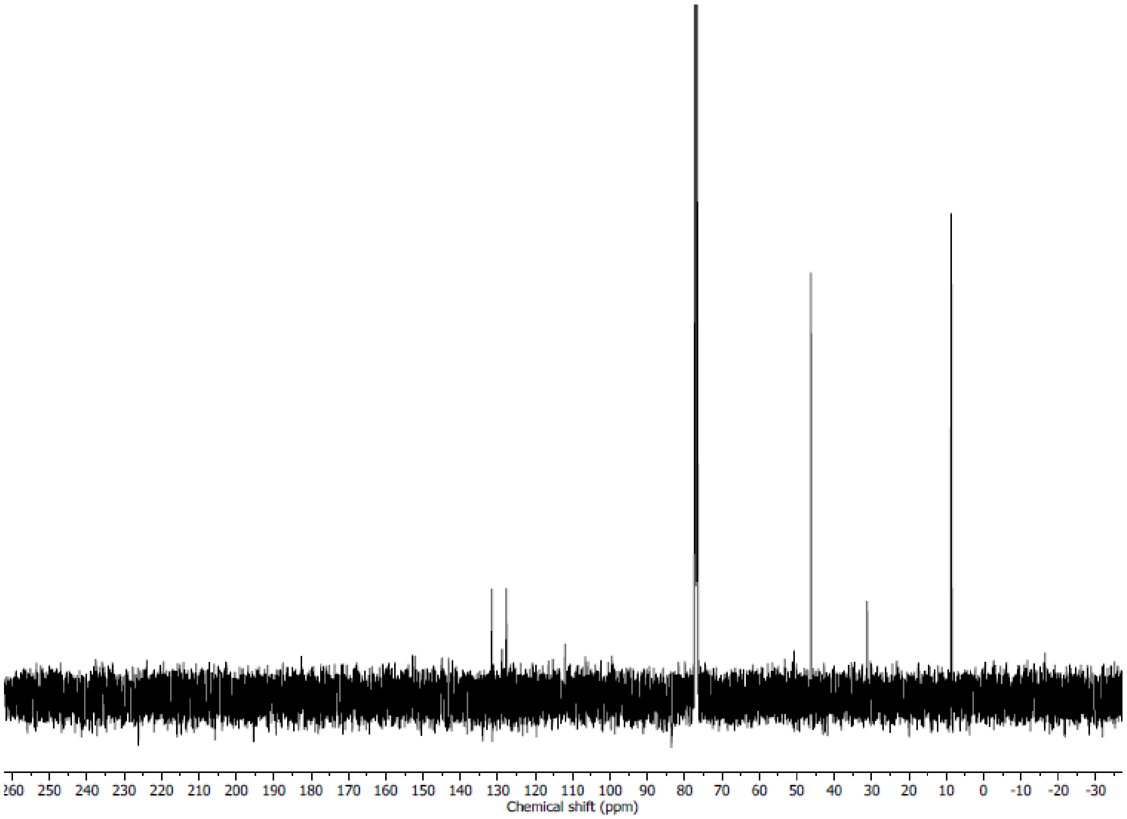

Triethylammonium 4-methyl-2-(methylamino)benzenesulfonate 1ba

500 MHz, MeOD-d_4

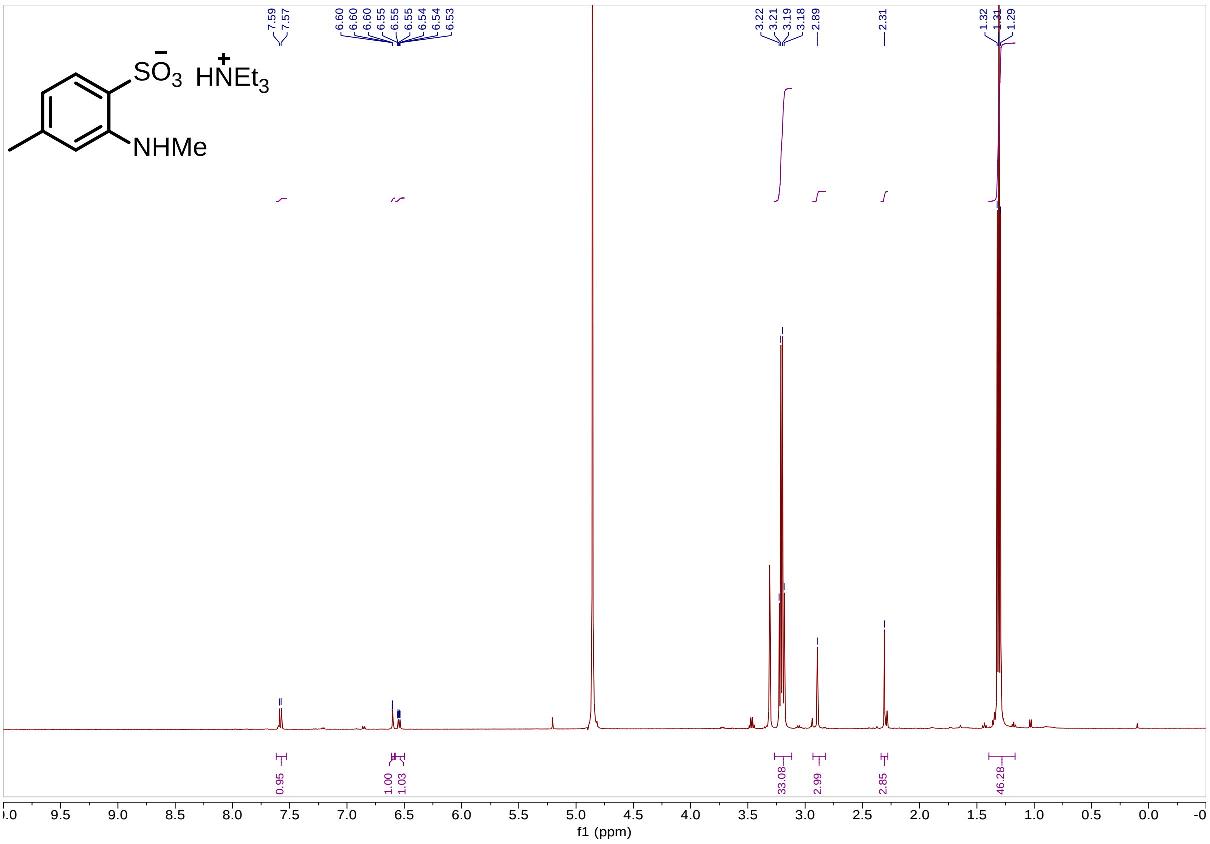

126 MHz, MeOD-d_4

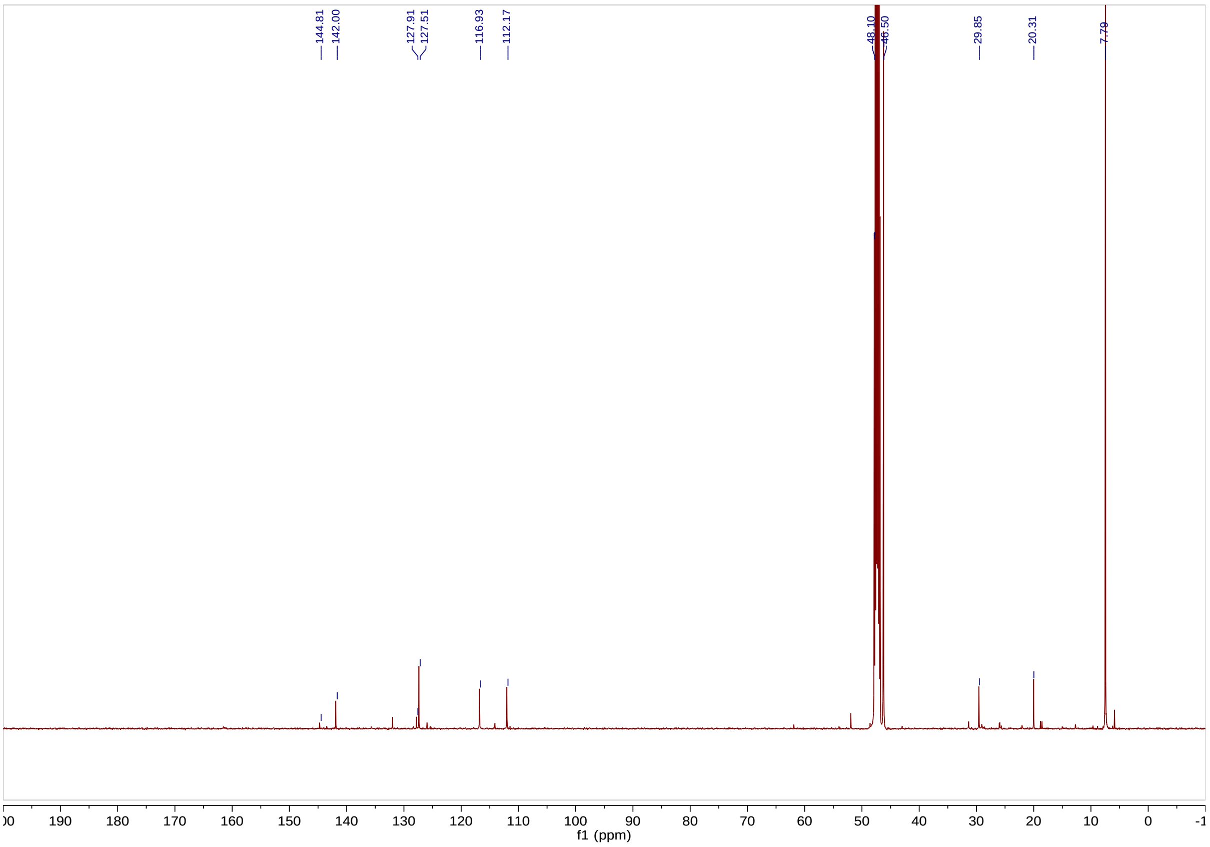

Tetrabutylammonium benzenesulfonate 6

400 MHz, $CDCl_3$

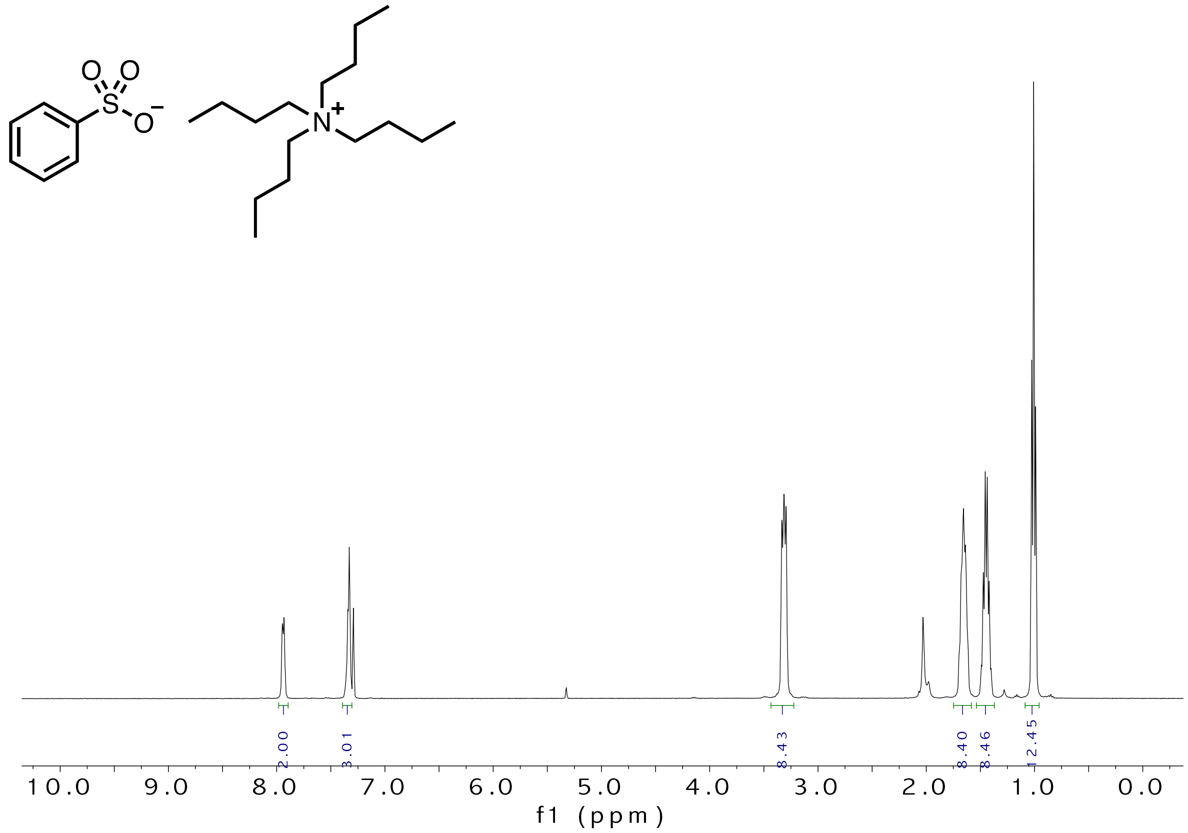

101 MHz, $CDCl_3$

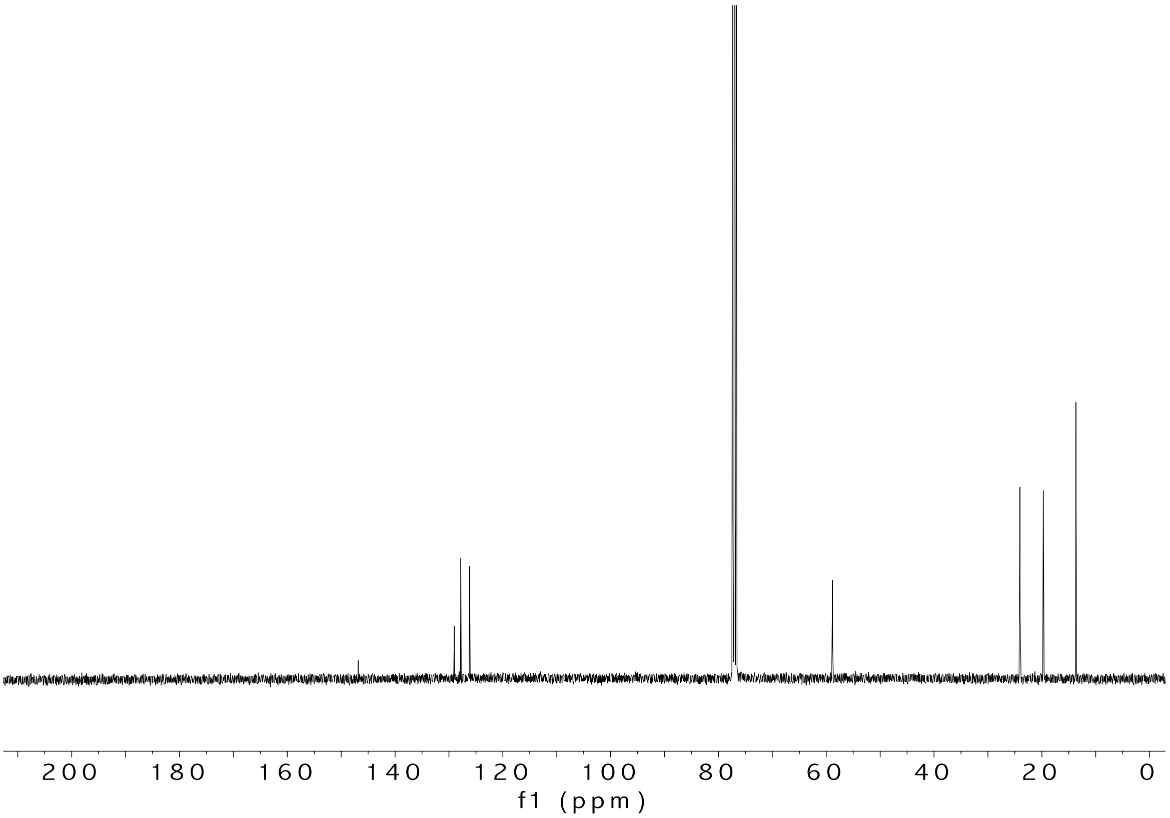

tert-Butyl ((mesitylsulfonyl)oxy)carbamate 7a

400 MHz, $CDCl_3$

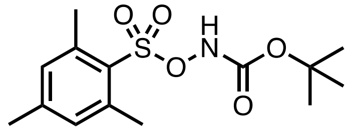

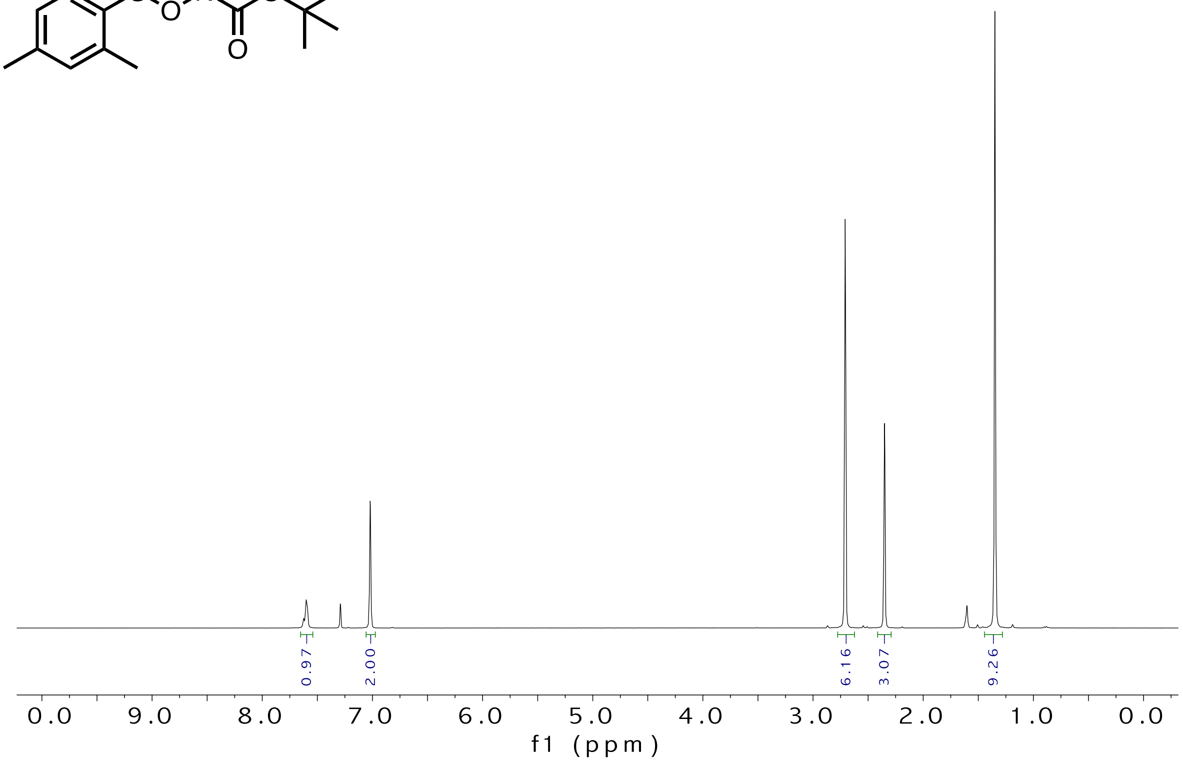

101 MHz, $CDCl_3$

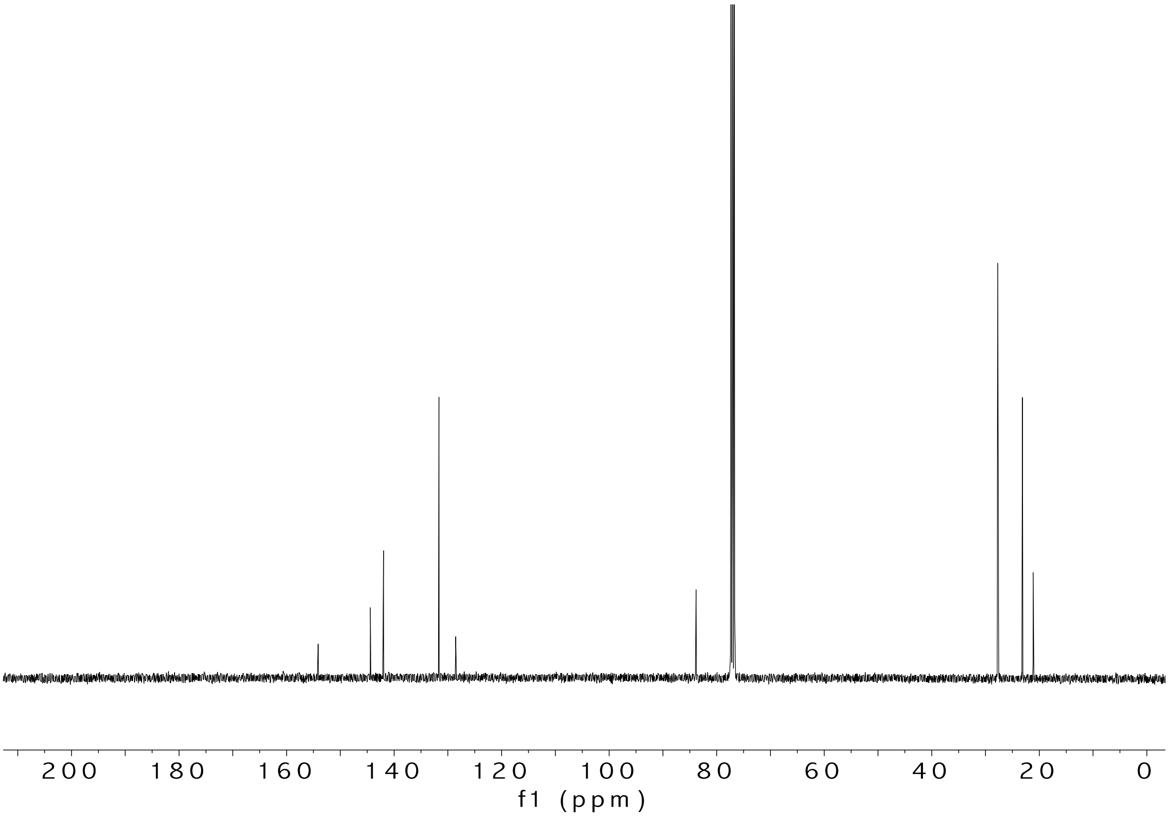

***tert*-Butyl ((methylsulfonyl)oxy)carbamate 7b**

400 MHz, $CDCl_3$

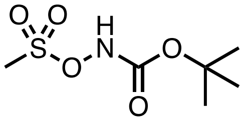

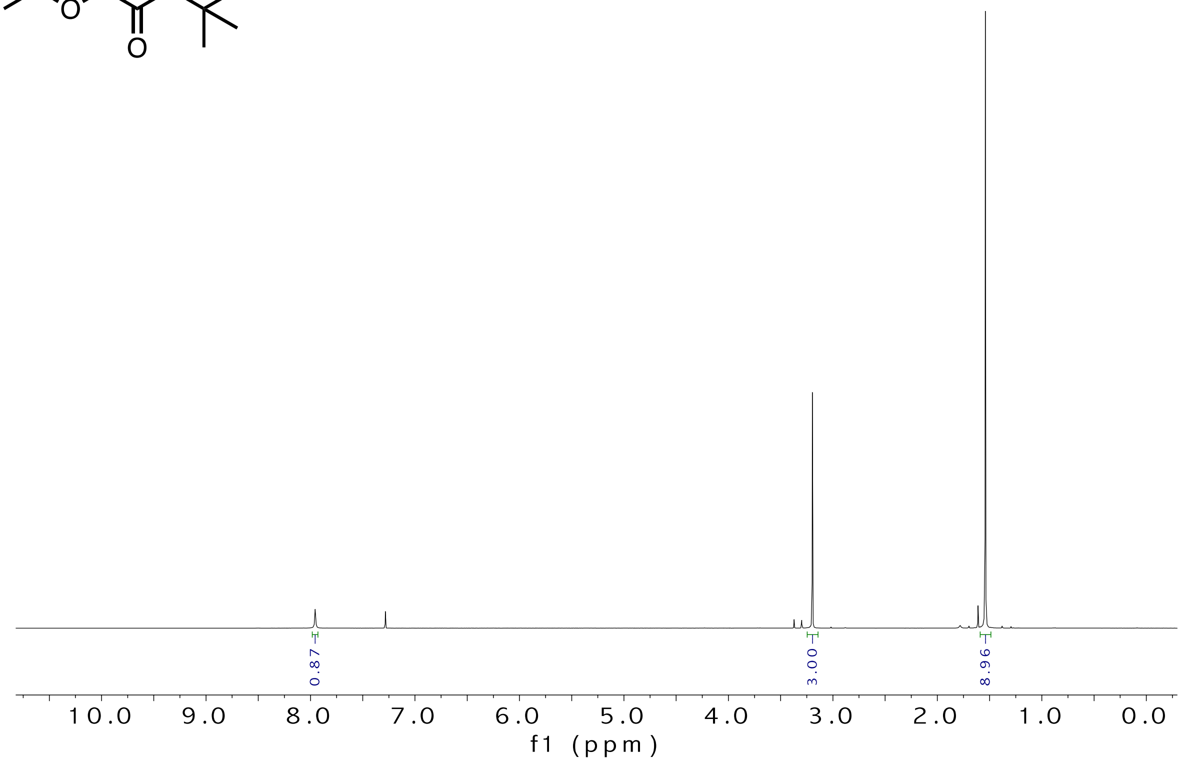

101 MHz, $CDCl_3$

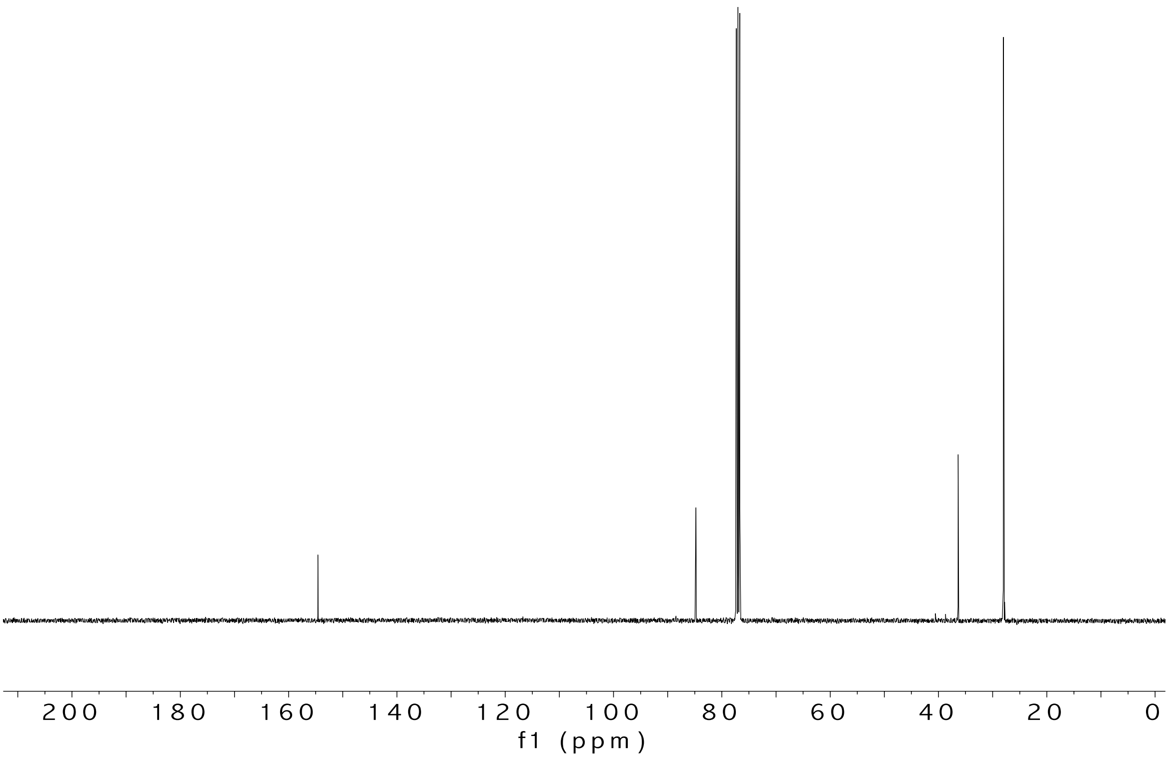

Supplement: Supplementary file 1 — Supporting Information [file ANIE-61-0-s001.pdf]
